# Identification and characterization of non-coding RNA networks in infected macrophages revealing the pathogenesis of *F. nucleatum*-associated diseases

**DOI:** 10.1186/s12864-022-09052-z

**Published:** 2022-12-13

**Authors:** Jieyu Zhou, Lin Liu, Peiyao Wu, Lei Zhao, Yafei Wu

**Affiliations:** 1 State Key Laboratory of Oral Diseases, National Clinical Research Center for Oral Diseases, West China Hospital of Stomatology, Sichuan University, Chengdu, China; 2 Department of Periodontics, West China Hospital of Stomatology, Sichuan University, Chengdu, China

**Keywords:** *F. nucleatum*, Macrophage, High-throughput sequencing, Competitive endogenous RNA, Non-coding RNA, Immune regulation

## Abstract

**Background:**

*F. nucleatum*, as an important periodontal pathogen, is not only closely associated with the development of periodontitis, but also implicated in systemic diseases. Macrophages may act as an important mediator in the pathogenic process of *F. nucleatum* infection. As non-coding RNAs (ncRNAs) have attracted extensive attention as important epigenetic regulatory mechanisms recently, we focus on the competing endogenous RNA (ceRNA) regulatory networks to elucidate the pathogenesis of *F. nucleatum*-associated diseases.

**Results:**

We screen abnormally expressed mRNAs, miRNAs, lncRNAs and circRNAs in macrophages after *F. nucleatum* infection via the whole transcriptome sequencing technology, including 375 mRNAs, 5 miRNAs, 64 lncRNAs, and 180 circRNAs. The accuracy of RNA-seq and microRNA-seq result was further verified by qRT-PCR analysis. GO and KEGG analysis show that the differentially expressed genes were mainly involved in MAPK pathway, Toll-like receptor pathway, NF-κB pathway and apoptosis. KEGG disease analysis reveals that they were closely involved in immune system diseases, cardiovascular disease, cancers, inflammatory bowel disease (IBD) et al. We constructed the underlying lncRNA/circRNA-miRNA-mRNA networks to understand their interaction based on the correlation analysis between the differentially expressed RNAs, and then screen the core non-coding RNAs. In which, AKT2 is controlled by hsa_circ_0078617, hsa_circ_0069227, hsa_circ_0084089, lncRNA NUP210, lncRNA ABCB9, lncRNA DIXDC1, lncRNA ATXN1 and lncRNA XLOC_237387 through miR-150-5p; hsa_circ_0001165, hsa_circ_0008460, hsa_circ_0001118, lncRNA XLOC_237387 and lncRNA ATXN1 were identified as the ceRNAs of hsa-miR-146a-3p and thereby indirectly modulating the expression of MITF.

**Conclusions:**

Our data identified promising candidate ncRNAs responsible for regulating immune response in the *F. nucleatum*-associated diseases, offering new insights regarding the pathogenic mechanism of this pathogen.

**Supplementary Information:**

The online version contains supplementary material available at 10.1186/s12864-022-09052-z.

## Background

*Fusobacterium nucleatum* (*F. nucleatum*) is an obligate gram-negative anaerobic bacterium, generally resident in the periodontal microenvironment, which is linked closely to the pathogenesis of periodontitis (PD). In addition, *F. nucleatum* can also be isolated from other tissues, organs or abscesses throughout the body as the most prevalent oral species [1–4]. Some research has shown that *F. nucleatum* could disseminate to the uterus, placental and fetal tissues through bacteremia, causing intrauterine infection, which is related to Adverse pregnancy outcome (APO) [5, 6]. Moreover, *F. nucleatum* has been recently reported linked to some inflammatory diseases, including inflammatory bowel disease (IBD), atherosclerosis, rheumatoid arthritis (RA), and tumors such as colon cancer, and other systemic diseases [2–4, 7]. The pathogenesis can be explained as follows that *F. nucleatum* has the ability to adhere to the surface of host cells such as monocytes/macrophages, polymorphonuclear leukocytes, epithelial cells, fibroblasts, endothelial cells, through FadA adhesin/invasion to induce a series of host reactions [8].

The role of macrophages in the body is diverse. Monocytes and macrophages are the main attributes of innate immunity, playing a major role in response to chronic infection [9, 10]. Multiple studies showed that the role of the macrophages might be significant during the development of periodontitis [11]. Macrophages are involved in the formation of atherosclerotic lesion development as well, and macrophage accumulation within the vascular wall is a hallmark of atherosclerosis [12]. Additionally, infiltrated macrophages are important cellular components in tumor microenvironment which can participate in pathological processes including tumor invasion and metastasis, angiogenesis and immunosuppression by secreting a variety of cytokines [13, 14].

The reported studies revealed that *F. nucleatum* is an intracellular pathogen that can diffuse and survive inside the macrophages causing impairment of blood lymphocytes’ function and attenuating apoptotic process by inducing indoleamine 2,3-dioxygenase (IDO) expression, which enhanced immunosuppression in the tumor microenvironment [15]. *F. nucleatum* and the LPS or OMVs derived from *F. nucleatum* could cause proinflammation and activate tumor-associated profiles in the human macrophages derived from monocytes resulting in the onset of colorectal cancer (CRC) [16]. *F. nucleatum* causes infiltration of macrophages via the activation of CCL20 and simultaneously induces M2 polarization of macrophage, resulting in the enhancement of CRC metastasis [17]. Some evidence revealed that *F. nucleatum* accumulated heavily in the intestine of ulcerative colitis (UC) sufferers along with IFN-γ secretion and M1 macrophages skewing [18]. *F. nucleatum* stimulates lipid uptake in macrophages by modulating FABP4 expression, and further accelerates the progression of atherosclerosis [19]. Thus, macrophages participate crucially in the pathological process of *F. nucleatum*-associated infection.

Recently non-coding RNAs (ncRNAs) such as microRNAs (miRNAs), circular RNAs (circRNAs), and long non-coding RNAs (lncRNAs) have attracted extensive attention as important epigenetic regulatory mechanisms. They have shown a considerable role in the regulation of multiple biological processes counting responses of the immune system against pathogenic infection [20–23]. MiRNAs can inhibit the translation of the complementary mRNAs, whereas lncRNAs/circRNAs can bind and modulate the function of miRNAs [24]. It is thought that there exist shared miRNA response elements (MREs) on different RNA, which are the key junction points of the competing endogenous RNA (ceRNA) network inside the cell and extremely regulate the expression of genes and responses of host cells towards different types of stimuli. It is further suggested that both the circRNA-miRNA-mRNA ceRNA and lncRNA-miRNA-mRNA contribute to the responses of innate and adaptive immunity against pathogens. Moreover, the role of miRNAs is central in the modulation of interactions between host and pathogen [25]. For instance, different types of miRNAs (such as miRNA-372-3p, miR-21, miR-603, miR-296-5p, and miR-495-3p) and lncRNAs (such as ENO-ITI and MIR4435-2HG) had been found to have links with the responses against infection of *F. nucleatum* and further implicated in various diseases process such as oral epithelial carcinoma, colorectal cancers, enterocolitis [26–30]. Despite there are a few studies on non-coding RNAs linking the pathogenic mechanism between *F. nucleatum* and cancers or enteritis as described above. However, as per our knowledge, there is no study about the exploration of roles of ceRNA networks in the regulation of *F. nucleatum* infection in macrophages to clarify the pathogenic mechanism in associated diseases.

In this study, we utilized a co-culture infection model between *F. nucleatum* and macrophage to study the differentially expressed profiles of miRNAs, lncRNAs, mRNAs, and circRNAs, systematically, using high-throughput sequencing. After that, we established the regulatory networks of circRNA-miRNA-mRNA and lncRNA-miRNA-mRNA for evaluating the interactions between mRNAs and ncRNAs under infection. The results of our study will lay out a precious foundation for the research of the process mediating *F. nucleatum*-related diseases.

## Results

### Expression profiles of mRNAs in *F. nucleatum* infection

**Fig. 1:**
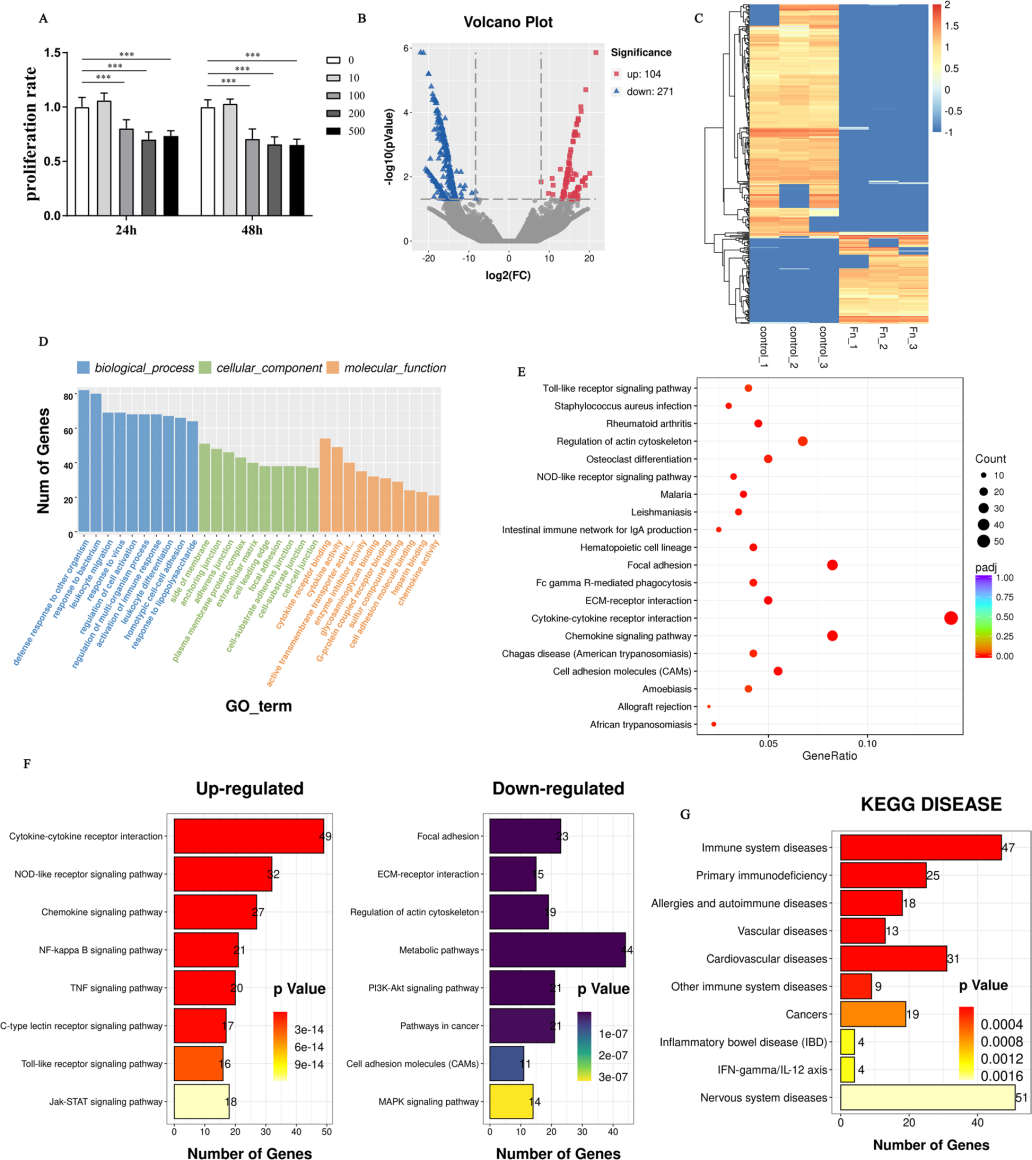
Analysis of differentially expressed mRNAs. **A** Cell viability of dTHP-1 cells were measured by MTT assay after infection with *F. nucleatum* at the indicated MOI (0, 10,100, 200 and 500) for 24 h or 48 h. The one-way ANOVA with Tukey’s post hoc Tests, ***P < 0.001, vs control group (MOI = 0). **B** Volcano plot depicting mRNAs. Gray dots represent RNAs not significantly differentially expressed (*Padj* > 0.05) and the other dots represent RNAs differentially expressed (*Padj* < 0.05). **C** Hierarchical cluster analysis of the differential mRNA expression profile. Yellow and blue represented increased and decreased expression, respectively. Each sample was indicated by a single column, and each RNA was denoted by a single row of colored boxes. **D** GO analysis results for the differentially expressed mRNAs (DEGs). **E** KEGG analysis for differentially expressed mRNAs (DEGs). **F** Top 8 KEGG pathways that the up-regulated and down-regulated genes involved in, respectively. **G** KEGG DISEASE analysis for differentially expressed mRNAs (DEGs)

To explore the infection effect of *F. nucleatum* on the viability of macrophages, dTHP1 cells were co-incubated with live *F. nucleatum* at different MOI for 24 h and 48 h. MTT results did not show a significant difference in the viability of dTHP1 cells at the MOI of 10 compared with the control group. While the MOI rose up to 100 or more, there was a significant inhibition effect on cell viability at a dose-dependent manner (Fig. 1A). Based on this, we chose the MOI of 100 and infection time for 24 h in the following co-culture model.

We performed RNA-seq between the control and infected samples. *Padj* < 0.05 and |fold change| > 2 was identified as significantly expressed. Accumulatively, 375 differentially expressed mRNAs were identified under infection, including 104 up-regulated and 271 down-regulated mRNAs (Fig. 1B) (Supplementary Table 2). To evaluate the differential expression of mRNAs, we performed a hierarchical cluster analysis (Fig. 1C). Of which, Top ten differentially expressed genes (DEGs) based on significant value were shown in Table 1, which were all upregulated, and the gene IDO1 ranked first. To examine the function of the genes expressed differentially during the infection process, we carried out the analysis of GO (Fig. 1D) and KEGG pathway (Fig. 1E) on them. As shown in Fig. 1F, the up-regulated genes were significantly involved in cytokine-cytokine receptor interaction, chemokine signaling pathway, NOD-like receptor signaling pathway and NF-κB signaling pathway, etc. The down-regulated genes played a considerable role in focal adhesion, ECM-receptor interaction, regulation of actin cytoskeleton, metabolic pathways, PI3K-Akt signaling pathway and pathways in cancer, etc. Many of them are important for immune system diseases, vascular diseases, cardiovascular diseases, cancers and inflammatory bowel disease (IBD) according to the disease KEGG analysis (Fig. 1G), which confirmed the disease above were associated with *F. nucleatum* infection. And these differentially expressed genes (DEG) were recognized as principal modulators in the process of *F. nucleatum*-associated diseases (Supplementary Table 3).

**Table 1:** Top 10 differentially expressed genes based on *Padj* during infection process

Gene_id	Gene_name	Status	***Padj***	log2FoldChange
ENSG00000131203	IDO1	Up	3.95E-98	9.367061
ENSG00000110944	IL23A	Up	1.80E-88	7.804578
ENSG00000156234	CXCL13	Up	1.64E-76	16.73139
ENSG00000134321	RSAD2	Up	3.81E-64	6.480449
ENSG00000137959	IFI44L	Up	1.52E-54	5.741726
ENSG00000121594	CD80	Up	5.08E-50	6.239785
ENSG00000117228	GBP1	Up	1.88E-46	5.654452
ENSG00000185745	IFIT1	Up	6.49E-43	4.848847
ENSG00000132274	TRIM22	Up	2.11E-42	4.901086
ENSG00000136688	IL36G	Up	3.74E-42	8.402793

**Fig. 2:**
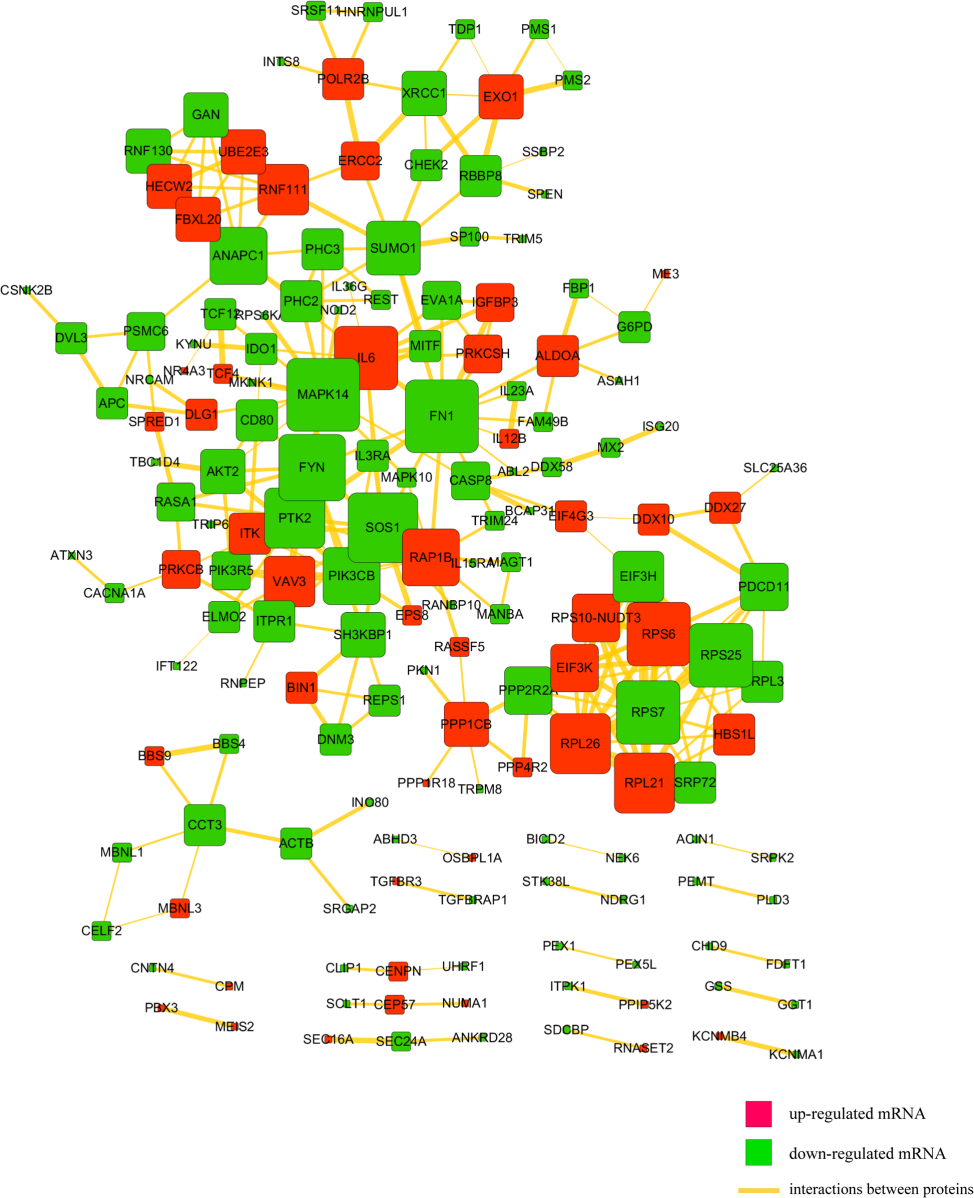
Protein and protein interaction (PPI) network for differentially expressed mRNAs. Square nodes represent mRNAs; Red shows up-regulation and green shows down-regulation. Yellow lines represent the interactions between proteins

Then we uploaded the DEGs to STRING and constructed the protein-protein interaction (PPI) network, 281 high-confidence interaction relationships (combined score > 0.7) were found to be present in 165 DEGs (Fig. 2). In this network, we found that MAPK14 genes encode the highest degree of protein nodes as cores of the network (Supplementary Table 4). MAPK14 could closely interact with AKT2, MITF, MKNK1, MAPK10, IL6 and CASP8.

### Analysis of the miRNA expression profiles during *F. nucleatum* infection

**Fig. 3:**
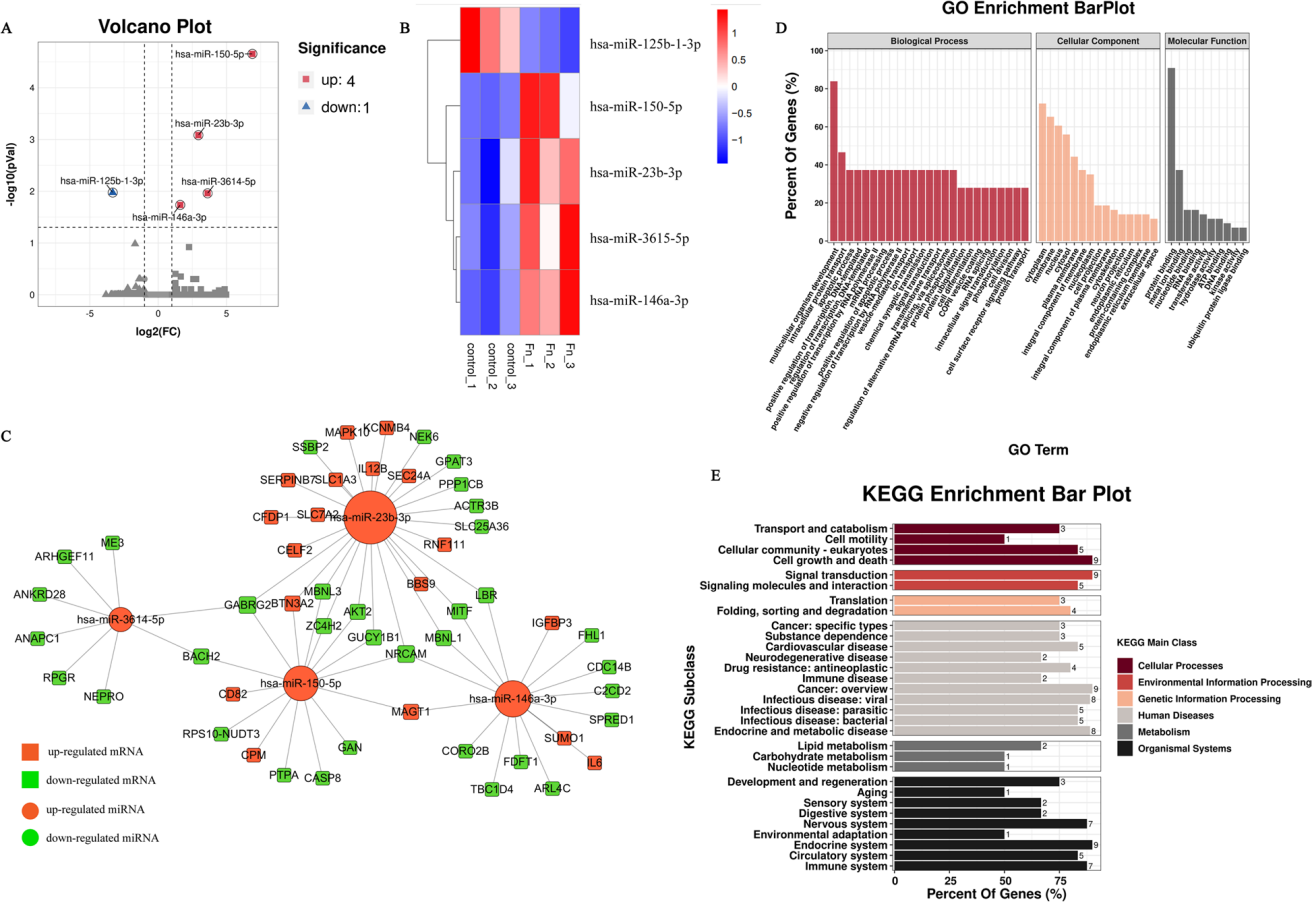
Analysis of DE-miRNAs in *F. nucleatum*-infected macrophages at 24 h. **A** Volcano plot depicting miRNAs. **B** Heat map of DE-miRNA expression profile. **C** The interaction network of DE-miRNAs and their target DEGs in infected macrophages. **D** GO analysis for DE-miRNA targets. **E** KEGG analysis for DE-miRNA targets

From the profile data of miRNA-seq, overall, 1481 mature miRNAs have been identified, including 1386 known miRNAs and 95 novel miRNAs (Supplementary Table 5). We found 5 miRNAs differentially expressed in infected macrophages with *Padj <* 0.05 and |FC| > 2, of which, 4 miRNAs upregulated, and 1 miRNA downregulated (Fig. 3A) (Supplementary Table 6). A heat map of DE miRNAs has been present in Fig. 3B. The differentially expressed microRNAs based on *Padj* were shown in Table 2. Furthermore, there were 68 interaction pairs predicted between 55 differentially expressed targets (DEGs) and 4 DE-miRNAs in infected macrophages by TargetScan and miRanda softwares (Supplementary Table 7). The DE-miRNAs-target DEGs pairs were visualized by Cytoscape software (Fig. 3C). GO and pathway analyses were performed to understand the functions of these 55 DE-miRNAs target DEGs. As shown in Fig. 3D, *F. nucleatum* infection were associated with apoptotic process (GO:0006915), vesicle-mediated transport (GO:0016192), ion transport (GO:0006811), cell junction (GO:0030054), etc. As can be seen from Fig. 3E, the KEGG pathway analysis were enriched in pathways in cancer, insulin resistance, Toll-like receptor signaling pathway, c-type lectin receptor signaling pathway, vascular smooth muscle contraction, Jak-STAT signaling pathway, etc.

**Table 2:** The differentially expressed miRNAs based on *Padj* value during infection process

miRNA_id	Status	***Padj***	log2FoldChange
hsa-miR-150-5p	Up	2.22E-05	6.8954
hsa-miR-23b-3p	Up	0.00082502	2.9443
hsa-miR-125b-1-3p	Down	0.010617	−3.3273
hsa-miR-3614-5p	Up	0.010966	3.6175
hsa-miR-146a-3p	Up	0.018427	1.6148

**Fig. 4:**
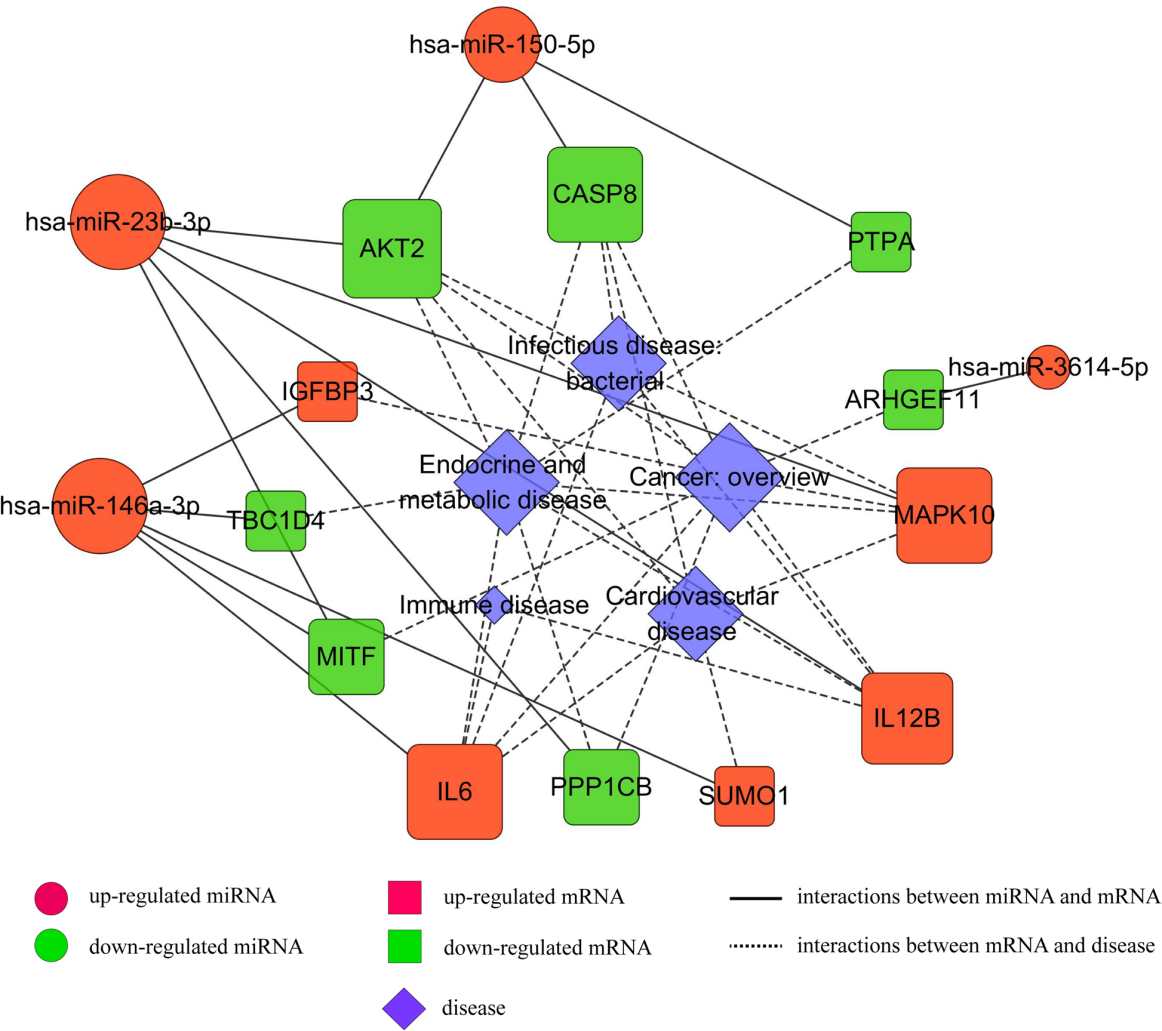
The interaction network of DE-miRNAs and their disease-related targets in infected macrophages

In addition, these DE-miRNA-targets were significantly implicated in various systemic diseases, such as cardiovascular diseases, cancer, immune disease, infectious disease, and endocrine and metabolic disease. These DE-miRNAs-target disease-related DEGs pairs were exhibited by Cytoscape software (Fig. 4). The results given above suggested that the genes of AKT2, MAPK10, CASP8, MITF, and the miRNAs of miR-150-5p, miR-3614-5p, miR-23b-3p, miR-146a-3p were largely involved in the disease process induced by *F. nucleatum* infection.

### Identification and analysis of IncRNA and circRNA expression profiles

**Fig. 5:**
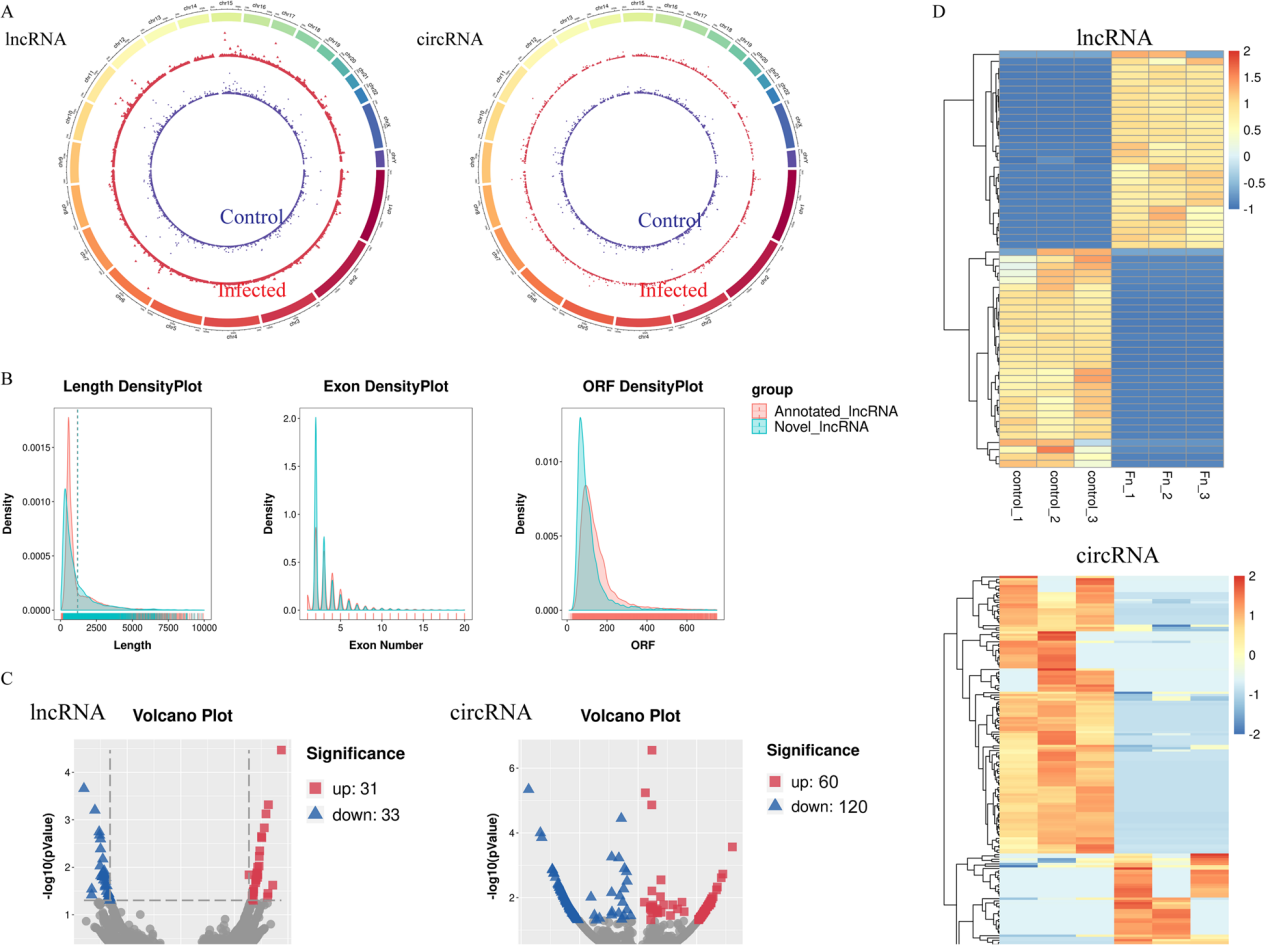
Differential expression of lncRNAs and circRNAs during *F. nucleatum* infection. **A** Circos plots showing all of the lncRNAs and circRNAs from control (the inner blue ring) and infected macrophages (the outer red ring). **B** Distribution of transcript lengths, open reading frames (ORF) lengths, and exon numbers in the novel and annotated lncRNAs in macrophages. **C** Differentially expressed lncRNAs (the left panel) and circRNAs (the right panel) are displayed by Volcano plot. The red spots represent up-regulation and blue spots represents down-regulation. **D** Heat map of lncRNAs profile (the left panel) and circRNAs profile (the right panel) with |Foldchange| > 2 and *Padj* < 0.05. Each row refers to one RNA, and each column refers to one sample. Red indicates up-regulation; blue indicates downregulation. The differentially expressed lncRNAs and circRNAs are clearly self-segregated into clusters

In our present research, we totally identified 63,335 lncRNAs, including 4490 novel lncRNAs which were filtered and predicted using Cuffmerge software following assembling transcripts via StringTie (Supplementary Table 8). We screened and named the candidate novel lncRNAs according to its position relationship with the coding gene and the HGNC naming guidelines for long noncoding RNAs. We also identified 3550 circRNAs totally, of which 1228 were novel circRNAs (Supplementary Table 9). The lncRNAs and circRNAs from two groups were widely scattered across the 24 pairs of human chromosomes according to their loci (Fig. 5)A. Comparative analysis of genomic architecture was conducted in Fig. 5B. The sequence length analysis showed that the lengths of lncRNA were mostly ranged from 200 to 1000 nucleotides. Moreover, the sequence of ORF (open reading frame) in lncRNA transcripts was mainly ranged from 50 to 200 nucleotides and the number of exons was mostly ranged from 1 to 8. Additionally, we compared the predicted novel lncRNA with annotated lncRNA, and found that the characters of them were similar in the density distribution of transcript length, exon number and ORF length. By analyzing the expression profile data of lncRNA transcripts, 64 lncRNAs were recognized as highly expressed in infection group compared to control group (*Padj* < 0.05 and |Fold change| > 2), consist of 31 upregulated and 33 downregulated lncRNAs (Fig. 5C, the left panel) (Supplementary Table 10). For circRNAs analysis, 180 circRNAs were identified to be aberrantly expressed between control and infected macrophages (*Padj* < 0.05 and |Fold change| > 2), including 60 upregulation and 120 downregulation as shown in the Volcano plot (Fig. 5C, the right panel) (Supplementary Table 11). Based on the *Padj*, top ten DE-lncRNAs and DE-circRNAs are shown in Tables 3 and 4, accordingly. The expression patterns of DE-lncRNAs and DE-circRNAs were distinctly shown in hierarchical clustering (Fig. 5D).

**Table 3:** Top 10 differentially expressed lncRNAs based on *Padj* during infection process

Transcripts_id	Transcripts _name	Status	***Padj***	log2FoldChange
ENST00000495849	STAT4–210	Up	3.40E-05	18.76018
TCONS_00089684	LINC925	Down	0.00022	−18.0714
TCONS_00474177	LINC4405	Up	0.000481	16.33195
ENST00000618522	DIXDC1–209	Down	0.000627	−16.0245
ENST00000332587	LINC00158–201	Up	0.000753	15.89057
TCONS_00266806	LINC2991	Up	0.00148	15.57846
TCONS_00118350	LINC1531	Down	0.001792	−15.2329
TCONS_00094346	OTOGL-OT1	Down	0.00211	−15.1471
ENST00000558942	ISG20–206	Up	0.002252	15.04127
ENST00000485300	IL6–209	Up	0.00238	15.10388

**Table 4:** Top 10 differentially expressed circRNAs based on *Padj* during infection process

CircRNA_id	Status	***Padj***	log2FoldChange
hsa_circ_0025765	Down	4.53E-06	−7.4898
novel_circ_0002171	Down	3.58E-05	−1.1504
novel_circ_0003369	Down	9.91E-05	−6.6856
hsa_circ_0039353	Down	0.000139	−6.5904
hsa_circ_0083619	Up	0.000271	6.447
hsa_circ_0017636	Down	0.000553	−1.8093
hsa_circ_0002138	Down	0.000591	−1.3191
hsa_circ_0006999	Down	0.00133	−5.8699
hsa_circ_0001821	Down	0.001388	−5.8561
hsa_circ_0032029	Down	0.001744	−5.7679

### Interaction network among lncRNAs/circRNAs, miRNAs, and mRNAs construction

**Fig. 6:**
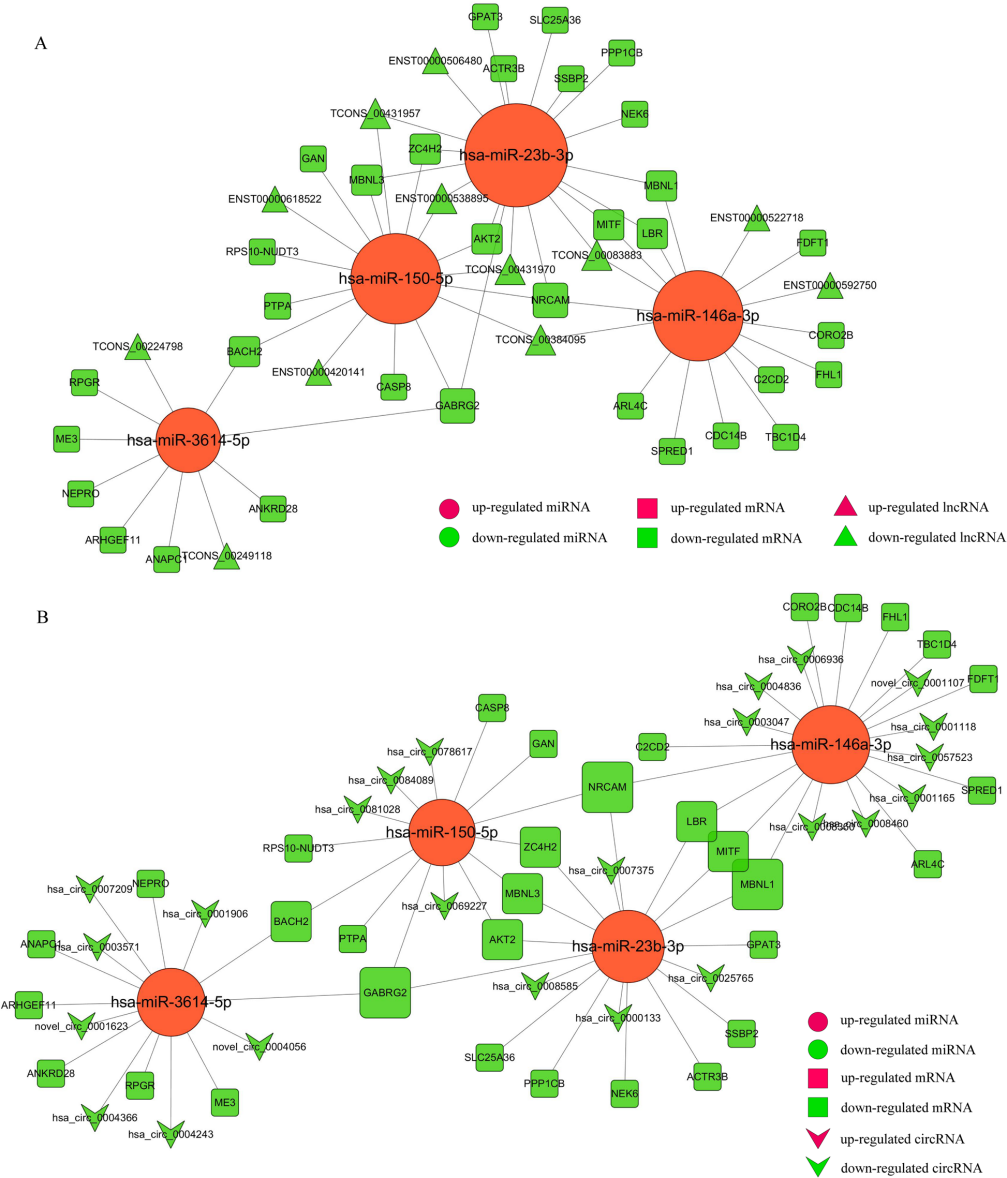
The view of DE lncRNA/DE circRNA-DE miRNA-DE mRNA triple network. **A** The ceRNA interaction network of DE-lncRNA-DE-miRNA-DE-mRNA. Square nodes represent mRNAs; Circular nodes represent miRNAs; and regular triangle nodes represent lncRNAs. Red shows up-regulation and green shows down-regulation. The network includes 4 up-regulated miRNAs, 12 down-regulated lncRNAs, 33 down-regulated mRNAs and 61 edges. **B** The ceRNA interaction network of DE-circRNA-DE-miRNA-DE-mRNA. Square nodes represent mRNAs; Circular nodes represent miRNAs; and V-shape nodes represent circRNAs. Red shows up-regulation and green shows down-regulation. The network includes 4 up-regulated miRNAs, 24 down-regulated circRNAs, 33 down-regulated mRNAs and 68 edges

Some lncRNAs and circRNAs could suppress the binding of miRNA with target genes in post-transcriptional regulation, serving as miRNA “sponges”. We constructed ceRNA networks, including the integration of matched expression profiles of lncRNAs, circRNAs, miRNAs and mRNAs, to systematically explore the effect of dynamic changes in ceRNA regulation on gene expression in macrophages with *F. nucleatum* infection. TargetScan and miRanda softwares were used to predict DE-circRNA–miRNA and DE-lncRNA-miRNA target pairs, respectively. By integrating with DE-miRNAs-DEGs pairs (Supplementary Table 7) and screening the common miRNAs, and mRNAs, lncRNAs, circRNAs that have targeted and negatively correlated relationship with the common miRNAs, lncRNA-miRNA-mRNA (including 4 DE-miRNAs, 12 DE-lncRNAs, and 33 DE-mRNAs) (Fig. 6A) and circRNA-miRNA-mRNA (including 4 DE-miRNAs, 24 DE-circRNAs, and 33 DE-mRNAs) (Fig. 6B) regulatory co-expression networks were constructed.

**Fig. 7:**
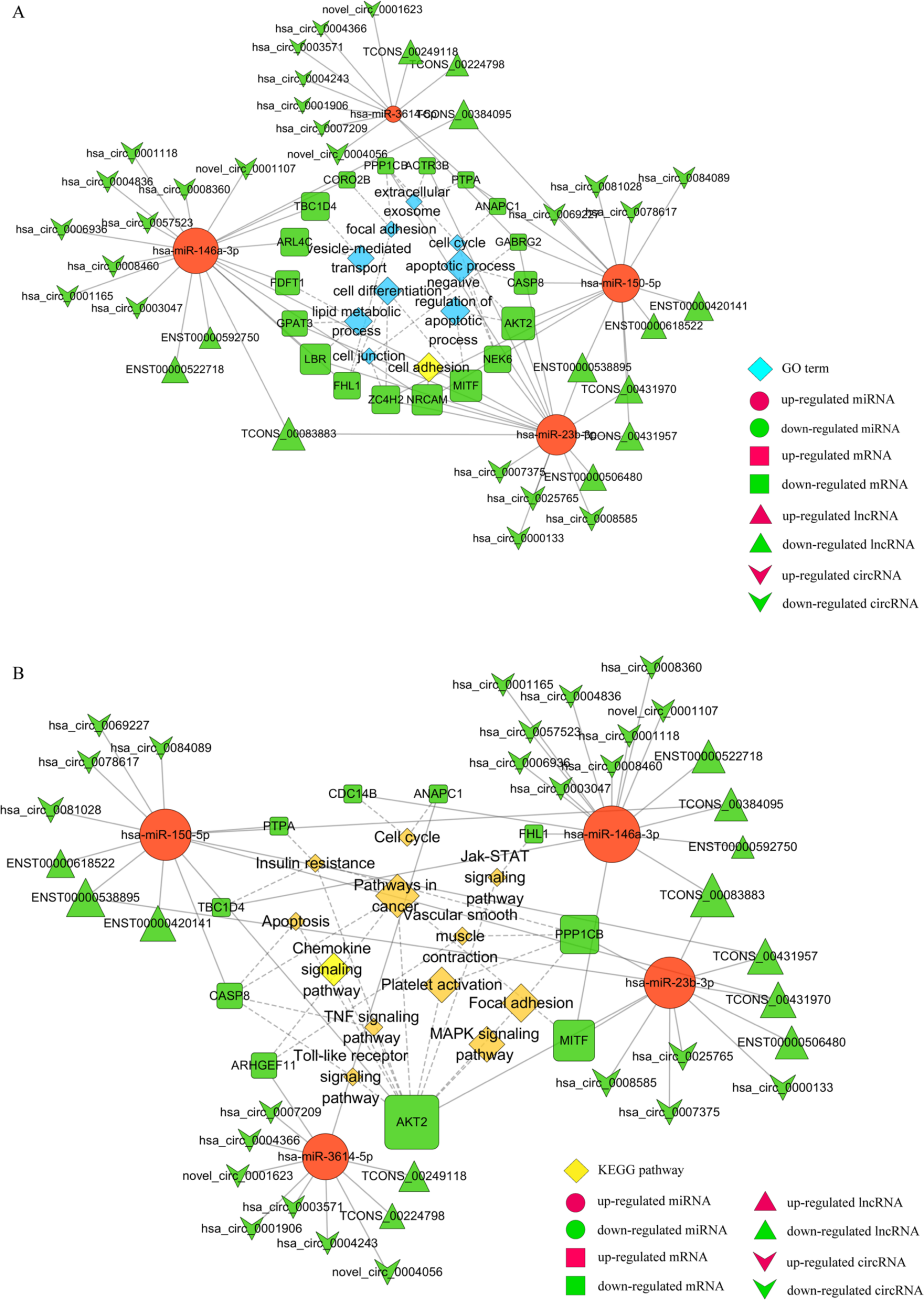
Dysregulated function analysis of ceRNA interaction networks during *F. nucleatum* infection. **A** The ceRNA network of lncRNAs/circRNAs-miRNAs-mRNAs significantly participated in GO:0006915(apoptotic process), GO:0043066(negative regulation of apoptotic), GO:0007155(cell adhesion), GO:0030154(cell differentiation), GO:0006629(lipid metabolic process), GO:0016192(vesicle-mediated transport), GO:0005925 (focal adhesion), GO:0070062(extracellular exosome), GO:0007049(cell cycle) and GO:0030054(cell junction). **B** The ceRNA network of lncRNAs/circRNAs-miRNAs-mRNAs significantly participated in insulin resistance, pathways in cancer, focal adhesion, platelet activation, chemokine signaling pathway, MAPK signaling pathway, cell cycle, TNF signaling pathway, Jak-STAT signaling pathway, apoptosis, Toll-like receptor signaling pathway and Vascular smooth muscle contraction. Square nodes represent mRNAs; Circular nodes represent miRNAs; Regular triangle nodes represent lncRNAs; V-shape nodes represent circRNAs; Blue diamond nodes represent GO terms; Yellow diamond nodes represent KEGG pathways. Red shows up-regulation and green shows down-regulation

From the GO functional analysis of these DE-mRNAs, we obtained GO:0006915 (apoptotic process), GO:0043066 (negative regulation of apoptotic), GO:0007155 (cell adhesion), GO:0030154 (cell differentiation), GO:0006629 (lipid metabolic process), GO:0016192 (vesicle-mediated transport), GO:0005925 (focal adhesion), GO:0070062(extracellular exosome), GO:0007049(cell cycle) and GO:0030054(cell junction), which played major pathogenic roles during *F. nucleatum* infection. The complex networks involved in these GO terms including 18 DE-mRNAs, 4 miRNAs, 12 lncRNAs, and 24 circRNAs are displayed by the cytoscape software in Fig. 7A. From KEGG analysis of these DE-mRNAs, insulin resistance, the pathways in cancer, focal adhesion, platelet activation, chemokine signaling, MAPK signaling pathway, cell cycle, TNF signaling pathway, Jak-STAT signaling pathway, apoptosis, Toll-like receptor signaling pathway and Vascular smooth muscle contraction were closely related to the process of *F. nucleatum*-associated diseases. The ceRNA networks associated with the above pathways were shown in Fig. 7B, including 10 DE-mRNAs, 4 miRNAs, 12 lncRNAs, and 24 circRNAs.

### Validation of the RNA-seq and miRNA-seq data by qRT-PCR

**Fig. 8:**
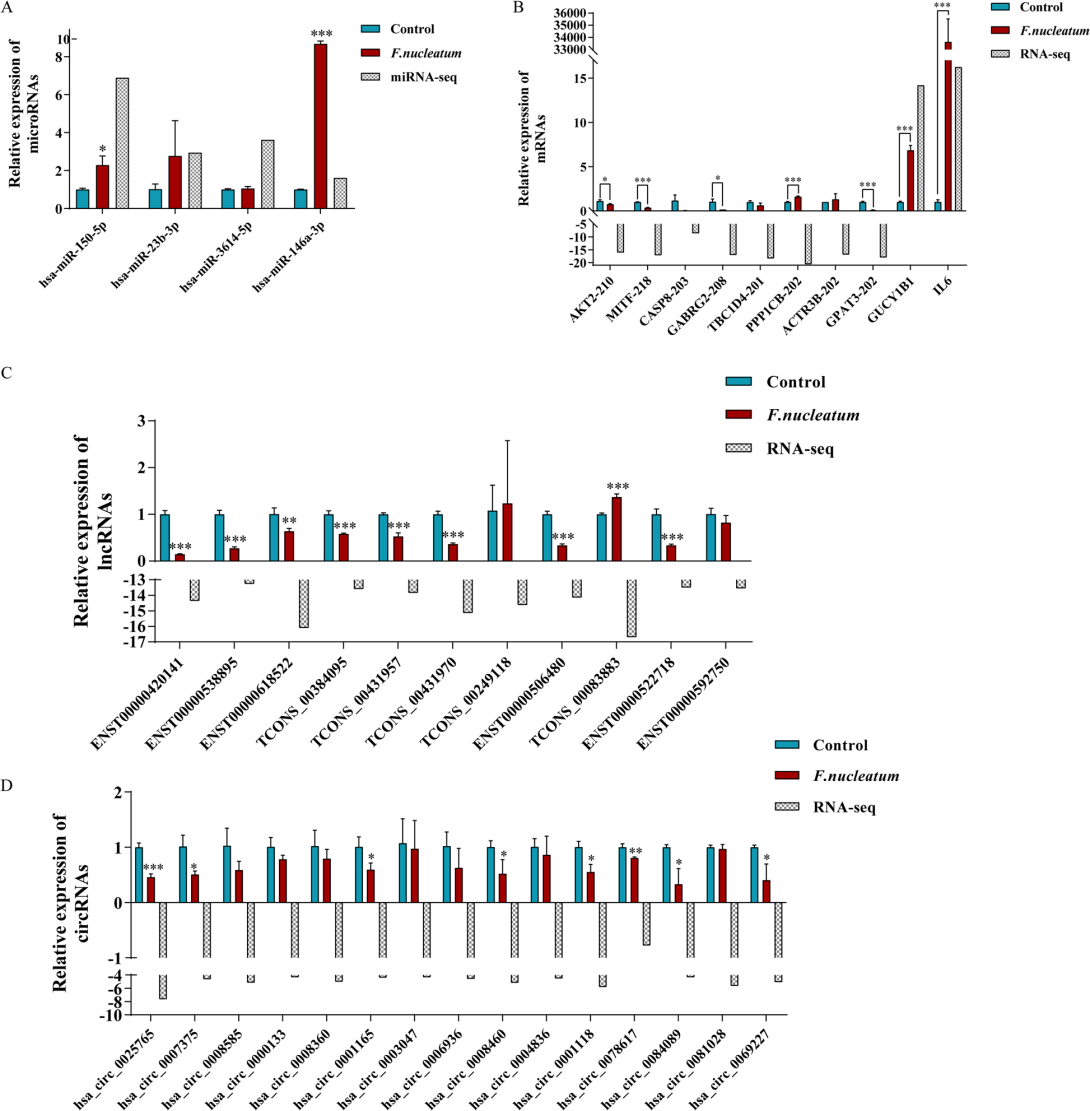
Validation of RNA-seq data and miRNA-seq by quantitative real-time PCR (qPCR). The qPCR results of selected (**A**) miRNAs, (**B**) mRNAs, (**C**) lncRNAs and (**D**) circRNAs in macrophages. *P < 0.05, **P < 0.01, ***P < 0.001 vs control group. Student’s t-test

The qRT-PCR analysis was conduct to verity the results of the RNA-seq and miRNA-seq. We selected the core differentially expressed miRNAs, mRNAs, lncRNAs and circRNAs based on ceRNA network and enrichment analysis established in Figs. 6 and 7, with readcount > 5 in at least one of the samples. Most of the qPCR data were consistent with the RNA-seq and miRNA-seq results (Fig. 8), indicating that the RNA-seq and miRNA-seq results were accurate. In this view, this finding offers compelling evidence that these mRNAs and non-coding RNAs may be employed in the future to explore the pathogenesis of *F. nucleatum* infection. We finally inference AKT2 and MITF and their upstream regulation network may play the critically regulatory roles in the immune process during *F. nucleatum* infection.

## Discussion

In the current study, we explored the function of non-coding RNAs in *F. nucleatum*-infected macrophages to characterize the pathogenesis of *F. nucleatum*-associated diseases. Through RNA-seq analysis, we screened 375 DEGs (*P <* 0.05 and |fold change| > 2) between the control and infected groups. Of which, indoleamine 2,3-dioxygenase (IDO) was identified to be the significantly differentially expressed gene (Table 1), which has been demonstrated to have an immunomodulatory role in the functions of macrophage by previous studies. Elevated activity of IDO is commonly linked with infectious diseases and tumors [31, 32]. During *F. nucleatum* infection, IDO is confirmed to have an important role in mediating proliferation of *F. nucleatum* inside the macrophages, further impairing the function of peripheral blood lymphocytes and induced immune tolerance, which may serve as critical pathogenesis for accelerating colorectal cancer growth in the tumor microenvironment [15]. We further conducted KEGG disease analysis to define these differentially expressed genes (DEGs), and found they were extensively involved in immune system diseases, vascular diseases, cardiovascular diseases, cancers, and inflammatory bowel disease (IBD) (Fig. 1F), which have been reported as *F. nucleatum*-associated diseases [33–36]. Therefore, it is possible that the dysregulation of DEGs may promote the pathogenic process of *F. nucleatum*-associated diseases.

Additionally, to explore the interrelationship of 375 differentially expressed genes (DEGs) during infection, we established a PPI regulatory network (Fig. 1G). Of which, MAPK14 were identified as the core of the PPI network, and well correlated with MITF (combined score 0.965) and AKT2 (combined score 0.752), which may serve as critical roles during *F. nucleatum*-induced pathological process.

After construction of the ceRNA network of lncRNAs/circRNAs-miRNAs-mRNAs, we found Akt2 was significantly participated in apoptotic process, insulin resistance, pathways in cancer, focal adhesion, platelet activation, chemokine signaling pathway, MAPK signaling pathway, TNF signaling pathway, Jak-STAT signaling pathway, Toll-like receptor signaling pathway, and implicated in cardiovascular diseases, cancer, infectious disease, and endocrine and metabolic disease. MITF was significantly participated in cell differentiation, negative regulation of apoptotic process, pathways in cancer, and implicated in cancer. By qRT-PCR analysis, we found that they both were significantly reduced, which was consistent with the RNA-seq results.

Akt2 (serine/threonine protein kinase 2) is critically important for cell survival, proliferation, and migration which are promoted by phosphorylation of serine/threonine in the multiple substrates of the downstream process [37]. Macrophages are noticeably resistant to stimuli of apoptosis which make them able to survive. This ability is mainly due to PI3K/Akt pathway activity. Besides that, it has been found that the phosphatidylinositol 3-kinase (PI3K)/Akt pathway is crucial for the migration and polarization of macrophages [38–40] In periodontitis, Akt2/JNK1/2/c-Jun signaling pathways was reported to modify the inflammatory status of periodontal by regulating macrophages polarization [41]. At in vivo level, in the colitis mice model, Akt kinase contributes to the polarization of macrophages and promotes M2 phenotype by deleting Akt2 [42]. Akt kinase may also dramatically affect atherogenesis. The double knockout (DKO) mice model which lacks both Ldlr and Akt2 has been shown to promote the impaired tolerance for glucose and, compared with Ldlr^−/−^ controls, more complex atherosclerotic plaques were formed [43]. These findings show the crucial role of Akt2 isoforms and alteration in the PI3K/Akt signaling pathway for studying the polarization and survival of macrophages, further altering the defense system of the host and disease process. Our study has confirmed the downregulation of Akt2 in *F. nucleatum*-infected macrophages, which may promote the disease progress such as periodontitis, colitis and atherosclerosis.

The family of microphthalmia transcription factors (MITF) diversely affect cellular processes, such as stress adaptation, metabolism, apoptosis, sensing of nutrients, proliferation, and formation of organelles [44]. It has been demonstrated that in periodontitis and rheumatic arthritis, the MITF family has an important role in osteoclast differentiation and function [45–47]. It has been also well reported as a master regulator of tumorigenesis in colon cancer [48]. MITF regulate lysosomal biogenesis and function, and lysosomal dysfunction in macrophages has been reported as a risk of atherosclerosis development [49]. Additionally, studies have shown that the MITF family play important role in the regulation of cardiovascular system homeostasis, thus is beneficial for the treatment of CVDs, including aortic aneurysm, atherosclerosis, cardiotoxicity, and postischemic [50]. These studies suggest that the disorder of MITF may be one of the pathogenic mechanisms leading to the progression of *F. nucleatum*-associated diseases.

We further analyzed the upstream regulatory non-coding RNAs of these DEGs using target prediction software. MicroRNAs are recently studied ncRNAs that suppress target genes’ expression by blocking the 3′ UTR of the genes [51]. We screened 5 aberrantly expressed microRNAs (*P <* 0.05 and |fold change| > 2). After qRT-PCR verification, the expression of miR-150-5p and miR-146a-3p were significantly up-regulated, highly consistent with the sequencing results. They have previously been shown to have a role in infection, inflammation, and immunity [52, 53].

To study the basic mechanism of functions of circRNA and lncRNA, we combined differentially expressed cricRNAs, lncRNAs, miRNAs, and mRNAs and constructed co-expression networks. The 2 core genes were screened to be further regulated by the ceRNA network: The expression of AKT2 may be controlled by hsa-miR-150-5p, of which the upstream non-coding RNA molecules include hsa_circ_0078617, hsa_circ_0069227, hsa_circ_0084089, lncRNA NUP210, lncRNA ABCB9, lncRNA DIXDC1, lncRNA ATXN1 and lncRNA XLOC_237387; MITF expression may be coordinated by hsa-miR-146a-3p, of which the upstream non-coding RNA molecules include hsa_circ_0001165, hsa_circ_0008460, hsa_circ_0001118, lncRNA XLOC_237387 and lncRNA ATXN1.

The upstream regulatory molecule of AKT2, miR-150-5p, is known as an immune-associated microRNA, highly expressed in monocytes/macrophages, and extensive involvement in inflammatory processes. Meanwhile, miR-150-5p was also the most significantly up-regulated microRNA in our sequencing results (Table 2). MiR-150 has been confirmed to directly target AKT2 and promote cell apoptosis before [54, 55]. In cancers, miR-150 is differentially downregulated which stimulates the PI3K-Akt pathway activation continuously that led to the activation of telomerase activity and makes the cancer cells immortal [56]. In inflamed gingiva tissues, miR-150 was con-firmed to be the most overexpressed miRNAs, and associated with chronic periodontitis lesions [57]. In THP-1 cells, miRNA-150 is packaged into macrovesicles (MVs) for secretion to HMEC-1 cells and circulating to mediate intracellular communication as signaling molecules, which effectively enhanced HMEC-1 cell migration and the risk of atherosclerosis [58]. MiR-150 was also reported to increase permeability in the epithelial cells of the intestine by changing the structure and function of tight junction proteins [59]. Serum miR-150-5p levels might serve as biomarkers in inflammatory bowel disease (IBD) [60]. Furthermore, miR-150 has been confirmed to modulate the growth of colon cancer and the progression of rheumatoid arthritis, which might to be new molecular biomarker with clinic relevance [61–64]. Among the circRNA/lncRNA-miRNA-mRNA triple network, we particularly focused on circRNAs/lncRNAs which can bind to miR-150-5p and regulate the AKT2 expression by acting as ceRNAs. The co-expression axes were discovered, namely hsa_circ_0078617/hsa_circ_0069227/hsa_circ_0084089/ has_miR-150-5p/AKT2 axis and lncRNA NUP210/lncRNA ABCB9/lncRNA DIXDC1/lncRNA ATXN1/lncRNA XLOC_237387/has_miR-150-5p/AKT2 axis. Interestingly, has_circ_0078617 has previously been reported to regulate MMP2 expression as ceRNA during virus infection, revealing its potential immunomodulatory role [65].

By exploring the regulatory molecules upstream of MITF, we set our sights on the hsa_circ_0001165/hsa_circ_0008460/hsa_circ_0001118/has_miR-146a-3p/MITF axis and lncRNA XLOC_237387/lncRNA ATXN1/has_miR-146a-3p/MITF axis. miR-146a is an important regulator in innate and adaptive immune responses, which is induced by pro-inflammatory factors. In turn, miR-146a can inhibit excessive inflammatory response of macrophages, thereby mediating bacterial immune tolerance. Macrophages contain high abundance of miR-146a [66, 67]. It has been reported that compared with M1-type macrophages, the expression of miR-146a-3p in M2-type macrophages is up-regulated [68]. Another study has shown that miR-146a-3p can exert M1 polarization of macrophages by targeting SIRT1 [69]. A meta-study has found that miR-146a is the most widely researched miRNA in periodontal diseases, and most studies reported higher expression levels of miR-146a in patients with periodontitis than in healthy controls [70]. miR-146a can also regulate lipid droplet formation by targeting ACSL1, which may be a potential biomarker and therapeutic target for atherosclerosis [71]. An animal study has shown that miR-146a-3p is increased in aortic wall tissues of atherosclerosis mice, and the inhibition of miR-146a-3p was correlated to lower plasma lipid level, reduced inflammatory factors in serum, attenuated aortic wall apoptosis, increased antioxidant stress capacity, and improved the stability of pathological plaque by targeting HDAC1 [72]. miR-146a-3p has also been reported to be associated with inflammatory bowel disease (IBD), which was significantly overexpressed in UC tissue as compared to adjacent normal tissue of patients with ulcerative colitis, as well as to normal mucosa from healthy controls [73]. miR-146a has also been found to be differentially expressed in rheumatoid arthritis (RA) involved in its pathogenesis [74]. In colorectal cancer, miR-146a was identified as a major negative regulator of colonic inflammation and associated tumorigenesis by modulating IL-17 responses [75]. The upstream non-coding RNA hsa_circ_0001165 may regulate TNF expression through hsa-miR-187-3p to induce EMT in prostate cancer cells [76]. As for hsa_circ_0008460, hsa_circ_0001118, lncRNA XLOC_237387 and lncRNA ATXN1, however, have not been previously reported. We hypothesized that their decreased expression enhanced the inhibitory effect of miR-146a-3p on MITF, thus accelerating the progression of *F. nucleatum*-associated diseases.

## Conclusions

Concluding, we studied specific lncRNAs and circRNAs that regulated responses of macrophages to *F. nucleatum* infection, functioning as ceRNAs. The two main miRNAs (miR-150-5p and miR-146a-3p) and their target mRNAs (AKT2 and MITF) were regarded as important moderators, which control immune responses related to *F. nucleatum* in this pathological context. Results of this study provide a base for prospective works about the mechanisms of circRNAs/lncRNAs governing *F. nucleatum* pathogenic role and might provide a promising therapeutic tool to prevent diseases associated with *F. nucleatum*.

## Methods

### Bacterial strain

*Fusobacterium nucleatum* ATCC 25586 was grown on blood agar plates (BD Bio-sciences, CA, USA) supplemented with a mixture of hemin 5 mg/ml, menadione 1 mg/ml, and sterile defibrinated sheep blood (Yikang Biotechnology Co., China) in anaerobic condition at 37 °C. The pure colonies of bacteria were then shifted to brain heart infusion broth (BD Biosciences, CA, USA) until they reached the stage of logarithmic growth. On the infection day, the suspension bacterial culture was centrifuged and washed with PBS. The O.D. was evaluated at 540 nm to quantify the bacterial cells’ number by using a microplate reader (Thermo Scientific, USA).

### Cell culture and infection

TIB-202 cell lines were provided by ATCC and grown in RPMI1640 medium (Gibco, USA). The Medium was enriched with 10% FBS (Hyclone, USA). The cells were incubated at 37 °C under 5% CO2. THP-1 monocytes were exposed to 100 nM PMA (phorbol 12-myristate 13 acetates, Sigma, USA) for 2 days of differentiation. The number of cells seeded in a 6-well plate was 1 × 10^6^ per milliliter. Macrophages were stimulated with *F. nucleatum* at a multiplicity of infection (MOI) of 100 for 24 h, and then they were harvested for RNA-seq and miRNA-seq.

### Cell viability assay

MTT cell proliferation kit (Beyotime Biotechnology, China) was used to measure cellular viability and proliferation according to the instructions of the manufacturer. Briefly, cells at the density of 1 × 10^5^ each well were seeded in 96-well plates. It was followed by infection of the cells with bacteria for indicated durations of time (24 h and 48 h). A microplate reader (Thermo Scientific, USA) was used to measure the absorbance at 570 nm.

### Total RNA isolation

Total RNA was extracted, and the RNA purity and concentration was measured by using a Nanodrop. The RNA integrity was examined with an Agilent 2100 bioanalyzer. Total RNA of each sample will be split in two for RNA-seq and miRNA-seq, respectively.

### Library construction and sequencing of the whole transcriptome

Strand-specific RNA-seq libraries were constructed as follows [77]. After getting rid of the rRNA, the total RNA was fragmented into short segments of 250-300 bp. The first strand of cDNA was synthesized by reverse transcribing the segmented RNA and random oligonucleotides were used as the primer; Then, RNase H was used to degrade the strands of RNA, and the second strand of cDNA was synthesized by DNA polymerase I system using dNTPs as the raw materials in. The purified double-stranded cDNA was repaired at the end and attached to the sequencing connector with an added tail. Products were purified (AMPure XP system). The second strand of cDNA containing U was degraded by the USER enzyme and PCR amplification was performed to construct the RNA-seq libraries (including lncRNA, mRNA, circRNA, etc). The quality of the library was assessed on the Agilent Bioanalyzer 2100 system. Finally, the library was sequenced on Illumina PE150 platform, and 150-bp paired-end reads were generated after cluster generation. The Raw data obtained by RNA-seq were used for the subsequent bioinformatic analysis.

### Library construction and sequencing of small RNAs

MiRNA-seq libraries for miRNA-seq were prepared with Small RNA Sample Pre-Kit. Using total RNA as the template, small RNA-specific 3′-adapter and 5′-adapter linker sequences were ligated to RNA samples. Synthesis of cDNA was performed by reverse transcription. Subsequently, the DNA fragments were purified by PCR amplification and PAGE gel electrophoresis to construct the miRNA-seq libraries. Library quality was evaluated on the Agilent Bioanalyzer 2100 system. MiRNA-seq was performed on the Illumina Novaseq™ 6000 platform, and the Raw data obtained were used for the subsequent bioinformatics analysis.

### Raw data information

The RNA-seq and miRNA-seq data have been submitted to the Genome Expression Omnibus (GEO) database, with the accession number of GSE190606 (https://www.ncbi.nlm.nih.gov/geo/query/acc.cgi?acc=GSE190606).

### RNA-seq and miRNA-seq data analysis

The Raw Data files obtained by sequencing were analyzed by Base Calling and transformed into Raw Reads and stored as FASTQ file. Quality control of the sequencing raw data was performed, mainly including raw data filtering, sequencing error rate check and GC content distribution check. After that, clean reads with high quality for subsequent analysis were obtained.

Hisat2 (http://ccb.jhu.edu/software/hisat2) software was applied for mapping of RNA-seq data. Mapped reads were spliced by using StringTie [78], and then merged by the cuffmerge software. The obtained transcripts were filtered as follows: (a) exon number ≥ 2, filtering single exon transcripts with low expression level and low confidence in the splicing result of the transcriptome; (b) the length of the transcript ≥200 bp; (c) The transcripts have no coding potential. Following that, compare with the known database by using Cuffcompare software, filter out the known transcripts and predict the coding potential of the new transcripts to obtain the novel lncRNAs and novel mRNAs. Referring to the naming guidelines for long non-coding RNA of HGNC (The HUGO Gene Nomenclature Committee), the candidate Novel_lncRNAs was finally screened and named according to its position relationship with coding genes [79]. The expressed levels of lncRNAs and mRNAs were estimated as FPKM (Fragments Per Kilobase of transcript sequence per Millions base pairs sequenced). CircRNA was identified by find_circ and CIRI softwares [80, 81]. The expression levels of all circRNAs were quantified and normalized by TPM [82].

The small RNAs after length screening were mapped to the reference sequence with Bowtie software. For known miRNAs, the mapped reads on the reference sequence were compared with the specified range sequence in miRbase. In addition, we integrated miREvo and Mirdeep2 software to predict novel miRNAs [83, 84]. The expressed levels of all miRNAs were also normalized as TPM.

The correlation analysis of gene expression among samples was conducted. The differential expressed mRNAs and lncRNAs were screened by edgeR software, while the aberrantly expressed circRNAs and miRNAs were analyzed by DESeq2 software [85]. The default screening condition for differential expression was *P* < 0.05 and |fold change (FC) | > 2.

### Functional enrichment analysis

To predict the potential functions and analyze the pathways involved in the differentially expressed mRNAs (DEGs), clusterProfiler software (http://www.bioconductor.org/packages/release/bioc/html/clusterProfiler.html) was applied with GO (http://www.geneontology.org) and KEGG (http://www.genome.jp/kegg) databases selected [86–89]. The *P* value < 0.05 and |fold change| > 2 was defined as statistically significant. The Kobas online website (http://kobas.cbi.pku.edu.cn/kobas3/?t=1) was used to conduct disease KEGG analysis.

### Protein-protein interaction (PPI) network analysis

The Search Tool STRING version 11.0 (https://string-db.org/) for the Retrieval of Interacting Genes/Proteins was used to retrieve the PPI network between DEGs identified previously [90]. Only STRING-derived interactors with high confidence (> 0.700) were imported into the software i.e., Cytoscape ver-3.6.1 to construct the PPI network [91].

### The co-expression network of differentially expressed miRNAs, lncRNAs, circRNAs and mRNAs

The existing miRNA target prediction methods were employed to evaluate the circRNA/lncRNA-miRNA-mRNA interactions. The Ensembl database was employed to obtain the UTR sequences. TargetScan and Miranda software were employed to predict putative targets based on the ceRNA hypothesis. Based on pre-existing data on co-expression, Cytoscape Ver-3.2.1 was employed to depict interactions among circRNA, LncRNA, miRNA, and mRNA.

### Real-time quantitative PCR (qRT-PCR) analysis

Total RNAs of each sample was extracted by using the miRNeasy Mini Kit (Qiagen, USA). Mir-X miRNA First-Strand Synthesis Kit (Takara Bio, Japan) was used for microRNA reverse-transcribed, and PrimeScript RT Reagent Kit (Takara Bio, Japan) was used for cDNA synthesis. qRT-PCR was performed in triplicates with TB Green® Kit (Takara Bio, Japan) on QPCR System LightCycler® 480 II (Roche Diagnostics, Germany). Relative miRNAs expression was normalized to U6. Relative expression of mRNAs, lncRNAs and circRNAs was normalized to GAPDH. All Primers (shown in Supplementary Table 1) were designed and synthesized by Sangon Biotech. Data were collected and quantitatively analyzed with the 2^−ΔΔCT^ method.

## Supplementary Information

**Additional file 1: Supplementary Table 1.** All Primers used in qRT-PCR analysis.

**Additional file 2: Supplementary Table 2.** Differentially expressed mRNAs screened by RNA-Seq.

**Additional file 3: Supplementary Table 3.** KEGG Disease analysis of the differentially expressed mRNAs.

**Additional file 4: Supplementary Table 4.** Protein and protein interaction (PPI) analysis of the differentially expressed mRNAs.

**Additional file 5: Supplementary Table 5.** All identified miRNAs in miRNA-Seq.

**Additional file 6: Supplementary Table 6.** Differentially expressed miRNAs screened by miRNA-Seq.

**Additional file 7: Supplementary Table 7.** DE-miRNAs-target DEGs pairs.

**Additional file 8: Supplementary Table 8.** All identified lncRNAs in RNA-Seq.

**Additional file 9: Supplementary Table 9.** All identified circRNAs in RNA-Seq.

**Additional file 10: Supplementary Table 10.** Differentially expressed lncRNAs screened by RNA-Seq.

**Additional file 11: Supplementary Table 11.** Differentially expressed circRNAs screened by RNA-Seq.

**Additional file 12: Supplementary txt1.** miRNA hairpin.

**Additional file 13: Supplementary txt2.** miRNA mature.

## Data Availability

All the data supporting our findings are contained within the manuscript. All raw transcriptome data reported in this article have been deposited in the Genome Expression Omnibus (GEO) database under accession number GSE190606.

## Abbreviations

IBD: Inflammatory bowel disease
*F. nucleatum, Fn*: *Fusobacterium nucleatum*
PD: Periodontitis
APO: Adverse pregnancy outcome
RA: Rheumatoid arthritis
IDO: Indoleamine 2,3-dioxygenase
CRC: Colorectal cancer
UC: Ulcerative colitis
ncRNA: Non-coding RNA
miRNA: microRNA
circRNA: Circular RNA
lncRNA: Long non-coding RNA
mRNA: Messenger RNA
MRE: miRNA response element
ceRNA: Competing endogenous RNA
THP-1: The human monocytic cell line
dTHP-1: THP-1-derived macrophages
MOI: Multiplicity of infection
GO: Gene Ontology
KEGG: Kyoto Encyclopedia of Genes and Genomes
PPI: Protein-protein interaction
DE-miRNA: Differentially expressed miRNA
DE-mRNA: Differentially expressed mRNA
DE-lncRNA: Differentially expressed lncRNA
DE-circRNA: Differentially expressed circRNA
FC: Foldchange
ORF: Open reading frame
Akt2: Serine/threonine protein kinase 2
MITF: Microphthalmia transcription factor
