# Supplementary material for: Identification and characterization of non-coding RNA networks in infected macrophages revealing the pathogenesis of F. nucleatum-associated diseases
Source: BMC Genomics. 2022 Dec 13;23:826. doi: 10.1186/s12864-022-09052-z (PMC9749367; doi:10.1186/s12864-022-09052-z)
Supplement: Supplementary file 12 — Additional file 12: Supplementary txt1. miRNA hairpin. [file 12864_2022_9052_MOESM12_ESM.docx]

>hsa-let-7a-1

UGGGAUGAGGUAGUAGGUUGUAUAGUUUUAGGGUCACACCCACCACUGGGAGAUAACUAUACAAUCUACUGUCUUUCCUA

>hsa-let-7a-2

AGGUUGAGGUAGUAGGUUGUAUAGUUUAGAAUUACAUCAAGGGAGAUAACUGUACAGCCUCCUAGCUUUCCU

>hsa-let-7a-3

GGGUGAGGUAGUAGGUUGUAUAGUUUGGGGCUCUGCCCUGCUAUGGGAUAACUAUACAAUCUACUGUCUUUCCU

>hsa-let-7b

CGGGGUGAGGUAGUAGGUUGUGUGGUUUCAGGGCAGUGAUGUUGCCCCUCGGAAGAUAACUAUACAACCUACUGCCUUCCCUG

>hsa-let-7c

GCAUCCGGGUUGAGGUAGUAGGUUGUAUGGUUUAGAGUUACACCCUGGGAGUUAACUGUACAACCUUCUAGCUUUCCUUGGAGC

>hsa-let-7d

CCUAGGAAGAGGUAGUAGGUUGCAUAGUUUUAGGGCAGGGAUUUUGCCCACAAGGAGGUAACUAUACGACCUGCUGCCUUUCUUAGG

>hsa-let-7e

CCCGGGCUGAGGUAGGAGGUUGUAUAGUUGAGGAGGACACCCAAGGAGAUCACUAUACGGCCUCCUAGCUUUCCCCAGG

>hsa-let-7f-1

UCAGAGUGAGGUAGUAGAUUGUAUAGUUGUGGGGUAGUGAUUUUACCCUGUUCAGGAGAUAACUAUACAAUCUAUUGCCUUCCCUGA

>hsa-let-7f-2

UGUGGGAUGAGGUAGUAGAUUGUAUAGUUUUAGGGUCAUACCCCAUCUUGGAGAUAACUAUACAGUCUACUGUCUUUCCCACG

>hsa-let-7g

AGGCUGAGGUAGUAGUUUGUACAGUUUGAGGGUCUAUGAUACCACCCGGUACAGGAGAUAACUGUACAGGCCACUGCCUUGCCA

>hsa-let-7i

CUGGCUGAGGUAGUAGUUUGUGCUGUUGGUCGGGUUGUGACAUUGCCCGCUGUGGAGAUAACUGCGCAAGCUACUGCCUUGCUA

>hsa-mir-1-1

UGGGAAACAUACUUCUUUAUAUGCCCAUAUGGACCUGCUAAGCUAUGGAAUGUAAAGAAGUAUGUAUCUCA

>hsa-mir-1-2

ACCUACUCAGAGUACAUACUUCUUUAUGUACCCAUAUGAACAUACAAUGCUAUGGAAUGUAAAGAAGUAUGUAUUUUUGGUAGGC

>hsa-mir-100

CCUGUUGCCACAAACCCGUAGAUCCGAACUUGUGGUAUUAGUCCGCACAAGCUUGUAUCUAUAGGUAUGUGUCUGUUAGG

>hsa-mir-101-1

UGCCCUGGCUCAGUUAUCACAGUGCUGAUGCUGUCUAUUCUAAAGGUACAGUACUGUGAUAACUGAAGGAUGGCA

>hsa-mir-101-2

ACUGUCCUUUUUCGGUUAUCAUGGUACCGAUGCUGUAUAUCUGAAAGGUACAGUACUGUGAUAACUGAAGAAUGGUGGU

>hsa-mir-10392

GCGCUUCGACGGGCUGGGCUGUGCGCCUGCGCAGUGUGGGUCGCUCCCGAUUCCCUGCCCCGGCCGGCCCCGCCUCGGCUCCGCACC

>hsa-mir-10393

AGAAUUCUCUUAUCCAACAUCAACAUCUUGGUCAGAUUUGAACUCUUCAA

>hsa-mir-10394

UCUGCAGGUCCUGGUGAACGCCAUCAUCAACAGUGGUCCCCGGGAGGACUCCACACGCAUUGGGCGCGCCGGGACUGUGAGAC

>hsa-mir-10395

GUGAUGGAGAGCAAUACCCGGGGAUGACUGUGACCACAUUGGGAUGUAUUCGUACUGUCUGAUG

>hsa-mir-10396a

GGCGGGGCUCGGAGCCGGGCUUCGGCCGGGCCCCGGGCCCUCGACCGGG

>hsa-mir-10396b

CGGCGGGGCUCGGAGCCGGGCUUCGGCCGGGCCCCGGGCCCUCGACCGGAC

>hsa-mir-10397

UCCUUGACCUGAUGCUGUAGGGCUUGGCAACUCUUGUGAAAUUUCAUAGAUCUCGUCGCUUACUGGGA

>hsa-mir-10398

UGGCUCCCUUCUCUCCGUCUGCCUCCUGGCCGCGGGGCCCGGAGAGCUGGGAGCCAG

>hsa-mir-10399

AAUUACAGAUUGUCUCAGAGAAAACAAAUGAGUUACUCUCUCGGACAAGCUGUAGGUC

>hsa-mir-103a-1

UACUGCCCUCGGCUUCUUUACAGUGCUGCCUUGUUGCAUAUGGAUCAAGCAGCAUUGUACAGGGCUAUGAAGGCAUUG

>hsa-mir-103a-2

UUGUGCUUUCAGCUUCUUUACAGUGCUGCCUUGUAGCAUUCAGGUCAAGCAGCAUUGUACAGGGCUAUGAAAGAACCA

>hsa-mir-103b-1

UCAUAGCCCUGUACAAUGCUGCUUGAUCCAUAUGCAACAAGGCAGCACUGUAAAGAAGCCGA

>hsa-mir-103b-2

UCAUAGCCCUGUACAAUGCUGCUUGACCUGAAUGCUACAAGGCAGCACUGUAAAGAAGCUGA

>hsa-mir-10401

CGUGUGGGAAGGCGUGGGGUGCGGACCCCGGCCCGACCUCGCCGUCCCGCCCGCCG

>hsa-mir-10522

AGAAGAAUUGGCCUACUCAGGUGUCAGCAAAGCCUUCCUCUCUGAAUAGGUCAAUUCCCCUCA

>hsa-mir-10527

AAAGCAAAUGUUGGGUGAACGGCUGUUUCCUCUUAUUCAAGCCAUGCACCUUACUCUUGCUGGUA

>hsa-mir-106a

CCUUGGCCAUGUAAAAGUGCUUACAGUGCAGGUAGCUUUUUGAGAUCUACUGCAAUGUAAGCACUUCUUACAUUACCAUGG

>hsa-mir-106b

CCUGCCGGGGCUAAAGUGCUGACAGUGCAGAUAGUGGUCCUCUCCGUGCUACCGCACUGUGGGUACUUGCUGCUCCAGCAGG

>hsa-mir-107

CUCUCUGCUUUCAGCUUCUUUACAGUGUUGCCUUGUGGCAUGGAGUUCAAGCAGCAUUGUACAGGGCUAUCAAAGCACAGA

>hsa-mir-10a

GAUCUGUCUGUCUUCUGUAUAUACCCUGUAGAUCCGAAUUUGUGUAAGGAAUUUUGUGGUCACAAAUUCGUAUCUAGGGGAAUAUGUAGUUGACAUAAACACUCCGCUCU

>hsa-mir-10b

CCAGAGGUUGUAACGUUGUCUAUAUAUACCCUGUAGAACCGAAUUUGUGUGGUAUCCGUAUAGUCACAGAUUCGAUUCUAGGGGAAUAUAUGGUCGAUGCAAAAACUUCA

>hsa-mir-11399

UCAGUGCUCUUUCAGGUCUGGGGCUGAAACCUGUCUUGUUUCCCCAUCAGAGGCAGUCCCAGGCCCGAAAGAGGAGAGAU

>hsa-mir-11400

CAAGGGCGACUCGGCUGUGUAUCUCUGUGUCAGAAGCUUAGCCACAGUGUGGCACAGUCGUGUCCUUCCUGCUCACA

>hsa-mir-11401

CUCGCGGCUGUCACGUCUGCGGCUGUCACGUCUGCUAGGGGGUUGGGGGGUCCAGGAAGGGCAGCGAGU

>hsa-mir-1179

GGCUGGAAAGGAAGAAGCAUUCUUUCAUUGGUUGGUGUGUAUUGCCUUGUCAACCAAUAAGAGGAUGCCAUUUAUCCUUUUCUGACUAGCU

>hsa-mir-1180

GCUGCUGGACCCACCCGGCCGGGAAUAGUGCUCCUGGUUGUUUCCGGCUCGCGUGGGUGUGUCGGCGGC

>hsa-mir-1181

UCCACUGCUGCCGCCGUCGCCGCCACCCGAGCCGGAGCGGGCUGGGCCGCCAAGGCAAGAUGGUGGACUACAGCGUGUGGG

>hsa-mir-1184-1

CUUGCAGAACGAGGUGAAGGAGGUGGUUCUGCUCAGCAGUCAACAGUGGCCACAUCUCCACCUGCAGCGACUUGAUGGCUUCCGUGUCCUUUUCGUGGG

>hsa-mir-1184-2

CUUGCAGAACGAGGUGAAGGAGGUGGUUCUGCUCAGCAGUCAACAGUGGCCACAUCUCCACCUGCAGCGACUUGAUGGCUUCCGUGUCCUUUUCGUGGG

>hsa-mir-1184-3

CUUGCAGAACGAGGUGAAGGAGGUGGUUCUGCUCAGCAGUCAACAGUGGCCACAUCUCCACCUGCAGCGACUUGAUGGCUUCCGUGUCCUUUUCGUGGG

>hsa-mir-12114

GUGCUGUCACUGACCAGGUGGAGGUGUGAGGUCCUGGGGCAUCAUGGCAGGACCUUGGACAUCCACUUUCUCAGGUCUGCAGC

>hsa-mir-12117

GUCUCAGUGAAGUGGAGCACAUCAGUGAAAGGGUGAACUUAACCUUUCACUGGUGUGCUCCAUCUCACUCAGAC

>hsa-mir-12124

GGCCUCGCUUUCUUUCCCCAUGCUCUGCACUCUGCCACGCACCCCUUCCUCGGCCAGGCGGACAGAGGAAAUGCAGAUGCUGGAGGCAGGAGCACUGGACU

>hsa-mir-12133

GAAGUGUACUUUUUAAUGGUGCCAAACAGCAGUUGAUCUAUAAUAACUGCUGCUUGGCACCAUUAAAAAGUACAUAUC

>hsa-mir-12136

GAAAAAGUCAUGGAGGCCAUGGGGUUGGCUUGAAACCAGCUUUGGGGGGUUCGAUUCCUUCCUUUUUUGUC

>hsa-mir-122

CCUUAGCAGAGCUGUGGAGUGUGACAAUGGUGUUUGUGUCUAAACUAUCAAACGCCAUUAUCACACUAAAUAGCUACUGCUAGGC

>hsa-mir-1225

GUGGGUACGGCCCAGUGGGGGGGAGAGGGACACGCCCUGGGCUCUGCCCAGGGUGCAGCCGGACUGACUGAGCCCCUGUGCCGCCCCCAG

>hsa-mir-1226

GUGAGGGCAUGCAGGCCUGGAUGGGGCAGCUGGGAUGGUCCAAAAGGGUGGCCUCACCAGCCCUGUGUUCCCUAG

>hsa-mir-1227

GUGGGGCCAGGCGGUGGUGGGCACUGCUGGGGUGGGCACAGCAGCCAUGCAGAGCGGGCAUUUGACCCCGUGCCACCCUUUUCCCCAG

>hsa-mir-1228

GUGGGCGGGGGCAGGUGUGUGGUGGGUGGUGGCCUGCGGUGAGCAGGGCCCUCACACCUGCCUCGCCCCCCAG

>hsa-mir-1229

GUGGGUAGGGUUUGGGGGAGAGCGUGGGCUGGGGUUCAGGGACACCCUCUCACCACUGCCCUCCCACAG

>hsa-mir-122b

CAGUAGCUAUUUAGUGUGAUAAUGGCGUUUGAUAGUUUAGACACAAACACCAUUGUCACACUCCACAGCUCUG

>hsa-mir-1233-1

GUGAGUGGGAGGCCAGGGCACGGCAGGGGGAGCUGCAGGGCUAUGGGAGGGGCCCCAGCGUCUGAGCCCUGUCCUCCCGCAG

>hsa-mir-1233-2

GUGAGUGGGAGGCCAGGGCACGGCAGGGGGAGCUGCAGGGCUAUGGGAGGGGCCCCAGCGUCUGAGCCCUGUCCUCCCGCAG

>hsa-mir-1234

GUGAGUGUGGGGUGGCUGGGGCGGGGGGGGCCCGGGGACGGCUUGGGCCUGCCUAGUCGGCCUGACCACCCACCCCACAG

>hsa-mir-1236

GUGAGUGACAGGGGAAAUGGGGAUGGACUGGAAGUGGGCAGCAUGGAGCUGACCUUCAUCAUGGCUUGGCCAACAUAAUGCCUCUUCCCCUUGUCUCUCCAG

>hsa-mir-1237

GUGGGAGGGCCCAGGCGCGGGCAGGGGUGGGGGUGGCAGAGCGCUGUCCCGGGGGCGGGGCCGAAGCGCGGCGACCGUAACUCCUUCUGCUCCGUCCCCCAG

>hsa-mir-1238

GUGAGUGGGAGCCCCAGUGUGUGGUUGGGGCCAUGGCGGGUGGGCAGCCCAGCCUCUGAGCCUUCCUCGUCUGUCUGCCCCAG

>hsa-mir-124-1

AGGCCUCUCUCUCCGUGUUCACAGCGGACCUUGAUUUAAAUGUCCAUACAAUUAAGGCACGCGGUGAAUGCCAAGAAUGGGGCUG

>hsa-mir-124-2

AUCAAGAUUAGAGGCUCUGCUCUCCGUGUUCACAGCGGACCUUGAUUUAAUGUCAUACAAUUAAGGCACGCGGUGAAUGCCAAGAGCGGAGCCUACGGCUGCACUUGAA

>hsa-mir-124-3

UGAGGGCCCCUCUGCGUGUUCACAGCGGACCUUGAUUUAAUGUCUAUACAAUUAAGGCACGCGGUGAAUGCCAAGAGAGGCGCCUCC

>hsa-mir-1243

CUAAAACUGGAUCAAUUAUAGGAGUGAAAUAAAGGUCCAUCUCCUGCCUAUUUAUUACUUUGCUUUGGUAAUAAAUCUAUUUUUAAAAGAACC

>hsa-mir-1244-1

AUCUUAUUCCGAGCAUUCCAGUAACUUUUUUGUGUAUGUACUUAGCUGUACUAUAAGUAGUUGGUUUGUAUGAGAUGGUUAAAAA

>hsa-mir-1244-2

AUCUUAUUCCGAGCAUUCCAGUAACUUUUUUGUGUAUGUACUUAGCUGUACUAUAAGUAGUUGGUUUGUAUGAGAUGGUUAAAAA

>hsa-mir-1244-3

AUCUUAUUCCGAGCAUUCCAGUAACUUUUUUGUGUAUGUACUUAGCUGUACUAUAAGUAGUUGGUUUGUAUGAGAUGGUUAAAAA

>hsa-mir-1244-4

AUCUUAUUCCGAGCAUUCCAGUAACUUUUUUGUGUAUGUACUUAGCUGUACUAUAAGUAGUUGGUUUGUAUGAGAUGGUUAAAAA

>hsa-mir-1246

UGUAUCCUUGAAUGGAUUUUUGGAGCAGGAGUGGACACCUGACCCAAAGGAAAUCAAUCCAUAGGCUAGCAAU

>hsa-mir-1247

CCGCUUGCCUCGCCCAGCGCAGCCCCGGCCGCUGGGCGCACCCGUCCCGUUCGUCCCCGGACGUUGCUCUCUACCCCGGGAACGUCGAGACUGGAGCGCCCGAACUGAGCCACCUUCGCGGACCCCGAGAGCGGCG

>hsa-mir-1248

UUUACCUUCUUGUAUAAGCACUGUGCUAAAAUUGCAGACACUAGGACCAUGUCUUGGUUUUUGCAAUAAUGCUAGCAGAGUACACACAAGAAGAAAAGUAACAGCA

>hsa-mir-1249

GGGAGGAGGGAGGAGAUGGGCCAAGUUCCCUCUGGCUGGAACGCCCUUCCCCCCCUUCUUCACCUG

>hsa-mir-1250

CUGUCCCGCUGGCCUGGCAGGUGACGGUGCUGGAUGUGGCCUUUUUGCCUUUUCUAAAGGCCACAUUUUCCAGCCCAUUCAACCUUCCAGAGCCCUCUGAAGUGGCCACAGGC

>hsa-mir-1255a

AUUGGAAAUCCUUUGAGUUGCUUCUCAAGGAUGAGCAAAGAAAGUAGAUUUUUUAGAUUCUAAAGAAACUAUCUUCUUUGCUCAUCCUUGAGAAGCAACUCCUUAUCCAUUAA

>hsa-mir-1255b-1

UACGGAUGAGCAAAGAAAGUGGUUUCUUAAAAUGGAAUCUACUCUUUGUGAAGAUGCUGUGAA

>hsa-mir-1255b-2

UCUUACGGAUGAGCAAAGAAAGUGGUUUGCGCCUCAAGAAACCACUUUCUUUGCUCAUCCAUAAGGA

>hsa-mir-1256

AGUCAGCCUGUUGAAGCUUUGAAGCUUUGAUGCCAGGCAUUGACUUCUCACUAGCUGUGAAAGUCCUAGCUAAAGAGAAGUCAAUGCAUGACAUCUUGUUUCAAUAGAUGGCUGUUUCA

>hsa-mir-125a

UGCCAGUCUCUAGGUCCCUGAGACCCUUUAACCUGUGAGGACAUCCAGGGUCACAGGUGAGGUUCUUGGGAGCCUGGCGUCUGGCC

>hsa-mir-125b-1

UGCGCUCCUCUCAGUCCCUGAGACCCUAACUUGUGAUGUUUACCGUUUAAAUCCACGGGUUAGGCUCUUGGGAGCUGCGAGUCGUGCU

>hsa-mir-125b-2

ACCAGACUUUUCCUAGUCCCUGAGACCCUAACUUGUGAGGUAUUUUAGUAACAUCACAAGUCAGGCUCUUGGGACCUAGGCGGAGGGGA

>hsa-mir-126

CGCUGGCGACGGGACAUUAUUACUUUUGGUACGCGCUGUGACACUUCAAACUCGUACCGUGAGUAAUAAUGCGCCGUCCACGGCA

>hsa-mir-1260a

ACCUUUCCAGCUCAUCCCACCUCUGCCACCAAAACACUCAUCGCGGGGUCAGAGGGAGUGCCAAAAAAGGUAA

>hsa-mir-1260b

UCUCCGUUUAUCCCACCACUGCCACCAUUAUUGCUACUGUUCAGCAGGUGCUGCUGGUGGUGAUGGUGAUAGUCUGGUGGGGGCGGUGG

>hsa-mir-1262

AUCUACAAUGGUGAUGGGUGAAUUUGUAGAAGGAUGAAAGUCAAAGAAUCCUUCUGGGAACUAAUUUUUGGCCUUCAACAAGAAUUGUGAUAU

>hsa-mir-1263

CUACCCCAAAAUAUGGUACCCUGGCAUACUGAGUAUUUUAAUACUGGCAUACUCAGUAUGCCAUGUUGCCAUAUUUUGGGGUAGCA

>hsa-mir-1264

AGGUCCUCAAUAAGUAUUUGUUGAAAGAAUAAAUAAACCAACAAGUCUUAUUUGAGCACCUGUUAUGUG

>hsa-mir-1266

ACAGGUAGUGUCCCUCAGGGCUGUAGAACAGGGCUGGGAUUACUAAAGCCCUGUUCUAUGCCCUGAGGGACACUGAGCAUGUCA

>hsa-mir-1268a

UAGCCGGGCGUGGUGGUGGGGGCCUGUGGUCCCAGCUACUUUGGAGGCUGAG

>hsa-mir-1269a

UGGAUUGCCUAGACCAGGGAAGCCAGUUGGCAUGGCUCAGUCCAAGUCUGACCACCUGAGGAAUGCCUGGACUGAGCCGUGCUACUGGCUUCCCUGGUCUCCAGC

>hsa-mir-1269b

UGAGGUUUCUGGACUGAGCCAUGCUACUGGCUUCUCUGGUUCUCCAGCUUACAGAUGGCUUAUCAUGGGACCUCU

>hsa-mir-127

UGUGAUCACUGUCUCCAGCCUGCUGAAGCUCAGAGGGCUCUGAUUCAGAAAGAUCAUCGGAUCCGUCUGAGCUUGGCUGGUCGGAAGUCUCAUCAUC

>hsa-mir-1270

CACAGAGUUAUACUGGAGAUAUGGAAGAGCUGUGUUGGGUAUAAGUAACAGGCUUUUCUUUAUCUUCUAUGUGGCUCUUUGCA

>hsa-mir-1271

CACCCAGAUCAGUGCUUGGCACCUAGCAAGCACUCAGUAAAUAUUUGUUGAGUGCCUGCUAUGUGCCAGGCAUUGUGCUGAGGGCU

>hsa-mir-1273c

UGCAGCCUGGGCGACAAAACGAGACCCUGUCUUUUUUUUUUUCUGAGACAGAGUCUCGUUCUGUUGCCCAAGCUGGA

>hsa-mir-1273h

UACUUGGGUGACUAAGGCAGGAUUGCUUGAGCCUGGGAGGUCAAGGCUGCAGUGUCGUGGUCACAGCUUGCUGCAGACUCGACCUCCCAGGCUUAAGCAAUCCUCCUGCUCGAGUG

>hsa-mir-1275

CCUCUGUGAGAAAGGGUGUGGGGGAGAGGCUGUCUUGUGUCUGUAAGUAUGCCAAACUUAUUUUCCCCAAGGCAGAGGGA

>hsa-mir-1276

CCCCAGCUAGGUAAAGAGCCCUGUGGAGACACCUGGAUUCAGAGAACAUGUCUCCACUGAGCACUUGGGCCUUGAUGGCGGCU

>hsa-mir-1277

ACCUCCCAAAUAUAUAUAUAUAUGUACGUAUGUGUAUAUAAAUGUAUACGUAGAUAUAUAUGUAUUUUUGGUGGGUUU

>hsa-mir-1278

AUUUGCUCAUAGAUGAUAUGCAUAGUACUCCCAGAACUCAUUAAGUUGGUAGUACUGUGCAUAUCAUCUAUGAGCGAAUAG

>hsa-mir-128-1

UGAGCUGUUGGAUUCGGGGCCGUAGCACUGUCUGAGAGGUUUACAUUUCUCACAGUGAACCGGUCUCUUUUUCAGCUGCUUC

>hsa-mir-128-2

UGUGCAGUGGGAAGGGGGGCCGAUACACUGUACGAGAGUGAGUAGCAGGUCUCACAGUGAACCGGUCUCUUUCCCUACUGUGUC

>hsa-mir-1283-1

CUCAAGCUAUGAGUCUACAAAGGAAAGCGCUUUCUGUUGUCAGAAAGAAGAGAAAGCGCUUCCCUUUUGAGGGUUACGGUUUGAGAA

>hsa-mir-1283-2

CUCAAGCUGUGAGUCUACAAAGGAAAGCGCUUUCUGUUGUCUGAAAGAAAAGAAAUCGCUUCCCUUUGGAGUGUUACGGUUUGAGAA

>hsa-mir-1284

AUUUUGAUAUAUAAGCCAGUUUAAUGUUUUCUAUACAGACCCUGGCUUUUCUUAAAUUUUAUAUAUUGGAAAGCCCAUGUUUGUAUUGGAAACUGCUGGUUUCUUUCAUACUGAAAAUCU

>hsa-mir-1285-1

UGUAGAGAUAGGAUCUCACUUUGUUGCCCAGGCUGGUCUCAAACUCCUGGUCUGGGCAACAAAGUGAGACCUUAUCUCUACAAG

>hsa-mir-1285-2

UUUGGGAGGCCGAGGCUGGUGCAUCACUUGAGCCCAGCAAUUUGAGACCAAUCUGGGCAACAAAGUGAGACCUCCGUCUCUACAAAGA

>hsa-mir-1286

UGUCCUCUGGGGACUCAGCUUGCUCUGGCUGCUGGAUUGAAUUAGCUGCAGGACCAAGAUGAGCCCUUGGUGGAGACA

>hsa-mir-1287

GUUGUGCUGUCCAGGUGCUGGAUCAGUGGUUCGAGUCUGAGCCUUUAAAAGCCACUCUAGCCACAGAUGCAGUGAUUGGAGCCAUGACAA

>hsa-mir-1288

GAGGGUGUUGAUCAGCAGAUCAGGACUGUAACUCACCAUAGUGGUGGACUGCCCUGAUCUGGAGACCACUGCCUU

>hsa-mir-1289-1

UUCUCAAUUUUUAGUAGGAAUUAAAAACAAAACUGGUAAAUGCAGACUCUUGGUUUCCACCCCCAGAGAAUCCCUAAACCGGGGGUGGAGUCCAGGAAUCUGCAUUUUAGAAAGUACCCAGGGUGAUUCUGAUAAUUGGGAACA

>hsa-mir-1289-2

CCACGGUCCUAGUUAAAAAGGCACAUUCCUAGACCCUGCCUCAGAACUACUGAACAGAGUCACUGGGUGUGGAGUCCAGGAAUCUGCAUUUUUACCCCUAUCGCCCCCGCC

>hsa-mir-129-1

GGAUCUUUUUGCGGUCUGGGCUUGCUGUUCCUCUCAACAGUAGUCAGGAAGCCCUUACCCCAAAAAGUAUCU

>hsa-mir-129-2

UGCCCUUCGCGAAUCUUUUUGCGGUCUGGGCUUGCUGUACAUAACUCAAUAGCCGGAAGCCCUUACCCCAAAAAGCAUUUGCGGAGGGCG

>hsa-mir-1291

GGUAGAAUUCCAGUGGCCCUGACUGAAGACCAGCAGUUGUACUGUGGCUGUUGGUUUCAAGCAGAGGCCUAAAGGACUGUCUUCCUG

>hsa-mir-1292

CCUGGGAACGGGUUCCGGCAGACGCUGAGGUUGCGUUGACGCUCGCGCCCCGGCUCCCGUUCCAGG

>hsa-mir-1293

AGGUUGUUCUGGGUGGUCUGGAGAUUUGUGCAGCUUGUACCUGCACAAAUCUCCGGACCACUUAGUCUUUA

>hsa-mir-1294

CACCUAAUGUGUGCCAAGAUCUGUUCAUUUAUGAUCUCACCGAGUCCUGUGAGGUUGGCAUUGUUGUCUGGCAUUGUCUGAUAUACAACAGUGCCAACCUCACAGGACUCAGUGAGGUGAAACUGAGGAUUAGGAAGGUGUA

>hsa-mir-1295a

AGGACAUUUUGCCCAGAUCCGUGGCCUAUUCAGAAAUGUGGCCUGUGAUUAGGCCGCAGAUCUGGGUGAAAUGUCCUCC

>hsa-mir-1295b

CACCCAGAUCUGCGGCCUAAUCACAGGCCACAUUUCUGAAUAGGCCACGGAUCUGGGCAA

>hsa-mir-1296

ACCUACCUAACUGGGUUAGGGCCCUGGCUCCAUCUCCUUUAGGAAAACCUUCUGUGGGGAGUGGGGCUUCGACCCUAACCCAGGUGGGCUGU

>hsa-mir-1298

AGACGAGGAGUUAAGAGUUCAUUCGGCUGUCCAGAUGUAUCCAAGUACCCUGUGUUAUUUGGCAAUAAAUACAUCUGGGCAACUGACUGAACUUUUCACUUUUCAUGACUCA

>hsa-mir-1299

CCUCAUGGCAGUGUUCUGGAAUCCUACGUGAGGGACAAUCAUUCAGACCCACGUAGCAGUGUUCUGGAAUUCUGUGUGAGGGA

>hsa-mir-1301

GGAUUGUGGGGGGUCGCUCUAGGCACCGCAGCACUGUGCUGGGGAUGUUGCAGCUGCCUGGGAGUGACUUCACACAGUCCUC

>hsa-mir-1302-1

CAGAAAGCCCAGUUAAAUUUGAAUUUCAAGUAAACAAUGAAUAAUUGUGUAUGUAAGAAUAUCCCAUACAAUAUUUGGGACAUACUUAUGCUAAAAAUUAUUCCUUGCUUAUCUGAAAUUCAAAUGUAACUAGGAUUCCUGUA

>hsa-mir-1302-10

GGAUGCCCAGCUAGUUUGAAUUUUAGAUAAACAACGAAUAAUUUCGUAGCAUAAAUAUGUCCCAAGCUUAGUUUGGGACAUACUUAUGCUAAAAAACAUUAUUGGUUGUUUAUCUGAGAUUCAGAAUUAAGCAUUUUA

>hsa-mir-1302-11

GGAUGCCCAGCUAGUUUGAAUUUUAGAUAAACAACGAAUAAUUUCGUAGCAUAAAUAUGUCCCAAGCUUAGUUUGGGACAUACUUAUGCUAAAAAACAUUAUUGGUUGUUUAUCUGAGAUUCAGAAUUAAGCAUUUUA

>hsa-mir-1302-2

GGAUGCCCAGCUAGUUUGAAUUUUAGAUAAACAACGAAUAAUUUCGUAGCAUAAAUAUGUCCCAAGCUUAGUUUGGGACAUACUUAUGCUAAAAAACAUUAUUGGUUGUUUAUCUGAGAUUCAGAAUUAAGCAUUUUA

>hsa-mir-1302-3

GGAUGCCCAGCUAGUUUGAAUUUUAGAUAAACAACGAAUAAUUUCGUAGCAUAAAUAUUUCCCAAGCUUAGUUUGGGACAUACUUAUGCUAAAAAACAUUAUUGGUUGUUUAUCUGAGAUUCAAAAUUAAGCAUUUUA

>hsa-mir-1302-4

AAUGCAGAAGCACAGCUAAAAUUUGAAUUUCAGAUAAACAAAUUUUUCUUAGAAUAAGUAUGUCUCCAUGCAACAUUUGGGACAUACUUAUGCUAAAAUAUUAUUUGUGUUUCAUCUGAAAUUCAAAUUCAACUGGACAUCCUGUAUUUU

>hsa-mir-1302-5

UGCCCGGCCUCCCAUUAAAUUGGUUUUUCAGACAAAUCACAAAUUUGUUUAGGUAUAAGUAUAUCCCAUGUAAUCUUUGGGACAUACUUAUGCUAAAAUAAUUGUUCCUUGUUGAUUGGAAAUUUUAAUUUUAAUUAGGUGUCCUGUAUU

>hsa-mir-1302-6

AACAAAUAAUUUGGUAAUAUAUGUAUGGCCCACACAAUAUUUAGGACAACAAUAUUUGGGACAUACUUAUGCUAAAAAAGUAUUUGUUGA

>hsa-mir-1302-7

ACAACAUGUUUUUAGGACAUGUAUGUCUGGUGCAAUAAUUGGGACAUACUUAUGCUAAAAAAAUUAGUGUUC

>hsa-mir-1302-8

CCCAUUUAAACUUGAAUUUCAUAUAAACACCGUAAUUUUCAGCAUUAGUGUAUCACAUGCAGUAUUUGGGACAUACUUAUGCUAAAAAAUUAGGUGGUGUUGAUCUGAAAUUCCAGUGUAGAUGGGCA

>hsa-mir-1302-9

GGAUGCCCAGCUAGUUUGAAUUUUAGAUAAACAACGAAUAAUUUCGUAGCAUAAAUAUGUCCCAAGCUUAGUUUGGGACAUACUUAUGCUAAAAAACAUUAUUGGUUGUUUAUCUGAGAUUCAGAAUUAAGCAUUUUA

>hsa-mir-1303

GGCUGGGCAACAUAGCGAGACCUCAACUCUACAAUUUUUUUUUUUUUAAAUUUUAGAGACGGGGUCUUGCUCUGUUGCCAGGCUUU

>hsa-mir-1304

AAACACUUGAGCCCAGCGGUUUGAGGCUACAGUGAGAUGUGAUCCUGCCACAUCUCACUGUAGCCUCGAACCCCUGGGCUCAAGUGAUUCA

>hsa-mir-1305

AAGAUCCUGCUGUUUCUACCAUUAGUUUUGAAUGUUUAUUGUAAAGAUACUUUUCAACUCUAAUGGGAGAGACAGCAGGAUUCUCC

>hsa-mir-1306

GUGAGCAGUCUCCACCACCUCCCCUGCAAACGUCCAGUGGUGCAGAGGUAAUGGACGUUGGCUCUGGUGGUGAUGGACAGUCCGA

>hsa-mir-1307

CAUCAAGACCCAGCUGAGUCACUGUCACUGCCUACCAAUCUCGACCGGACCUCGACCGGCUCGUCUGUGUUGCCAAUCGACUCGGCGUGGCGUCGGUCGUGGUAGAUAGGCGGUCAUGCAUACGAAUUUUCAGCUCUUGUUCUGGUGAC

>hsa-mir-130a

UGCUGCUGGCCAGAGCUCUUUUCACAUUGUGCUACUGUCUGCACCUGUCACUAGCAGUGCAAUGUUAAAAGGGCAUUGGCCGUGUAGUG

>hsa-mir-130b

GGCCUGCCCGACACUCUUUCCCUGUUGCACUACUAUAGGCCGCUGGGAAGCAGUGCAAUGAUGAAAGGGCAUCGGUCAGGUC

>hsa-mir-132

CCGCCCCCGCGUCUCCAGGGCAACCGUGGCUUUCGAUUGUUACUGUGGGAACUGGAGGUAACAGUCUACAGCCAUGGUCGCCCCGCAGCACGCCCACGCGC

>hsa-mir-1322

AGUAUCAUGAAUUAGAAACCUACUUAUUACAUAGUUUACAUAAGAAGCGUGAUGAUGCUGCUGAUGCUGUA

>hsa-mir-1323

ACUGAGGUCCUCAAAACUGAGGGGCAUUUUCUGUGGUUUGAAAGGAAAGUGCACCCAGUUUUGGGGAUGUCAA

>hsa-mir-133a-1

ACAAUGCUUUGCUAGAGCUGGUAAAAUGGAACCAAAUCGCCUCUUCAAUGGAUUUGGUCCCCUUCAACCAGCUGUAGCUAUGCAUUGA

>hsa-mir-133a-2

GGGAGCCAAAUGCUUUGCUAGAGCUGGUAAAAUGGAACCAAAUCGACUGUCCAAUGGAUUUGGUCCCCUUCAACCAGCUGUAGCUGUGCAUUGAUGGCGCCG

>hsa-mir-133b

CCUCAGAAGAAAGAUGCCCCCUGCUCUGGCUGGUCAAACGGAACCAAGUCCGUCUUCCUGAGAGGUUUGGUCCCCUUCAACCAGCUACAGCAGGGCUGGCAAUGCCCAGUCCUUGGAGA

>hsa-mir-134

CAGGGUGUGUGACUGGUUGACCAGAGGGGCAUGCACUGUGUUCACCCUGUGGGCCACCUAGUCACCAACCCUC

>hsa-mir-1343

GCUGGCGUCGGUGCUGGGGAGCGGCCCCCGGGUGGGCCUCUGCUCUGGCCCCUCCUGGGGCCCGCACUCUCGCUCUGGGCCCGC

>hsa-mir-135a-1

AGGCCUCGCUGUUCUCUAUGGCUUUUUAUUCCUAUGUGAUUCUACUGCUCACUCAUAUAGGGAUUGGAGCCGUGGCGCACGGCGGGGACA

>hsa-mir-135a-2

AGAUAAAUUCACUCUAGUGCUUUAUGGCUUUUUAUUCCUAUGUGAUAGUAAUAAAGUCUCAUGUAGGGAUGGAAGCCAUGAAAUACAUUGUGAAAAAUCA

>hsa-mir-135b

CACUCUGCUGUGGCCUAUGGCUUUUCAUUCCUAUGUGAUUGCUGUCCCAAACUCAUGUAGGGCUAAAAGCCAUGGGCUACAGUGAGGGGCGAGCUCC

>hsa-mir-136

UGAGCCCUCGGAGGACUCCAUUUGUUUUGAUGAUGGAUUCUUAUGCUCCAUCAUCGUCUCAAAUGAGUCUUCAGAGGGUUCU

>hsa-mir-137

GGUCCUCUGACUCUCUUCGGUGACGGGUAUUCUUGGGUGGAUAAUACGGAUUACGUUGUUAUUGCUUAAGAAUACGCGUAGUCGAGGAGAGUACCAGCGGCA

>hsa-mir-138-1

CCCUGGCAUGGUGUGGUGGGGCAGCUGGUGUUGUGAAUCAGGCCGUUGCCAAUCAGAGAACGGCUACUUCACAACACCAGGGCCACACCACACUACAGG

>hsa-mir-138-2

CGUUGCUGCAGCUGGUGUUGUGAAUCAGGCCGACGAGCAGCGCAUCCUCUUACCCGGCUAUUUCACGACACCAGGGUUGCAUCA

>hsa-mir-139

GUGUAUUCUACAGUGCACGUGUCUCCAGUGUGGCUCGGAGGCUGGAGACGCGGCCCUGUUGGAGUAAC

>hsa-mir-140

UGUGUCUCUCUCUGUGUCCUGCCAGUGGUUUUACCCUAUGGUAGGUUACGUCAUGCUGUUCUACCACAGGGUAGAACCACGGACAGGAUACCGGGGCACC

>hsa-mir-141

CGGCCGGCCCUGGGUCCAUCUUCCAGUACAGUGUUGGAUGGUCUAAUUGUGAAGCUCCUAACACUGUCUGGUAAAGAUGGCUCCCGGGUGGGUUC

>hsa-mir-142

GACAGUGCAGUCACCCAUAAAGUAGAAAGCACUACUAACAGCACUGGAGGGUGUAGUGUUUCCUACUUUAUGGAUGAGUGUACUGUG

>hsa-mir-143

GCGCAGCGCCCUGUCUCCCAGCCUGAGGUGCAGUGCUGCAUCUCUGGUCAGUUGGGAGUCUGAGAUGAAGCACUGUAGCUCAGGAAGAGAGAAGUUGUUCUGCAGC

>hsa-mir-144

UGGGGCCCUGGCUGGGAUAUCAUCAUAUACUGUAAGUUUGCGAUGAGACACUACAGUAUAGAUGAUGUACUAGUCCGGGCACCCCC

>hsa-mir-145

CACCUUGUCCUCACGGUCCAGUUUUCCCAGGAAUCCCUUAGAUGCUAAGAUGGGGAUUCCUGGAAAUACUGUUCUUGAGGUCAUGGUU

>hsa-mir-1468

GGUGGGUGGUUUCUCCGUUUGCCUGUUUCGCUGAUGUGCAUUCAACUCAUUCUCAGCAAAAUAAGCAAAUGGAAAAUUCGUCCAUC

>hsa-mir-146a

CCGAUGUGUAUCCUCAGCUUUGAGAACUGAAUUCCAUGGGUUGUGUCAGUGUCAGACCUCUGAAAUUCAGUUCUUCAGCUGGGAUAUCUCUGUCAUCGU

>hsa-mir-146b

CCUGGCACUGAGAACUGAAUUCCAUAGGCUGUGAGCUCUAGCAAUGCCCUGUGGACUCAGUUCUGGUGCCCGG

>hsa-mir-147b

UAUAAAUCUAGUGGAAACAUUUCUGCACAAACUAGAUUCUGGACACCAGUGUGCGGAAAUGCUUCUGCUACAUUUUUAGG

>hsa-mir-148a

GAGGCAAAGUUCUGAGACACUCCGACUCUGAGUAUGAUAGAAGUCAGUGCACUACAGAACUUUGUCUC

>hsa-mir-148b

CAAGCACGAUUAGCAUUUGAGGUGAAGUUCUGUUAUACACUCAGGCUGUGGCUCUCUGAAAGUCAGUGCAUCACAGAACUUUGUCUCGAAAGCUUUCUA

>hsa-mir-149

GCCGGCGCCCGAGCUCUGGCUCCGUGUCUUCACUCCCGUGCUUGUCCGAGGAGGGAGGGAGGGACGGGGGCUGUGCUGGGGCAGCUGGA

>hsa-mir-150

CUCCCCAUGGCCCUGUCUCCCAACCCUUGUACCAGUGCUGGGCUCAGACCCUGGUACAGGCCUGGGGGACAGGGACCUGGGGAC

>hsa-mir-151a

UUUCCUGCCCUCGAGGAGCUCACAGUCUAGUAUGUCUCAUCCCCUACUAGACUGAAGCUCCUUGAGGACAGGGAUGGUCAUACUCACCUC

>hsa-mir-151b

ACCUCUGAUGUGUCAGUCUCUCUUCAGGGCUCCCGAGACACAGAAACAGACACCUGCCCUCGAGGAGCUCACAGUCUAGACAAACAAACCCAGGGU

>hsa-mir-152

UGUCCCCCCCGGCCCAGGUUCUGUGAUACACUCCGACUCGGGCUCUGGAGCAGUCAGUGCAUGACAGAACUUGGGCCCGGAAGGACC

>hsa-mir-153-1

CUCACAGCUGCCAGUGUCAUUUUUGUGAUCUGCAGCUAGUAUUCUCACUCCAGUUGCAUAGUCACAAAAGUGAUCAUUGGCAGGUGUGGC

>hsa-mir-153-2

AGCGGUGGCCAGUGUCAUUUUUGUGAUGUUGCAGCUAGUAAUAUGAGCCCAGUUGCAUAGUCACAAAAGUGAUCAUUGGAAACUGUG

>hsa-mir-1537

ACAGCUGUAAUUAGUCAGUUUUCUGUCCUGUCCACACAGAAAACCGUCUAGUUACAGUUGU

>hsa-mir-1538

GGGAACAGCAGCAACAUGGGCCUCGCUUCCUGCCGGCGCGGCCCGGGCUGCUGCUGUUCCU

>hsa-mir-155

CUGUUAAUGCUAAUCGUGAUAGGGGUUUUUGCCUCCAACUGACUCCUACAUAUUAGCAUUAACAG

>hsa-mir-15a

CCUUGGAGUAAAGUAGCAGCACAUAAUGGUUUGUGGAUUUUGAAAAGGUGCAGGCCAUAUUGUGCUGCCUCAAAAAUACAAGG

>hsa-mir-15b

UUGAGGCCUUAAAGUACUGUAGCAGCACAUCAUGGUUUACAUGCUACAGUCAAGAUGCGAAUCAUUAUUUGCUGCUCUAGAAAUUUAAGGAAAUUCAU

>hsa-mir-16-1

GUCAGCAGUGCCUUAGCAGCACGUAAAUAUUGGCGUUAAGAUUCUAAAAUUAUCUCCAGUAUUAACUGUGCUGCUGAAGUAAGGUUGAC

>hsa-mir-16-2

GUUCCACUCUAGCAGCACGUAAAUAUUGGCGUAGUGAAAUAUAUAUUAAACACCAAUAUUACUGUGCUGCUUUAGUGUGAC

>hsa-mir-17

GUCAGAAUAAUGUCAAAGUGCUUACAGUGCAGGUAGUGAUAUGUGCAUCUACUGCAGUGAAGGCACUUGUAGCAUUAUGGUGAC

>hsa-mir-181a-1

UGAGUUUUGAGGUUGCUUCAGUGAACAUUCAACGCUGUCGGUGAGUUUGGAAUUAAAAUCAAAACCAUCGACCGUUGAUUGUACCCUAUGGCUAACCAUCAUCUACUCCA

>hsa-mir-181a-2

AGAAGGGCUAUCAGGCCAGCCUUCAGAGGACUCCAAGGAACAUUCAACGCUGUCGGUGAGUUUGGGAUUUGAAAAAACCACUGACCGUUGACUGUACCUUGGGGUCCUUA

>hsa-mir-181b-1

CCUGUGCAGAGAUUAUUUUUUAAAAGGUCACAAUCAACAUUCAUUGCUGUCGGUGGGUUGAACUGUGUGGACAAGCUCACUGAACAAUGAAUGCAACUGUGGCCCCGCUU

>hsa-mir-181b-2

CUGAUGGCUGCACUCAACAUUCAUUGCUGUCGGUGGGUUUGAGUCUGAAUCAACUCACUGAUCAAUGAAUGCAAACUGCGGACCAAACA

>hsa-mir-181c

CGGAAAAUUUGCCAAGGGUUUGGGGGAACAUUCAACCUGUCGGUGAGUUUGGGCAGCUCAGGCAAACCAUCGACCGUUGAGUGGACCCUGAGGCCUGGAAUUGCCAUCCU

>hsa-mir-181d

GUCCCCUCCCCUAGGCCACAGCCGAGGUCACAAUCAACAUUCAUUGUUGUCGGUGGGUUGUGAGGACUGAGGCCAGACCCACCGGGGGAUGAAUGUCACUGUGGCUGGGCCAGACACGGCUUAAGGGGAAUGGGGAC

>hsa-mir-182

GAGCUGCUUGCCUCCCCCCGUUUUUGGCAAUGGUAGAACUCACACUGGUGAGGUAACAGGAUCCGGUGGUUCUAGACUUGCCAACUAUGGGGCGAGGACUCAGCCGGCAC

>hsa-mir-183

CCGCAGAGUGUGACUCCUGUUCUGUGUAUGGCACUGGUAGAAUUCACUGUGAACAGUCUCAGUCAGUGAAUUACCGAAGGGCCAUAAACAGAGCAGAGACAGAUCCACGA

>hsa-mir-184

CCAGUCACGUCCCCUUAUCACUUUUCCAGCCCAGCUUUGUGACUGUAAGUGUUGGACGGAGAACUGAUAAGGGUAGGUGAUUGA

>hsa-mir-1843

GCAGCGAUCCUACAUAAAUAUAUGGAGGUCUCUGUCUGGCUUAGGACAGCUGGCUAAGUCUGAUCGUUCCCCUCCGUACAGCCUUUAAAACUGCCACUCUAAAUACUUGG

>hsa-mir-185

AGGGGGCGAGGGAUUGGAGAGAAAGGCAGUUCCUGAUGGUCCCCUCCCCAGGGGCUGGCUUUCCUCUGGUCCUUCCCUCCCA

>hsa-mir-186

UGCUUGUAACUUUCCAAAGAAUUCUCCUUUUGGGCUUUCUGGUUUUAUUUUAAGCCCAAAGGUGAAUUUUUUGGGAAGUUUGAGCU

>hsa-mir-187

GGUCGGGCUCACCAUGACACAGUGUGAGACCUCGGGCUACAACACAGGACCCGGGCGCUGCUCUGACCCCUCGUGUCUUGUGUUGCAGCCGGAGGGACGCAGGUCCGCA

>hsa-mir-188

UGCUCCCUCUCUCACAUCCCUUGCAUGGUGGAGGGUGAGCUUUCUGAAAACCCCUCCCACAUGCAGGGUUUGCAGGAUGGCGAGCC

>hsa-mir-18a

UGUUCUAAGGUGCAUCUAGUGCAGAUAGUGAAGUAGAUUAGCAUCUACUGCCCUAAGUGCUCCUUCUGGCA

>hsa-mir-18b

UGUGUUAAGGUGCAUCUAGUGCAGUUAGUGAAGCAGCUUAGAAUCUACUGCCCUAAAUGCCCCUUCUGGCA

>hsa-mir-1908

CGGGAAUGCCGCGGCGGGGACGGCGAUUGGUCCGUAUGUGUGGUGCCACCGGCCGCCGGCUCCGCCCCGGCCCCCGCCCC

>hsa-mir-1909

CAUCCAGGACAAUGGUGAGUGCCGGUGCCUGCCCUGGGGCCGUCCCUGCGCAGGGGCCGGGUGCUCACCGCAUCUGCCCC

>hsa-mir-190a

UGCAGGCCUCUGUGUGAUAUGUUUGAUAUAUUAGGUUGUUAUUUAAUCCAACUAUAUAUCAAACAUAUUCCUACAGUGUCUUGCC

>hsa-mir-190b

UGCUUCUGUGUGAUAUGUUUGAUAUUGGGUUGUUUAAUUAGGAACCAACUAAAUGUCAAACAUAUUCUUACAGCAGCAG

>hsa-mir-191

CGGCUGGACAGCGGGCAACGGAAUCCCAAAAGCAGCUGUUGUCUCCAGAGCAUUCCAGCUGCGCUUGGAUUUCGUCCCCUGCUCUCCUGCCU

>hsa-mir-1910

UGUCCCUUCAGCCAGUCCUGUGCCUGCCGCCUUUGUGCUGUCCUUGGAGGGAGGCAGAAGCAGGAUGACAAUGAGGGCAA

>hsa-mir-1911

UCGGCAUCUGCUGAGUACCGCCAUGUCUGUUGGGCAUCCACAGUCUCCCACCAGGCAUUGUGGUCUCCGCUGACGCUUUG

>hsa-mir-1912

CUCUAGGAUGUGCUCAUUGCAUGGGCUGUGUAUAGUAUUAUUCAAUACCCAGAGCAUGCAGUGUGAACAUAAUAGAGAUU

>hsa-mir-1913

ACCUCUACCUCCCGGCAGAGGAGGCUGCAGAGGCUGGCUUUCCAAAACUCUGCCCCCUCCGCUGCUGCCAAGUGGCUGGU

>hsa-mir-1914

CGUGUGAGCCCGCCCUGUGCCCGGCCCACUUCUGCUUCCUCUUAGCGCAGGAGGGGUCCCGCACUGGGAGGGGCCCUCAC

>hsa-mir-1915

UGAGAGGCCGCACCUUGCCUUGCUGCCCGGGCCGUGCACCCGUGGGCCCCAGGGCGACGCGGCGGGGGCGGCCCUAGCGA

>hsa-mir-192

GCCGAGACCGAGUGCACAGGGCUCUGACCUAUGAAUUGACAGCCAGUGCUCUCGUCUCCCCUCUGGCUGCCAAUUCCAUAGGUCACAGGUAUGUUCGCCUCAAUGCCAGC

>hsa-mir-193a

CGAGGAUGGGAGCUGAGGGCUGGGUCUUUGCGGGCGAGAUGAGGGUGUCGGAUCAACUGGCCUACAAAGUCCCAGUUCUCGGCCCCCG

>hsa-mir-193b

GUGGUCUCAGAAUCGGGGUUUUGAGGGCGAGAUGAGUUUAUGUUUUAUCCAACUGGCCCUCAAAGUCCCGCUUUUGGGGUCAU

>hsa-mir-194-1

AUGGUGUUAUCAAGUGUAACAGCAACUCCAUGUGGACUGUGUACCAAUUUCCAGUGGAGAUGCUGUUACUUUUGAUGGUUACCAA

>hsa-mir-194-2

UGGUUCCCGCCCCCUGUAACAGCAACUCCAUGUGGAAGUGCCCACUGGUUCCAGUGGGGCUGCUGUUAUCUGGGGCGAGGGCCAG

>hsa-mir-195

AGCUUCCCUGGCUCUAGCAGCACAGAAAUAUUGGCACAGGGAAGCGAGUCUGCCAAUAUUGGCUGUGCUGCUCCAGGCAGGGUGGUG

>hsa-mir-196a-1

GUGAAUUAGGUAGUUUCAUGUUGUUGGGCCUGGGUUUCUGAACACAACAACAUUAAACCACCCGAUUCAC

>hsa-mir-196a-2

UGCUCGCUCAGCUGAUCUGUGGCUUAGGUAGUUUCAUGUUGUUGGGAUUGAGUUUUGAACUCGGCAACAAGAAACUGCCUGAGUUACAUCAGUCGGUUUUCGUCGAGGGC

>hsa-mir-196b

ACUGGUCGGUGAUUUAGGUAGUUUCCUGUUGUUGGGAUCCACCUUUCUCUCGACAGCACGACACUGCCUUCAUUACUUCAGUUG

>hsa-mir-197

GGCUGUGCCGGGUAGAGAGGGCAGUGGGAGGUAAGAGCUCUUCACCCUUCACCACCUUCUCCACCCAGCAUGGCC

>hsa-mir-1972-1

UAUAGGCAUGUGCCACCACACCUGGCUUAAAUGUGUCAUUUAAAAAUUCAGGCCAGGCACAGUGGCUCAUGCCUGUA

>hsa-mir-1972-2

UAUAGGCAUGUGCCACCACACCUGGCUUAAAUGUGUCAUUUAAAAAUUCAGGCCAGGCACAGUGGCUCAUGCCUGUA

>hsa-mir-1976

GCAGCAAGGAAGGCAGGGGUCCUAAGGUGUGUCCUCCUGCCCUCCUUGCUGU

>hsa-mir-199a-1

GCCAACCCAGUGUUCAGACUACCUGUUCAGGAGGCUCUCAAUGUGUACAGUAGUCUGCACAUUGGUUAGGC

>hsa-mir-199a-2

AGGAAGCUUCUGGAGAUCCUGCUCCGUCGCCCCAGUGUUCAGACUACCUGUUCAGGACAAUGCCGUUGUACAGUAGUCUGCACAUUGGUUAGACUGGGCAAGGGAGAGCA

>hsa-mir-199b

CCAGAGGACACCUCCACUCCGUCUACCCAGUGUUUAGACUAUCUGUUCAGGACUCCCAAAUUGUACAGUAGUCUGCACAUUGGUUAGGCUGGGCUGGGUUAGACCCUCGG

>hsa-mir-19a

GCAGUCCUCUGUUAGUUUUGCAUAGUUGCACUACAAGAAGAAUGUAGUUGUGCAAAUCUAUGCAAAACUGAUGGUGGCCUGC

>hsa-mir-19b-1

CACUGUUCUAUGGUUAGUUUUGCAGGUUUGCAUCCAGCUGUGUGAUAUUCUGCUGUGCAAAUCCAUGCAAAACUGACUGUGGUAGUG

>hsa-mir-19b-2

ACAUUGCUACUUACAAUUAGUUUUGCAGGUUUGCAUUUCAGCGUAUAUAUGUAUAUGUGGCUGUGCAAAUCCAUGCAAAACUGAUUGUGAUAAUGU

>hsa-mir-200a

CCGGGCCCCUGUGAGCAUCUUACCGGACAGUGCUGGAUUUCCCAGCUUGACUCUAACACUGUCUGGUAACGAUGUUCAAAGGUGACCCGC

>hsa-mir-200b

CCAGCUCGGGCAGCCGUGGCCAUCUUACUGGGCAGCAUUGGAUGGAGUCAGGUCUCUAAUACUGCCUGGUAAUGAUGACGGCGGAGCCCUGCACG

>hsa-mir-200c

CCCUCGUCUUACCCAGCAGUGUUUGGGUGCGGUUGGGAGUCUCUAAUACUGCCGGGUAAUGAUGGAGG

>hsa-mir-202

CGCCUCAGAGCCGCCCGCCGUUCCUUUUUCCUAUGCAUAUACUUCUUUGAGGAUCUGGCCUAAAGAGGUAUAGGGCAUGGGAAAACGGGGCGGUCGGGUCCUCCCCAGCG

>hsa-mir-203a

GUGUUGGGGACUCGCGCGCUGGGUCCAGUGGUUCUUAACAGUUCAACAGUUCUGUAGCGCAAUUGUGAAAUGUUUAGGACCACUAGACCCGGCGGGCGCGGCGACAGCGA

>hsa-mir-203b

GCGCCCGCCGGGUCUAGUGGUCCUAAACAUUUCACAAUUGCGCUACAGAACUGUUGAACUGUUAAGAACCACUGGACCCAGCGCGC

>hsa-mir-204

GGCUACAGUCUUUCUUCAUGUGACUCGUGGACUUCCCUUUGUCAUCCUAUGCCUGAGAAUAUAUGAAGGAGGCUGGGAAGGCAAAGGGACGUUCAAUUGUCAUCACUGGC

>hsa-mir-205

AAAGAUCCUCAGACAAUCCAUGUGCUUCUCUUGUCCUUCAUUCCACCGGAGUCUGUCUCAUACCCAACCAGAUUUCAGUGGAGUGAAGUUCAGGAGGCAUGGAGCUGACA

>hsa-mir-206

UGCUUCCCGAGGCCACAUGCUUCUUUAUAUCCCCAUAUGGAUUACUUUGCUAUGGAAUGUAAGGAAGUGUGUGGUUUCGGCAAGUG

>hsa-mir-208a

UGACGGGCGAGCUUUUGGCCCGGGUUAUACCUGAUGCUCACGUAUAAGACGAGCAAAAAGCUUGUUGGUCA

>hsa-mir-208b

CCUCUCAGGGAAGCUUUUUGCUCGAAUUAUGUUUCUGAUCCGAAUAUAAGACGAACAAAAGGUUUGUCUGAGGGCAG

>hsa-mir-20a

GUAGCACUAAAGUGCUUAUAGUGCAGGUAGUGUUUAGUUAUCUACUGCAUUAUGAGCACUUAAAGUACUGC

>hsa-mir-20b

AGUACCAAAGUGCUCAUAGUGCAGGUAGUUUUGGCAUGACUCUACUGUAGUAUGGGCACUUCCAGUACU

>hsa-mir-21

UGUCGGGUAGCUUAUCAGACUGAUGUUGACUGUUGAAUCUCAUGGCAACACCAGUCGAUGGGCUGUCUGACA

>hsa-mir-210

ACCCGGCAGUGCCUCCAGGCGCAGGGCAGCCCCUGCCCACCGCACACUGCGCUGCCCCAGACCCACUGUGCGUGUGACAGCGGCUGAUCUGUGCCUGGGCAGCGCGACCC

>hsa-mir-211

UCACCUGGCCAUGUGACUUGUGGGCUUCCCUUUGUCAUCCUUCGCCUAGGGCUCUGAGCAGGGCAGGGACAGCAAAGGGGUGCUCAGUUGUCACUUCCCACAGCACGGAG

>hsa-mir-2110

CAGGGGUUUGGGGAAACGGCCGCUGAGUGAGGCGUCGGCUGUGUUUCUCACCGCGGUCUUUUCCUCCCACUCUUG

>hsa-mir-2113

UUUUCAAAGCAAUGUGUGACAGGUACAGGGACAAAUCCCGUUAAUAAGUAAGAGGAUUUGUGCUUGGCUCUGUCACAUGCCACUUUGAAAA

>hsa-mir-2114

CCUCCAUGCUCCUAGUCCCUUCCUUGAAGCGGUCGGAUAAUCACAUGACGAGCCUCAAGCAAGGGACUUCAAGCUGGUGG

>hsa-mir-2115

ACUGUCAUCCCACUGCUUCCAGCUUCCAUGACUCCUGAUGGAGGAAUCACAUGAAUUCAUCAGAAUUCAUGGAGGCUAGAAGCAGUAUGAGGAUCAUUUA

>hsa-mir-2116

GACCUAGGCUAGGGGUUCUUAGCAUAGGAGGUCUUCCCAUGCUAAGAAGUCCUCCCAUGCCAAGAACUCCCAGACUAGGA

>hsa-mir-212

CGGGGCACCCCGCCCGGACAGCGCGCCGGCACCUUGGCUCUAGACUGCUUACUGCCCGGGCCGCCCUCAGUAACAGUCUCCAGUCACGGCCACCGACGCCUGGCCCCGCC

>hsa-mir-214

GGCCUGGCUGGACAGAGUUGUCAUGUGUCUGCCUGUCUACACUUGCUGUGCAGAACAUCCGCUCACCUGUACAGCAGGCACAGACAGGCAGUCACAUGACAACCCAGCCU

>hsa-mir-215

AUCAUUCAGAAAUGGUAUACAGGAAAAUGACCUAUGAAUUGACAGACAAUAUAGCUGAGUUUGUCUGUCAUUUCUUUAGGCCAAUAUUCUGUAUGACUGUGCUACUUCAA

>hsa-mir-216a

GAUGGCUGUGAGUUGGCUUAAUCUCAGCUGGCAACUGUGAGAUGUUCAUACAAUCCCUCACAGUGGUCUCUGGGAUUAUGCUAAACAGAGCAAUUUCCUAGCCCUCACGA

>hsa-mir-216b

GCAGACUGGAAAAUCUCUGCAGGCAAAUGUGAUGUCACUGAGGAAAUCACACACUUACCCGUAGAGAUUCUACAGUCUGACA

>hsa-mir-217

AGUAUAAUUAUUACAUAGUUUUUGAUGUCGCAGAUACUGCAUCAGGAACUGAUUGGAUAAGAAUCAGUCACCAUCAGUUCCUAAUGCAUUGCCUUCAGCAUCUAAACAAG

>hsa-mir-218-1

GUGAUAAUGUAGCGAGAUUUUCUGUUGUGCUUGAUCUAACCAUGUGGUUGCGAGGUAUGAGUAAAACAUGGUUCCGUCAAGCACCAUGGAACGUCACGCAGCUUUCUACA

>hsa-mir-218-2

GACCAGUCGCUGCGGGGCUUUCCUUUGUGCUUGAUCUAACCAUGUGGUGGAACGAUGGAAACGGAACAUGGUUCUGUCAAGCACCGCGGAAAGCACCGUGCUCUCCUGCA

>hsa-mir-219a-1

CCGCCCCGGGCCGCGGCUCCUGAUUGUCCAAACGCAAUUCUCGAGUCUAUGGCUCCGGCCGAGAGUUGAGUCUGGACGUCCCGAGCCGCCGCCCCCAAACCUCGAGCGGG

>hsa-mir-219a-2

ACUCAGGGGCUUCGCCACUGAUUGUCCAAACGCAAUUCUUGUACGAGUCUGCGGCCAACCGAGAAUUGUGGCUGGACAUCUGUGGCUGAGCUCCGGG

>hsa-mir-219b

GGAGCUCAGCCACAGAUGUCCAGCCACAAUUCUCGGUUGGCCGCAGACUCGUACAAGAAUUGCGUUUGGACAAUCAGUGGCGAAGCCC

>hsa-mir-22

GGCUGAGCCGCAGUAGUUCUUCAGUGGCAAGCUUUAUGUCCUGACCCAGCUAAAGCUGCCAGUUGAAGAACUGUUGCCCUCUGCC

>hsa-mir-221

UGAACAUCCAGGUCUGGGGCAUGAACCUGGCAUACAAUGUAGAUUUCUGUGUUCGUUAGGCAACAGCUACAUUGUCUGCUGGGUUUCAGGCUACCUGGAAACAUGUUCUC

>hsa-mir-222

GCUGCUGGAAGGUGUAGGUACCCUCAAUGGCUCAGUAGCCAGUGUAGAUCCUGUCUUUCGUAAUCAGCAGCUACAUCUGGCUACUGGGUCUCUGAUGGCAUCUUCUAGCU

>hsa-mir-223

CCUGGCCUCCUGCAGUGCCACGCUCCGUGUAUUUGACAAGCUGAGUUGGACACUCCAUGUGGUAGAGUGUCAGUUUGUCAAAUACCCCAAGUGCGGCACAUGCUUACCAG

>hsa-mir-224

GGGCUUUCAAGUCACUAGUGGUUCCGUUUAGUAGAUGAUUGUGCAUUGUUUCAAAAUGGUGCCCUAGUGACUACAAAGCCC

>hsa-mir-2276

GUGUUCUUCCAGUCCGCCCUCUGUCACCUUGCAGACGGCUUUCUCUCCGAAUGUCUGCAAGUGUCAGAGGCGAGGAGUGGCAGCUGCAU

>hsa-mir-2277

GUGCUUCCUGCGGGCUGAGCGCGGGCUGAGCGCUGCCAGUCAGCGCUCACAUUAAGGCUGACAGCGCCCUGCCUGGCUCGGCCGGCGAAGCUC

>hsa-mir-2278

GUGCUGCAGGUGUUGGAGAGCAGUGUGUGUUGCCUGGGGACUGUGUGGACUGGUAUCACCCAGACAGCUUGCACUGACUCCAGACCCUGCCGUCAU

>hsa-mir-2355

CAGACGUGUCAUCCCCAGAUACAAUGGACAAUAUGCUAUUAUAAUCGUAUGGCAUUGUCCUUGCUGUUUGGAGAUAAUACUGCUGAC

>hsa-mir-23a

GGCCGGCUGGGGUUCCUGGGGAUGGGAUUUGCUUCCUGUCACAAAUCACAUUGCCAGGGAUUUCCAACCGACC

>hsa-mir-23b

CUCAGGUGCUCUGGCUGCUUGGGUUCCUGGCAUGCUGAUUUGUGACUUAAGAUUAAAAUCACAUUGCCAGGGAUUACCACGCAACCACGACCUUGGC

>hsa-mir-23c

AGUGACUUUCCAGGUGUCACACAGUGAGUGGCAUAAUCAGAGUACAAUUUGAGUCAUGCCCAUACAUCACAUUGCCAGUGAUUACCCAAGGAAAGUGACG

>hsa-mir-24-1

CUCCGGUGCCUACUGAGCUGAUAUCAGUUCUCAUUUUACACACUGGCUCAGUUCAGCAGGAACAGGAG

>hsa-mir-24-2

CUCUGCCUCCCGUGCCUACUGAGCUGAAACACAGUUGGUUUGUGUACACUGGCUCAGUUCAGCAGGAACAGGG

>hsa-mir-2467

GGACAGGCACCUGAGGCUCUGUUAGCCUUGGCUCUGGGUCCUGCUCCUUAGAGCAGAGGCAGAGAGGCUCAGGGUCUGUCU

>hsa-mir-25

GGCCAGUGUUGAGAGGCGGAGACUUGGGCAAUUGCUGGACGCUGCCCUGGGCAUUGCACUUGUCUCGGUCUGACAGUGCCGGCC

>hsa-mir-2682

ACCUUCCUGAAAGAGGUUGGGGCAGGCAGUGACUGUUCAGACGUCCAAUCUCUUUGGGACGCCUCUUCAGCGCUGUCUUCCCUGCCUCUGCCUUUAGGACGAGUCUCAAA

>hsa-mir-26a-1

GUGGCCUCGUUCAAGUAAUCCAGGAUAGGCUGUGCAGGUCCCAAUGGGCCUAUUCUUGGUUACUUGCACGGGGACGC

>hsa-mir-26a-2

GGCUGUGGCUGGAUUCAAGUAAUCCAGGAUAGGCUGUUUCCAUCUGUGAGGCCUAUUCUUGAUUACUUGUUUCUGGAGGCAGCU

>hsa-mir-26b

CCGGGACCCAGUUCAAGUAAUUCAGGAUAGGUUGUGUGCUGUCCAGCCUGUUCUCCAUUACUUGGCUCGGGGACCGG

>hsa-mir-27a

CUGAGGAGCAGGGCUUAGCUGCUUGUGAGCAGGGUCCACACCAAGUCGUGUUCACAGUGGCUAAGUUCCGCCCCCCAG

>hsa-mir-27b

ACCUCUCUAACAAGGUGCAGAGCUUAGCUGAUUGGUGAACAGUGAUUGGUUUCCGCUUUGUUCACAGUGGCUAAGUUCUGCACCUGAAGAGAAGGUG

>hsa-mir-28

GGUCCUUGCCCUCAAGGAGCUCACAGUCUAUUGAGUUACCUUUCUGACUUUCCCACUAGAUUGUGAGCUCCUGGAGGGCAGGCACU

>hsa-mir-296

AGGACCCUUCCAGAGGGCCCCCCCUCAAUCCUGUUGUGCCUAAUUCAGAGGGUUGGGUGGAGGCUCUCCUGAAGGGCUCU

>hsa-mir-29a

AUGACUGAUUUCUUUUGGUGUUCAGAGUCAAUAUAAUUUUCUAGCACCAUCUGAAAUCGGUUAU

>hsa-mir-29b-1

CUUCAGGAAGCUGGUUUCAUAUGGUGGUUUAGAUUUAAAUAGUGAUUGUCUAGCACCAUUUGAAAUCAGUGUUCUUGGGGG

>hsa-mir-29b-2

CUUCUGGAAGCUGGUUUCACAUGGUGGCUUAGAUUUUUCCAUCUUUGUAUCUAGCACCAUUUGAAAUCAGUGUUUUAGGAG

>hsa-mir-29c

AUCUCUUACACAGGCUGACCGAUUUCUCCUGGUGUUCAGAGUCUGUUUUUGUCUAGCACCAUUUGAAAUCGGUUAUGAUGUAGGGGGA

>hsa-mir-301a

ACUGCUAACGAAUGCUCUGACUUUAUUGCACUACUGUACUUUACAGCUAGCAGUGCAAUAGUAUUGUCAAAGCAUCUGAAAGCAGG

>hsa-mir-301b

GCCGCAGGUGCUCUGACGAGGUUGCACUACUGUGCUCUGAGAAGCAGUGCAAUGAUAUUGUCAAAGCAUCUGGGACCA

>hsa-mir-302a

CCACCACUUAAACGUGGAUGUACUUGCUUUGAAACUAAAGAAGUAAGUGCUUCCAUGUUUUGGUGAUGG

>hsa-mir-302b

GCUCCCUUCAACUUUAACAUGGAAGUGCUUUCUGUGACUUUAAAAGUAAGUGCUUCCAUGUUUUAGUAGGAGU

>hsa-mir-302c

CCUUUGCUUUAACAUGGGGGUACCUGCUGUGUGAAACAAAAGUAAGUGCUUCCAUGUUUCAGUGGAGG

>hsa-mir-302d

CCUCUACUUUAACAUGGAGGCACUUGCUGUGACAUGACAAAAAUAAGUGCUUCCAUGUUUGAGUGUGG

>hsa-mir-3059

AGGUGGUACACCCUUUCCUCUCUGCCCCAUAGGGUGUAGCUCUAACUACCCUCUAGGGAAGAGAAGGUUGGGUGAACAGCCU

>hsa-mir-3064

GGUCUGGCUGUUGUGGUGUGCAAAACUCCGUACAUUGCUAUUUUGCCACACUGCAACACCUUACAG

>hsa-mir-3065

CUGCCCUCUUCAACAAAAUCACUGAUGCUGGAGUCGCCUGAGUCAUCACUCAGCACCAGGAUAUUGUUGGAGAGGACAG

>hsa-mir-3074

GCUCGACUCCUGUUCCUGCUGAACUGAGCCAGUGUGUAAAAUGAGAACUGAUAUCAGCUCAGUAGGCACCGGAGGGCGGGU

>hsa-mir-30a

GCGACUGUAAACAUCCUCGACUGGAAGCUGUGAAGCCACAGAUGGGCUUUCAGUCGGAUGUUUGCAGCUGC

>hsa-mir-30b

ACCAAGUUUCAGUUCAUGUAAACAUCCUACACUCAGCUGUAAUACAUGGAUUGGCUGGGAGGUGGAUGUUUACUUCAGCUGACUUGGA

>hsa-mir-30c-1

ACCAUGCUGUAGUGUGUGUAAACAUCCUACACUCUCAGCUGUGAGCUCAAGGUGGCUGGGAGAGGGUUGUUUACUCCUUCUGCCAUGGA

>hsa-mir-30c-2

AGAUACUGUAAACAUCCUACACUCUCAGCUGUGGAAAGUAAGAAAGCUGGGAGAAGGCUGUUUACUCUUUCU

>hsa-mir-30d

GUUGUUGUAAACAUCCCCGACUGGAAGCUGUAAGACACAGCUAAGCUUUCAGUCAGAUGUUUGCUGCUAC

>hsa-mir-30e

GGGCAGUCUUUGCUACUGUAAACAUCCUUGACUGGAAGCUGUAAGGUGUUCAGAGGAGCUUUCAGUCGGAUGUUUACAGCGGCAGGCUGCCA

>hsa-mir-31

GGAGAGGAGGCAAGAUGCUGGCAUAGCUGUUGAACUGGGAACCUGCUAUGCCAACAUAUUGCCAUCUUUCC

>hsa-mir-3115

UCUGAAUAUGGGUUUACUAGUUGGUGGUGAAUUCAUGAGUCGCCAACUAUUAGGCCUUUAUGUCCAGA

>hsa-mir-3117

CCCUAAAGGGCCAGACACUAUACGAGUCAUAUAAGGGAAGGCAUUAUAGGACUCAUAUAGUGCCAGGUGUUUUGUGGG

>hsa-mir-3118-1

CACACAUACAAUAAUAUUCAUAAUGCAAUCACACACAAUCACCAUGUGACUGCAUUAUGAAAAUUCUUCUAGUGUG

>hsa-mir-3118-2

CAUACUACAAUAAUUUUCAUAAUGCAAUCACACACAAUCACCGUGUGACUGCAUUAUGAAAAUUCUUCUAGUGUG

>hsa-mir-3118-3

CAUACUACAAUAAUUUUCAUAAUGCAAUCACACACAAUCACCGUGUGACUGCAUUAUGAAAAUUCUUCUAGUGUG

>hsa-mir-3118-4

CAUACUACAAUAAUUUUCAUAAUGCAAUCACACACAAUCACCGUGUGACUGCAUUAUGAAAAUUCUUCUAGUGUG

>hsa-mir-3120

GUCAUGUGACUGCCUGUCUGUGCCUGCUGUACAGGUGAGCGGAUGUUCUGCACAGCAAGUGUAGACAGGCAGACACAUGAC

>hsa-mir-3121

AAAUGGUUAUGUCCUUUGCCUAUUCUAUUUAAGACACCCUGUACCUUAAAUAGAGUAGGCAAAGGACAGAAACAUUU

>hsa-mir-3122

ACCAGCUCUGUUGGGACAAGAGGACGGUCUUCUUUUGGAAGGAAGACCAUCAUCUUGUCCGAAGAGAGCUGGU

>hsa-mir-3124

GCGGGCUUCGCGGGCGAAGGCAAAGUCGAUUUCCAAAAGUGACUUUCCUCACUCCCGUGAAGUCGGC

>hsa-mir-3126

AUGAUUAUAUGAGGGACAGAUGCCAGAAGCACUGGUUAUGAUUUGCAUCUGGCAUCCGUCACACAGAUAAUUAU

>hsa-mir-3127

GGCCAGGCCCAUCAGGGCUUGUGGAAUGGGAAGGAGAAGGGACGCUUCCCCUUCUGCAGGCCUGCUGGGUGUGGCU

>hsa-mir-3128

UUCCUCUGGCAAGUAAAAAACUCUCAUUUUCCUUAAAAAAUGAGAGUUUUUUACUUGCAAUAGGAA

>hsa-mir-3129

GUACUUGGGCAGUAGUGUAGAGAUUGGUUUGCCUGUUAAUGAAUUCAAACUAAUCUCUACACUGCUGCCCAAGAGC

>hsa-mir-3130-1

CUUGUCAUGUCUUACCCAGUCUCCGGUGCAGCCUGUUGUCAAGGCUGCACCGGAGACUGGGUAAGACAUGACAAG

>hsa-mir-3130-2

CUUGUCAUGUCUUACCCAGUCUCCGGUGCAGCCUUGACAACAGGCUGCACCGGAGACUGGGUAAGACAUGACAAG

>hsa-mir-3132

GGUGGGAUGGGUAGAGAAGGAGCUCAGAGGACGGUGCGCCUUGUUUCCCUUGAGCCCUCCCUCUCUCAUCCCACC

>hsa-mir-3133

CAGAAAUUGUAAAGAACUCUUAAAACCCAAUAGUAAAAAGACAACCUGUUGAGUUUUAAGAGUUCUUUAUAUAUUCUG

>hsa-mir-3134

UGUAUCCAAUGUGUAGUCUUUUAUCCCUCACAUGGAGUAAAAUAUGAUGGAUAAAAGACUACAUAUUGGGUACA

>hsa-mir-3135a

UCACUUUGGUGCCUAGGCUGAGACUGCAGUGGUGCAAUCUCAGUUCACUGCAGCCUUGACCUCCUGGGCUCAGGUGA

>hsa-mir-3135b

UGCCCAGGCUGGAGCGAGUGCAGUGGUGCAGUCAGUCCUAGCUCACUGCAGCCUCGAACUCCUGGGCU

>hsa-mir-3136

AAUAUGAAACUGACUGAAUAGGUAGGGUCAUUUUUCUGUGACUGCACAUGGCCCAACCUAUUCAGUUAGUUCCAUAUU

>hsa-mir-3137

UACAGGUCUGUAGCCUGGGAGCAAUGGGGUGUAUGGUAUAGGGGUAGCCUCGUGCUCCUGGGCUACAAACCUGUA

>hsa-mir-3138

CCCUCCUCGGCACUUCCCCCACCUCACUGCCCGGGUGCCCACAAGACUGUGGACAGUGAGGUAGAGGGAGUGCCGAGGAGGG

>hsa-mir-3139

GGCUCAGAGUAGGAGCUCAACAGAUGCCUGUUGACUGAAUAAUAAACAGGUAUCGCAGGAGCUUUUGUUAUGUGCC

>hsa-mir-3140

CCUCUUGAGGUACCUGAAUUACCAAAAGCUUUAUGUAUUCUGAAGUUAUUGAAAAUAAGAGCUUUUGGGAAUUCAGGUAGUUCAGGAGUG

>hsa-mir-3141

UCACCCGGUGAGGGCGGGUGGAGGAGGAGGGUCCCCACCAUCAGCCUUCACUGGGACGGGA

>hsa-mir-3142

UUCAGAAAGGCCUUUCUGAACCUUCAGAAAGGCUGCUGAAUCUUCAGAAAGGCCUUUCUGAACCUUCAGAAAGGCUGCUGAA

>hsa-mir-3143

UAGAUAACAUUGUAAAGCGCUUCUUUCGCGGUUGGGCUGGAGCAACUCUUUACAAUGUUUCUA

>hsa-mir-3144

AACUACACUUUAAGGGGACCAAAGAGAUAUAUAGAUAUCAGCUACCUAUAUACCUGUUCGGUCUCUUUAAAGUGUAGUU

>hsa-mir-3145

UAUAUGAGUUCAACUCCAAACACUCAAAACUCAUUGUUGAAUGGAAUGAGAUAUUUUGAGUGUUUGGAAUUGAACUCGUAUA

>hsa-mir-3146

GCUAAGUCCCUUCUUUCUAUCCUAGUAUAACUUGAAGAAUUCAAAUAGUCAUGCUAGGAUAGAAAGAAUGGGACUUGGC

>hsa-mir-3148

GAGUUAAGAUGGAAAAAACUGGUGUGUGCUUAUUGAUGUAGCCAACAAGCAUACAUCAGUUUUUUCCAACUUAACUC

>hsa-mir-3149

AUACAUACAUGUACACACACAUGUCAUCCACACACAUACAUAUAUAUAUGUUUGUAUGGAUAUGUGUGUGUAUGUGUGUGUAU

>hsa-mir-3150a

GGGAAGCAGGCCAACCUCGACGAUCUCCUCAGCACCUGAACGCCAAGGCUGGGGAGAUCCUCGAGGUUGGCCUGCUUUCC

>hsa-mir-3150b

GAGGGAAAGCAGGCCAACCUCGAGGAUCUCCCCAGCCUUGGCGUUCAGGUGCUGAGGAGAUCGUCGAGGUUGGCCUGCUUCCCCUC

>hsa-mir-3151

GGGGUGAUGGGUGGGGCAAUGGGAUCAGGUGCCUCAAAGGGCAUCCCACCUGAUCCCACAGCCCACCUGUCACCCC

>hsa-mir-3154

GGCCCCUCCUUCUCAGCCCCAGCUCCCGCUCACCCCUGCCACGUCAAAGGAGGCAGAAGGGGAGUUGGGAGCAGAGAGGGGACC

>hsa-mir-3155a

UCCGGGCAUCACCUCCCACUGCAGAGCCUGGGGAGCCGGACAGCUCCCUUCCCAGGCUCUGCAGUGGGAACUGAUGCCUGGA

>hsa-mir-3155b

CCACUGCAGAGCCUGGGAAGGGAGCUGUCCGGCUCCCCAGGCUCUGCAGUGGGAGG

>hsa-mir-3157

GGGAAGGGCUUCAGCCAGGCUAGUGCAGUCUGCUUUGUGCCAACACUGGGGUGAUGACUGCCCUAGUCUAGCUGAAGCUUUUCCC

>hsa-mir-3158-1

AUUCAGGCCGGUCCUGCAGAGAGGAAGCCCUUCUGCUUACAGGUAUUGGAAGGGCUUCCUCUCUGCAGGACCGGCCUGAAU

>hsa-mir-3158-2

AUUCAGGCCGGUCCUGCAGAGAGGAAGCCCUUCCAAUACCUGUAAGCAGAAGGGCUUCCUCUCUGCAGGACCGGCCUGAAU

>hsa-mir-3159

CCAAAGUCCUAGGAUUACAAGUGUCGGCCACGGGCUGGGCACAGUGGCUCACGCCUGUAAUCCCAGCAUUUUGG

>hsa-mir-3160-1

GGACCUGCCCUGGGCUUUCUAGUCUCAGCUCUCCUCCAGCUCAGCUGGUCAGGAGAGCUGAGACUAGAAAGCCCAGGGCAGGUUC

>hsa-mir-3160-2

ACCUGCCCUGGGCUUUCUAGUCUCAGCUCUCCUGACCAGCUGAGCUGGAGGAGAGCUGAGACUAGAAAGCCCAGGGCAGGU

>hsa-mir-3162

CUGACUUUUUUAGGGAGUAGAAGGGUGGGGAGCAUGAACAAUGUUUCUCACUCCCUACCCCUCCACUCCCCAAAAAAGUCAG

>hsa-mir-3163

UUCCUCAUCUAUAAAAUGAGGGCAGUAAGACCUUCCUUCCUUGUCUUACUACCCCCAUUUUAUAGAUGAGGAA

>hsa-mir-3164

CUUGGAAACUGUGACUUUAAGGGAAAUGGCGCACAGCAGACCCUGCAAUCAUGCCGUUUUGCUUGAAGUCGCAGUUUCCCAGG

>hsa-mir-3165

CCGGUGGCAAGGUGGAUGCAAUGUGACCUCAACUCUUGGUCCUCUGAGGUCACAUUGUAUCCACCUUACCACUGG

>hsa-mir-3170

CUGGUAACACUGGGGUUCUGAGACAGACAGUGUUAGCUCCAGAAGCAUUGCCUGUCUUAGAACCCCUAUGUUACCAG

>hsa-mir-3173

UCCCUGCCCUGCCUGUUUUCUCCUUUGUGAUUUUAUGAGAACAAAGGAGGAAAUAGGCAGGCCAGGGA

>hsa-mir-3174

GUUACCUGGUAGUGAGUUAGAGAUGCAGAGCCCUGGGCUCCUCAGCAAACCUACUGGAUCUGCAUUUUAAUUCACAUGCAUGGUAAU

>hsa-mir-3175

CCUGGGGGGCGGGGAGAGAACGCAGUGACGUCUGGCCGCGUGCGCAUGUCGGGCGCUUUCUCCUCCCCCUACCCAGG

>hsa-mir-3176

UGGCCUCUCCAGUCUGCAGCUCCCGGCAGCCUCGGGCCACACUCCCGGGAUCCCCAGGGACUGGCCUGGGACUACCGGGGGUGGCGGCCG

>hsa-mir-3177

CCACGUGCCAUGUGUACACACGUGCCAGGCGCUGUCUUGAGACAUUCGCGCAGUGCACGGCACUGGGGACACGUGGCACUGG

>hsa-mir-3179-1

CAGGAUCACAGACGUUUAAAUUACACUCCUUCUGCUGUGCCUUACAGCAGUAGAAGGGGUGAAAUUUAAACGUCUGUGAUCCUG

>hsa-mir-3179-2

CAGGAUCACAGACGUUUAAAUUACACUCCUUCUGCUGUGCCUUACAGCAGUAGAAGGGGUGAAAUUUAAACGUCUGUGAUCCUG

>hsa-mir-3179-3

CAGGAUCACAGACGUUUAAAUUACACUCCUUCUGCUGUGCCUUACAGCAGUAGAAGGGGUGAAAUUUAAACGUCUGUGAUCCUG

>hsa-mir-3179-4

CAGGAUCACAGACGUUUAAAUUACACUCCUUCUGCUGUGCCUUACAGCAGUAGAAGGGGUGAAAUUUAAACGUCUGUGAUCCUG

>hsa-mir-3180-1

CAGUGCGACGGGCGGAGCUUCCAGACGCUCCGCCCCACGUCGCAUGCGCCCCGGGAAAGCGUGGGGCGGAGCUUCCGGAGGCCCCGCCCUGCUG

>hsa-mir-3180-2

GCGACGGGCGGAGCUUCCAGACGCUCCGCCCCACGUCGCAUGCGCCCCGGGAAAGCGUGGGGCGGAGCUUCCGGAGGCCCCGCCCUGC

>hsa-mir-3180-3

CAGUGCGACGGGCGGAGCUUCCAGACGCUCCGCCCCACGUCGCAUGCGCCCCGGGAAAGCGUGGGGCGGAGCUUCCGGAGGCCCCGCCCUGCUG

>hsa-mir-3180-4

GCUCCGCCCCACGUCGCAUGCGCCCCGGGAACGCGUGGGGCGGAGCUUCCGGAGGCCCCGCUCUGCUGCCGACCCUGUGGAGCGGAGGGUGAAGCCUCCGGAUGCCAGUCCCUCAUCGCUGGCCUGGUCGCGCUGUGGCGAAGGGGGCGGAGC

>hsa-mir-3180-5

GCUCCGCCCCACGUCGCAUGCGCCCCGGGAACGCGUGGGGCGGAGCUUCCGGAGGCCCCGCCCUGCUGCCGACCCUGUGGAGCGGAGGGUGAAGCCUCCGGAUGCCAGUCCCUCAUCGCUGGCCCGGUCGCGCUGUGGCGAAGGGGGCGGAGC

>hsa-mir-3181

CGGCGACCAUCGGGCCCUCGGCGCCGGCCCGUUAGUUGCCCGGGCCCGAGCCGGCCGGGCCCGCGGGUUGCCG

>hsa-mir-3183

CUCUGCCCUGCCUCUCUCGGAGUCGCUCGGAGCAGUCACGUUGACGGAAUCCUCCGGCGCCUCCUCGAGGGAGGAGAGGCAGGG

>hsa-mir-3184

AAGCAAGACUGAGGGGCCUCAGACCGAGCUUUUGGAAAAUAGAAAAGUCUCGCUCUCUGCCCCUCAGCCUAACUU

>hsa-mir-3186

AGCCUGCGGUUCCAACAGGCGUCUGUCUACGUGGCUUCAACCAAGUUCAAAGUCACGCGGAGAGAUGGCUUUGGAACCAGGGGCU

>hsa-mir-3187

GCUGGCCCUGGGCAGCGUGUGGCUGAAGGUCACCAUGUUCUCCUUGGCCAUGGGGCUGCGCGGGGCCAGC

>hsa-mir-3188

GGCGCCUCCUGCUCUGCUGUGCCGCCAGGGCCUCCCCUAGCGCGCCUUCUGGAGAGGCUUUGUGCGGAUACGGGGCUGGAGGCCU

>hsa-mir-3189

GCCUCAGUUGCCCCAUCUGUGCCCUGGGUAGGAAUAUCCUGGAUCCCCUUGGGUCUGAUGGGGUAGCCGAUGC

>hsa-mir-3190

CUGGGGUCACCUGUCUGGCCAGCUACGUCCCCACGGCCCUUGUCAGUGUGGAAGGUAGACGGCCAGAGAGGUGACCCCGG

>hsa-mir-3191

GGGGUCACCUCUCUGGCCGUCUACCUUCCACACUGACAAGGGCCGUGGGGACGUAGCUGGCCAGACAGGUGACCCC

>hsa-mir-3192

GGAAGGGAUUCUGGGAGGUUGUAGCAGUGGAAAAAGUUCUUUUCUUCCUCUGAUCGCCCUCUCAGCUCUUUCCUUCU

>hsa-mir-3194

AGGUGGCAGGGCCAGCCACCAGGAGGGCUGCGUGCCACCCGGGCAGCUCUGCUGCUCACUGGCAGUGUCACCU

>hsa-mir-3198-1

GACUGUGCUCUCACUGUUCACCCAGCACUAGCAGUACCAGACGGUUCUGUGGAGUCCUGGGGAAUGGAGAGAGCACAGUC

>hsa-mir-3198-2

GACUCUGCUCUCACUGUUCACCCAGCACUAGCAGUACCAGAUGGUUCUGUGGAGUCCUGGGGAAUGGAGAGAGCACAGUC

>hsa-mir-3199-1

GGUGACUCCAGGGACUGCCUUAGGAGAAAGUUUCUGGAAGUUCUGACAUUCCAGAAACUUUCUCCUAAGGCAGUCCCUGGGAGUCACU

>hsa-mir-3199-2

GUGACUCCCAGGGACUGCCUUAGGAGAAAGUUUCUGGAAUGUCAGAACUUCCAGAAACUUUCUCCUAAGGCAGUCCCUGGAGUCAC

>hsa-mir-32

GGAGAUAUUGCACAUUACUAAGUUGCAUGUUGUCACGGCCUCAAUGCAAUUUAGUGUGUGUGAUAUUUUC

>hsa-mir-3200

GGUGGUCGAGGGAAUCUGAGAAGGCGCACAAGGUUUGUGUCCAAUACAGUCCACACCUUGCGCUACUCAGGUCUGCUCGUGCCCU

>hsa-mir-3202-1

UAUUAAUAUGGAAGGGAGAAGAGCUUUAAUGAUUGGAGUCAUUUUCAGAGCAUUAAAGCUCUUCUCCCUUCCAUAUUAAUG

>hsa-mir-3202-2

AUUAAUAUGGAAGGGAGAAGAGCUUUAAUGCUCUGAAAAUGACUCCAAUCAUUAAAGCUCUUCUCCCUUCCAUAUUAAU

>hsa-mir-320a

CUCCCCUCCGCCUUCUCUUCCCGGUUCUUCCCGGAGUCGGGAAAAGCUGGGUUGAGAGGGCGAAAAAGGAUG

>hsa-mir-320b-1

AUAAAUUAAUCCCUCUCUUUCUAGUUCUUCCUAGAGUGAGGAAAAGCUGGGUUGAGAGGGCAAACAAAUUAA

>hsa-mir-320b-2

GUCUCUUAGGCUUUCUCUUCCCAGAUUUCCCAAAGUUGGGAAAAGCUGGGUUGAGAGGGCAAAAGGAAAAA

>hsa-mir-320c-1

AAAAAUGAGGCCUUCUCUUCCCAGUUCUUCCCAGAGUCAGGAAAAGCUGGGUUGAGAGGGUAGAAAAAAAAU

>hsa-mir-320c-2

GCAUGACAGGCCUUCUCUUUCCAGUUCUUCCCAGAAUUGGGAAAAGCUGGGUUGAGAGGGUAAGAAAAGAAA

>hsa-mir-320d-1

AAAAUGUUGGCCUUCUCGUCCCAGUUCUUCCCAAAGUUGAGAAAAGCUGGGUUGAGAGGAUGAAAAGAAAAA

>hsa-mir-320d-2

UAUCAAUAAGCCUUCUCUUCCCAGUUCUUCUUGGAGUCAGGAAAAGCUGGGUUGAGAGGAGCAGAAAAGAAA

>hsa-mir-320e

CUCCAUGGGGCCUUCUCUUCCCAGUUCUUCCUGGAGUCGGGGAAAAGCUGGGUUGAGAAGGUGAAAAGAAAAA

>hsa-mir-323a

UUGGUACUUGGAGAGAGGUGGUCCGUGGCGCGUUCGCUUUAUUUAUGGCGCACAUUACACGGUCGACCUCUUUGCAGUAUCUAAUC

>hsa-mir-324

CUGACUAUGCCUCCCCGCAUCCCCUAGGGCAUUGGUGUAAAGCUGGAGACCCACUGCCCCAGGUGCUGCUGGGGGUUGUAGUC

>hsa-mir-326

CUCAUCUGUCUGUUGGGCUGGAGGCAGGGCCUUUGUGAAGGCGGGUGGUGCUCAGAUCGCCUCUGGGCCCUUCCUCCAGCCCCGAGGCGGAUUCA

>hsa-mir-328

UGGAGUGGGGGGGCAGGAGGGGCUCAGGGAGAAAGUGCAUACAGCCCCUGGCCCUCUCUGCCCUUCCGUCCCCUG

>hsa-mir-329-1

GGUACCUGAAGAGAGGUUUUCUGGGUUUCUGUUUCUUUAAUGAGGACGAAACACACCUGGUUAACCUCUUUUCCAGUAUC

>hsa-mir-329-2

GUGGUACCUGAAGAGAGGUUUUCUGGGUUUCUGUUUCUUUAUUGAGGACGAAACACACCUGGUUAACCUCUUUUCCAGUAUCAA

>hsa-mir-330

CUUUGGCGAUCACUGCCUCUCUGGGCCUGUGUCUUAGGCUCUGCAAGAUCAACCGAGCAAAGCACACGGCCUGCAGAGAGGCAGCGCUCUGCCC

>hsa-mir-331

GAGUUUGGUUUUGUUUGGGUUUGUUCUAGGUAUGGUCCCAGGGAUCCCAGAUCAAACCAGGCCCCUGGGCCUAUCCUAGAACCAACCUAAGCUC

>hsa-mir-335

UGUUUUGAGCGGGGGUCAAGAGCAAUAACGAAAAAUGUUUGUCAUAAACCGUUUUUCAUUAUUGCUCCUGACCUCCUCUCAUUUGCUAUAUUCA

>hsa-mir-338

UCUCCAACAAUAUCCUGGUGCUGAGUGAUGACUCAGGCGACUCCAGCAUCAGUGAUUUUGUUGAAGA

>hsa-mir-339

CGGGGCGGCCGCUCUCCCUGUCCUCCAGGAGCUCACGUGUGCCUGCCUGUGAGCGCCUCGACGACAGAGCCGGCGCCUGCCCCAGUGUCUGCGC

>hsa-mir-33a

CUGUGGUGCAUUGUAGUUGCAUUGCAUGUUCUGGUGGUACCCAUGCAAUGUUUCCACAGUGCAUCACAG

>hsa-mir-33b

GCGGGCGGCCCCGCGGUGCAUUGCUGUUGCAUUGCACGUGUGUGAGGCGGGUGCAGUGCCUCGGCAGUGCAGCCCGGAGCCGGCCCCUGGCACCAC

>hsa-mir-340

UUGUACCUGGUGUGAUUAUAAAGCAAUGAGACUGAUUGUCAUAUGUCGUUUGUGGGAUCCGUCUCAGUUACUUUAUAGCCAUACCUGGUAUCUUA

>hsa-mir-342

GAAACUGGGCUCAAGGUGAGGGGUGCUAUCUGUGAUUGAGGGACAUGGUUAAUGGAAUUGUCUCACACAGAAAUCGCACCCGUCACCUUGGCCUACUUA

>hsa-mir-345

ACCCAAACCCUAGGUCUGCUGACUCCUAGUCCAGGGCUCGUGAUGGCUGGUGGGCCCUGAACGAGGGGUCUGGAGGCCUGGGUUUGAAUAUCGACAGC

>hsa-mir-346

GGUCUCUGUGUUGGGCGUCUGUCUGCCCGCAUGCCUGCCUCUCUGUUGCUCUGAAGGAGGCAGGGGCUGGGCCUGCAGCUGCCUGGGCAGAGCGG

>hsa-mir-34a

GGCCAGCUGUGAGUGUUUCUUUGGCAGUGUCUUAGCUGGUUGUUGUGAGCAAUAGUAAGGAAGCAAUCAGCAAGUAUACUGCCCUAGAAGUGCUGCACGUUGUGGGGCCC

>hsa-mir-34b

GUGCUCGGUUUGUAGGCAGUGUCAUUAGCUGAUUGUACUGUGGUGGUUACAAUCACUAACUCCACUGCCAUCAAAACAAGGCAC

>hsa-mir-34c

AGUCUAGUUACUAGGCAGUGUAGUUAGCUGAUUGCUAAUAGUACCAAUCACUAACCACACGGCCAGGUAAAAAGAUU

>hsa-mir-3529

GGCACCAUUAGGUAGACUGGGAUUUGUUGUUGAGCGCAGUAAGACAACAACAAAAUCACUAGUCUUCCAGAUGGGGCC

>hsa-mir-3605

ACUUUAUACGUGUAAUUGUGAUGAGGAUGGAUAGCAAGGAAGCCGCUCCCACCUGACCCUCACGGCCUCCGUGUUACCUGUCCUCUAGGUGGGACGCUCG

>hsa-mir-3609

GUAACAGUAACUUUUAUUCUCAUUUUCCUUUUCUCUACCUUGUAGAGAAGCAAAGUGAUGAGUAAUACUGGCUGGAGCCC

>hsa-mir-361

GGAGCUUAUCAGAAUCUCCAGGGGUACUUUAUAAUUUCAAAAAGUCCCCCAGGUGUGAUUCUGAUUUGCUUC

>hsa-mir-3610

AAGAGCCGCGGCGUAACGGCAGCCAUCUUGUUUGUUUGAGUGAAUCGGAAAGGAGGCGCCGGCUGUGGCGGCG

>hsa-mir-3611

AGCAGGUCUAAUAAGAAUUUCUUUUUCUUCACAAUUAUGAAAGAAAAGAAAUUGUGAAGAAAGAAAUUCUUACUAGUUUUGCU

>hsa-mir-3614

GGUUCUGUCUUGGGCCACUUGGAUCUGAAGGCUGCCCCUUUGCUCUCUGGGGUAGCCUUCAGAUCUUGGUGUUUUGAAUUCUUACU

>hsa-mir-3615

GACUCUGGGACGCUCAGACGCCGCGCGGGGCGGGGAUUGGUCUGUGGUCCUCUCUCGGCUCCUCGCGGCUCGCGGCGGCCGACGGUU

>hsa-mir-3617

AGGUCAUAGAAAGACAUAGUUGCAAGAUGGGAUUAGAAACCAUAUGUCUCAUCAGCACCCUAUGUCCUUUCUCUGCCCU

>hsa-mir-3618

UAAGCUGAGUGCAUUGUGAUUUCCAAUAAUUGAGGCAGUGGUUCUAAAAGCUGUCUACAUUAAUGAAAAGAGCAAUGUGGCCAGCUUG

>hsa-mir-3619

ACGGCAUCUUUGCACUCAGCAGGCAGGCUGGUGCAGCCCGUGGUGGGGGACCAUCCUGCCUGCUGUGGGGUAAGGACGGCUGU

>hsa-mir-362

CUUGAAUCCUUGGAACCUAGGUGUGAGUGCUAUUUCAGUGCAACACACCUAUUCAAGGAUUCAAA

>hsa-mir-3620

GUGAGGUGGGGGCCAGCAGGGAGUGGGCUGGGCUGGGCUGGGCCAAGGUACAAGGCCUCACCCUGCAUCCCGCACCCAG

>hsa-mir-363

UGUUGUCGGGUGGAUCACGAUGCAAUUUUGAUGAGUAUCAUAGGAGAAAAAUUGCACGGUAUCCAUCUGUAAACC

>hsa-mir-3648-1

CGCGACUGCGGCGGCGGUGGUGGGGGGAGCCGCGGGGAUCGCCGAGGGCCGGUCGGCCGCCCCGGGUGCCGCGCGGUGCCGCCGGCGGCGGUGAGGCCCCGCGCGUGUGUCCCGGCUGCGGUCGGCCGCGCUCGAGGGGUCCCCGUGGCGUCCCCUUCCCCGCCGGCCGCCUUUCUCGCG

>hsa-mir-3648-2

CGCGACUGCGGCGGCGGUGGUGGGGGGAGCCGCGGGGAUCGCCGAGGGCCGGUCGGCCGCCCCGGGUGCCGCGCGGUGCCGCCGGCGGCGGUGAGGCCCCGCGCGUGUGUCCCGGCUGCGGUCGGCCGCGCUCGAGGGGUCCCCGUGGCGUCCCCUUCCCCGCCGGCCGCCUUUCUCGCG

>hsa-mir-3651

GAUUCGAUGGGCCAUAGCAAUCCUGUGAUUUAUGCAUGGAGGCUGCUUCUCCUCAGCAGCUGCCAUAGCCCGGUCGCUGGUACAUGAUUC

>hsa-mir-3652

CGGCUGGAGGUGUGAGGAUCCGAACCCAGGGGUGGGGGGUGGAGGCGGCUCCUGCGAUCGAAGGGGACUUGAGACUCACCGGCCGCACGCCAUGAGGGCCCUGUGGGUGCUGGGCCUCUGCUGCGUCCUGC

>hsa-mir-3654

UUCAUGAGCUGCAAUCUCAUCACUGGAAUGUUCCAGCGACUGGACAAGCUGAGGAA

>hsa-mir-3655

GCUUGUCGCUGCGGUGUUGCUGUUGGAGACUCGAUUGUUGGUGACAGCGAAAGAACGAUAACAAAAUGCCGGAGCGAGAUAGU

>hsa-mir-3657

UGUGUCCCAUAAUUAAAUAAUGAAAUCUGAAAUCACCAAUAAUGGGACACUAAUGUGAUUAAUGUUGUUGUGUCCCAUUAUUGGUGAUUUCAGAUUUCAUAUAUGAUUAAGGACAUA

>hsa-mir-365a

ACCGCAGGGAAAAUGAGGGACUUUUGGGGGCAGAUGUGUUUCCAUUCCACUAUCAUAAUGCCCCUAAAAAUCCUUAUUGCUCUUGCA

>hsa-mir-365b

AGAGUGUUCAAGGACAGCAAGAAAAAUGAGGGACUUUCAGGGGCAGCUGUGUUUUCUGACUCAGUCAUAAUGCCCCUAAAAAUCCUUAUUGUUCUUGCAGUGUGCAUCGGG

>hsa-mir-3661

CACCUUCUCGCAGAGGCUCUUGACCUGGGACUCGGACAGCUGCUUGCACUCGUUCAGCUGCUCGAUCCACUGGUCCAGCUCCUUGGUGAACACCUU

>hsa-mir-3662

UGUGUUUUCCUCAACGCUCACAGUUACACUUCUUACUCUCAAUCCAUUCAUAUUGAAAAUGAUGAGUAGUGACUGAUGAAGCACAAAUCAGCCAA

>hsa-mir-3667

UGAGGAUGAAAGACCCAUUGAGGAGAAGGUUCUGCUGGCUGAGAACCUUCCUCUCCAUGGGUCUUUCAUCCUCA

>hsa-mir-367

CCAUUACUGUUGCUAAUAUGCAACUCUGUUGAAUAUAAAUUGGAAUUGCACUUUAGCAAUGGUGAUGG

>hsa-mir-3672

UCUUUGUGAUUACCAUGAGACUCAUGUAAAACAUCUUAGACUAUUACAAGAUGUUUUAUGAGUCUCAUGAUAAUCACAAAGA

>hsa-mir-3677

GGCAGUGGCCAGAGCCCUGCAGUGCUGGGCAUGGGCUUCUCGUGGGCUCUGGCCACGGCC

>hsa-mir-3678

GAAUCCGGUCCGUACAAACUCUGCUGUGUUGAAUGAUUGGUGAGUUUGUUUGCUCAUUGAUUGAAUCACUGCAGAGUUUGUACGGACCGGAUUC

>hsa-mir-3679

CGUGGUGAGGAUAUGGCAGGGAAGGGGAGUUUCCCUCUAUUCCCUUCCCCCCAGUAAUCUUCAUCAUG

>hsa-mir-3680-1

AAAUUUAAGGAGGGACUCACUCACAGGAUUGUGCAAAUGCAAAGUUGGCUUUUGCAUGACCCUGGGAGUAGGUGCCUCCUUAAAUUU

>hsa-mir-3680-2

AAAUUUAAGGAGGGACUCACUCACAGGAUUGUGCAAAUGCAAAGUUGGCUUUUGCAUGACCCUGGGAGUAGGUGCCUCCUUAAAUUU

>hsa-mir-3681

ACUUCCAGUAGUGGAUGAUGCACUCUGUGCAGGGCCAACUGUGCACACAGUGCUUCAUCCACUACUGGAAGU

>hsa-mir-3682

UAAGUUAUAUAUGUCUACUUCUACCUGUGUUAUCAUAAUAAAGGUGUCAUGAUGAUACAGGUGGAGGUAGAAAUAUAUAACUUA

>hsa-mir-3684

AAUCUAAAGGACCUGUACUAGGUUUAACAUGUUGAGCAUUACUCAUGUUAGACCUAGUACACGUCCUUUAGAUU

>hsa-mir-3685

GUACAUUUCCUACCCUACCUGAAGACUUGAGAUUAUAGUCUUUGGGGGGAUGGGCAAAGUAC

>hsa-mir-3686

CUCACCUCAUUCAUUUACCUUCUCUUACAGAUCACUUUUCUGCACUGGACAGUGAUCUGUAAGAGAAAGUAAAUGAAAGAGGUGAG

>hsa-mir-3688-1

UCUUCACUUUCAAGAGUGGCAAAGUCUUUCCAUAUGUAUGUAUGUAUGUCUGUUACACAUAUGGAAAGACUUUGCCACUCUUUAAAGUGAAGA

>hsa-mir-3688-2

UCACUUUAAAGAGUGGCAAAGUCUUUCCAUAUGUGUAACAGACAUACAUACAUACAUAUGGAAAGACUUUGCCACUCUUGAAAGUGA

>hsa-mir-369

UUGAAGGGAGAUCGACCGUGUUAUAUUCGCUUUAUUGACUUCGAAUAAUACAUGGUUGAUCUUUUCUCAG

>hsa-mir-3690-1

CCCAUCUCCACCUGGACCCAGCGUAGACAAAGAGGUGUUUCUACUCCAUAUCUACCUGGACCCAGUGUAGAUGGG

>hsa-mir-3690-2

CCCAUCUCCACCUGGACCCAGCGUAGACAAAGAGGUGUUUCUACUCCAUAUCUACCUGGACCCAGUGUAGAUGGG

>hsa-mir-3691

UUGAGGCACUGGGUAGUGGAUGAUGGAGACUCGGUACCCACUGCUGAGGGUGGGGACCAAGUCUGCGUCAUCCUCUCCUCAGUGCCUCAA

>hsa-mir-3692

CCAUUCCUGCUGGUCAGGAGUGGAUACUGGAGCAAUAGAUACAGUUCCACACUGACACUGCAGAAGUGG

>hsa-mir-370

AGACAGAGAAGCCAGGUCACGUCUCUGCAGUUACACAGCUCACGAGUGCCUGCUGGGGUGGAACCUGGUCUGUCU

>hsa-mir-371a

GUGGCACUCAAACUGUGGGGGCACUUUCUGCUCUCUGGUGAAAGUGCCGCCAUCUUUUGAGUGUUAC

>hsa-mir-371b

GGUAACACUCAAAAGAUGGCGGCACUUUCACCAGAGAGCAGAAAGUGCCCCCACAGUUUGAGUGCC

>hsa-mir-372

GUGGGCCUCAAAUGUGGAGCACUAUUCUGAUGUCCAAGUGGAAAGUGCUGCGACAUUUGAGCGUCAC

>hsa-mir-373

GGGAUACUCAAAAUGGGGGCGCUUUCCUUUUUGUCUGUACUGGGAAGUGCUUCGAUUUUGGGGUGUCCC

>hsa-mir-374a

UACAUCGGCCAUUAUAAUACAACCUGAUAAGUGUUAUAGCACUUAUCAGAUUGUAUUGUAAUUGUCUGUGUA

>hsa-mir-374b

ACUCGGAUGGAUAUAAUACAACCUGCUAAGUGUCCUAGCACUUAGCAGGUUGUAUUAUCAUUGUCCGUGUCU

>hsa-mir-374c

ACACGGACAAUGAUAAUACAACCUGCUAAGUGCUAGGACACUUAGCAGGUUGUAUUAUAUCCAUCCGAGU

>hsa-mir-375

CCCCGCGACGAGCCCCUCGCACAAACCGGACCUGAGCGUUUUGUUCGUUCGGCUCGCGUGAGGC

>hsa-mir-376a-1

UAAAAGGUAGAUUCUCCUUCUAUGAGUACAUUAUUUAUGAUUAAUCAUAGAGGAAAAUCCACGUUUUC

>hsa-mir-376a-2

GGUAUUUAAAAGGUAGAUUUUCCUUCUAUGGUUACGUGUUUGAUGGUUAAUCAUAGAGGAAAAUCCACGUUUUCAGUAUC

>hsa-mir-376b

CAGUCCUUCUUUGGUAUUUAAAACGUGGAUAUUCCUUCUAUGUUUACGUGAUUCCUGGUUAAUCAUAGAGGAAAAUCCAUGUUUUCAGUAUCAAAUGCUG

>hsa-mir-376c

AAAAGGUGGAUAUUCCUUCUAUGUUUAUGUUAUUUAUGGUUAAACAUAGAGGAAAUUCCACGUUUU

>hsa-mir-377

UUGAGCAGAGGUUGCCCUUGGUGAAUUCGCUUUAUUUAUGUUGAAUCACACAAAGGCAACUUUUGUUUG

>hsa-mir-378a

AGGGCUCCUGACUCCAGGUCCUGUGUGUUACCUAGAAAUAGCACUGGACUUGGAGUCAGAAGGCCU

>hsa-mir-378c

GGAGGCCAUCACUGGACUUGGAGUCAGAAGAGUGGAGUCGGGUCAGACUUCAACUCUGACUUUGAAGGUGGUGAGUGCCUC

>hsa-mir-378d-1

ACUGUUUCUGUCCUUGUUCUUGUUGUUAUUACUGGACUUGGAGUCAGAAACAGG

>hsa-mir-378d-2

GAAUGGUUACAAGGAGAGAACACUGGACUUGGAGUCAGAAAACUUUCAUCCAAGUCAUUCCCUGCUCUAAGUCCCAUUUCUGUUCCAUGAGAUUGUUU

>hsa-mir-378e

CUGACUCCAGUGUCCAGGCCAGGGGCAGACAGUGGACAGAGAACAGUGCCCAAGACCACUGGACUUGGAGUCAGGACAU

>hsa-mir-378f

GUCAGGUCCUGGACUCCCAUAGUUUUCAGGCUGCUAAACAACAGAACGAGCACUGGACUUGGAGCCAGAAGUCUUGGG

>hsa-mir-378g

CACUGGGCUUGGAGUCAGAAGACCUGGCUCCAGCCCAGCUC

>hsa-mir-378h

ACAGGAACACUGGACUUGGUGUCAGAUGGGAUGAGCCCUGGCUCUGUUUCCUAGCAGCAAUCUGAUCUUGAGCUAGUCACUGG

>hsa-mir-378i

GGGAGCACUGGACUAGGAGUCAGAAGGUGGAGUUCUGGGUGCUGUUUUCCCACUCUUGGGCCCUGGGCAUGUUCUG

>hsa-mir-379

AGAGAUGGUAGACUAUGGAACGUAGGCGUUAUGAUUUCUGACCUAUGUAACAUGGUCCACUAACUCU

>hsa-mir-380

AAGAUGGUUGACCAUAGAACAUGCGCUAUCUCUGUGUCGUAUGUAAUAUGGUCCACAUCUU

>hsa-mir-381

UACUUAAAGCGAGGUUGCCCUUUGUAUAUUCGGUUUAUUGACAUGGAAUAUACAAGGGCAAGCUCUCUGUGAGUA

>hsa-mir-382

UACUUGAAGAGAAGUUGUUCGUGGUGGAUUCGCUUUACUUAUGACGAAUCAUUCACGGACAACACUUUUUUCAGUA

>hsa-mir-383

CUCCUCAGAUCAGAAGGUGAUUGUGGCUUUGGGUGGAUAUUAAUCAGCCACAGCACUGCCUGGUCAGAAAGAG

>hsa-mir-3907

GGGUUGGAAAGCUGUAGGUGUGGAGGGGCAUGGAUACGGGGGCCAUGAGGGUGGGGUCCAGGCUGGACCAGGCCUGCCCUGAGUCCCCCAGCAGGUGCUCCAGGCUGGCUCACACCCUCUGCCUCUCUCUCUUCCUUCCUGGCCCCAACCC

>hsa-mir-3908

GCCUGAGCAAUGUAGGUAGACUGUUUCUAAAAAAAUAAAAAGUUAAAAAAAUUUAUGUUAACGUGUAAUGUGUUUACUAAUUUUUUUUUUUUUUUUUGGAGACAGAGUCUCCCUCUGUCGCCAGGC

>hsa-mir-3909

GGUAUGCUGUUGCGCUGUCCUUCCUCUGGGGAGCAGGCUCCGGGGGACAGGGAAAAGCACACAAGGAACUUGUCCUCUAGGGCCUGCAGUCUCAUGGGAGAGUGACAUGCACCAGGACC

>hsa-mir-3910-1

CUUUUGCUGUCAGUUUUUCUGUUGCUUGUCUUGGUUUUAUGCCUUUUAUAUCAAGGCACAUAAAAGGCAUAAAACCAAGACAAGCAACAAAAAAAGGAUUGAUCACAGAAG

>hsa-mir-3910-2

UUUUUUUGUUGCUUGUCUUGGUUUUAUGCCUUUUAUGUGCCUUGAUAUAAAAGGCAUAAAACCAAGACAAGCAACAGAAAAA

>hsa-mir-3911

GGGUGAGGAUGUGUGUGGAUCCUGGAGGAGGCAGAGAAGACAGUGAGCUUGCCAGUUCUGGUUUCCAACACUUCCUUUCCUGCGCUUCUCGAUUCCCAGAUCUGCACCC

>hsa-mir-3912

AGAGAGGAAUGAACAGUUAAAUUAUAACAUGUCCAUAUUAUGGGUUAGUUGUGGACACAUACUAACGCAUAAUAUGGACAUGUUAUAAUUUAACUGUUCCUUUCU

>hsa-mir-3913-1

UUGUUUAUAAUAAACUGAAAUAUUUGGGACUGAUCUUGAUGUCUGCCAAAACCUUGGCAGACAUCAAGAUCAGUCCCAAAUAUUUCAGUUUAUUAUAGACAG

>hsa-mir-3913-2

UGUCUAUAAUAAACUGAAAUAUUUGGGACUGAUCUUGAUGUCUGCCAAGGUUUUGGCAGACAUCAAGAUCAGUCCCAAAUAUUUCAGUUUAUUAUAAACA

>hsa-mir-3916

AUCCCAGAGAAGAAGGAAGAAGAGGAAGAAAUGGCUGGUUCUCAGGUGAAUGUGUCUGGGUUCAGGGGAUGUGUCUCCUCUUUUCUUCUGGGAU

>hsa-mir-3917

GGCGCUUUUGUGCGCGCCCGGGUCUGUUGGUGCUCAGAGUGUGGUCAGGCGGCUCGGACUGAGCAGGUGGGUGCGGGGCUCGGAGGAGGCGGC

>hsa-mir-3918

AGGCGGUUAAGCCAUGGGACAGGGCCGCAGAUGGAGACUGCUCAAGGUCAAAGGGGUCUCCAGCUGGGACCCUGCACCUGGUUCGUAGCCCCU

>hsa-mir-3919

CCUGAGCACCAUUUACUGAGUCCUUUGUUCUCUACUAGUUUGUAGUAGUUCGUAGCAGAGAACAAAGGACUCAGUAAAUGGUGCUCAGG

>hsa-mir-3922

GGAAGAGUCAAGUCAAGGCCAGAGGUCCCACAGCAGGGCUGGAAAGCACACCUGUGGGACUUCUGGCCUUGACUUGACUCUUUC

>hsa-mir-3924

UAAAUGAAAAAGUAGUAGUCAAAUAUGCAGAUCUAUGUCAUAUAUACAGAUAUGUAUAUGUGACUGCUACUUUUUUGUUUA

>hsa-mir-3925

GUGGGAAUAGCAAGAGAACUGAAAGUGGAGCCUGUCACAUCUCCAGACUCCAGUUUUAGUUCUCUUGCUAUUUCCAC

>hsa-mir-3928

GCUGAAGCUCUAAGGUUCCGCCUGCGGGCAGGAAGCGGAGGAACCUUGGAGCUUCGGC

>hsa-mir-3929

AGUGGCUCACACCAGUAAUCCCAGCACUUUGGGAGGCUGAUGUGAGUAGACCACU

>hsa-mir-3934

CACAGCCCUUCCUGUCCCCAGUUUUCAGGUGUGGAAACUGAGGCAGGAGGCAGUGAAGUAACUUGCUCAGGUUGCACAGCUGGGAAGUGGAGCAGGGAUUUGAAUCC

>hsa-mir-3936

AUGAUUCAGAGCAUCUGUCCAGUGUCUGCUGUAGAUCCCUCAAAUCCGUGUUUGGACGCUUCUGGUAAGGGGUGUAUGGCAGAUGCACCCGACAGAUGCACUUGGCAGCA

>hsa-mir-3937

AGAAGAAUGCCCAACCAGCCCUCAGUUGCUACAGUUCCCUGUUGUUUCAGCUCGACAACAACAGGCGGCUGUAGCAAUGGGGGGCUGGAUGGGCAUCUCAAUGUGC

>hsa-mir-3939

CUGGCUUCCAAAGGCCUCUGUGUGUUCCUGUAUGUGGGCGUGCACGUACCUGUCACAUGUGUACGCGCAGACCACAGGAUGUCCACACUGGCUUCCAAACACAUCU

>hsa-mir-3940

GCUUAUCGAGGAAAAGAUCGAGGUGGGUUGGGGCGGGCUCUGGGGAUUUGGUCUCACAGCCCGGAUCCCAGCCCACUUACCUUGGUUACUCUCCUUCCUUCU

>hsa-mir-3942

UCUUCAGUAUGACACCUCAAAGAAGCAAUACUGUUACCUGAAAUAGGCUGCGAAGAUAACAGUAUUUCAGAUAACAGUAUUACAUCUUUGAAGUGUCAUAUUCACUGAC

>hsa-mir-3943

CACACAGACGGCAGCUGCGGCCUAGCCCCCAGGCUUCACUUGGCGUGGACAACUUGCUAAGUAAAGUGGGGGGUGGGCCACGGCUGGCUCCUACCUGGAC

>hsa-mir-3944

UCCACCCAGCAGGCGCAGGUCCUGUGCAGCAGGCCAACCGAGAAGCGCCUGCGUCUCCCAUUUUCGGGCUGGCCUGCUGCUCCGGACCUGUGCCUGAUCUUAAUGCUG

>hsa-mir-409

UGGUACUCGGGGAGAGGUUACCCGAGCAACUUUGCAUCUGGACGACGAAUGUUGCUCGGUGAACCCCUUUUCGGUAUCA

>hsa-mir-410

GGUACCUGAGAAGAGGUUGUCUGUGAUGAGUUCGCUUUUAUUAAUGACGAAUAUAACACAGAUGGCCUGUUUUCAGUACC

>hsa-mir-411

UGGUACUUGGAGAGAUAGUAGACCGUAUAGCGUACGCUUUAUCUGUGACGUAUGUAACACGGUCCACUAACCCUCAGUAUCAAAUCCAUCCCCGAG

>hsa-mir-412

CUGGGGUACGGGGAUGGAUGGUCGACCAGUUGGAAAGUAAUUGUUUCUAAUGUACUUCACCUGGUCCACUAGCCGUCCGUAUCCGCUGCAG

>hsa-mir-421

GCACAUUGUAGGCCUCAUUAAAUGUUUGUUGAAUGAAAAAAUGAAUCAUCAACAGACAUUAAUUGGGCGCCUGCUCUGUGAUCUC

>hsa-mir-422a

GAGAGAAGCACUGGACUUAGGGUCAGAAGGCCUGAGUCUCUCUGCUGCAGAUGGGCUCUCUGUCCCUGAGCCAAGCUUUGUCCUCCCUGG

>hsa-mir-423

AUAAAGGAAGUUAGGCUGAGGGGCAGAGAGCGAGACUUUUCUAUUUUCCAAAAGCUCGGUCUGAGGCCCCUCAGUCUUGCUUCCUAACCCGCGC

>hsa-mir-424

CGAGGGGAUACAGCAGCAAUUCAUGUUUUGAAGUGUUCUAAAUGGUUCAAAACGUGAGGCGCUGCUAUACCCCCUCGUGGGGAAGGUAGAAGGUGGGG

>hsa-mir-425

GAAAGCGCUUUGGAAUGACACGAUCACUCCCGUUGAGUGGGCACCCGAGAAGCCAUCGGGAAUGUCGUGUCCGCCCAGUGCUCUUUC

>hsa-mir-4254

CUUGGGAGGAGGGUGGGGUGGCUCCUCUGCAGUGAGUAGGUCUGCCUGGAGCUACUCCACCAUCUCCCCCAGCCCC

>hsa-mir-4286

UACUUAUGGCACCCCACUCCUGGUACCAUAGUCAUAAGUUAGGAGAUGUUAGAGCUGUGAGUACCAUGACUUAAGUGUGGUGGCUUAAACAUG

>hsa-mir-429

CGCCGGCCGAUGGGCGUCUUACCAGACAUGGUUAGACCUGGCCCUCUGUCUAAUACUGUCUGGUAAAACCGUCCAUCCGCUGC

>hsa-mir-431

UCCUGCUUGUCCUGCGAGGUGUCUUGCAGGCCGUCAUGCAGGCCACACUGACGGUAACGUUGCAGGUCGUCUUGCAGGGCUUCUCGCAAGACGACAUCCUCAUCACCAACGACG

>hsa-mir-4315-1

UGGGCUUUGCCCGCUUUCUGAGCUGGACCCUCUCUCUACCUCUGGUGCAGAACUACAGCGGAAGGAAUCUCUG

>hsa-mir-4315-2

UGGGCUUUGCCCGCUUUCUGAGCUGGACCCUCUCUCUACCUCUGGUGCAGAACUACAGCGGAAGGAAUCUCUG

>hsa-mir-432

UGACUCCUCCAGGUCUUGGAGUAGGUCAUUGGGUGGAUCCUCUAUUUCCUUACGUGGGCCACUGGAUGGCUCCUCCAUGUCUUGGAGUAGAUCA

>hsa-mir-4323

CGGGGCCCAGGCGGGCAUGUGGGGUGUCUGGAGACGCCAGGCAGCCCCACAGCCUCAGACCUCGGGCAC

>hsa-mir-4326

GCUGCUCUGCUGUUCCUCUGUCUCCCAGACUCUGGGUGGAUGGAGCAGGUCGGGGGCCA

>hsa-mir-433

CCGGGGAGAAGUACGGUGAGCCUGUCAUUAUUCAGAGAGGCUAGAUCCUCUGUGUUGAGAAGGAUCAUGAUGGGCUCCUCGGUGUUCUCCAGG

>hsa-mir-4420

CUCUUGGUAUGAACAUCUGUGUGUUCAUGUCUCUCUGUGCACAGGGGACGAGAGUCACUGAUGUCUGUAGCUGAGAC

>hsa-mir-4421

CUGGGUCUCCUUUCUGCUGAGAGUUGAACACUUGUUGGGACAACCUGUCUGUGGAAAGGAGCUACCUAC

>hsa-mir-4422

AGUUCUUCUGCAGACAAAAGCAUCAGGAAGUACCCACCAUGUACCAGUGGGCCCUUCUUGAUGCUCUUGAUUGCAGAGGAGCC

>hsa-mir-4423

AUCAUGUACUGCAGUUGCCUUUUUGUUCCCAUGCUGUUUAAGCCUAGCAUAGGCACCAAAAAGCAACAACAGUAUGUGAA

>hsa-mir-4424

CUUACAUCACACACAGAGUUAACUCAAAAUGGACUAAUUUUUCCACUAGUUAGUCCAUUUCAAGUUAACUCUGUGUGUGAUGUAGU

>hsa-mir-4425

GUGCUUUACAUGAAUGGUCCCAUUGAAUCCCAACAGCUUUGCGAAGUGUUGUUGGGAUUCAGCAGGACCAUUCGUGUAAAGUAA

>hsa-mir-4426

AGUUGGAAGAUGGACGUACUUUGUCUGACUACAAUAUUCAAAAGGAGUCUACUCUUCAUCUUG

>hsa-mir-4431

UGGUUUGCGACUCUGAAAACUAGAAGGUUUAUGACUGGGCAUUUCUCACCCAAUGCCCAAUAUUGAACUUUCUAGUUGUCAGAGUCAUUAACCC

>hsa-mir-4433a

CAUCCUCCUUACGUCCCACCCCCCACUCCUGUUUCUGGUGAAAUAUUCAAACAGGAGUGGGGGUGGGACAUAAGGAGGAUA

>hsa-mir-4433b

UGUGUUCCCUAUCCUCCUUAUGUCCCACCCCCACUCCUGUUUGAAUAUUUCACCAGAAACAGGAGUGGGGGGUGGGACGUAAGGAGGAUGGGGGAAAGAACA

>hsa-mir-4434

UCACUUUAGGAGAAGUAAAGUAGAACUUUGGUUUUCAACUUUUCCUACAGUGU

>hsa-mir-4435-1

AGGCAGCAAAUGGCCAGAGCUCACACAGAGGGAUGAGUGCACUUCACCUGCAGUGUGACUCAGCAGGCCAACAGAUGCUA

>hsa-mir-4435-2

GCAAAUGGCCAGAGCUCACACAGAGGGAUGAGUGCACUUCACCUGCAGUGUGACUCAGCAGGCCAACAGAUGCU

>hsa-mir-4437

ACUUUGUGCAUUGGGUCCACAAGGAGGGGAUGACCCUUGUGGGCUCAGGGUACAAAGGUU

>hsa-mir-4439

CCAGUGACUGAUACCUUGGAGGCAUUUUAUCUAAGAUACACACAAAGCAAAUGCCUCUAAGGUAUCAGUUUACCAGGCCA

>hsa-mir-4440

CUCUCACCAAGCAAGUGCAGUGGGGCUUGCUGGCUUGCACCGUGACUCCCUCUCACCAAGCAAGUGUCGUGGGGCUUGCUGGCUUGCACUGUGAAGAU

>hsa-mir-4442

GCGCCCUCCCUCUCUCCCCGGUGUGCAAAUGUGUGUGUGCGGUGUUAUGCCGGACAAGAGGGAGGUG

>hsa-mir-4444-1

GUGACGACUGGCCCCGCCUCUUCCUCUCGGUCCCAUAUUGAACUCGAGUUGGAAGAGGCGAGUCCGGUCUCAAA

>hsa-mir-4444-2

GUGACGACUGGCCCCGCCUCUUCCUCUCGGUCCCAUAUUGAACUCGAGUUGGAAGAGGCGAGUCCGGUCUCAAA

>hsa-mir-4445

UUCCUGCAGAUUGUUUCUUUUGCCGUGCAAGUUUAAGUUUUUGCACGGCAAAAGAAACAAUCCAGAGGGU

>hsa-mir-4449

AGCAGCCCUCGGCGGCCCGGGGGGCGGGCGGCGGUGCCCGUCCCGGGGCUGCGCGAGGCACAGGCG

>hsa-mir-4453

UGGAGAGCUUGGUCUGUAGCGGUUUCCUUCGGGGCAGGUGGGGACUGCUCCUUUGGGAGGAAGGAGGAGGCCCAGGCCGCGUCUUCAGG

>hsa-mir-4454

CCGGAUCCGAGUCACGGCACCAAAUUUCAUGCGUGUCCGUGUGAAGAGACCACCA

>hsa-mir-4457

GGAGUACUCCAGUCAAUACCGUGUGAGUUAGAAAAGCUCAAUUCACAAGGUAUUGACUGGCGUAUUCA

>hsa-mir-4458

GAGCGCACAGAGGUAGGUGUGGAAGAAAGUGAAACACUAUUUUAGGUUUUAGUUACACUCUGCUGUGGUGUGCUG

>hsa-mir-4465

CAUGUGUCCCCUGGCACGCUAUUUGAGGUUUACUAUGGAACCUCAAGUAGUCUGACCAGGGGACACAUGA

>hsa-mir-4466

ACGCGGGUGCGGGCCGGCGGGGUAGAAGCCACCCGGCCCGGCCCGGCCCGGCGA

>hsa-mir-4467

UGGUGGCGGCGGUAGUUAUGGGCUUCUCUUUCUCACCAGCAGCCCCUGGGCCGCCGCCUCCCU

>hsa-mir-4469

CCGACGCGGAGAGCGGCUCUAGGUGGGUUUGGCGGCGGCGAGGACACCGCCGCUCCCUCUAGGGUCGCUCGGAGCGUGA

>hsa-mir-4470

CGAGCCUCUUUCGGCUUUCCAGUUUGUCUCGGUCCUUUGGAACGUGGCAAACGUGGAAGCCGAGAGGGCUCU

>hsa-mir-4471

CCAAAUUUAAAACUUAAACCUCUACUAAGUUUCCAUGAAAAGAACCCAUGGGAACUUAGUAGAGGUUUAAGUUUUAAAUUUGA

>hsa-mir-4472-2

UGGUGGGGGUGGGGGGUGUUGUUUUUGUUUUUGAGACAGAGUCUUGCUCCGUCGCCCAGGCCGGAGU

>hsa-mir-4473

AAGGAACAGGGGACACUUGUAAUGGAGAACACUAAGCUAUGGACUGCUAUGGACUGCUAGUGCUCUCCGUUACAAGUAUCCCCUGUUACCU

>hsa-mir-4477a

UCCUCCUCCCAUCAAUCACAAAUGUCCUUAAUGGCAUUUAAGGAUUGCUAUUAAGGACAUUUGUGAUUCACGGGAGGAGGU

>hsa-mir-4477b

ACCUCCUCCCGUGAAUCACAAAUGUCCUUAAUAGCAAUCCUUAAAUGCCAUUAAGGACAUUUGUGAUUGAUGGGAGGAGGA

>hsa-mir-4479

GAAACCAAGUCCGAGCGUGGCUGGCGCGGGAAAGUUCGGGAACGCGCGCGGCCGUGCUCGGAGCAGCGCCA

>hsa-mir-448

GCCGGGAGGUUGAACAUCCUGCAUAGUGCUGCCAGGAAAUCCCUAUUUCAUAUAAGAGGGGGCUGGCUGGUUGCAUAUGUAGGAUGUCCCAUCUCCCAGCCCACUUCGUCA

>hsa-mir-4482

AGUGAGCAACCCAGUGGGCUAUGGAAAUGUGUGGAAGAUGGCAUUUCUAUUUCUCAGUGGGGCUCUUACC

>hsa-mir-4484

GGGUUUCCUCUGCCUUUUUUUCCAAUGAAAAUAACGAAACCUGUUAUUUCCCAUUGAGGGGGAAAAAGGCGGGAGAAGCCCCA

>hsa-mir-4485

AGAGGCACCGCCUGCCCAGUGACAUGCGUUUAACGGCCGCGGUACCCUAACUGUGCA

>hsa-mir-4487

ACUGUCCUUCAGCCAGAGCUGGCUGAAGGGCAGAAGGGAACUGUCCUUCAGCCAGAGCUGGCUGAAGGGCAGA

>hsa-mir-4488

GGUAGGGGGCGGGCUCCGGCGCUGGGACCCCACUAGGGUGGCGCCUUGGCCCCGCCCCGCCC

>hsa-mir-4498

AGGGCUGGGCUGGCAGGGCAAGUGCUGCAGAUCUUUGUCUAAGCAGCCCCUGCCUUGGAUCUCCCA

>hsa-mir-449a

CUGUGUGUGAUGAGCUGGCAGUGUAUUGUUAGCUGGUUGAAUAUGUGAAUGGCAUCGGCUAACAUGCAACUGCUGUCUUAUUGCAUAUACA

>hsa-mir-449b

UGACCUGAAUCAGGUAGGCAGUGUAUUGUUAGCUGGCUGCUUGGGUCAAGUCAGCAGCCACAACUACCCUGCCACUUGCUUCUGGAUAAAUUCUUCU

>hsa-mir-449c

GCUGGGAUGUGUCAGGUAGGCAGUGUAUUGCUAGCGGCUGUUAAUGAUUUUAACAGUUGCUAGUUGCACUCCUCUCUGUUGCAUUCAGAAGC

>hsa-mir-4501

UAUGUGACCUCGGAUGAAUCACUGAAAUAUGUCUGAGCUUCUGUUUCAUCAGAUGUCACAUUUU

>hsa-mir-4504

CUAAGAUAAUGUCCUCCAGGUUCAUCUCUGUUGUCAUUUGUGGCAUGGACCAUUUGUGACAAUAGAGAUGAACAUGGAGGAUAUUAUCUUAA

>hsa-mir-4505

GGAGGCUGGGCUGGGACGGACACCCGGCCUCCACUUUCUGUGGCAGGUACCUCCUCCAUGUCGGCCCGCCUUG

>hsa-mir-4507

UCUGGGCUGAGCCGAGCUGGGUUAAGCCGAGCUGGGUUGGGCUGGGCUGGGU

>hsa-mir-450a-1

AAACGAUACUAAACUGUUUUUGCGAUGUGUUCCUAAUAUGCACUAUAAAUAUAUUGGGAACAUUUUGCAUGUAUAGUUUUGUAUCAAUAUA

>hsa-mir-450a-2

CCAAAGAAAGAUGCUAAACUAUUUUUGCGAUGUGUUCCUAAUAUGUAAUAUAAAUGUAUUGGGGACAUUUUGCAUUCAUAGUUUUGUAUCAAUAAUAUGG

>hsa-mir-450b

GCAGAAUUAUUUUUGCAAUAUGUUCCUGAAUAUGUAAUAUAAGUGUAUUGGGAUCAUUUUGCAUCCAUAGUUUUGUAU

>hsa-mir-4511

AAAAAAAAGGGAAAGAAGAACUGUUGCAUUUGCCCUGCACUCAGUUUGCACAGGGUAAAUGCAAUAGUUCUUCUUUCCCUUUUUUUA

>hsa-mir-4512

CUCAGCCCGGGCAAUAUAGUGAGACCUCGUCUCUACAAAAAAUUGAGACAGGGCCUCACUGUAUCGCCCAGGCUGGA

>hsa-mir-4515

GCGGGAGGUGUAACAGGACUGGACUCCCGGCAGCCCCAGGGCAGGGGCGUGGGGAGCUGGUCCUAGCUCAGCGCUCCCGGA

>hsa-mir-4517

AGGUAAAUAUGAUGAAACUCACAGCUGAGGAGCUUAGCAAGUAGCUAAGGCCAGAGCUUGUGUUUGGGUGGUGUGGCUG

>hsa-mir-4518

UGGGGGAAAAGUGCUGGGAUUGAUUAGUGAUGUCUGCUGGGGAACCGGGGCUCAGGGAUGAUAACUGUGCUGAGAAGCCCCCU

>hsa-mir-4519

AACCUCAGCAGUGCGCAGGGCUGCACUGUCUCCGUCUGCGGCCUGCAGUAAGCGGGUA

>hsa-mir-451a

CUUGGGAAUGGCAAGGAAACCGUUACCAUUACUGAGUUUAGUAAUGGUAAUGGUUCUCUUGCUAUACCCAGA

>hsa-mir-451b

UGGGUAUAGCAAGAGAACCAUUACCAUUACUAAACUCAGUAAUGGUAACGGUUUCCUUGCCAUUCCCA

>hsa-mir-452

GCUAAGCACUUACAACUGUUUGCAGAGGAAACUGAGACUUUGUAACUAUGUCUCAGUCUCAUCUGCAAAGAAGUAAGUGCUUUGC

>hsa-mir-4521

UCGGCUAAGGAAGUCCUGUGCUCAGUUUUGUAGCAUCAAAACUAGGAUUUCUCUUGUUAC

>hsa-mir-4522

GCGGGCGUUGCCUGGGGGCCUCGCAGGGGGAGAUCCAGCCCAGGCUGGUUCCGCUGACUCUGCCUGUAGGCCGGUGGCGUCUUCUGG

>hsa-mir-4523

GCGGGGGACCGAGAGGGCCUCGGCUGUGUGAGGACUAGAGGCGGCCGAGGCCCGGGCCGGUUCCCCCGA

>hsa-mir-4524a

GAACGAUAGCAGCAUGAACCUGUCUCACUGCAGAAUUAUUUUGAGACAGGCUUAUGCUGCUAUCCUUCA

>hsa-mir-4524b

UAGCUGGGUGGAUGUGUUCUUUUGAAGGAUAGCAGCAUAAGCCUGUCUCAAAAUAAUUCUGCAGUGAGACAGGUUCAUGCUGCUAUCGUUCCAAAGAGGAAGGGUAAUCACUGUC

>hsa-mir-4525

GUCAGAGGGGGGAUGUGCAUGCUGGUUGGGGUGGGCUGCCUGUGGACCAAUCAGCGUGCACUUCCCCACCCUGAA

>hsa-mir-4526

UGCGGUGACAUCAGGGCCCAGUCCCUGCUGUCAUGCCCCAGGUGACGUGCUGGGCUGACAGCAGGGCUGGCCGCUAACGUCACUGUC

>hsa-mir-4527

CCAGAAGUGGUCUGCAAAGAGAUGACUGUGAAUCCAAGAUCCACAUCAGCUCUGUGCUGCCUACAUCUGA

>hsa-mir-4529

AUGACAGGCCAUCAGCAGUCCAAUGAAGACAUGAAGACCCAAUGUCUUCAUUGGACUGCUGAUGGCCCGUCACUGGGA

>hsa-mir-4536-1

AUGUGGUAGAUAUAUGCACGAUAUAUAUACUGCCCUGCUUUUAUACAUACAUACAUACAUACCUAUAUCGUGCAUAUAUCUACCACAU

>hsa-mir-4536-2

AUGUGGUAGAUAUAUGCACGAUAUAGGUAUGUAUGUAUGUAUGUAUAAAAGCAGGGCAGUAUAUAUAUCGUGCAUAUAUCUACCACAU

>hsa-mir-454

UCUGUUUAUCACCAGAUCCUAGAACCCUAUCAAUAUUGUCUCUGCUGUGUAAAUAGUUCUGAGUAGUGCAAUAUUGCUUAUAGGGUUUUGGUGUUUGGAAAGAACAAUGGGCAGG

>hsa-mir-455

UCCCUGGCGUGAGGGUAUGUGCCUUUGGACUACAUCGUGGAAGCCAGCACCAUGCAGUCCAUGGGCAUAUACACUUGCCUCAAGGCCUAUGUCAUC

>hsa-mir-4632

GAGGGCAGCGUGGGUGUGGCGGAGGCAGGCGUGACCGUUUGCCGCCCUCUCGCUGCUCUAG

>hsa-mir-4637

CCCUUACUUGGAUCUGCAAUUAGUAUUUUAAUCAUAGAUUGUAUUUAGUUAGUUUUUAAUACUAACUGCAGAUUCAAGUGAGGG

>hsa-mir-4638

GACUCGGCUGCGGUGGACAAGUCCGGCUCCAGAACCUGGACACCGCUCAGCCGGCCGCGGCAGGGGUC

>hsa-mir-4639

UUGCUAAGUAGGCUGAGAUUGAUGUCAGGUUAUCCCCAAGCAUAACCUCACUCUCACCUUGCUUUGCAG

>hsa-mir-4641

GGGGGGCAGGGGGCAGAGGGCAUCAGAGGACAGCCGCCUGGUGCCCAUGCCAUACUUUUGCCUCAG

>hsa-mir-4642

CACAACUGCAUGGCAUCGUCCCCUGGUGGCUGUGGCCUAGGGCAAGCCACAAAGCCACUCAGUGAUGAUGCCAGCAGUUGUG

>hsa-mir-4643

GUGUGCCCUAGCAUUUAUAAUCAUGUGUUCAUUCACAUGAUCAUAAGUGGACACAUGACCAUAAAUGCUAAAGCACAC

>hsa-mir-4644

GCGGCGGUGCUCUGCCUCUUUCUCCAUCCACCCUGGUCCAGGUCCACAGCAGUGGAGAGAGAAAAGAGACAGAAGGAUGGCCGU

>hsa-mir-4645

UGAUAGGGAAACCAGGCAAGAAAUAUUGUCUCCUCAAGUUGCGACGAGACAGUAGUUCUUGCCUGGUUUCUCUAUCA

>hsa-mir-4646

ACUGGGAAGAGGAGCUGAGGGACAUUGCGGAGAGGGUCUCACAUUGUCCCUCUCCCUUCCCAG

>hsa-mir-4647

CCAGGAGGGUGAAGAUGGUGCUGUGCUGAGGAAAGGGGAUGCAGAGCCCUGCCCAGCACCACCACCUCCUAUGCUCCUGG

>hsa-mir-4648

UGUGGGACUGCAAAUGGGAGCUCAGCACCUGCCUGCCACCCACGCAGACCAGCCCCUGCUCUGUUCCCACAG

>hsa-mir-4651

CGGCGACGGCGGGGUGGGUGAGGUCGGGCCCCAAGACUCGGGGUUUGCCGGGCGCCUCAGUUCACCGCGGCCG

>hsa-mir-4654

CUGGCUGGUUGUGGGAUCUGGAGGCAUCUGGGGUUGGAAUGUGACCCCAGUCUCCUUUUCCCUCAUCAUCUGCCAG

>hsa-mir-4657

AAUGUGGAAGUGGUCUGAGGCAUAUAGAGUAUAUGCCAAGAACACUACCAUAU

>hsa-mir-4658

GCUGCCCUUCACUCAGAGCAUCUACACCCACUACCGGUGAGUGUGGAUCCUGGAGGAAUCGUGGC

>hsa-mir-4659a

GAAACUGCUGAAGCUGCCAUGUCUAAGAAGAAAACUUUGGAGAAAAAUUUUCUUCUUAGACAUGGCAACGUCAACAGUUUC

>hsa-mir-4659b

CUGUUGACGUUGCCAUGUCUAAGAAGAAAAUUUUUCUCCAAAGUUUUCUUCUUAGACAUGGCAGCUUCAGCAG

>hsa-mir-466

GUGUGUGUAUAUGUGUGUUGCAUGUGUGUAUAUGUGUGUAUAUAUGUACACAUACACAUACACGCAACACACAUAUAUACAUGC

>hsa-mir-4660

ACUCCUUCUGCAGCUCUGGUGGAAAAUGGAGAAGACUUUUCCUUUCCUCCAUCUCCCCCAGGGCCUGGUGGAGU

>hsa-mir-4661

UUUACUCUGAACUAGCUCUGUGGAUCCUGACAGACAGCCUGAUAGACAGGAUCCACAGAGCUAGUCCAGAGUAAA

>hsa-mir-4662a

UCUAUUUAGCCAAUUGUCCAUCUUUAGCUAUUCUGAAUGCCUAAAGAUAGACAAUUGGCUAAAUAGA

>hsa-mir-4662b

CACAAUUUCUAUUUAGCCAAUUGUCUAUCUUUAGGCAUUCAGAAUAGCUAAAGAUGGACAAUUGGCUAAAUAGACACUGUG

>hsa-mir-4664

GUUGGGGGCUGGGGUGCCCACUCCGCAAGUUAUCACUGAGCGACUUCCGGUCUGUGAGCCCCGUCCUCCGC

>hsa-mir-4665

CUCGAGGUGCUGGGGGACGCGUGAGCGCGAGCCGCUUCCUCACGGCUCGGCCGCGGCGCGUAGCCCCCGCCACAUCGGG

>hsa-mir-4666a

AUCACUUAAAUACAUGUCAGAUUGUAUGCCUACAAAAUCCCUCCAGACUGGCAUACAAUCUGACAUGUAUUUAAGAGAU

>hsa-mir-4667

UGACUGGGGAGCAGAAGGAGAACCCAAGAAAAGCUGACUUGGAGGUCCCUCCUUCUGUCCCCACAG

>hsa-mir-4669

GCCUCCCUUCACUUCCUGGCCAUCCAGGCAUCUGUGUCUGUGUCCGGGAAGUGGAGGAGGGC

>hsa-mir-4670

CUCUAGGAAGCGACCAUGAUGUAACUUCACAGACUCUCCAAAAGUCUGAAGUUACAUCAUGGUCGCUUCCUAGAG

>hsa-mir-4671

UAUUUUAAGACCGAAGACUGUGCGCUAAUCUCUUAGCACUGAAGAUUAGUGCAUAGUCUUUGGUCUCAAAAUA

>hsa-mir-4672

GGCUGCUUCUCGCCUCUGUCCAGCUGUGUGGCCUUGGACAAGCCUCUUGGUUACACAGCUGGACAGAGGCACGAAACAGCC

>hsa-mir-4673

GUCCAGGCAGGAGCCGGACUGGACCUCAGGGAAGAGGCUGACCCGGCCCCUCUUGCGGC

>hsa-mir-4676

UGAAUGAAAGAGCCAGUGGUGAGACAGUGAGUUGAUUACUUCUCACUGUUUCACCACUGGCUCUUUGGUUCA

>hsa-mir-4677

GCAAAGCAGCAAUUGUUCUUUGGUCUUUCAGCCAUGACCUGACCUUCUGUCUGUGAGACCAAAGAACUACUUUGCUUGGC

>hsa-mir-4679-1

GUCUUUUUUCUGUGAUAGAGAUUCUUUGCUUUGUUAGAAACAAAAAGCAAAGAAUCUCUAUCACAGAAAAAAGAU

>hsa-mir-4679-2

UAUCUUUUUUCUGUGAUAGAGAUUCUUUGCUUUUUGUUUCUAACAAAGCAAAGAAUCUCUAUCACAGAAAAAAGACG

>hsa-mir-4680

UAUAAGAACUCUUGCAGUCUUAGAUGUUAUAAAAAUAUAUAUCUGAAUUGUAAGAGUUGUUAGCAC

>hsa-mir-4683

GACACGCAAGACGAGGCGGGCCUGGAGGUGCACCAGUUCUGGCCGCUGGUGGAGAUCCAGUGCUCGCCCGAUCUCAAGUUC

>hsa-mir-4684

GCACCAGGGGUACCUCUCUACUGACUUGCAACAUACAUUUGUCUUGGUGUGUUGCAAGUCGGUGGAGACGUACCCUUGGUGC

>hsa-mir-4685

UAGCCCAGGGCUUGGAGUGGGGCAAGGUUGUUGGUGAUAUGGCUUCCUCUCCCUUCCUGCCCUGGCUAG

>hsa-mir-4687

ACCUGAGGAGCCAGCCCUCCUCCCGCACCCAAACUUGGAGCACUUGACCUUUGGCUGUUGGAGGGGGCAGGCUCGCGGGU

>hsa-mir-4688

GUCUACUCCCAGGGUGCCAAGCUGUUUCGUGUUCCCUCCCUAGGGGAUCCCAGGUAGGGGCAGCAGAGGACCUGGGCCUGGAC

>hsa-mir-4689

GGUUUCUCCUUGAGGAGACAUGGUGGGGGCCGGUCAGGCAGCCCAUGCCAUGUGUCCUCAUGGAGAGGCC

>hsa-mir-4690

GAGCAGGCGAGGCUGGGCUGAACCCGUGGGUGAGGAGUGCAGCCCAGCUGAGGCCUCUGC

>hsa-mir-4691

GGAGCACUCCCAGGUCCUCCAGGCCAUGAGCUGCGGCCCUGAUGUCUCUACUCCAGCCACGGACUGAGAGUGCAUAGGAGUGUCC

>hsa-mir-4695

CCUGCAGGAGGCAGUGGGCGAGCAGGCGGGGCAGCCCAAUGCCAUGGGCCUGAUCUCACCGCUGCCUCCUUCCC

>hsa-mir-4699

AGCAAUUGGAGAAGAUUGCAGAGUAAGUUCCUGAUUAAGAAAUGGAAUUUACUCUGCAAUCUUCUCCAAUUGCU

>hsa-mir-4700

UCAGUGAGGUCUGGGGAUGAGGACAGUGUGUCCUGAAAUUCACAGGACUGACUCCUCACCCCAGUGCACGAGGA

>hsa-mir-4703

UUAUGCAUAUUAGCAAUACAGUACAAAUAUAGUGUGUUUGAUUUGCACUGUAGUUGUAUUGUAUUGCCACUCUGUAUAA

>hsa-mir-4706

GCUACGGGGAGCGGGGAGGAAGUGGGCGCUGCUUCUGCGUUAUCUGGAAGGAGCAGCCCACUCCUGUCCUGGGCUCUGUGGU

>hsa-mir-4707

GGUUCCGGAGCCCCGGCGCGGGCGGGUUCUGGGGUGUAGACGCUGCUGGCCAGCCCGCCCCAGCCGAGGUUCUCGGCACC

>hsa-mir-4709

CUGCUUCAACAACAGUGACUUGCUCUCCAAUGGUAUCCAGUGAUUCGUUGAAGAGGAGGUGCUCUGUAGCAG

>hsa-mir-4711

AAAUGUGCAUCAGGCCAGAAGACAUGAGCCCUUUGGAAAGGUCUCGUGUCUUCUGGCUUGAUGCACAUUU

>hsa-mir-4712

GACAGGAUUCCAGUACAGGUCUCUCAUUUCCUUCAUGAUUAGGAAUACUACUUUGAAAUGAGAGACCUGUACUGUAUCUGUU

>hsa-mir-4713

GUCCCCAUUUUUCUCCCACUACCAGGCUCCCAUAAGGGUCGAAUGGGAUCCAGACAGUGGGAGAAAAAUGGGGAC

>hsa-mir-4715

GGGGAAUGAAAGUUGGCUGCAGUUAAGGUGGCUAAUCAGCUGAUGGUGCCACCUUAACUGCAGCCAAUUCUAAUUCCCC

>hsa-mir-4717

GGCAGUGUUUAGGCCACAGCCACCCAUGUGUAGGGGUGGCUACACAUGGGUGGCUGUGGCCUAAACACUGCC

>hsa-mir-4722

GGCAGGAGGGCUGUGCCAGGUUGGCUGGGCCAGGCCUGACCUGCCAGCACCUCCCUGCAG

>hsa-mir-4723

AGUUGGUGGGGGAGCCAUGAGAUAAGAGCACCUCCUAGAGAAUGUUGAACUAAAGGUGCCCUCUCUGGCUCCUCCCCAAAG

>hsa-mir-4724

ACGCAAAAUGAACUGAACCAGGAGUGAGCUUCGUGUACAUUAUCUAUUAGAAAAUGAAGUACCUUCUGGUUCAGCUAGUCCCUGUGCGU

>hsa-mir-4725

GUGUCUCUCUGGAGACCCUGCAGCCUUCCCACCCACCAGGGAGCUUUCCAUGGGCUGUGGGGAAGGCGUCAGUGUCGGGUGAGGGAACAC

>hsa-mir-4726

AGGGCCAGAGGAGCCUGGAGUGGUCGGGUCGACUGAACCCAGGUUCCCUCUGGCCGCA

>hsa-mir-4727

AAUCUGCCAGCUUCCACAGUGGCAGAUUUUCCCAUAGUGGGAAGCUGGCAGAUUC

>hsa-mir-4729

UCUGUUUCCUCAUUUAUCUGUUGGGAAGCUAACUGUGACCUUAGCGUCCCAGCAGAUAAAUGAGGAAACAGA

>hsa-mir-4730

CGCAGGCCUCUGGCGGAGCCCAUUCCAUGCCAGAUGCUGAGCGAUGGCUGGUGUGUGCUGCUCCACAGGCCUGGUG

>hsa-mir-4732

GAGGGAGCUGUAGAGCAGGGAGCAGGAAGCUGUGUGUGUCCAGCCCUGACCUGUCCUGUUCUGCCCCCAGCCCCUC

>hsa-mir-4733

GGUCGCUUAAAUCCCAAUGCUAGACCCGGUGGCAAUCAAGGUCUAGCCACCAGGUCUAGCAUUGGGAUUUAAGCCC

>hsa-mir-4734

CUCGGGCCCGACCGCGCCGGCCCGCACCUCCCGGCCCGGAGCUGCGGGCUGCGGUCAGGGCGAUCCCGGG

>hsa-mir-4737

CUGCACAGGAUGCGAGGAUGCUGACAGUGCCUCACAGCCGCACAGGACCGAGGAUGCUGACGGUGCCUCACAGCCACACAG

>hsa-mir-4738

GGUCGCAUUUCUCCUUCUUACCAGCGCGUUUUCAGUUUCAUAGGGAAGCCUUUCCAUGAAACUGGAGCGCCUGGAGGAGAAGGGGCC

>hsa-mir-4739

GGGAGGAAGAAGGGAGGAGGAGCGGAGGGGCCCUUGUCUUCCCAGAGCCUCUCCCUUCCUCCCCUCCCCCUCCC

>hsa-mir-4740

GCCAAGGACUGAUCCUCUCGGGCAGGGAGUCAGAGGGGACCGCCCGAGAGGAUCCGUCCCUGC

>hsa-mir-4741

CGGGCGGGGCGGGUCCGGCCGCCUCCGAGCCCGGCCGGCAGCCCCCGGCCUUAAAGCGCGGGCUGUCCGGAGGGGUCGGCUUUCCCACCG

>hsa-mir-4742

UCAGGCAAAGGGAUAUUUACAGAUACUUUUUAAAAUUUGUUUGAGUUGAGGCAGAUUAAAUAUCUGUAUUCUCCUUUGCCUGCAG

>hsa-mir-4743

GCUGGCCGGAUGGGACAGGAGGCAUGAAUGAGCCAUCUUUCCAAUGCCUUUCUGUCUUUUCUGGUCCAG

>hsa-mir-4744

GUAAUCACAUCUAAAGACUAGACUUCGCUAUGACCAGGCCAUAGUAAACAUCAUAGUAUGUCUAGUCUUUAGGUUUGAUUAC

>hsa-mir-4745

GUGAGUGGGGCUCCCGGGACGGCGCCCGCCCUGGCCCUGGCCCGGCGACGUCUCACGGUCCC

>hsa-mir-4746

GUGUCUGUGCCGGUCCCAGGAGAACCUGCAGAGGCAUCGGGUCAGCGGUGCUCCUGCGGGCCGACACUCAC

>hsa-mir-4747

AGGGAAGGAGGCUUGGUCUUAGCACGGGGUCUAAGGCCCGGGCUUUCCUCCCAG

>hsa-mir-4748

UGGCUGGCUGAGGUUUGGGGAGGAUUUGCUGGUGCUAGAGAGGAAAGCAGACCCUACCCAACCCCACGCCCUACUACAGCCA

>hsa-mir-4749

CCUGCGGGGACAGGCCAGGGCAUCUAGGCUGUGCACAGUGACGCCCCUCCUGCCCCCACAG

>hsa-mir-4750

CGCUCGGGCGGAGGUGGUUGAGUGCCGACUGGCGCCUGACCCACCCCCUCCCGCAG

>hsa-mir-4753

AUAUCUACACAAGGCCAAAGGAAGAGAACAGAUAUAUCCACAGUACACUUGGCUGUUCUCUUUCUUUAGCCUUGUGUAGAUAU

>hsa-mir-4754

ACGCGCCUGAUGCGGACCUGGGUUAGCGGAGUGAGGCCCAGUGGUCACCGCCGCCCUCCGCAGGUCCAGGUUGCCGUGCGCAUGUGCCU

>hsa-mir-4755

AGAUUCAGCUUUCCCUUCAGAGCCUGGCUUUGGCAUCUAUGAAAGCCAGGCUCUGAAGGGAAAGUUGAAUCU

>hsa-mir-4757

UUCCAGCCCGAGGCCUCUGUGACGUCACGGUGUCUGCGGGAGGAGACCAUGACGUCACAGAGGCUUCGCGCUCUGAG

>hsa-mir-4758

GGUGAGUGGGAGCCGGUGGGGCUGGAGUAAGGGCACGCCCGGGGCUGCCCCACCUGCUGACCACCCUCCCC

>hsa-mir-4761

GGACAAGGUGUGCAUGCCUGACCCGUUGUCAGACCUGGAAAAAGGGCCGGCUGUGGGCAGGGAGGGCAUGCGCACUUUGUCC

>hsa-mir-4762

CUGAUACCCCAAAUCUUGAUCAGAAGCCUUGAUCAGAAGCUAGGAAGGCUUCUGAUCAAGAUUUGUGGUGUCAAG

>hsa-mir-4763

CCUGUCCCUCCUGCCCUGCGCCUGCCCAGCCCUCCUGCUCUGGUGACUGAGGACCGCCAGGCAGGGGCUGGUGCUGGGCGGGGGGCGGCGGG

>hsa-mir-4765

UGGUGAUUUUGAACGUAGCUAUCCACCACUCAGCCUGGAAAAAGCUGAGUGAUUGAUAGCUAUGUUCAAAAUCACCA

>hsa-mir-4766

CUGAAGCUCCUUCUGAAAGAGCAGUUGGUGUUUAUUUUUUACUAAAUAGCAAUUGCUCUUUUGGAAGGAACUUGAG

>hsa-mir-4767

ACAUGGGCCCGCGGGCGCUCCUGGCCGCCGCCCGACUUCGGGGCCAGCCGGGGGCAGAGCGCGCGGGAGCCCGAGCGU

>hsa-mir-4769

GAGGAGAGGUGGGAUGGAGAGAAGGUAUGAGCUAAAAAUCCCCAAGCUCUGCCAUCCUCCCUCCCCUACUUCUCCCC

>hsa-mir-4771-1

GCUCUAGCCUAAUUUUAGAUCUGGUCUGCUUCAGUUUCACUCCAAGCAGACUUGACCUACAAUUAGCCUAGAGC

>hsa-mir-4771-2

GCUCUAGCCUAAUUUUAGAUCUGGUCUGCUUCAGUUUCACUCCAAGCAGACUUGACCUACAAUUAGCCUAGAGC

>hsa-mir-4772

GUGAUUGCCUCUGAUCAGGCAAAAUUGCAGACUGUCUUCCCAAAUAGCCUGCAACUUUGCCUGAUCAGAGGCAGUCAC

>hsa-mir-4773-1

UGCUCCCCAGCCUUUCUAUGCUCCUGUUCUGCUUUAUUUCAUCAAAGCAGAACAGGAGCAUAGAAAGGCUGGGGAGCA

>hsa-mir-4773-2

UGCUCCCCAGCCUUUCUAUGCUCCUGUUCUGCUUUGAUGAAAUAAAGCAGAACAGGAGCAUAGAAAGGCUGGGGAGCA

>hsa-mir-4774

UAUAUUGUUGUCUGGUAUGUAGUAGGUAAUAACUGACAAACAGACAAUUGCCUAACAUGUGCCAGAAAACAACAUA

>hsa-mir-4775

AUUAAGCUUUUAAUUUUUUGUUUCGGUCACUCUUGAUAGCAGACAUUGACUGAAACAAAAAAUUAAAAGCUUUAU

>hsa-mir-4777

UAGAAUAUUUCGGCAUUCUAGAUGAGAGAUAUAUAUAUACCUCAUAUGUAUAUGGUAUACCUCAUCUAGAAUGCUGUAAUAUUCUA

>hsa-mir-4779

UAAAUGUCUUACUGCUUUUACUGUUCCCUCCUAGAGUCCAUUCUUUACUCUAGGAGGGAAUAGUAAAAGCAGUAAGACAUUUA

>hsa-mir-4781

AGGUGCACGCUCUAGCGGGGAUUCCAAUAUUGGGCCAAUUCCCCCAAUGUUGGAAUCCUCGCUAGAGCGUGCACUU

>hsa-mir-4782

AUUGCCCAGUUCUGGAUAUGAAGACAAUCAAGAAAAGAUUUGGUGUUCUUGAUUGUCUUCAUAUCUAGAACUGGGCAGU

>hsa-mir-4783

GGGAAAGCGGAGGGCGCGCCCAGCUCCCGGGCUGAUUGCGCUAACAGUGGCCCCGGUGUUGGGGCGCGUCUGCCGCUGCCCC

>hsa-mir-4784

UGACUGGGCUGAGGAGAUGCUGGGACUGAGAGUGUCAUGGUGGAGCCUCCGUCCCUGCUCAUCCUCUCCGCAUGUUG

>hsa-mir-4785

GUAGGUGGGGACGCGGCGGCGCUGCUCCUCCGCUGCCGCCGGGAGAGUCGGCGACGCCGCCAGCUCCGCGCGC

>hsa-mir-4786

GGGCAUGGCCUGAGACCAGGACUGGAUGCACCACUCUCCCUGUGAUGAGGUGAAGCCAGCUCUGGUCUGGGCCAUUUCAC

>hsa-mir-4787

CGGUCCAGACGUGGCGGGGGUGGCGGCGGCAUCCCGGACGGCCUGUGAGGGAUGCGCCGCCCACUGCCCCGCGCCGCCUGACCG

>hsa-mir-4789

CAUGCUACGUAUGUAUACACCUGAUAUGUGUAUGUGUAAAUACAUAUCCACACACAUAGCAGGUGUAUAUAUAGGUAGCCUG

>hsa-mir-4791

UAAGAACUGGAUAUGAUGACUGAAAUAAGCUCCAUAUCAAUGAGAAUUUCAAUGGGAUUAUGUGCAGUCAAUGUCCAGUAAUUA

>hsa-mir-4793

UUUCUCCUCGCUGCCCGCACAUCCUGCUCCACAGGGCAGAGGGAGGCCAAGAAGACCUCUGCACUGUGAGUUGGCUGGCUGGAGGAA

>hsa-mir-4794

UUUUAACAUCUGGCUAUCUCACGAGACUGUAUGUCCUAACAGUGCUUGUAGUCUCAUGAGAUAGCCAGAUGUUAAAA

>hsa-mir-4795

UGAUAUGGAAGAAAUCCAGAAGUGGCUAAUAAUAUUGACACUAUAACAAUAAUGUCAAUAUUAUUAGCCACUUCUGGAUUUAUGAAUCA

>hsa-mir-4796

UAAAUUUGUGUCUAUACUCUGUCACUUUACUUUUGGCCUCAAGUCAUUGCAGUAAAGUGGCAGAGUAUAGACACAAAUUUA

>hsa-mir-4797

GACUCAGAAGACAGAGUGCCACUUACUGAAAGGUUUUUUCUCUCAGUAAGUGGCACUCUGUCUUCUGAGUU

>hsa-mir-4798

AAGUACAACUUCGGUAUACUUUGUGAAUUGGCUUUUACAAAAGACCAACUCACGAAGUAUACCGAAGUCAUACUU

>hsa-mir-4799

ACUGCUAAUAUCUAAAUGCAGCAUGCCAGUCCUGAGAUGCAGGGACUGGCAUGCUGCAUUUAUAUAUUAGCAGU

>hsa-mir-4800

GGAGAAAGGAGUGGACCGAGGAAGGAAGGAAGGCAAGGCUGUCUGUCCAUCCGUCCGUCUGUCCACCUACCUGUCAGUCC

>hsa-mir-4802

CUGACUGGCUUGUAUGGAGGUUCUAGACCAUGUUAGUGUUCAAGUCUACAUGGAUGGAAACCUUCAAGCAGGCCAAGCAG

>hsa-mir-4803

AGUGGGAUUUAACAUAAUAGUGUGGAUUGAAUCACACACACAUUUCAACCCACACUAUGAUGUUAAAUCCCAUU

>hsa-mir-4804

UCAGUGUAUUUGGACGGUAAGGUUAAGCAAGGUGCGUCGUAUCUUGCUUAACCUUGCCCUCGAAAUACACUGA

>hsa-mir-483

GAGGGGGAAGACGGGAGGAAAGAAGGGAGUGGUUCCAUCACGCCUCCUCACUCCUCUCCUCCCGUCUUCUCCUCUC

>hsa-mir-484

AGCCUCGUCAGGCUCAGUCCCCUCCCGAUAAACCCCUAAAUAGGGACUUUCCCGGGGGGUGACCCUGGCUUUUUUGGCG

>hsa-mir-485

ACUUGGAGAGAGGCUGGCCGUGAUGAAUUCGAUUCAUCAAAGCGAGUCAUACACGGCUCUCCUCUCUUUUAGU

>hsa-mir-486-1

GCAUCCUGUACUGAGCUGCCCCGAGGCCCUUCAUGCUGCCCAGCUCGGGGCAGCUCAGUACAGGAUAC

>hsa-mir-486-2

UCCUGUACUGAGCUGCCCCGAGCUGGGCAGCAUGAAGGGCCUCGGGGCAGCUCAGUACAGGAUG

>hsa-mir-487b

UUGGUACUUGGAGAGUGGUUAUCCCUGUCCUGUUCGUUUUGCUCAUGUCGAAUCGUACAGGGUCAUCCACUUUUUCAGUAUCAA

>hsa-mir-489

GUGGCAGCUUGGUGGUCGUAUGUGUGACGCCAUUUACUUGAACCUUUAGGAGUGACAUCACAUAUACGGCAGCUAAACUGCUAC

>hsa-mir-490

UGGAGGCCUUGCUGGUUUGGAAAGUUCAUUGUUCGACACCAUGGAUCUCCAGGUGGGUCAAGUUUAGAGAUGCACCAACCUGGAGGACUCCAUGCUGUUGAGCUGUUCACAAGCAGCGGACACUUCCA

>hsa-mir-491

UUGACUUAGCUGGGUAGUGGGGAACCCUUCCAUGAGGAGUAGAACACUCCUUAUGCAAGAUUCCCUUCUACCUGGCUGGGUUGG

>hsa-mir-493

CUGGCCUCCAGGGCUUUGUACAUGGUAGGCUUUCAUUCAUUCGUUUGCACAUUCGGUGAAGGUCUACUGUGUGCCAGGCCCUGUGCCAG

>hsa-mir-494

GAUACUCGAAGGAGAGGUUGUCCGUGUUGUCUUCUCUUUAUUUAUGAUGAAACAUACACGGGAAACCUCUUUUUUAGUAUC

>hsa-mir-495

UGGUACCUGAAAAGAAGUUGCCCAUGUUAUUUUCGCUUUAUAUGUGACGAAACAAACAUGGUGCACUUCUUUUUCGGUAUCA

>hsa-mir-497

CCACCCCGGUCCUGCUCCCGCCCCAGCAGCACACUGUGGUUUGUACGGCACUGUGGCCACGUCCAAACCACACUGUGGUGUUAGAGCGAGGGUGGGGGAGGCACCGCCGAGG

>hsa-mir-4999

AUAGAAAAUAAAACACAUACUGCUGUAUUGUCAGGUAGUGAUAGGAUUUAUCACUACCUGACAAUACAGUAUGUGUUUGUUUUAUAUAUUU

>hsa-mir-499a

GCCCUGUCCCCUGUGCCUUGGGCGGGCGGCUGUUAAGACUUGCAGUGAUGUUUAACUCCUCUCCACGUGAACAUCACAGCAAGUCUGUGCUGCUUCCCGUCCCUACGCUGCCUGGGCAGGGU

>hsa-mir-499b

GGAAGCAGCACAGACUUGCUGUGAUGUUCACGUGGAGAGGAGUUAAACAUCACUGCAAGUCUUAACAGCCGCC

>hsa-mir-5000

CUGAAGAGUAGAGUGUGUGGUCCCAGUUCAGAAGUGUUCCUGAGUAACUUGUGCUUAUAACUCAGGACACUUCUGAACUUGGACCAUACAGGUCUCCCUGCUU

>hsa-mir-5001

AGCUCAGGGCGGCUGCGCAGAGGGCUGGACUCAGCGGCGGAGCUGGCUGCUGGCCUCAGUUCUGCCUCUGUCCAGGUCCUUGUGACCCGCCCGCUCUCCU

>hsa-mir-5002

UCUUCCUCUCUGUCCUCUGGAAUUUGGUUUCUGAGGCACUUAGUAGGUGAUAGCAUGACUGACUGCCUCACUGACCACUUCCAGAUGAGGGUUACUC

>hsa-mir-5004

GGCACUUGCUUGGGGGUUAGUGAGGACAGGGCAAAUUCACGAGAUUGGGUUGUGCAGAGGCUGACACUUGGAUUUUCCUGGGCCUCAGGACUUCCUUUCAGACAUGG

>hsa-mir-5008

GGGCUGACCCCUAGGGUCAGGUGAGGCCCUUGGGGCACAGUGGUGCCAUCUCCCCUGUGCUCCCAGGGCCUCGCCUGUCCCUUGAGGUCGGCCC

>hsa-mir-5009

GACCAGAAGUGUUUUGGAUUUUGGACUUUUUCAGAUUUGGGGAUAUUUGCAUUAUACUUAUCCUAAAUCUGAAAGUCCAAAACCUGAAAUGACCAAUAAG

>hsa-mir-500a

GCUCCCCCUCUCUAAUCCUUGCUACCUGGGUGAGAGUGCUGUCUGAAUGCAAUGCACCUGGGCAAGGAUUCUGAGAGCGAGAGC

>hsa-mir-500b

CCCCCUCUCUAAUCCUUGCUACCUGGGUGAGAGUGCUUUCUGAAUGCAGUGCACCCAGGCAAGGAUUCUGCAAGGGGGA

>hsa-mir-501

GCUCUUCCUCUCUAAUCCUUUGUCCCUGGGUGAGAGUGCUUUCUGAAUGCAAUGCACCCGGGCAAGGAUUCUGAGAGGGUGAGC

>hsa-mir-5010

GAUCCAGGGAACCCUAGAGCAGGGGGAUGGCAGAGCAAAAUUCAUGGCCUACAGCUGCCUCUUGCCAAACUGCACUGGAUUUUGUGUCUCCCAUUCCCCAGAGCUGUCUGAGGUGCUUUG

>hsa-mir-502

UGCUCCCCCUCUCUAAUCCUUGCUAUCUGGGUGCUAGUGCUGGCUCAAUGCAAUGCACCUGGGCAAGGAUUCAGAGAGGGGGAGCU

>hsa-mir-503

UGCCCUAGCAGCGGGAACAGUUCUGCAGUGAGCGAUCGGUGCUCUGGGGUAUUGUUUCCGCUGCCAGGGUA

>hsa-mir-504

GCUGCUGUUGGGAGACCCUGGUCUGCACUCUAUCUGUAUUCUUACUGAAGGGAGUGCAGGGCAGGGUUUCCCAUACAGAGGGC

>hsa-mir-5047

GAAGCGCUUGCCUAGACGAGACACAGUGCAUAAAAACAACUUUUGGGGGACAGGUAUGUUUUCUUGCAGCUGCGGUUGUAAGGUCUUGGCAAGACAAGCA

>hsa-mir-505

GAUGCACCCAGUGGGGGAGCCAGGAAGUAUUGAUGUUUCUGCCAGUUUAGCGUCAACACUUGCUGGUUUCCUCUCUGGAGCAUC

>hsa-mir-506

GCCACCACCAUCAGCCAUACUAUGUGUAGUGCCUUAUUCAGGAAGGUGUUACUUAAUAGAUUAAUAUUUGUAAGGCACCCUUCUGAGUAGAGUAAUGUGCAACAUGGACAACAUUUGUGGUGGC

>hsa-mir-508

CCACCUUCAGCUGAGUGUAGUGCCCUACUCCAGAGGGCGUCACUCAUGUAAACUAAAACAUGAUUGUAGCCUUUUGGAGUAGAGUAAUACACAUCACGUAACGCAUAUUUGGUGG

>hsa-mir-5087

AGCUUUCUACGGGUUUGUAGCUUUGCUGGCAUGUUAAGUGUUGUCCUACAGUCGCAAGCAUAAGAAAGAGAAAGUA

>hsa-mir-5089

AAGGACUUCAGUGGGAUUUCUGAGUAGCAUCCUUGGAAUCUGCACUCAAGGGAUGCUACUCGGAAAUCCCACUGAAGUCCUUUU

>hsa-mir-509-1

CAUGCUGUGUGUGGUACCCUACUGCAGACAGUGGCAAUCAUGUAUAAUUAAAAAUGAUUGGUACGUCUGUGGGUAGAGUACUGCAUGACACAUG

>hsa-mir-509-2

CAUGCUGUGUGUGGUACCCUACUGCAGACAGUGGCAAUCAUGUAUAAUUAAAAAUGAUUGGUACGUCUGUGGGUAGAGUACUGCAUGACAC

>hsa-mir-509-3

GUGGUACCCUACUGCAGACGUGGCAAUCAUGUAUAAUUAAAAAUGAUUGGUACGUCUGUGGGUAGAGUACUGCAU

>hsa-mir-5090

UCUGAGGUACCCGGGGCAGAUUGGUGUAGGGUGCAAAGCCUGCCCGCCCCCUAAGCCUUCUGCCCCCAACUCCAGCCUGUCAGGA

>hsa-mir-5091

GACUGUGGCGACGGAGACGACAAGACUGUGCUGGUCGCGGGUUGUGGGGUUUAGGUCACCGGCAGGGGUCUGGAGUCCCUGGAGGUUAGGGCU

>hsa-mir-5092

AUCCCAGAUCAGAUGCCAAAGCCAGUGGGGACUGGACAACAUGAUGAGCCCAAACCAAUCCACGCUGAGCUUGGCAUCUGAUUUGGGA

>hsa-mir-5094

AAAAGAAAAAAAUCAGUGAAUGCCUUGAACCUAACACACUGCCUUUUAUGUGGUAGGUACAGUGGGCUCACUGAAACAUUCAACU

>hsa-mir-5100

CCAUGAGGAGCUGGCAGUGGGAUGGCCUGGGGGUAGGAGCGUGGCUUCUGGAGCUAGACCACAUGGGUUCAGAUCCCAGCGGUGCCUCUAACUGGCCACAGGACCUUGGGCAGUCAGCU

>hsa-mir-511

CAAUAGACACCCAUCGUGUCUUUUGCUCUGCAGUCAGUAAAUAUUUUUUUGUGAAUGUGUAGCAAAAGACAGAAUGGUGGUCCAUUG

>hsa-mir-512-1

UCUCAGUCUGUGGCACUCAGCCUUGAGGGCACUUUCUGGUGCCAGAAUGAAAGUGCUGUCAUAGCUGAGGUCCAAUGACUGAGG

>hsa-mir-512-2

GGUACUUCUCAGUCUGUGGCACUCAGCCUUGAGGGCACUUUCUGGUGCCAGAAUGAAAGUGCUGUCAUAGCUGAGGUCCAAUGACUGAGGCGAGCACC

>hsa-mir-513a-1

GGGAUGCCACAUUCAGCCAUUCAGCGUACAGUGCCUUUCACAGGGAGGUGUCAUUUAUGUGAACUAAAAUAUAAAUUUCACCUUUCUGAGAAGGGUAAUGUACAGCAUGCACUGCAUAUGUGGUGUCCC

>hsa-mir-513a-2

GGAUGCCACAUUCAGCCAUUCAGUGUGCAGUGCCUUUCACAGGGAGGUGUCAUUUAUGUGAACUAAAAUAUAAAUUUCACCUUUCUGAGAAGGGUAAUGUACAGCAUGCACUGCAUAUGUGGUGUCC

>hsa-mir-514a-1

AACAUGUUGUCUGUGGUACCCUACUCUGGAGAGUGACAAUCAUGUAUAAUUAAAUUUGAUUGACACUUCUGUGAGUAGAGUAACGCAUGACACGUACG

>hsa-mir-514a-2

GUUGUCUGUGGUACCCUACUCUGGAGAGUGACAAUCAUGUAUAACUAAAUUUGAUUGACACUUCUGUGAGUAGAGUAACGCAUGACAC

>hsa-mir-514a-3

GUUGUCUGUGGUACCCUACUCUGGAGAGUGACAAUCAUGUAUAACUAAAUUUGAUUGACACUUCUGUGAGUAGAGUAACGCAUGACAC

>hsa-mir-515-1

UCUCAUGCAGUCAUUCUCCAAAAGAAAGCACUUUCUGUUGUCUGAAAGCAGAGUGCCUUCUUUUGGAGCGUUACUGUUUGAGA

>hsa-mir-515-2

UCUCAUGCAGUCAUUCUCCAAAAGAAAGCACUUUCUGUUGUCUGAAAGCAGAGUGCCUUCUUUUGGAGCGUUACUGUUUGAGA

>hsa-mir-516a-1

UCUCAGGCUGUGACCUUCUCGAGGAAAGAAGCACUUUCUGUUGUCUGAAAGAAAAGAAAGUGCUUCCUUUCAGAGGGUUACGGUUUGAGA

>hsa-mir-516a-2

UCUCAGGUUGUGACCUUCUCGAGGAAAGAAGCACUUUCUGUUGUCUGAAAGAAAAGAAAGUGCUUCCUUUCAGAGGGUUACGGUUUGAGA

>hsa-mir-516b-1

UCUCAGGCUGUGACCAUCUGGAGGUAAGAAGCACUUUCUGUUUUGUGAAAGAAAAGAAAGUGCUUCCUUUCAGAGGGUUACUCUUUGAGA

>hsa-mir-516b-2

UCUCAUGAUGUGACCAUCUGGAGGUAAGAAGCACUUUGUGUUUUGUGAAAGAAAGUGCUUCCUUUCAGAGGGUUACUCUUUGAGA

>hsa-mir-517a

UCUCAGGCAGUGACCCUCUAGAUGGAAGCACUGUCUGUUGUAUAAAAGAAAAGAUCGUGCAUCCCUUUAGAGUGUUACUGUUUGAGA

>hsa-mir-517b

GUGACCCUCUAGAUGGAAGCACUGUCUGUUGUCUAAGAAAAGAUCGUGCAUCCCUUUAGAGUGUUAC

>hsa-mir-517c

GAAGAUCUCAGGCAGUGACCCUCUAGAUGGAAGCACUGUCUGUUGUCUAAGAAAAGAUCGUGCAUCCUUUUAGAGUGUUACUGUUUGAGAAAAUC

>hsa-mir-5187

GACUAAGGGUGGGAUGAGGGAUUGAAGUGGAGCAGGAAUGCGCUUUUCUCCACUGAAUCCUCUUUUCCUCAGGUGG

>hsa-mir-5188

GGGAGGCAUGGAAAUUUCUCUGGUUUCAAUGGGUACGAUUAUUGUAAGCAGGAUCCAUUCAAUAAUCGGACCCAUUUAAACCGGAGAUUUUAAAAGACAGGAAUAGAAUCCCA

>hsa-mir-5189

GGCCCGCCUUUUAGGGGCCUCGCUGUCUGGGCACAGGCGGAUGGACAGGCUGGCCUCUGGAUGACCUGCCAACCGUCAGAGCCCAGACCCACGUGGCCUCAGUUGGGGACCAGG

>hsa-mir-518b

UCAUGCUGUGGCCCUCCAGAGGGAAGCGCUUUCUGUUGUCUGAAAGAAAACAAAGCGCUCCCCUUUAGAGGUUUACGGUUUGA

>hsa-mir-518d

UCCCAUGCUGUGACCCUCUAGAGGGAAGCACUUUCUGUUGUCUGAAAGAAACCAAAGCGCUUCCCUUUGGAGCGUUACGGUUUGAGA

>hsa-mir-518e

UCUCAGGCUGUGACCCUCUAGAGGGAAGCGCUUUCUGUUGGCUAAAAGAAAAGAAAGCGCUUCCCUUCAGAGUGUUAACGCUUUGAGA

>hsa-mir-518f

UCUCAUGCUGUGACCCUCUAGAGGGAAGCACUUUCUCUUGUCUAAAAGAAAAGAAAGCGCUUCUCUUUAGAGGAUUACUCUUUGAGA

>hsa-mir-5193

CCUAGGAAAGGCUGCUGGUAACUGGGAUGGGGGUUGGGGGGAGGUAAGAAGUCUCUGACUCCUCCUCUACCUCAUCCCAGUUCCAUCACCUGAAGUGGACCUCUUGGGA

>hsa-mir-5196

UCUGAGGAGACCUGGGCUGUCAGAGGCCAGGGAAGGGGACGAGGGUUGGGGAACAGGUGGUUAGCACUUCAUCCUCGUCUCCCUCCCAGGUUAGAAGGGCCCCCCUCUCUGAAGG

>hsa-mir-519a-1

CUCAGGCUGUGACACUCUAGAGGGAAGCGCUUUCUGUUGUCUGAAAGAAAGGAAAGUGCAUCCUUUUAGAGUGUUACUGUUUGAG

>hsa-mir-519a-2

UCUCAGGCUGUGUCCCUCUACAGGGAAGCGCUUUCUGUUGUCUGAAAGAAAGGAAAGUGCAUCCUUUUAGAGUGUUACUGUUUGAGA

>hsa-mir-519b

CAUGCUGUGACCCUCUAGAGGGAAGCGCUUUCUGUUGUCUGAAAGAAAAGAAAGUGCAUCCUUUUAGAGGUUUACUGUUUG

>hsa-mir-519c

UCUCAGCCUGUGACCCUCUAGAGGGAAGCGCUUUCUGUUGUCUGAAAGAAAAGAAAGUGCAUCUUUUUAGAGGAUUACAGUUUGAGA

>hsa-mir-519d

UCCCAUGCUGUGACCCUCCAAAGGGAAGCGCUUUCUGUUUGUUUUCUCUUAAACAAAGUGCCUCCCUUUAGAGUGUUACCGUUUGGGA

>hsa-mir-520a

CUCAGGCUGUGACCCUCCAGAGGGAAGUACUUUCUGUUGUCUGAGAGAAAAGAAAGUGCUUCCCUUUGGACUGUUUCGGUUUGAG

>hsa-mir-520e

UCUCCUGCUGUGACCCUCAAGAUGGAAGCAGUUUCUGUUGUCUGAAAGGAAAGAAAGUGCUUCCUUUUUGAGGGUUACUGUUUGAGA

>hsa-mir-520f

UCUCAGGCUGUGACCCUCUAAAGGGAAGCGCUUUCUGUGGUCAGAAAGAAAAGCAAGUGCUUCCUUUUAGAGGGUUACCGUUUGGGA

>hsa-mir-520g

UCCCAUGCUGUGACCCUCUAGAGGAAGCACUUUCUGUUUGUUGUCUGAGAAAAAACAAAGUGCUUCCCUUUAGAGUGUUACCGUUUGGGA

>hsa-mir-522

UCUCAGGCUGUGUCCCUCUAGAGGGAAGCGCUUUCUGUUGUCUGAAAGAAAAGAAAAUGGUUCCCUUUAGAGUGUUACGCUUUGAGA

>hsa-mir-523

UCUCAUGCUGUGACCCUCUAGAGGGAAGCGCUUUCUGUUGUCUGAAAGAAAAGAACGCGCUUCCCUAUAGAGGGUUACCCUUUGAGA

>hsa-mir-525

CUCAAGCUGUGACUCUCCAGAGGGAUGCACUUUCUCUUAUGUGAAAAAAAAGAAGGCGCUUCCCUUUAGAGCGUUACGGUUUGGG

>hsa-mir-526a-1

CUCAGGCUGUGACCCUCUAGAGGGAAGCACUUUCUGUUGCUUGAAAGAAGAGAAAGCGCUUCCUUUUAGAGGAUUACUCUUUGAG

>hsa-mir-526a-2

GUGACCCUCUAGAGGGAAGCACUUUCUGUUGAAAGAAAAGAACAUGCAUCCUUUCAGAGGGUUAC

>hsa-mir-526b

UCAGGCUGUGACCCUCUUGAGGGAAGCACUUUCUGUUGUCUGAAAGAAGAGAAAGUGCUUCCUUUUAGAGGCUUACUGUCUGA

>hsa-mir-532

CGACUUGCUUUCUCUCCUCCAUGCCUUGAGUGUAGGACCGUUGGCAUCUUAAUUACCCUCCCACACCCAAGGCUUGCAGAAGAGCGAGCCU

>hsa-mir-539

AUACUUGAGGAGAAAUUAUCCUUGGUGUGUUCGCUUUAUUUAUGAUGAAUCAUACAAGGACAAUUUCUUUUUGAGUAU

>hsa-mir-541

ACGUCAGGGAAAGGAUUCUGCUGUCGGUCCCACUCCAAAGUUCACAGAAUGGGUGGUGGGCACAGAAUCUGGACUCUGCUUGUG

>hsa-mir-542

CAGAUCUCAGACAUCUCGGGGAUCAUCAUGUCACGAGAUACCAGUGUGCACUUGUGACAGAUUGAUAACUGAAAGGUCUGGGAGCCACUCAUCUUCA

>hsa-mir-543

UACUUAAUGAGAAGUUGCCCGUGUUUUUUUCGCUUUAUUUGUGACGAAACAUUCGCGGUGCACUUCUUUUUCAGUAUC

>hsa-mir-545

CCCAGCCUGGCACAUUAGUAGGCCUCAGUAAAUGUUUAUUAGAUGAAUAAAUGAAUGACUCAUCAGCAAACAUUUAUUGUGUGCCUGCUAAAGUGAGCUCCACAGG

>hsa-mir-548a-1

UGCAGGGAGGUAUUAAGUUGGUGCAAAAGUAAUUGUGAUUUUUGCCAUUAAAAGUAACGACAAAACUGGCAAUUACUUUUGCACCAAACCUGGUAUU

>hsa-mir-548a-2

UGUGAUGUGUAUUAGGUUUGUGCAAAAGUAAUUGGGGUUUUUUGCCGUUAAAAGUAAUGGCAAAACUGGCAAUUACUUUUGCACCAAACUAAUAUAA

>hsa-mir-548a-3

CCUAGAAUGUUAUUAGGUCGGUGCAAAAGUAAUUGCGAGUUUUACCAUUACUUUCAAUGGCAAAACUGGCAAUUACUUUUGCACCAACGUAAUACUU

>hsa-mir-548ab

AUGUUGGUGCAAAAGUAAUUGUGGAUUUUGCUAUUACUUGUAUUUAUUUGUAAUGCAAAACCCGCAAUUAGUUUUGCACCAACC

>hsa-mir-548ac

GUAUUAGGUUGGUGCAAAAGUUAUUGUGGUUUUUGCUAUUUUUUUUUAAUGGCAAAAACCGGCAAUUACUUUUGCACUAACCUAGUAG

>hsa-mir-548ad

CUGUUAGGUUGGUGCAAAAGUAAUUGUGGUUUUUGAAAGUAACUUGGCGAAAACGACAAUGACUUUUGCACCAAUCUAAUAC

>hsa-mir-548ae-1

GCAGUUUUUGCCAUUAAGUUGCGGUUUUUGCCAUUAUAAUGGCAAAAACUGCAAUUACUUUCACACCUGC

>hsa-mir-548ae-2

UGUGCAAAAGUAAUUGUGGUUUUUGUCAUUUAAAAGUAAUGGCAAAAACUGCAAUUACUUUCACACC

>hsa-mir-548ag-2

UGCAAAGGUAAUUGUGGUUUCUGCCAUUGAAAGUAAAGGCAAGAACCUCAAUUACCUUUGCAGC

>hsa-mir-548ah

AGGUUGGUGCAAAAGUGAUUGCAGUGUUUGCCAAUAAAAGUAAUGACAAAAACUGCAGUUACUUUUGCACCAGCCC

>hsa-mir-548aj-1

AUUGGUGUAAAAGUAAUUGCAGGUUAUGCCAUUAAAAGUAAUGGUAAAAACUGCAAUUACUUUUACACUAAC

>hsa-mir-548aj-2

AAGGUAUUAGGUUGGUGCAAAAGUAAUUGCAGUUUUUGCUAUUACUUUUAAUGGUAAAAACUGCAAUUACUUUUACACCAACCUAAUAUUUA

>hsa-mir-548ak

GUGCAAAAGUAACUGCGGUUUUUGAGAAGUAAUUGAAAACCGCAAUUACUUUUGCAG

>hsa-mir-548al

GGUCGGUGCAAAAGUAAUUGCUGUUUUUGCCAUUAAAAAUAAUGGCAUUAAAAGUAAUGGCAAAAACGGCAAUGACUUUUGUACCAAUCUAAUAUCU

>hsa-mir-548am

AGUUGGUGCAAAAGUAAUUGCGGUUUUUGCCGUCGAAAAUAAUGGCAAAAACUGCAGUUACUUUUGUACCAAUG

>hsa-mir-548an

CAUUAGGUUGGUGCAAAAGGCAUUGUGGUUUUUGCCUAUAAAAGUAAUGGCAAAAACCGCAAUUCCUUUUGCACCAACCUAAU

>hsa-mir-548ao

AACUAUUCUUAGGUUGAUGCAGAAGUAACUACGGUUUUUGCAGUUGAAAGUAAUGGCAAAGACCGUGACUACUUUUGCAACAGCCUAAUAGUUUCU

>hsa-mir-548ap

ACCAAUUCCUAGGUUGGUGCAAAAGUAAUUGCGGUCUUUGUCAUUAAAACCAAUAACAAAAACCACAAUUACUUUUUACUGACCUAAAGAUUAAUU

>hsa-mir-548aq

GAAAGUAAUUGCUGUUUUUGCCAUUACUUUCAGUGGCAAAAACUGCAAUUACUUUUGC

>hsa-mir-548ar

AAAAGUAAUUGCAGUUUUUGCUGUUGAACGUAGUGGUAAAACUGCAGUUAUUUUUGC

>hsa-mir-548at

AAAAGUUAUUGCGGUUUUGGCUGCCAAAAGAAAUGGCCAAAACCGCAGUAACUUUUGU

>hsa-mir-548au

AAAAGUAAUUGCGGUUUUUGCUAUUGGUUUUAAUGGCAGUUACUUUUGCACCAG

>hsa-mir-548av

AAAAGUACUUGCGGAUUUGCCAUCACCUUUACCUUUAAUGGCAAAACUGCAGUUACUUUUGC

>hsa-mir-548aw

UAGGUCGGUGCAAAAGUCAUCACGGUUUUUACCAUUAAAACCGCGAUGACUUUUGCAUCAACCUA

>hsa-mir-548ax

GAUUGGUGCAGAAGUAAUUGCGGUUUUGCCAUGGAAAGUAAUGGCAAAAACCGUAAUUACUUUUGUACCAACC

>hsa-mir-548ay

AGAAGAUGCUUACUACUAGGUUGGUGCAAAAGUAAUUGUGGUUUUUGCAUUUAAAGUAAUGGCCAAAACCGCGAUUACUCUUGCACGAACCUAACGGUAACACUUCU

>hsa-mir-548az

AGAUUGUAUUAGGUUGGUGCAAAAGUGAUUGUGGUUUUUGCUGUUACUUUUAAUGGCAAAAACUGCAAUCACUUUUGCACCAACCUAAUAAAUUU

>hsa-mir-548b

CAGACUAUAUAUUUAGGUUGGCGCAAAAGUAAUUGUGGUUUUGGCCUUUAUUUUCAAUGGCAAGAACCUCAGUUGCUUUUGUGCCAACCUAAUACUU

>hsa-mir-548ba

AAAGGUAACUGUGAUUUUUGCUAUUAGAAAGUAAUGGCAAAAACUGCAAUUACUUU

>hsa-mir-548bb

UUAGAUUGGUGCAAAAGUAACUAUGGUUUUUGCCCAAAAACCAUAGUUACUUUUGCACCAAGCUAA

>hsa-mir-548bc

UCUUUUGUUACAGGUUGGUGAGAAGUAAUCGCGGUUUUUGCCAUUACUGCUCAUGGCAAAAACUGUGAUUACUUUUGCACCAACCUAAUAUAAUAUUUGGUCUUCAAUUA

>hsa-mir-548c

CAUUGGCAUCUAUUAGGUUGGUGCAAAAGUAAUUGCGGUUUUUGCCAUUACUUUCAGUAGCAAAAAUCUCAAUUACUUUUGCACCAACUUAAUACUU

>hsa-mir-548d-1

AAACAAGUUAUAUUAGGUUGGUGCAAAAGUAAUUGUGGUUUUUGCCUGUAAAAGUAAUGGCAAAAACCACAGUUUCUUUUGCACCAGACUAAUAAAG

>hsa-mir-548d-2

GAGAGGGAAGAUUUAGGUUGGUGCAAAAGUAAUUGUGGUUUUUGCCAUUGAAAGUAAUGGCAAAAACCACAGUUUCUUUUGCACCAACCUAAUAAAA

>hsa-mir-548e

UUAUUAGGUUGGUACAAAAGCAAUCGCGGUUUUUGCUAUUACUUUUAAAGGCAAAAACUGAGACUACUUUUGCACCAACCUGAUAGAA

>hsa-mir-548f-1

AUUAGGUUGGUGCAAAAGUAAUCACAGUUUUUGACAUUACUUUCAAAGACAAAAACUGUAAUUACUUUUGGACCAACCUAAUAG

>hsa-mir-548f-2

UAAUAACUAUUAGGUUGGUGCGAACAUAAUUGCAGUUUUUAUCAUUACUUUUAAUGGCAAAAACUGUAAUUACUUUUGCACCAACCUAAUAUUUUAGU

>hsa-mir-548f-3

AUUAGGUUGGUGCAAACCUAAUUGCAAUUUUUGCAGUUUUUUUAAGUAAUUGCAAAAACUGUAAUUACUUUUGCACCAACCUAAUAC

>hsa-mir-548f-4

GAGUUCUAACGUAUUAGGUUGGUGCAAAAGUAAUAGUGGUUUUUGCCAUUAAAAGUAAUGACAAAAACUGUAAUUACUUUUGGAACAAUAUUAAUAGAAUUUCAG

>hsa-mir-548g

AGUUAUUAGAUUAGUGCAAAAGUAAUUGCAGUUUUUGCAUUACGUUCUAUGGCAAAACUGUAAUUACUUUUGUACCAACAUAAUACUUC

>hsa-mir-548h-1

UCUGUCCAUUAGGUGGGUGCAAAAGUAAUCGCGGUUUUUGUCAUUACUUUUAAUGGUAAAAACUGGAAUUACUUUUGCACUGACCUAAUAUUAAGCCAGAUA

>hsa-mir-548h-2

GUAUUAGGUUGGUGCAAAAGUAAUCGCGGUUUUUGUCAUUACUUUCAAUGGCAAACACCACAAUUACUUUUGCACCAACCUAAUAUAA

>hsa-mir-548h-3

UCUGAUUCUGCAUGUAUUAGGUUGGUGCAAAAGUAAUCGCGGUUUUUGUCAUUGAAAGUAAUAGCAAAAACUGCAAUUACUUUUGCACCAACCUAAAAGUAGUCACUGUCUUCAGAUA

>hsa-mir-548h-4

GCUAUUAGGUUGGUGCAAAAGUAAUCGCGGUUUUUGUCAUUACUUUAAUUACUUUACGUUUCAUUAAUGACAAAAACCGCAAUUACUUUUGCACCAACCUAAUACUUGCUA

>hsa-mir-548h-5

ACAAAAGUAAUCGCGGUUUUUGUCAUUACUUUUAACUGUAAAAACCACGGUUGCUUUUGC

>hsa-mir-548i-1

CAGAUGGCUCUGAAGUUUGCACCCUAUUAGGUUGGUGCAAAAGUAAUUGCGGAUUUUGCCAUUAAAAGUAAUGGCAAAAAUAGCAAUUAUUUUUGUACCAGCCUAGUAUCUUUUCUCCUUCUACCAAACUUUGUCCCUGAGCCAUCUCA

>hsa-mir-548i-2

UAGAUGGCUCCGAAGUUUGCAUCCUAUUAGUUUGGUGCAAAAGUAAUUGCGGAUUUUGCCAUUAAAAGUAAUGGCAAAAAUAGCAAUUAUUUUUGUACCAGCCUAGUAUCUUUUCUCCUUCUAACAAAGUUCGUCCCUGAUCCAUCUCA

>hsa-mir-548i-3

CAGAUGGCUCCGAAGUUUACAUCCUAUUAGGUUUGUGCAAAAGUAAUUGCGGAUUUUGCCAUUAAAAGUAAUGGCAAAAAUAGCAAUUAUUUUUGUACCAGCCUAGUAUCUUUUCUCCUUCUACCAAACUUUGUCCCUGAGCCAUCUCA

>hsa-mir-548i-4

AGGUUGGUGCAAAAGUAAUUGCGGAUUUUGCCAUACUUUUAACGGCAAAAACCACAAAUAUUAUUGCACCAACCUAU

>hsa-mir-548j

GGGCAGCCAGUGAAUAGUUAGCUGGUGCAAAAGUAAUUGCGGUCUUUGGUAUUACUUUCAGUGGCAAAAACUGCAUUACUUUUGCACCAGCCUACUAGAACGCUGAGUUCAG

>hsa-mir-548k

CUUUUCUCAAGUAUUGCUGUUAGGUUGGUGCAAAAGUACUUGCGGAUUUUGCUUUACUUUUAAUGGCAAAAACCGCAAUUAUUUUUGCUUCAACCUAAUAUGAUGCAAAAUUGGCU

>hsa-mir-548l

UAUUAGGUUGGUGCAAAAGUAUUUGCGGGUUUUGUCGUAGAAAGUAAUGGCAAAAACUGCAGUUACUUGUGCACCAACCAAAUGCU

>hsa-mir-548n

AGGUUGGUGCAAAAGUAAUUGUGGAUUUUGUCGUUAAAAAUAGCAAAACCCGCAAUUACUUUUGCACCAACCUAA

>hsa-mir-548o

UGGUGAAAAUGUGUUGAUUGUAAUGGUUCCUAUUCUGAUCAAUAAACAUGGUUUGAGCCUAGUUACAAUGAUCUAAAAUUCACGGUCCAAAACUGCAGUUACUUUUGCACCAAC

>hsa-mir-548o-2

UGGUGCAAAAGUAAUUGCGGUUUUUGCCAUUAAAAGUAAUGCGGCCAAAACUGCAGUUACUUUUGCACCC

>hsa-mir-548p

AUUAGGUUGGUAUAAAAUUAAUUGCAGUUUUUGUCAUUACUUUCAAUAGCAAAAACUGCAGUUACUUUUGCACCAAUGUAAUAC

>hsa-mir-548q

AUAUUAGGCUGGUGCAAAAGUAAUGGCGGUUUUUGCCAUUACUUUUCAUUUUUACCAUUAAAAGUAAUGGCAAAAAGCAUGAUUACUUUUUCACCAACCU

>hsa-mir-548s

UUGCUGCAAAAAUAAUUGCAGUUUUUGCCAUUAUUUUUAAUAAUUAUAAUAAUGGCCAAAACUGCAGUUAUUUUUGCACCAA

>hsa-mir-548t

AGGGUGGUGCAAAAGUGAUCGUGGUUUUUGCAAUUUUUUAAUGACAAAAACCACAAUUACUUUUGCACCAACCU

>hsa-mir-548u

AUUAGGAUGGUGCAAAAGUAAUGUGGUUUUUUUCUUUACUUUUAAUGGCAAAGACUGCAAUUACUUUUGCGCCAACCUAAU

>hsa-mir-548v

AAUACUAGGUUUGAGCAAAAGUAAUUGCGGUUUUGCCAUCAUGCCAAAAGCUACAGUUACUUUUGCACCAGCCUAAUAUU

>hsa-mir-548w

GGUUGGUGCAAAAGUAACUGCGGUUUUUGCCUUUCAACAUAAUGGCAAAACCCACAAUUACUUUUGCACCAAUC

>hsa-mir-548x

AGGUUAGUGCAAAAGUAAUUGCAGUUUUUGCGUUACUUUCAAUCGUAAAAACUGCAAUUACUUUCACACCAAUCU

>hsa-mir-548x-2

AUGCCAAAUAUUAGGUUGGCACAAAAGUAAUUGUGGCUUUUGCCAUUAAAAGUAAUGGUAAAAACUGCAAUUACUUUCGUGCCAACCUAAUAUUUGUGUG

>hsa-mir-548y

GCCUAAACUAUUAGGUUGGUGCAAAAGUAAUCACUGUUUUUGCCAUUACUCUCAGUGGCAAAAACCGUGAUUACUUUUGCACCAACCUAGUAACACCUUCACUGUGGGGG

>hsa-mir-549a

AGACAUGCAACUCAAGAAUAUAUUGAGAGCUCAUCCAUAGUUGUCACUGUCUCAAAUCAGUGACAACUAUGGAUGAGCUCUUAAUAUAUCCCAGGC

>hsa-mir-550a-1

UGAUGCUUUGCUGGCUGGUGCAGUGCCUGAGGGAGUAAGAGCCCUGUUGUUGUAAGAUAGUGUCUUACUCCCUCAGGCACAUCUCCAACAAGUCUCU

>hsa-mir-550a-2

UGAUGCUUUGCUGGCUGGUGCAGUGCCUGAGGGAGUAAGAGCCCUGUUGUUGUCAGAUAGUGUCUUACUCCCUCAGGCACAUCUCCAGCGAGUCUCU

>hsa-mir-550a-3

GAUGCUUUGCUGGCUGGUGCAGUGCCUGAGGGAGUAAGAGUCCUGUUGUUGUAAGAUAGUGUCUUACUCCCUCAGGCACAUCUCCAACAAGUCUC

>hsa-mir-550b-1

AGAGACUUGUUGGAGAUGUGCCUGAGGGAGUAAGACACUAUCUUACAACAACAGGGCUCUUACUCCCUCAGGCACUGCACCAGCCAGCAAAGCAUCA

>hsa-mir-550b-2

AGAGACUCGCUGGAGAUGUGCCUGAGGGAGUAAGACACUAUCUGACAACAACAGGGCUCUUACUCCCUCAGGCACUGCACCAGCCAGCAAAGCAUCA

>hsa-mir-551a

GGGGACUGCCGGGUGACCCUGGAAAUCCAGAGUGGGUGGGGCCAGUCUGACCGUUUCUAGGCGACCCACUCUUGGUUUCCAGGGUUGCCCUGGAAA

>hsa-mir-552

AACCAUUCAAAUAUACCACAGUUUGUUUAACCUUUUGCCUGUUGGUUGAAGAUGCCUUUCAACAGGUGACUGGUUAGACAAACUGUGGUAUAUACA

>hsa-mir-555

GGAGUGAACUCAGAUGUGGAGCACUACCUUUGUGAGCAGUGUGACCCAAGGCCUGUGGACAGGGUAAGCUGAACCUCUGAUAAAACUCUGAUCUAU

>hsa-mir-556

GAUAGUAAUAAGAAAGAUGAGCUCAUUGUAAUAUGAGCUUCAUUUAUACAUUUCAUAUUACCAUUAGCUCAUCUUUUUUAUUACUACCUUCAACA

>hsa-mir-5579

UAUGGUACUCCUUAAGCUAACAGGCCCCUGUCACCAUUAGCUUAAGGAGUACCAGAUC

>hsa-mir-558

GUGUGUGUGUGUGUGUGUGGUUAUUUUGGUAUAGUAGCUCUAGACUCUAUUAUAGUUUCCUGAGCUGCUGUACCAAAAUACCACAAACGGGCUG

>hsa-mir-5581

AGCCUUCCAGGAGAAAUGGAGACCCUAUACAUACCUGUUUCCAUGCCUCCUAGAAGUUCC

>hsa-mir-5582

UAGGCACACUUAAAGUUAUAGCUACAUCAGUUAUAACUAUAUCAGUUAAAACUUUAAGUGUGCCUAGG

>hsa-mir-5585

UGAAGUACCAGCUACUCGAGAGGUCAGAGGAUUGCUCCUGAAUAGCUGGGACUACAGGU

>hsa-mir-5586

UAUCCAGCUUGUUACUAUAUGCUUUUUAAAUGGGGCACAGAGUGACAAGCUGGUUAAAG

>hsa-mir-5587

AUGGUCACCUCCGGGACUCAGCCCUGUGCUGAGCCCCGGGCAGUGUGAUCAUC

>hsa-mir-5588

ACUGGCAUUAGUGGGACUUUUUUUUUUUUUUUUUUUAAUGUUAAAAGUCCCACUAAUGCCAGC

>hsa-mir-564

CGGGCAGCGGGUGCCAGGCACGGUGUCAGCAGGCAACAUGGCCGAGAGGCCGGGGCCUCCGGGCGGCGCCGUGUCCGCGACCGCGUACCCUGAC

>hsa-mir-5680

GCAUUGGGUUAGCAGGUUAGCCCAGCAUUUCCCUUCCUGGACACACAGGAGGAGAAAUGCUGGACUAAUCUGCUAAUCCAAUGC

>hsa-mir-5682

GGCCCAUGGGUCUUAUCCUGCAAGGUGCUGCAGAGACGAGGCCUGUAGCACCUUGCAGGAUAAGGUCUACUGGGCC

>hsa-mir-5684

GCUGAACUCUAGCCUGAGCAACAGAGUGAGAUGGUCUUGUUUUGUUGCCCAGGCUGGAGUCCAGU

>hsa-mir-5685

CUCUACAUCACAGCCCAGCAGUUAUCACGGGCCCCUCCCCUCAAUGGGCCCGUGAUAACUGCAGGGCUGUGAUGUAGAG

>hsa-mir-5687

CCUCACUUAUCUGACUCUGAAAUCUUCUAAAUGGUACCCACUUUAUUUAGAACGUUUUAGGGUCAAAUAAGUACAGG

>hsa-mir-5690

CUUUUAAUUUCAGCUACUACCUCUAUUAGGAUUUGGGAGUUAUACUAAUAGAGGUAAUAGUUGAAAUUAAGAG

>hsa-mir-5692a-1

GACAGUACAAAUAAUACCACAGUGGGUGUACCUCAUGUGUGUACACCCUGUGAUAUUAUUUGUAAUAUC

>hsa-mir-5692c-1

UAUAACAUUGUAUAUACCCACUGUGAUAUUAAGAGUAAUAGCUCUCUAGGUUAUUAUGAAUAAUAUCACAGUAGGUGUACACAAUGUUGUA

>hsa-mir-5692c-2

UGUGUACACCAACUGUGAUAUUAGGAGUCCUAUUUAUUUUUAGGAUAUUAGGAAUAAUAUCACAGUAGGUGUACACA

>hsa-mir-5693

CUGGGAAGUUAGUUCAUUUCAGUCUGUGCUGUGAGCUAGCCAGCAGUGGCUCUGAAAUGAACUCAAACUCUAG

>hsa-mir-5694

GCCAACUGCAGAUCAUGGGACUGUCUCAGCCCCAUAUGUAUCUGAAGGCUGAGAAGUCCCAUGAUCCGCACUUGGC

>hsa-mir-5695

CAAGGCCUAUCUAUCUAGAUUCUUCUUGGCCUCUCUGAGCAUGCAUUCCUGAGACUCCAAGAAGAAUCUAGACAGAUAGGCCUUG

>hsa-mir-5696

GUGCUCAUUUAAGUAGUCUGAUGCCUACUACUGAUGACAUACAAUGUAAGUGCUCAUUUAGGCGUCAGACUACCUAAAUGAGCAC

>hsa-mir-5697

AGCAUAUUCUCAAGUAGUUUCAUGAUAAAGGGUGUAUGAGAGAUCAACCCUUUAUCAUGAAACGCUUGAGGAUACGCU

>hsa-mir-5698

CUGUGCACCUGGGGGAGUGCAGUGAUUGUGGAAUGCAAAGUCCCACAAUCACUGUACUCCCCAGGUGCACAG

>hsa-mir-5699

CUGUACCCCUGCCCCAACAAGGAAGGACAAGAGGUGUGAGCCACACACACGCCUGGCCUCCUGUCUUUCCUUGUUGGAGCAGGGAUGUAG

>hsa-mir-570

CUAGAUAAGUUAUUAGGUGGGUGCAAAGGUAAUUGCAGUUUUUCCCAUUAUUUUAAUUGCGAAAACAGCAAUUACCUUUGCACCAACCUGAUGGAGU

>hsa-mir-5701-1

GAUUGGACUUUAUUGUCACGUUCUGAUUGGUUAGCCUAAGACUUGUUCUGAUCCAAUCAGAACAUGAAAAUAACGUCCAAUC

>hsa-mir-5701-2

GAUUGGACUUUAUUGUCACGUUCUGAUUGGUUAGCCUAAGACUUGUUCUGAUCCAAUCAGAACAUGAAAAUAACGUCCAAUC

>hsa-mir-5701-3

GAUUGGACUUUAUUGUCACGUUCUGAUUGGUUAGCCUAAGACUUGUUCUGAUCCAAUCAGAACAUGAAAAUAACGUCCAAUC

>hsa-mir-5704

UGAUCUUGUUUAGGCCAUCAUCCCAUUAUGCUAAGUCCAUGGGCAAACAUAACAGGAUGAUGGCCUAAACAAGACCA

>hsa-mir-5706

AGCUAGGUCUUCUGGAUAACAUGCUGAAGCUUCUACGUCAUUCAGCACUUGCUUCAGCAUGUUUUCCAGAGGAUCUAGCU

>hsa-mir-5708

AUUACAGACAUGAGCGACUGUGCCUGACCAAAAGUCAACAUUAAACAACAAAUCUUGGCCAGGCACAGUGGCUCAUGCCUGUAAU

>hsa-mir-572

GUCGAGGCCGUGGCCCGGAAGUGGUCGGGGCCGCUGCGGGCGGAAGGGCGCCUGUGCUUCGUCCGCUCGGCGGUGGCCCAGCCAGGCCCGCGGGA

>hsa-mir-573

UUUAGCGGUUUCUCCCUGAAGUGAUGUGUAACUGAUCAGGAUCUACUCAUGUCGUCUUUGGUAAAGUUAUGUCGCUUGUCAGGGUGAGGAGAGUUUUUG

>hsa-mir-574

GGGACCUGCGUGGGUGCGGGCGUGUGAGUGUGUGUGUGUGAGUGUGUGUCGCUCCGGGUCCACGCUCAUGCACACACCCACACGCCCACACUCAGG

>hsa-mir-576

UACAAUCCAACGAGGAUUCUAAUUUCUCCACGUCUUUGGUAAUAAGGUUUGGCAAAGAUGUGGAAAAAUUGGAAUCCUCAUUCGAUUGGUUAUAACCA

>hsa-mir-577

UGGGGGAGUGAAGAGUAGAUAAAAUAUUGGUACCUGAUGAAUCUGAGGCCAGGUUUCAAUACUUUAUCUGCUCUUCAUUUCCCCAUAUCUACUUAC

>hsa-mir-578

AGAUAAAUCUAUAGACAAAAUACAAUCCCGGACAACAAGAAGCUCCUAUAGCUCCUGUAGCUUCUUGUGCUCUAGGAUUGUAUUUUGUUUAUAUAU

>hsa-mir-579

CAUAUUAGGUUAAUGCAAAAGUAAUCGCGGUUUGUGCCAGAUGACGAUUUGAAUUAAUAAAUUCAUUUGGUAUAAACCGCGAUUAUUUUUGCAUCAAC

>hsa-mir-580

AUAAAAUUUCCAAUUGGAACCUAAUGAUUCAUCAGACUCAGAUAUUUAAGUUAACAGUAUUUGAGAAUGAUGAAUCAUUAGGUUCCGGUCAGAAAUU

>hsa-mir-581

GUUAUGUGAAGGUAUUCUUGUGUUCUCUAGAUCAGUGCUUUUAGAAAAUUUGUGUGAUCUAAAGAACACAAAGAAUACCUACACAGAACCACCUGC

>hsa-mir-582

AUCUGUGCUCUUUGAUUACAGUUGUUCAACCAGUUACUAAUCUAACUAAUUGUAACUGGUUGAACAACUGAACCCAAAGGGUGCAAAGUAGAAACAUU

>hsa-mir-583

AACUCACACAUUAACCAAAGAGGAAGGUCCCAUUACUGCAGGGAUCUUAGCAGUACUGGGACCUACCUCUUUGGU

>hsa-mir-584

UAGGGUGACCAGCCAUUAUGGUUUGCCUGGGACUGAGGAAUUUGCUGGGAUAUGUCAGUUCCAGGCCAACCAGGCUGGUUGGUCUCCCUGAAGCAAC

>hsa-mir-585

UGGGGUGUCUGUGCUAUGGCAGCCCUAGCACACAGAUACGCCCAGAGAAAGCCUGAACGUUGGGCGUAUCUGUAUGCUAGGGCUGCUGUAACAA

>hsa-mir-586

AUGGGGUAAAACCAUUAUGCAUUGUAUUUUUAGGUCCCAAUACAUGUGGGCCCUAAAAAUACAAUGCAUAAUGGUUUUUCACUCUUUAUCUUCUUAU

>hsa-mir-588

AGCUUAGGUACCAAUUUGGCCACAAUGGGUUAGAACACUAUUCCAUUGUGUUCUUACCCACCAUGGCCAAAAUUGGGCCUAAG

>hsa-mir-589

UCCAGCCUGUGCCCAGCAGCCCCUGAGAACCACGUCUGCUCUGAGCUGGGUACUGCCUGUUCAGAACAAAUGCCGGUUCCCAGACGCUGCCAGCUGGCC

>hsa-mir-590

UAGCCAGUCAGAAAUGAGCUUAUUCAUAAAAGUGCAGUAUGGUGAAGUCAAUCUGUAAUUUUAUGUAUAAGCUAGUCUCUGAUUGAAACAUGCAGCA

>hsa-mir-592

UAUUAUGCCAUGACAUUGUGUCAAUAUGCGAUGAUGUGUUGUGAUGGCACAGCGUCAUCACGUGGUGACGCAACAUCAUGACGUAAGACGUCACAAC

>hsa-mir-597

UACUUACUCUACGUGUGUGUCACUCGAUGACCACUGUGAAGACAGUAAAAUGUACAGUGGUUCUCUUGUGGCUCAAGCGUAAUGUAGAGUACUGGUC

>hsa-mir-598

GCUUGAUGAUGCUGCUGAUGCUGGCGGUGAUCCCGAUGGUGUGAGCUGGAAAUGGGGUGCUACGUCAUCGUUGUCAUCGUCAUCAUCAUCAUCCGAG

>hsa-mir-599

AAAGACAUGCUGUCCACAGUGUGUUUGAUAAGCUGACAUGGGACAGGGAUUCUUUUCACUGUUGUGUCAGUUUAUCAAACCCAUACUUGGAUGAC

>hsa-mir-600

AAGUCACGUGCUGUGGCUCCAGCUUCAUAGGAAGGCUCUUGUCUGUCAGGCAGUGGAGUUACUUACAGACAAGAGCCUUGCUCAGGCCAGCCCUGCCC

>hsa-mir-602

UUCUCACCCCCGCCUGACACGGGCGACAGCUGCGGCCCGCUGUGUUCACUCGGGCCGAGUGCGUCUCCUGUCAGGCAAGGGAGAGCAGAGCCCCCCUG

>hsa-mir-605

GCCCUAGCUUGGUUCUAAAUCCCAUGGUGCCUUCUCCUUGGGAAAAACAGAGAAGGCACUAUGAGAUUUAGAAUCAAGUUAGG

>hsa-mir-606

UGUAUCCUUGGUUUUUAGUAGUUUUACUAUGAUGAGGUGUGCCAUCCACCCCAUCAUAGUAAACUACUGAAAAUCAAAGAUACAAGUGCCUGACCA

>hsa-mir-607

UUGCCUAAAGUCACACAGGUUAUAGAUCUGGAUUGGAACCCAGGGAGCCAGACUGCCUGGGUUCAAAUCCAGAUCUAUAACUUGUGUGACUUUGGG

>hsa-mir-6084

AGCGUCCAGGCUGGGCCGCAGGACCGGGCGCGGAGCCUCGCAGGGUCGGGCUCGGGCUCCCUAACCGUCUCCGCUUCUUCCGCCAGUCGGUGGCCGGGCUGGCGGCGCGG

>hsa-mir-610

UCUAUUUGUCUUAGGUGAGCUAAAUGUGUGCUGGGACACAUUUGAGCCAAAUGUCCCAGCACACAUUUAGCUCACAUAAGAAAAAUGGACUCUAGU

>hsa-mir-612

UCCCAUCUGGACCCUGCUGGGCAGGGCUUCUGAGCUCCUUAGCACUAGCAGGAGGGGCUCCAGGGGCCCUCCCUCCAUGGCAGCCAGGACAGGACUCUCA

>hsa-mir-6125

GCUCUGGGGCGUGCCGCCGCCGUCGCUGCCACCUCCCCUACCGCUAGUGGAAGAAGAUGGCGGAAGGCGGAGCGGCGGAUCUGGACACCCAGCGGU

>hsa-mir-6127

ACUUUCUCCAUCUACCAAGAUGAGGGAGUGGGUGGGAGGUGGGAAGGCUGCCCCAAAUGGCCUCUAACAUCCCUUCCAGUCUCCUCCUCCUCCUCCUCCUUCUUCUUCU

>hsa-mir-6133

GGAAUGUCACCUGUGUGUUUUCUCUGCAUGCCCUCUUCAUUGUUCUGCUGAAGACUGGUCUCUUCAUGUGUGAGGGAGGAGGUUGGGUAUUGAGGGAAAACAGGGGGC

>hsa-mir-615

CUCGGGAGGGGCGGGAGGGGGGUCCCCGGUGCUCGGAUCUCGAGGGUGCUUAUUGUUCGGUCCGAGCCUGGGUCUCCCUCUUCCCCCCAACCCCCC

>hsa-mir-616

UUAGGUAAUUCCUCCACUCAAAACCCUUCAGUGACUUCCAUGACAUGAAAUAGGAAGUCAUUGGAGGGUUUGAGCAGAGGAAUGACCUGUUUUAAAA

>hsa-mir-617

CAUCAUAAGGAGCCUAGACUUCCCAUUUGAAGGUGGCCAUUUCCUACCACCUUCAAAUGGUAAGUCCAGGCUCCUUCUGAUUCAAUAAAUGAGGAGC

>hsa-mir-618

CUCUUGUUCACAGCCAAACUCUACUUGUCCUUCUGAGUGUAAUUACGUACAUGCAGUAGCUCAGGAGACAAGCAGGUUUACCCUGUGGAUGAGUCUGA

>hsa-mir-619

CGCCCACCUCAGCCUCCCAAAAUGCUGGGAUUACAGGCAUGAGCCACUGCGGUCGACCAUGACCUGGACAUGUUUGUGCCCAGUACUGUCAGUUUGCAG

>hsa-mir-622

AGAGAAGCUGGACAAGUACUGGUCUCAGCAGAUUGAGGAGAGCACCACAGUGGUCAUCACACAGUCUGCUGAGGUUGGAGCUGCUGAGAUGACACU

>hsa-mir-624

AAUGCUGUUUCAAGGUAGUACCAGUACCUUGUGUUCAGUGGAACCAAGGUAAACACAAGGUAUUGGUAUUACCUUGAGAUAGCAUUACACCUAAGUG

>hsa-mir-625

AGGGUAGAGGGAUGAGGGGGAAAGUUCUAUAGUCCUGUAAUUAGAUCUCAGGACUAUAGAACUUUCCCCCUCAUCCCUCUGCCCU

>hsa-mir-627

UACUUAUUACUGGUAGUGAGUCUCUAAGAAAAGAGGAGGUGGUUGUUUUCCUCCUCUUUUCUUUGAGACUCACUACCAAUAAUAAGAAAUACUACUA

>hsa-mir-628

AUAGCUGUUGUGUCACUUCCUCAUGCUGACAUAUUUACUAGAGGGUAAAAUUAAUAACCUUCUAGUAAGAGUGGCAGUCGAAGGGAAGGGCUCAU

>hsa-mir-629

UCCCUUUCCCAGGGGAGGGGCUGGGUUUACGUUGGGAGAACUUUUACGGUGAACCAGGAGGUUCUCCCAACGUAAGCCCAGCCCCUCCCCUCUGCCU

>hsa-mir-632

CGCCUCCUACCGCAGUGCUUGACGGGAGGCGGAGCGGGGAACGAGGCCGUCGGCCAUUUUGUGUCUGCUUCCUGUGGGACGUGGUGGUAGCCGU

>hsa-mir-635

CAGAGAGGAGCUGCCACUUGGGCACUGAAACAAUGUCCAUUAGGCUUUGUUAUGGAAACUUCUCCUGAUCAUUGUUUUGUGUCCAUUGAGCUUCCAAU

>hsa-mir-636

UGGCGGCCUGGGCGGGAGCGCGCGGGCGGGGCCGGCCCCGCUGCCUGGAAUUAACCCCGCUGUGCUUGCUCGUCCCGCCCGCAGCCCUAGGCGGCGUCG

>hsa-mir-637

UGGCUAAGGUGUUGGCUCGGGCUCCCCACUGCAGUUACCCUCCCCUCGGCGUUACUGAGCACUGGGGGCUUUCGGGCUCUGCGUCUGCACAGAUACUUC

>hsa-mir-639

UGGCCGACGGGGCGCGCGCGGCCUGGAGGGGCGGGGCGGACGCAGAGCCGCGUUUAGUCUAUCGCUGCGGUUGCGAGCGCUGUAGGGAGCCUGUGCUG

>hsa-mir-640

GUGACCCUGGGCAAGUUCCUGAAGAUCAGACACAUCAGAUCCCUUAUCUGUAAAAUGGGCAUGAUCCAGGAACCUGCCUCUACGGUUGCCUUGGGG

>hsa-mir-641

UGGGUGAAAGGAAGGAAAGACAUAGGAUAGAGUCACCUCUGUCCUCUGUCCUCUACCUAUAGAGGUGACUGUCCUAUGUCUUUCCUUCCUCUUACCCCU

>hsa-mir-642a

AUCUGAGUUGGGAGGGUCCCUCUCCAAAUGUGUCUUGGGGUGGGGGAUCAAGACACAUUUGGAGAGGGAACCUCCCAACUCGGCCUCUGCCAUCAUU

>hsa-mir-642b

GAGUUGGGAGGUUCCCUCUCCAAAUGUGUCUUGAUCCCCCACCCCAAGACACAUUUGGAGAGGGACCCUCCCAACUC

>hsa-mir-643

ACCAAGUGAUAUUCAUUGUCUACCUGAGCUAGAAUACAAGUAGUUGGCGUCUUCAGAGACACUUGUAUGCUAGCUCAGGUAGAUAUUGAAUGAAAAA

>hsa-mir-6500

CCUGCCUGCAGAAAGGAGCUAUCCACUCCAGGUGUCCUUUCUUCUGAGAGCUGGACACUUGUUGGGAUGACCUGCCUGCAGGUAGG

>hsa-mir-6501

GGAGUUGCCAGGGCUGCCUUUGGUGACAGCAGCAGUAGAGUUGCCAGAGCAGCCUGCGGUAACAGUA

>hsa-mir-6502

CAGAGUGGGAGCUCUAGAAAGAUUGUUGACCAAUCAUCUUAUUGACUAGACCAUCUUUCUAGAGUAUAACUAUUUU

>hsa-mir-6503

AAUGGUCCCCCCAGGGAGGUCUGCAUUCAAAUCCCCAGAAGCUGAGGAUUAGGGGACUAGGAUGCAGACCUCCCUGGGGGACCAUU

>hsa-mir-6504

GCAGUCUGGCUGUGCUGUAAUGCAGUCUGCACCCUGCUGCAUUACAGCACAGCCAUUCUCU

>hsa-mir-6506

GACUGGGAUGUCACUGAAUAUGGUGUUUGUGAGUUGAUUGACAUCGUAUCAGAGAUUCCAGACACA

>hsa-mir-6508

UUCCUCUAGAAAUGCAUGACCCACCCUGAGUUUUGGUGGGCCAUGCAUUUCUAGAACUCC

>hsa-mir-6509

UUUUUGUGUGUGAAAUUAGGUAGUGGCAGUGGAACACUAUAUUAAUCAGGUUUCCACUGCCACUACCUAAUUUCUCAGAUGGAAA

>hsa-mir-651

AAUCUAUCACUGCUUUUUAGGAUAAGCUUGACUUUUGUUCAAAUAAAAAUGCAAAAGGAAAGUGUAUCCUAAAAGGCAAUGACAGUUUAAUGUGUUU

>hsa-mir-6510

AGCAGCAGGGGAGAGAGAGGAGUCCUCUAGACACCGACUCUGUCUCCUGCAGAU

>hsa-mir-6511a-1

CCUGCAGGCAGAAGUGGGGCUGACAGGGCAGAGGGUUGCGCCCCCUCACCAUCCCUUCUGCCUGCAG

>hsa-mir-6511a-2

CCUGCAGGCAGAAGUGGGGCUGACAGGGCAGAGGGUUGCGCCCCCUCACCAUCCCUUCUGCCUGCAG

>hsa-mir-6511a-3

CCUGCAGGCAGAAGUGGGGCUGACAGGGCAGAGGGUUGCGCCCCCUCACCAUCCCUUCUGCCUGCAG

>hsa-mir-6511a-4

CCUGCAGGCAGAAGUGGGGCUGACAGGGCAGAGGGUUGCGCCCCCUCACCAUCCCUUCUGCCUGCAG

>hsa-mir-6511b-1

GGGACGGGGCCUGCAGGCAGAAGUGGGGCUGACAGGGCAGAGGGUUGCGCCCCCUCACCACCCCUUCUGCCUGCAGCGGUGGGCU

>hsa-mir-6511b-2

GGGGCCUGCAGGCAGAAGUGGGGCUGACAGGGCAGAGGGUUGCGCCCCCUCACCACCCCUUCUGCCUGCAG

>hsa-mir-6513

GCUUUGGGAUUGACGCCACAUGUCUCAGGUCCCCAGCUGAGUCAAGUGUCAUCUGUCCCUAGGC

>hsa-mir-6514

UAUGGAGUGGACUUUCAGCUGGCAUUUACGAGUCAGAGUUCUUACAGAGCUGCCUGUUCUUCCACUCCAG

>hsa-mir-6515

CAUUGGAGGGUGUGGAAGACAUCUGGGCCAACUCUGAUCUCUUCAUCUACCCCCCAG

>hsa-mir-6516

UGGGUUUUGAAUUUGCAGUAACAGGUGUGAGCAUUCUAGCAGCAGUUUGAUGAUCAUGUAUGAUACUGCAAACAGGACCUA

>hsa-mir-652

ACGAAUGGCUAUGCACUGCACAACCCUAGGAGAGGGUGCCAUUCACAUAGACUAUAAUUGAAUGGCGCCACUAGGGUUGUGCAGUGCACAACCUACAC

>hsa-mir-6529

AACCUGUUCUCUUGAGAGAUCAGAGGCGCAGAGUGCGUCAGUGUCAAUGAAGCCUGUGCCUUUUACUUCUUUAAGAGCGUACGGU

>hsa-mir-653

UUCAUUCCUUCAGUGUUGAAACAAUCUCUACUGAACCAGCUUCAAACAAGUUCACUGGAGUUUGUUUCAAUAUUGCAAGAAUGAUAAGAUGGAAGC

>hsa-mir-654

GGGUAAGUGGAAAGAUGGUGGGCCGCAGAACAUGUGCUGAGUUCGUGCCAUAUGUCUGCUGACCAUCACCUUUAGAAGCCC

>hsa-mir-656

CUGAAAUAGGUUGCCUGUGAGGUGUUCACUUUCUAUAUGAUGAAUAUUAUACAGUCAACCUCUUUCCGAUAUCGAAUC

>hsa-mir-658

GCUCGGUUGCCGUGGUUGCGGGCCCUGCCCGCCCGCCAGCUCGCUGACAGCACGACUCAGGGCGGAGGGAAGUAGGUCCGUUGGUCGGUCGGGAACGAGG

>hsa-mir-659

UACCGACCCUCGAUUUGGUUCAGGACCUUCCCUGAACCAAGGAAGAGUCACAGUCUCUUCCUUGGUUCAGGGAGGGUCCCCAACAAUGUCCUCAUGG

>hsa-mir-660

CUGCUCCUUCUCCCAUACCCAUUGCAUAUCGGAGUUGUGAAUUCUCAAAACACCUCCUGUGUGCAUGGAUUACAGGAGGGUGAGCCUUGUCAUCGUG

>hsa-mir-663a

CCUUCCGGCGUCCCAGGCGGGGCGCCGCGGGACCGCCCUCGUGUCUGUGGCGGUGGGAUCCCGCGGCCGUGUUUUCCUGGUGGCCCGGCCAUG

>hsa-mir-663b

GGUGCCGAGGGCCGUCCGGCAUCCUAGGCGGGUCGCUGCGGUACCUCCCUCCUGUCUGUGGCGGUGGGAUCCCGUGGCCGUGUUUUCCUGGUGGCCCGGCCGUGCCUGAGGUUUC

>hsa-mir-664a

GAACAUUGAAACUGGCUAGGGAAAAUGAUUGGAUAGAAACUAUUAUUCUAUUCAUUUAUCCCCAGCCUACAAAAUGAAAAAA

>hsa-mir-664b

UGGGCUAAGGGAGAUGAUUGGGUAGAAAGUAUUAUUCUAUUCAUUUGCCUCCCAGCCUACA

>hsa-mir-670

GUUUAGGGGUGGACCUGAUGUCCCUGAGUGUAUGUGGUGAACCUGAAUUUGCCUUGGGUUUCCUCAUAUUCAUUCAGGAGUGUCAGUUGCCCCUUCAC

>hsa-mir-671

GCAGGUGAACUGGCAGGCCAGGAAGAGGAGGAAGCCCUGGAGGGGCUGGAGGUGAUGGAUGUUUUCCUCCGGUUCUCAGGGCUCCACCUCUUUCGGGCCGUAGAGCCAGGGCUGGUGC

>hsa-mir-6716

GAGAGGCCAAGACCUUGGGAAUGGGGGUAAGGGCCUUCUGAGCCCAGGUCCGAACUCUCCAUUCCUCUGCAGAGCGCUCU

>hsa-mir-6717

CUGGUGUUUGAGGCGAUGUGGGGAUGUAGAGACAACUUCCCAGUCUCAUUUCCUCAUCCUGCCAGGCCACCAU

>hsa-mir-6718

AGUGAAUCCCUAGUGGUCAGAGGGCUUAUGAUAUAUUGUGAGAGCCAUGUCAUAAGCCUUUUGGCCACUAGGGAUUCAAU

>hsa-mir-6720

UUGAGCGAGAGAUUGUGGCGCACCGAGUUCUUCCAGCCCUGGUAGGCGCCGCGGAAGAAGGGGAAGCGCGCCUGCAGGAACUGGUAGAUCUCGCUGAG

>hsa-mir-6721

CCCUCAUCUCUGGGCAGGGGCUUAUUGUAGGAGUCUCUGAAGAGAGCUGUGGACUGACCUGCUUUAACCCUUCCCCAGGUUCCCAUU

>hsa-mir-6724-1

CGCUGCGCUUCUGGGCCCGCGGCGGGCGUGGGGCUGCCCGGGCCGGUCGACCAGCGCGCCGUAGCUCCCGAGGCCCGAGCCGCGACCCGCGG

>hsa-mir-6724-2

CGCUGCGCUUCUGGGCCCGCGGCGGGCGUGGGGCUGCCCGGGCCGGUCGACCAGCGCGCCGUAGCUCCCGAGGCCCGAGCCGCGACCCGCGG

>hsa-mir-6724-3

CGCUGCGCUUCUGGGCCCGCGGCGGGCGUGGGGCUGCCCGGGCCGGUCGACCAGCGCGCCGUAGCUCCCGAGGCCCGAGCCGCGACCCGCGG

>hsa-mir-6724-4

CGCUGCGCUUCUGGGCCCGCGGCGGGCGUGGGGCUGCCCGGGCCGGUCGACCAGCGCGCCGUAGCUCCCGAGGCCCGAGCCGCGACCCGCGG

>hsa-mir-6727

GGGUGCUCGGGGCAGGCGGCUGGGAGCGGCCCUCACAUUGAUGGCUCCUGCCACCUCCUCCGCAG

>hsa-mir-6728

CUAGAUUGGGAUGGUAGGACCAGAGGGGCUUACUGCCCUGUGGGGCUCUCUGGACCCAGUGCCAUGCUUCUCUGCUCUGCUCUCCCCAG

>hsa-mir-6729

GAGGGUGGGCGAGGGCGGCUGAGCGGCUCCAUCCCCCGGCCUGCUCAUCCCCCUCGCCCUCUCAG

>hsa-mir-6730

CCGAAAGAAAGGUGGAGGGGUUGUCAGAGCUGCAGCUGCUCCACCCUGACACCCCAUCUGCCCUCAG

>hsa-mir-6731

ACAGGUGGGAGAGCAGGGUAUUGUGGAAGCUCCAGGUGCCAACCACCUGCCUCUAUUCCCCACUCUCCCCAG

>hsa-mir-6732

AGGCCUAGGGGGUGGCAGGCUGGCCAUCAGUGUGGGCUAACCCUGUCCUCUCCCUCCCAG

>hsa-mir-6733

GUGCUUGGGAAAGACAAACUCAGAGUUCCCUUCUUGUGAGCUCAGUGUCUGGAUUUCCUAG

>hsa-mir-6734

AGAACUUGAGGGGAGAAUGAGGUGGAGAAGCCCAGGUUCUGAAUCCCCUUCCCUCACUCUUCUCUCAG

>hsa-mir-6735

GCAGCCAGGGCAGAGGGCACAGGAAUCUGAGGUGACUGGCACAGAAGACUCAGGCCUGUGGCUCCUCCCUCAG

>hsa-mir-6736

CUGAGCUGGGUGAGGGCAUCUGUGGUUUGCUGGCUGCCUCAGCUCCUCUCUACCCACAG

>hsa-mir-6737

UUGGGUUGGGGUGGUCGGCCCUGGAGGGGGUUUGUUUGCUUAUUCCCCUCUGUGCUUCACCCCUACCCAG

>hsa-mir-6738

GAAGGCGAGGGGUAGAAGAGCACAGGGGUUCUGAUAAACCCUUCUGCCUGCAUUCUACUCCCAG

>hsa-mir-6739

GAAUGUGGGAAAGAGAAAGAACAAGUAAAAGGAAUUUUCAUUUUCCAGCCCCUAAUUGUUCUGUCUUUCUCCCAG

>hsa-mir-6740

GAAAGAGUUUGGGAUGGAGAGAGGAGAAACUUGAGGUCUCUGGGAGUUGCUUAAACCAGUUGACCGUAACCUGGCCAGAGAAUUCUGAUAGUGUCUUCUCUCCUCCCAAACAG

>hsa-mir-6741

AAUGGGUGGGUGCUGGUGGGAGCCGUGCCCUGGCCACUCAUUCGGCUCUCUCCCUCACCCUAG

>hsa-mir-6742

GAGGGAGUGGGGUGGGACCCAGCUGUUGGCCAUGGCGACAACACCUGGGUUGUCCCCUCUAG

>hsa-mir-6743

GGGUAAAGGGGCAGGGACGGGUGGCCCCAGGAAGAAGGGCCUGGUGGAGCCGCUCUUCUCCCUGCCCACAG

>hsa-mir-6746

CUUGCCCGGGAGAAGGAGGUGGCCUGGAGAGCUGCUGUCUCCAGCCGCCGCCUGUCUCCACAG

>hsa-mir-6747

UUUGGAGGGGUGUGGAAAGAGGCAGAACAUUCGUUCACUUUCCUGCCUUCCUCUGCACCAG

>hsa-mir-6748

UGGUGUGUGGGUGGGAAGGACUGGAUUUGAAAUGGUCCCACUCCUGACGAUCCUGUCCCUGUCUCCUACAG

>hsa-mir-6749

GGCCCUCGGGCCUGGGGUUGGGGGAGCUCUGUCCUGUCUCACUCAUUGCUCCUCCCCUGCCUGGCCCAG

>hsa-mir-675

CCCAGGGUCUGGUGCGGAGAGGGCCCACAGUGGACUUGGUGACGCUGUAUGCCCUCACCGCUCAGCCCCUGGG

>hsa-mir-6751

UCUUCUUGGGGGUGAGGUUGGUGUCUGGCCCCAGCAGCCCAGACUGAGCCUCUCUCUCUCCAG

>hsa-mir-6753

CACCAGGGCAGAGCAGGGCUGAUCAUCUCACGUCAGAGAGAGGGGAAGGGGCUGCCCAGUGAGCCCCCACAGGGCUCUACAUCUCCAGCUGGGCCUGGCUGGAGAUCCCAGGGUCCCUGAAGGCCCCCGCCACCGUUCUGGUCUGUCUCUGCCCUGGCACCCAG

>hsa-mir-6754

GGCUGCCAGGGAGGCUGGUUUGGAGGAGUCUGGUGGCCUGUUCUCUUCACCUGCCUCUGCCUGCAG

>hsa-mir-6755

UGUUUUAGGGUAGACACUGACAACGUUAUGUGUGGUCUUUAACCUGUUGUCAUGUUUUUUCCCUAG

>hsa-mir-6758

UGGGCUAGAGAGGGGAAGGAUGUGAUGUGAGCAGAUGGUUCUCACUCAUUCUCCUCUGUCCAG

>hsa-mir-6759

UAUUGUUGUGGGUGGGCAGAAGUCUGUUUUCUUCAUGGUUUUCUGACCUUUGCCUCUCCCCUCAG

>hsa-mir-676

GCAUGACUCUUCAACCUCAGGACUUGCAGAAUUAAUGGAAUGCUGUCCUAAGGUUGUUGAGUUGUGC

>hsa-mir-6761

UCUGCUCUGAGAGAGCUCGAUGGCAGGUGCCUCCGUGUUGCCGAACCCUCCUACGCUGCUCUCUCACUCCAG

>hsa-mir-6762

AGAGCCGGGGCCAUGGAGCAGCCUGUGUAGACGGGGACCUGCCCUGCAUGGGCACCCCCUCACUGGCUGCUUCCCUUGGUCUCCAG

>hsa-mir-6763

UUCUCCUGGGGAGUGGCUGGGGAGCAGACAGACCCAACCUCAUGCUCCCCGGCCUCUGCCCCCAG

>hsa-mir-6764

CUGACUCCCAGGGUCUGGUCAGAGUUGCUGAGUGGGUUGAUCUCUGGUCUUUCCUUGACAG

>hsa-mir-6765

GUGAGGCGGGGCCAGGAGGGUGUGUGGCGUGGGUGCUGCGGGGCCGUCAGGGUGCCUGCGGGACGCUCACCUGGCUGGCCCGCCCAG

>hsa-mir-6766

AUGAGCGGGUGGGAGCAGAUCUUAUUGAGAGUUCCUUCUCCUGCUCCUGAUUGUCUUCCCCCACCCUCACAG

>hsa-mir-6767

UGAAAUCGCAGACAGGGACACAUGGAGAACGCCCCCACCAGUUCCCACGUGCUUCUCUUUCCGCAG

>hsa-mir-6769a

AGGCCAGGUGGGUAUGGAGGAGCCCUCAUAUGGCAGUUGGCGAGGGCCCAGUGAGCCCCUCUCUGCUCUCCAG

>hsa-mir-6769b

CUUCCUGGUGGGUGGGGAGGAGAAGUGCCGUCCUCAUGAGCCCCUCUCUGUCCCACCCAUAG

>hsa-mir-6770-1

UAUCCUGAGAAGGCACAGCUUGCACGUGACCUCCUGGGCCUGGCGGCUGUGUCUUCACAG

>hsa-mir-6770-2

UAUCCUGAGAAGGCACAGCUUGCACGUGACCUCCUGGGCCUGGCGGCUGUGUCUUCACAG

>hsa-mir-6770-3

UAUCCUGAGAAGGCACAGCUUGCACGUGACCUCCUGGGCCUGGCGGCUGUGUCUUCACAG

>hsa-mir-6771

GGUGCCUCGGGAGGGCAUGGGCCAGGCCACAUAAUGAGCCAAACCCCUGUCUACCCGCAG

>hsa-mir-6772

AGGCCUGGGUGUAGGCUGGAGCUGAGGACUGAGGCUCACCUUGCUCCUGACUCUGUGCCCACAG

>hsa-mir-6774

UGUGCACUUGGGCAGGAGGGACCCUGUAUGUCUCCCCGCAGCACCGUCAUCGUGUCCCUCUUGUCCACAG

>hsa-mir-6775

GAACCUCGGGGCAUGGGGGAGGGAGGCUGGACAGGAGAGGGCUCACCCAGGCCCUGUCCUCUGCCCCAG

>hsa-mir-6776

CGGGCUCUGGGUGCAGUGGGGGUUCCCACGCCGCGGCAACCACCACUGUCUCUCCCCAG

>hsa-mir-6777

UCAAGACGGGGAGUCAGGCAGUGGUGGAGAUGGAGAGCCCUGAGCCUCCACUCUCCUGGCCCCCAG

>hsa-mir-6779

GAGCUCUGGGAGGGGCUGGGUUUGGCAGGACAGUUUCCAAGCCCUGUCUCCUCCCAUCUUCCAG

>hsa-mir-6780a

GACACUUGGGAGGGAAGACAGCUGGAGAGUAUGGUCACAGCAGCAUCCUCCUCUGUUUUCUUUCCUAG

>hsa-mir-6780b

CAGCCUGGGGAAGGCUUGGCAGGGAAGACACAUGAGCAGUGCCUCCACUUCACGCCUCUCCCUUGUCUCCUUUCCCUAG

>hsa-mir-6781

AACCCCGGGCCGGAGGUCAAGGGCGUCGCUUCUCCCUAAUGUUGCCUCUUUUCCACGGCCUCAG

>hsa-mir-6782

UGGGGUAGGGGUGGGGGAAUUCAGGGGUGUCGAACUCAUGGCUGCCACCUUUGUGUCCCCAUCCUGCAG

>hsa-mir-6783

CCUGUUAGGGGAAAAGUCCUGAUCCGGGAACCCACAGCCCCGUUCCUGGGCUUCUCCUCUGUAG

>hsa-mir-6785

CUCCCUGGGAGGGCGUGGAUGAUGGUGGGAGAGGAGCCCCACUGUGGAAGUCUGACCCCCACAUCGCCCCACCUUCCCCAG

>hsa-mir-6786

GCCGGGUGGGGCGGGGCGGCCUCAGGAGGGGCCCAGCUCCCCUGGAUGUGCUGCGGUGGGGCCGGAGGGGCGUCACGUGCACCCAAGUGACGCCCCUUCUGAUUCUGCCUCAG

>hsa-mir-6787

UCGGCUGGCGGGGGUAGAGCUGGCUGCAGGCCCGGCCCCUCUCAGCUGCUGCCCUCUCCAG

>hsa-mir-6788

GACGGCUGGGAGAAGAGUGGUGAAGAAGAGUAUUGAUUGUGCUGUUCGCCACUUCCCUCCCUGCAG

>hsa-mir-6789

CGAGGUAGGGGCGUCCCGGGCGCGCGGGCGGGUCCCAGGCUGGGCCCCUCGGAGGCCGGGUGCUCACUGCCCCGUCCCGGCGCCCGUGUCUCCUCCAG

>hsa-mir-6790

GUGAGUGUGGAUUUGGCGGGGUUCGGGGGUUCCGACGGCGACCUCGGCGACCCCUCACUCACC

>hsa-mir-6791

CCAGACCCCUGGGGCUGGGCAGGCGGAAAGAGGUCUGAACUGCCUCUGCCUCCUUGGUCUCCGGCAG

>hsa-mir-6793

GUCACUGUGGGUUCUGGGUUGGGGUGAUACACAAGCCUGACCCUCCCCAACCCCUGCCCGCAG

>hsa-mir-6795

AGGGUUGGGGGGACAGGAUGAGAGGCUGUCUUCAUUCCCUCUUGACCACCCCUCGUUUCUUCCCCCAG

>hsa-mir-6796

UUACCUUGUGGGGUUGGAGAGCUGGCUGGUCCAGCCCCUCAGAAGCUCUCCCCUCCCCGCAG

>hsa-mir-6797

CAGCCAGGAGGGAAGGGGCUGAGAACAGGACCUGUGCUCACUGGGGCCUGCAUGACCCUUCCCUCCCCACAG

>hsa-mir-6798

GGCAGCCAGGGGGAUGGGCGAGCUUGGGCCCAUUCCUUUCCUUACCCUACCCCCCAUCCCCCUGUAG

>hsa-mir-6799

GAGGAGGGGAGGUGUGCAGGGCUGGGGUCACUGACUCUGCUUCCCCUGCCCUGCAUGGUGUCCCCACAG

>hsa-mir-6800

ACCUGUAGGUGACAGUCAGGGGCGGGGUGUGGUGGGGCUGGGGCUGGCCCCCUCCUCACACCUCUCCUGGCAUCGCCCCCAG

>hsa-mir-6801

UGGCCUGGUCAGAGGCAGCAGGAAAUGAGAGUUAGCCAGGAGCUUUGCAUACUCACCCCUGCCACUCACUGGCCCCCAG

>hsa-mir-6802

GAGGGCUAGGUGGGGGGCUUGAAGCCCCGAGAUGCCUCACGUCUUCACCCCUCUCACCUAAGCAG

>hsa-mir-6803

CUCCUCUGGGGGUGGGGGGCUGGGCGUGGUGGACAGCGAUGCAUCCCUCGCCUUCUCACCCUCAG

>hsa-mir-6804

GGAUGUGAGGGUGUCAGCAGGUGACGGUGGGGGCCACGCUGACAGCCGCACCUGCCUCUCACCCACAG

>hsa-mir-6805

UGGCCUAGGGGGCGGCUUGUGGAGUGUAUGGGCUGAGCCUUGCUCUGCUCCCCCGCCCCCAG

>hsa-mir-6806

UGCUCUGUAGGCAUGAGGCAGGGCCCAGGUUCCAUGUGAUGCUGAAGCUCUGACAUUCCUGCAG

>hsa-mir-6807

GUGAGCCAGUGGAAUGGAGAGGCUGUGGGCAGGGGGAGAUGUGAAGGAAAGAACUAGGACCCAUUCAUCCACUGCAUUCCUGCUUGGCCCAG

>hsa-mir-6808

GGGGCCAGGCAGGGAGGUGGGACCAUGGGGGCCUUGCUGUGUGACCACCGUUCCUGCAG

>hsa-mir-6810

CUGGGAUGGGGACAGGGAUCAGCAUGGCACAGAUCCAAUACCUUCUGUCCCCUGCUCCCUUGUUCCCCAG

>hsa-mir-6812

UGAGGAUGGGGUGAGAUGGGGAGGAGCAGCCAGUCCUGUCUCACCGCUCUUCCCCUGACCCCAG

>hsa-mir-6813

GUAGGCAGGGGCUGGGGUUUCAGGUUCUCAGUCAGAACCUUGGCCCCUCUCCCCAG

>hsa-mir-6815

CACUGUAGGUGGCGCCGGAGGAGUCAUUUCCCAUCACUAAUGGCUUCUCUUGCACACCCAG

>hsa-mir-6816

CCGAGUGGGGCGGGGCAGGUCCCUGCAGGGACUGUGACACUGAAGGACCUGCACCUUCGCCCACAG

>hsa-mir-6817

AGGAUUCUGCCAUAGGAAGCUUGGAGUGGAACUGACCUGCCCCCUUUCUCUCUGACUCCAUGGCAG

>hsa-mir-6818

CUAUUUUGUGUGAGUACAGAGAGCAUCUGAAUGGGUACAGUUGUUGUCUCUUGUUCCUCACACAG

>hsa-mir-6819

GAGGGUUGGGGUGGAGGGCCAAGGAGCUGGGUGGGGUGCCAAGCCUCUGUCCCCACCCCAG

>hsa-mir-6820

CCUUCUGCGGCAGAGCUGGGGUCACCAGCCCUCAUGUACUUGUGACUUCUCCCCUGCCACAG

>hsa-mir-6821

GUGCGUGGUGGCUCGAGGCGGGGGUGGGGGCCUCGCCCUGCUUGGGCCCUCCCUGACCUCUCCGCUCCGCACAG

>hsa-mir-6822

UGGCCCAGGGAACCAGUUGGGGCUUCCGCUCUGCAGAGGCUCUAACUGGCUUUCCCUGCAG

>hsa-mir-6823

CAAGGUCAGGGUUGGUAGGGGUUGCUGUUGCUGUGAAAGCUGAGCCUCUCCUUCCCUCCAG

>hsa-mir-6824

GAGGUGUAGGGGAGGUUGGGCCAGGGAUGCCUUCACUGUGUCUCUCUGGUCUUGCCACCCCAG

>hsa-mir-6825

GGGCAUGGGGAGGUGUGGAGUCAGCAUGGGGCUAGGAGGCCCCGCGCUGACCCGCCUUCUCCGCAG

>hsa-mir-6826

CUUGGUCAAUAGGAAAGAGGUGGGACCUCCUGGCUUUUCCUCUGCAGCAUGGCUCGGACCUAGUGCAAUGUUUAAGCUCCCCUCUCUUUCCUGUUCAG

>hsa-mir-6832

GGUGGAGUAGAGAGGAAAAGUUAGGGUCAGUGGCAGAGCCAGGCAGAUGCUGACCCUUUUUCUCUUUCCCAG

>hsa-mir-6833

AAACGGUGUGGAAGAUGGGAGGAGAAAAAUCCCUGUUAACUUUCUCUCUCCACUUCCUCAG

>hsa-mir-6835

UGAUGAGGGGGUAGAAAGUGGCUGAAGCGAGAUGUUUGUCUAAAAGCACUUUUCUGUCUCCCAG

>hsa-mir-6837

GUGGGACCAGGGCCAGCAGGGAAUGUCAGGGCCACCCCUGACCUUCACUGUGACUCUGCUGCAG

>hsa-mir-6838

CAGGGAAGCAGCAGUGGCAAGACUCCUAGGUCACGGAAGUCCUGCUUCUGUUGCAG

>hsa-mir-6839

UGUAGUCUGGAUUGAAGAGACGACCCAAGCAGGCUUUGUGUGAGCAGUGAGGCUAUUUAUUCACUUGGGUGCGAGCUCACACGAAGCCUGCUUGGGUUUUCUCUUCAAUCCAG

>hsa-mir-6840

UGACCACCCCCGGGCAAAGACCUGCAGAUCCCCUGUUAGAGACGGGCCCAGGACUUUGUGCGGGGUGCCCA

>hsa-mir-6842

AGCCCUGGGGGUGGUCUCUAGCCAAGGCUCUGGGGUCUCACCCUUGGCUGGUCUCUGCUCCGCAG

>hsa-mir-6844

GAACUUAAGAAUUUUGUAGAAAUCAAGCUAUUUGCUAAAAGUUCUUUGUUUUUAAUUCACAG

>hsa-mir-6846

CAGGCUGGGGGCUGGAUGGGGUAGAGUAGGAGAGCCCACUGACCCCUUCUGUCUCCCUAG

>hsa-mir-6847

GACCCACAGAGGACAGUGGAGUGUGAGCUGGAAGGAGUGGGCCUGGCUCAUGUGUCUGUCCUCUUCCAG

>hsa-mir-6848

GUCCCUGGGGGCUGGGAUGGGCCAUGGUGUGCUCUGAUCCCCCUGUGGUCUCUUGGCCCCCAGGAACUCC

>hsa-mir-6851

CAGGGAGGAGGUGGUACUAGGGGCCAGCAACCUGAUUACCCCUCUUUGGCCCUUUGUACCCCUCCAG

>hsa-mir-6852

UGCUGCCCUGGGGUUCUGAGGACAUGCUCUGACUCCCCUGAUGUCCUCUGUUCCUCAGGUGCUGGG

>hsa-mir-6853

GGGAAAGCGUGGGAUGUCCAUGAAGUCAGGUGAUGGUGAUAAGGUCAAGGCCUGUUCAUUGGAACCCUGCGCAG

>hsa-mir-6854

AAAGCAAGCUCAGGUUUGAGAACUGCUGAUGUCAUCAGUCAUAACUUCUGCGUUUCUCCUCUUGAGCAG

>hsa-mir-6855

GCUGCUUGGGGUUUGGGGUGCAGACAUUGCCAGAGGAUGGGCAGCAGACUGACCUUCAACCCCACAG

>hsa-mir-6856

UGGAAAAGAGAGGAGCAGUGGUGCUGUGGCAGUGGCAGAGGUCGCUACAGCCCUGUGAUCUUUCCAG

>hsa-mir-6857

GCUUGUUGGGGAUUGGGUCAGGCCAGUGUUCAAGGGCCCCUCCUCUAGUACUCCCUGUUUGUGUUCUGCCACUGACUGAGCUUCUCCCCACAG

>hsa-mir-6858

GUGAGGAGGGGCUGGCAGGGACCCCUCCAAGUUGGGGACGGCAGCCAGCCCCUGCUCACCCCUCGCC

>hsa-mir-6859-1

UGUGGGAGAGGAACAUGGGCUCAGGACAGCGGGUGUCAGCUUGCCUGACCCCCAUGUCGCCUCUGUAG

>hsa-mir-6859-2

UGUGGGAGAGGAACAUGGGCUCAGGACAGCGGGUGUCAGCUUGCCUGACCCCCAUGUCGCCUCUGUAG

>hsa-mir-6859-3

UGUGGGAGAGGAACAUGGGCUCAGGACAGCGGGUGUCAGCUUGCCUGACCCCCAUGUCGCCUCUGUAG

>hsa-mir-6859-4

UGUGGGAGAGGAACAUGGGCUCAGGACAGCGGGUGUCAGCUUGCCUGACCCCCAUGUCGCCUCUGUAG

>hsa-mir-6860

GUUAAGCAUUGGGGAGUUUGGAGUCGGUGGGUGGAGCCAAACUGGGCAGGGCUGUGGUGAGUGAGU

>hsa-mir-6862-1

CGAAGCGGGCAUGCUGGGAGAGACUUUGUGAUUUGUCUCCAAAGCCUCACCCAGCUCUCUGGCCCUCUAG

>hsa-mir-6862-2

CGAAGCGGGCAUGCUGGGAGAGACUUUGUGAUUUGUCUCCAAAGCCUCACCCAGCUCUCUGGCCCUCUAG

>hsa-mir-6863

AUUUAUGAGGCAGCAGAGUCUACAAGUAAAUCAUGAAUCCAGUUGAAAAUGUUAAUGAGGCCAUAGACGUGGUGAAGGAUUGAGUGACCU

>hsa-mir-6865

AUCCAUAGGUGGCAGAGGAGGGACUUCAGAUGCUUCAUGACACCCUCUUUCCCUACCGCCUACAG

>hsa-mir-6866

CCAUUUUAGAGGCUGGAAUAGAGAUUCUUGAGGCUUGGAAGAGUAAGGAUCCCUUUAUCUGUCCUCUAG

>hsa-mir-6867

CCCGGUGUGUGUGUAGAGGAAGAAGGGAAGCUGGGAACCUGACUGCCUCUCCCUCUUUACCCACUAG

>hsa-mir-6868

CCAGGACUGGCAGAACACUGAAGCAGCAGGCACCUGCUUCCUUCUGUUGUCUGUGCAG

>hsa-mir-6869

GUGAGUAGUGGCGCGCGGCGGCUCGGAGUACCUCUGCCGCCGCGCGCAUCGGCUCAGCAUGC

>hsa-mir-6870

CAAGGUGGGGGAGAUGGGGGUUGAACUUCAUUUCUCAUGCUCAUCCCCAUCUCCUUUCAG

>hsa-mir-6873

CCCAGCAGAGGGAAUACAGAGGGCAAUCAGGACUGGGUCAUUCUCUCUGUCUUUCUCUCUCAG

>hsa-mir-6874

GCCACAUGGAGCUGGAACCAGAUCAGGCUUUAAUGUUUGAAGUAAUGUCAGUUCUGCUGUUCUGACUCUAG

>hsa-mir-6875

GAGUCUGAGGGACCCAGGACAGGAGAAGGCCUAUGGUGAUUUGCAUUCUUCCUGCCCUGGCUCCAUCCUCAG

>hsa-mir-6876

AGUUGCAGGAAGGAGACAGGCAGUUCAGGAGGUGGCACUGCUGUGUGUGAGCUGUCUGUGUUUUCCUUCUCAG

>hsa-mir-6877

AGUUCAGGGCCGAAGGGUGGAAGCUGCUGGUGCUCAUCUCAGCCUCUGCCCUUGGCCUCCCCAG

>hsa-mir-6878

AUGAGAGGGAGAAAGCUAGAAGCUGAAGAUUCUGAAAAUCACUAACUGGCCUCUUCUUUCUCCUAG

>hsa-mir-6881

UGCACUGGGGUAAGGAUAGGAGGGUCAGGUCUGCAGCCUUGUAUCUGCUGAUCCUCUUUCGUCCUUCCCACUCCAG

>hsa-mir-6882

GGCUUUACAAGUCAGGAGCUGAAGCAGCUGGAAUUCAAGCCCUGCUGCCUCUCCUCUUGCCUGCAG

>hsa-mir-6883

CAGACAGGGAGGGUGUGGUAUGGAUGUGUUGACCCCUGAAGUGGUUCUGAUGACCUUUCCCUAUCUCACUCUCCUCAG

>hsa-mir-6884

CCCGCAGAGGCUGAGAAGGUGAUGUUGGCUCAAGAAAGGGAGAUAGAUGGUAGCCCAUCACCUUUCCGUCUCCCCUAG

>hsa-mir-6885

CCUGGAGGGGGGCACUGCGCAAGCAAAGCCAGGGACCCUGAGAGGCUUUGCUUCCUGCUCCCCUAG

>hsa-mir-6886

CUUGGCCCGCAGGUGAGAUGAGGGCUCCUGGCGCUGAUGCCCUUCUCUCCUCCUGCCUCAG

>hsa-mir-6891

GUAAGGAGGGGGAUGAGGGGUCAUAUCUCUUCUCAGGGAAAGCAGGAGCCCUUCAGCAGGGUCAGGGCCCCUCAUCUUCCCCUCCUUUCCCAG

>hsa-mir-6892

GUAAGGGACCGGAGAGUAGGAAAAGCAGGGCUCAGGGCCAGAGAGACUGGGCAUAGAACUAAGGAGGAUGGUGUCCUCCUGACUGCAUCUCUCUUCCCUCUCCCACCCCUUGCAG

>hsa-mir-6893

CCGGGCAGGCAGGUGUAGGGUGGAGCCCACUGUGGCUCCUGACUCAGCCCUGCUGCCUUCACCUGCCAG

>hsa-mir-6894

CAAGAAGGAGGAUGGAGAGCUGGGCCAGACAUGCUCUUGCCUGCCCUCUUCCUCCAG

>hsa-mir-6895

CAGCUCAGGGCCAGGCACAGAGUAAGCAUCAAUAGCAUUGGCAAGUUGAACUGAGCUGUCUCUCGCCCUUGGCCUUAG

>hsa-mir-7-1

UUGGAUGUUGGCCUAGUUCUGUGUGGAAGACUAGUGAUUUUGUUGUUUUUAGAUAACUAAAUCGACAACAAAUCACAGUCUGCCAUAUGGCACAGGCCAUGCCUCUACAG

>hsa-mir-7-2

CUGGAUACAGAGUGGACCGGCUGGCCCCAUCUGGAAGACUAGUGAUUUUGUUGUUGUCUUACUGCGCUCAACAACAAAUCCCAGUCUACCUAAUGGUGCCAGCCAUCGCA

>hsa-mir-7-3

AGAUUAGAGUGGCUGUGGUCUAGUGCUGUGUGGAAGACUAGUGAUUUUGUUGUUCUGAUGUACUACGACAACAAGUCACAGCCGGCCUCAUAGCGCAGACUCCCUUCGAC

>hsa-mir-708

AACUGCCCUCAAGGAGCUUACAAUCUAGCUGGGGGUAAAUGACUUGCACAUGAACACAACUAGACUGUGAGCUUCUAGAGGGCAGGGA

>hsa-mir-7108

GUGUGGCCGGCAGGCGGGUGGGCGGGGGCGGCCGGUGGGAACCCCGCCCCGCCCCGCGCCCGCACUCACCCGCCCGUCUCCCCACAG

>hsa-mir-7109

GUCUCCUGGGGGGAGGAGACCCUGCUCUCCCUGGCAGCAAGCCUCUCCUGCCCUUCCAGAUUAGC

>hsa-mir-711

ACUGACUUUGAGUCUCUCCUCAGGGUGCUGCAGGCAAAGCUGGGGACCCAGGGAGAGACGUAAGUGAGGGGAGAUG

>hsa-mir-7110

GGGGCUGGGGGUGUGGGGAGAGAGAGUGCACAGCCAGCUCAGGGAUUAAAGCUCUUUCUCUCUCUCUCUCUCCCACUUCCCUGCAG

>hsa-mir-7111

CUGGGGGAGGAAGGACAGGCCAUCUGCUAUUCGUCCACCAACCUGACUUGAUCCUCUCUUCCCUCCUCCCAG

>hsa-mir-7113

CUCCAGGGAGACAGUGUGUGAGGCCUCUUGCCAUGGCCUCCCUGCCCGCCUCUCUGCAG

>hsa-mir-7114

UCCGCUCUGUGGAGUGGGGUGCCUGUCCCCUGCCACUGGGUGACCCACCCCUCUCCACCAG

>hsa-mir-7151

GAUCCAUCUCUGCCUGUAUUGGCUUGGAUUCUGCAAAGCCUACAGGCUGGAAUGGGCUCA

>hsa-mir-7152

UUUCCUGUCCUCCAACCAGACCAUGCCACAUCCGUCUGGUCCUGGACAGGAGGC

>hsa-mir-7160

UGCUGAGGUCCGGGCUGUGCCCCGUACCGGACAGGGCCCUGGCUUUAGCAGA

>hsa-mir-744

UUGGGCAAGGUGCGGGGCUAGGGCUAACAGCAGUCUUACUGAAGGUUUCCUGGAAACCACGCACAUGCUGUUGCCACUAACCUCAACCUUACUCGGUC

>hsa-mir-758

GCCUGGAUACAUGAGAUGGUUGACCAGAGAGCACACGCUUUAUUUGUGCCGUUUGUGACCUGGUCCACUAACCCUCAGUAUCUAAUGC

>hsa-mir-760

GGCGCGUCGCCCCCCUCAGUCCACCAGAGCCCGGAUACCUCAGAAAUUCGGCUCUGGGUCUGUGGGGAGCGAAAUGCAAC

>hsa-mir-762

GGCCCGGCUCCGGGUCUCGGCCCGUACAGUCCGGCCGGCCAUGCUGGCGGGGCUGGGGCCGGGGCCGAGCCCGCGGCGGGGCC

>hsa-mir-765

UUUAGGCGCUGAUGAAAGUGGAGUUCAGUAGACAGCCCUUUUCAAGCCCUACGAGAAACUGGGGUUUCUGGAGGAGAAGGAAGGUGAUGAAGGAUCUGUUCUCGUGAGCCUGAA

>hsa-mir-766

GCAUCCUCAGGACCUGGGCUUGGGUGGUAGGAGGAAUUGGUGCUGGUCUUUCAUUUUGGAUUUGACUCCAGCCCCACAGCCUCAGCCACCCCAGCCAAUUGUCAUAGGAGC

>hsa-mir-767

GCUUUUAUAUUGUAGGUUUUUGCUCAUGCACCAUGGUUGUCUGAGCAUGCAGCAUGCUUGUCUGCUCAUACCCCAUGGUUUCUGAGCAGGAACCUUCAUUGUCUACUGC

>hsa-mir-769

GCCUUGGUGCUGAUUCCUGGGCUCUGACCUGAGACCUCUGGGUUCUGAGCUGUGAUGUUGCUCUCGAGCUGGGAUCUCCGGGGUCUUGGUUCAGGGCCGGGGCCUCUGGGUUCCAAGC

>hsa-mir-7702

CUUAGACUGCCAGACUCCCUGAGGACAUGCAGUUUCAGGGAGUCUGGUAGUCUAAGUGU

>hsa-mir-7703

UUAGGGGAGGUGGAUGAGUGGGGGCAGGCAGGGGAUUGGCUAAGAACCUGACUCUUGCACUCUGGCCUUCUCCCAGG

>hsa-mir-7704

CGGGGUCGGCGGCGACGUGCUCAGCUUGGCACCCAAGUUCUGCCGCUCCGACGCCCGGC

>hsa-mir-7705

AAUAGCUCAGAAUGUCAGUUCUGUUUUAAGUAACAGAAUUGAUAACUGAGCAAGGAA

>hsa-mir-7706

UGGAGCUGUGUGCAGGGCCAGCGCGGAGCCCGAGCAGCCGCGGUGAAGCGCCUGUGCUCUGCCGAGA

>hsa-mir-7845

GCAAGGGACAGGGAGGGUCGUGGCGACACUCGCGCCAGCUCCCGGGACGGCUGGGCUCGGGCUGGUCGCCGACCUCCGACCCUCCACUAGAUGCCUGGC

>hsa-mir-7846

GCCCCGCCGCCUGGCCUCUGGCCCGCUGGGGCGCGGGCUUUCGCUUUCAGUCGAGGGCUAGCGAGCGCAGCGGAGCCUGGAGAGAAGGCGCUGGGC

>hsa-mir-7848

GCUGGGGCUGGGUGGGUGUGGCAGGCCCACCUUGGGUAUGCAAAGCUCUGACAGUGUUUCACUUGCUACCCUCGGUCUGCUUACCACACUCCCAGUUCUGC

>hsa-mir-7851

UGGCUCACUGCAGCCUCCCCGCCCCCUCAGGUGAUCCUCCCACCUCAUCCUCCCAAGUAGCUGGGAAUACAGGUGUGUGCCACCAUGCCUCUACAAGCUACCUGGGAGACUGAGGUUGGAAGAUUGCUUGAGCCUAGGAGGUCGAGGCGACAGUGAGCCA

>hsa-mir-7852

UAAAUGCCUUUGACUACUACAUACAUAUGCCUACUACUACUUUCAUAUGUAGGCAUAUGUAUGUAGUAGUCAAAGGCAUUUA

>hsa-mir-7854

UUCCUUCCAUCUCCAUCACCUUGAGCAUCUUCUGGGCAGCUGAGGUGACCGCAGAUGGGAAGGAA

>hsa-mir-7974

GCUCGGCCCCCACAGCGAAACGGCCGCCUAAACCACCCAGGCCUUAUGGCUUCAUAGGCUGUGAUGCUCUCCUGAGCCC

>hsa-mir-7976

UGCCCUGAGACUUUUGCUCUAAUAAUUUAUUCUAAUAAUAAUUUAGAUCAAAAGCCUCAGGGCAGA

>hsa-mir-7977

UUCCCAGCCAACGCACCAAAAAUGAUAUGGGUCUGUUGUCUGGAGAAAC

>hsa-mir-8072

GCGUCAAGAUGGCGGCGGGGAGGUAGGCAGAGCAGGACGCCGCUGCUGCCGCCGCCACCGCCGCCUCCGCUCCAGUCGCC

>hsa-mir-8075

CCUUGCUGAUGGCAGAUGUCGGAUCUGCCUCGCUUAUACGUGCCCUUGCUGAUGGCAGAUGUCGGGUCUGCCUCGCUUAU

>hsa-mir-8085

CUAGGAGGGAUGGGAGAGAGGACUGUGAGGCAUGGGUGGCUCUAUGGUCACGCCCAUCUUCCUAC

>hsa-mir-8086

UCCACUAGUCUGCUAGUCUGGACUGAUAUGGUUAGCUUUUUUUUUUUUUUGAGAUGGAGUCUGGCUCUGUUGCCCAGGCUGGAGUACAGUGGC

>hsa-mir-8485

UCUGUGAUAUACGUGUGUGUGUGUGUGUAUAUAGCAUAUGUGUAUACAUACACACACACACACACACACACACACACACACACACACGUAU

>hsa-mir-873

GUGUGCAUUUGCAGGAACUUGUGAGUCUCCUAUUGAAAAUGAACAGGAGACUGAUGAGUUCCCGGGAACACCCACAA

>hsa-mir-874

UUAGCCCUGCGGCCCCACGCACCAGGGUAAGAGAGACUCUCGCUUCCUGCCCUGGCCCGAGGGACCGACUGGCUGGGC

>hsa-mir-875

UUAGUGGUACUAUACCUCAGUUUUAUCAGGUGUUCUUAAAAUCACCUGGAAACACUGAGGUUGUGUCUCACUGAAC

>hsa-mir-877

GUAGAGGAGAUGGCGCAGGGGACACGGGCAAAGACUUGGGGGUUCCUGGGACCCUCAGACGUGUGUCCUCUUCUCCCUCCUCCCAG

>hsa-mir-885

CCGCACUCUCUCCAUUACACUACCCUGCCUCUUCUCCAUGAGAGGCAGCGGGGUGUAGUGGAUAGAGCACGGGU

>hsa-mir-887

GUGCAGAUCCUUGGGAGCCCUGUUAGACUCUGGAUUUUACACUUGGAGUGAACGGGCGCCAUCCCGAGGCUUUGCACAG

>hsa-mir-888

GGCAGUGCUCUACUCAAAAAGCUGUCAGUCACUUAGAUUACAUGUGACUGACACCUCUUUGGGUGAAGGAAGGCUCA

>hsa-mir-889

GUGCUUAAAGAAUGGCUGUCCGUAGUAUGGUCUCUAUAUUUAUGAUGAUUAAUAUCGGACAACCAUUGUUUUAGUAUCC

>hsa-mir-891a

CCUUAAUCCUUGCAACGAACCUGAGCCACUGAUUCAGUAAAAUACUCAGUGGCACAUGUUUGUUGUGAGGGUCAAAAGA

>hsa-mir-892a

GCAGUGCCUUACUCAGAAAGGUGCCAGUCACUUACACUACAUGUCACUGUGUCCUUUCUGCGUAGAGUAAGGCUC

>hsa-mir-892b

UGCAAUGCCCUACUCAGAAAGGUGCCAUUUAUGUAGAUUUUAUGUCACUGGCUCCUUUCUGGGUAGAGCAAGGCUCA

>hsa-mir-9-1

CGGGGUUGGUUGUUAUCUUUGGUUAUCUAGCUGUAUGAGUGGUGUGGAGUCUUCAUAAAGCUAGAUAACCGAAAGUAAAAAUAACCCCA

>hsa-mir-9-2

GGAAGCGAGUUGUUAUCUUUGGUUAUCUAGCUGUAUGAGUGUAUUGGUCUUCAUAAAGCUAGAUAACCGAAAGUAAAAACUCCUUCA

>hsa-mir-9-3

GGAGGCCCGUUUCUCUCUUUGGUUAUCUAGCUGUAUGAGUGCCACAGAGCCGUCAUAAAGCUAGAUAACCGAAAGUAGAAAUGAUUCUCA

>hsa-mir-92a-1

CUUUCUACACAGGUUGGGAUCGGUUGCAAUGCUGUGUUUCUGUAUGGUAUUGCACUUGUCCCGGCCUGUUGAGUUUGG

>hsa-mir-92a-2

UCAUCCCUGGGUGGGGAUUUGUUGCAUUACUUGUGUUCUAUAUAAAGUAUUGCACUUGUCCCGGCCUGUGGAAGA

>hsa-mir-92b

CGGGCCCCGGGCGGGCGGGAGGGACGGGACGCGGUGCAGUGUUGUUUUUUCCCCCGCCAAUAUUGCACUCGUCCCGGCCUCCGGCCCCCCCGGCCC

>hsa-mir-93

CUGGGGGCUCCAAAGUGCUGUUCGUGCAGGUAGUGUGAUUACCCAACCUACUGCUGAGCUAGCACUUCCCGAGCCCCCGG

>hsa-mir-933

ACUUGGGUCAGUUCAGAGGUCCUCGGGGCGCGCGUCGAGUCAGCCGUGUGCGCAGGGAGACCUCUCCCACCCACAGU

>hsa-mir-935

GGCGGGGGCGCGGGCGGCAGUGGCGGGAGCGGCCCCUCGGCCAUCCUCCGUCUGCCCAGUUACCGCUUCCGCUACCGCCGCCGCUCCCGCU

>hsa-mir-937

AGCACUGCCCCCGGUGAGUCAGGGUGGGGCUGGCCCCCUGCUUCGUGCCCAUCCGCGCUCUGACUCUCUGCCCACCUGCAGGAGCU

>hsa-mir-939

UGUGGGCAGGGCCCUGGGGAGCUGAGGCUCUGGGGGUGGCCGGGGCUGACCCUGGGCCUCUGCUCCCCAGUGUCUGACCGCG

>hsa-mir-940

GUGAGGUGUGGGCCCGGCCCCAGGAGCGGGGCCUGGGCAGCCCCGUGUGUUGAGGAAGGAAGGCAGGGCCCCCGCUCCCCGGGCCUGACCCCAC

>hsa-mir-941-1

UGUGGACAUGUGCCCAGGGCCCGGGACAGCGCCACGGAAGAGGACGCACCCGGCUGUGUGCACAUGUGCCCA

>hsa-mir-941-2

UGUGCACAUGUGCCCAGGGCCCGGGACAGCGCCACGGAAGAGGACGCACCCGGCUGUGUGCACAUGUGCCCA

>hsa-mir-941-3

UGUGCACAUGUGCCCAGGGCCCGGGACAGCGCCACGGAAGAGGACGCACCCGGCUGUGUGCACAUGUGCCCA

>hsa-mir-941-4

UGUGCACAUGUGCCCAGGGCCCGGGACAGCGCCACGGAAGAGGACGCACCCGGCUGUGUGCACAUGUGCCCA

>hsa-mir-941-5

UGUGCACAUGUGCCCAGGGCCCGGGACAGCGCCACGGAAGAGGACGCACCCGGCUGUGUGCACAUGUGCCCA

>hsa-mir-942

AUUAGGAGAGUAUCUUCUCUGUUUUGGCCAUGUGUGUACUCACAGCCCCUCACACAUGGCCGAAACAGAGAAGUUACUUUCCUAAU

>hsa-mir-943

GGGACGUUCUGAGCUCGGGGUGGGGGACGUUUGCCGGUCACUGCUGCUGGCGCCCUGACUGUUGCCGUCCUCCAGCCCCACUCAAAGGCAUCCC

>hsa-mir-944

GUUCCAGACACAUCUCAUCUGAUAUACAAUAUUUUCUUAAAUUGUAUAAAGAGAAAUUAUUGUACAUCGGAUGAGCUGUGUCUGGGAU

>hsa-mir-95

AACACAGUGGGCACUCAAUAAAUGUCUGUUGAAUUGAAAUGCGUUACAUUCAACGGGUAUUUAUUGAGCACCCACUCUGUG

>hsa-mir-96

UGGCCGAUUUUGGCACUAGCACAUUUUUGCUUGUGUCUCUCCGCUCUGAGCAAUCAUGUGCAGUGCCAAUAUGGGAAA

>hsa-mir-98

AGGAUUCUGCUCAUGCCAGGGUGAGGUAGUAAGUUGUAUUGUUGUGGGGUAGGGAUAUUAGGCCCCAAUUAGAAGAUAACUAUACAACUUACUACUUUCCCUGGUGUGUGGCAUAUUCA

>hsa-mir-9901

GGAGAGGGUGGGGGAGCCCGUCCCGGUCGCCGCGGUUCGCCGCCGCCCCUGGUGGCGGUCCGGCGACCGGCCCACCAUCGCUCCAGCGCCCCUCCU

>hsa-mir-9902-1

GCAGGGAAAGGGAACCCAGAAAUCUGGUAUGCCAGCAAAGAGAGUAAGAACUUCUGACAAGCCAGGCUUCUGGUCUCUCUCUCUCUGUCUCUC

>hsa-mir-9902-2

GCAGGGAAAGGGAACCCAGAAAUCUGGUAUGCCAGCAAAGAGAGUAAGAACUUCUGACAAGCCAGGCUUCUGGUCUCUCUCUCUCUGUCUCUC

>hsa-mir-9903

CCAGCUCUGGUUCCCCAGCCUACUGGAGGAUAAGAGGAUAUAAAGGUCUCUUAUCCUCCAGUAGACUAGGGAGCCAGAGCUGGUAAUAAAAGUC

>hsa-mir-9985

CCCAUGUUCAUUGAGCUUUAUUCACAGUGGCUAAGCUAUGGAAUCCAUCCAAGUGUUCACUGAUGGAUAAAUGGAUACAGAAAAUGUUUAUAUAUAUAUAUAUAAUAUU

>hsa-mir-99a

CCCAUUGGCAUAAACCCGUAGAUCCGAUCUUGUGGUGAAGUGGACCGCACAAGCUCGCUUCUAUGGGUCUGUGUCAGUGUG

>hsa-mir-99b

GGCACCCACCCGUAGAACCGACCUUGCGGGGCCUUCGCCGCACACAAGCUCGUGUCUGUGGGUCCGUGUC
