# Supplementary material for: Identification and characterization of non-coding RNA networks in infected macrophages revealing the pathogenesis of F. nucleatum-associated diseases
Source: BMC Genomics. 2022 Dec 13;23:826. doi: 10.1186/s12864-022-09052-z (PMC9749367; doi:10.1186/s12864-022-09052-z)
Supplement: Supplementary file 13 — Additional file 13: Supplementary txt2. miRNA mature. [file 12864_2022_9052_MOESM13_ESM.docx]

>hsa-let-7a-5p

UGAGGUAGUAGGUUGUAUAGUU

>hsa-let-7a-3p

CUAUACAAUCUACUGUCUUUC

>hsa-let-7a-2-3p

CUGUACAGCCUCCUAGCUUUCC

>hsa-let-7b-5p

UGAGGUAGUAGGUUGUGUGGUU

>hsa-let-7b-3p

CUAUACAACCUACUGCCUUCCC

>hsa-let-7c-5p

UGAGGUAGUAGGUUGUAUGGUU

>hsa-let-7c-3p

CUGUACAACCUUCUAGCUUUCC

>hsa-let-7d-5p

AGAGGUAGUAGGUUGCAUAGUU

>hsa-let-7d-3p

CUAUACGACCUGCUGCCUUUCU

>hsa-let-7e-5p

UGAGGUAGGAGGUUGUAUAGUU

>hsa-let-7e-3p

CUAUACGGCCUCCUAGCUUUCC

>hsa-let-7f-5p

UGAGGUAGUAGAUUGUAUAGUU

>hsa-let-7f-1-3p

CUAUACAAUCUAUUGCCUUCCC

>hsa-let-7f-2-3p

CUAUACAGUCUACUGUCUUUCC

>hsa-let-7g-5p

UGAGGUAGUAGUUUGUACAGUU

>hsa-let-7g-3p

CUGUACAGGCCACUGCCUUGC

>hsa-let-7i-5p

UGAGGUAGUAGUUUGUGCUGUU

>hsa-let-7i-3p

CUGCGCAAGCUACUGCCUUGCU

>hsa-miR-1-3p

UGGAAUGUAAAGAAGUAUGUAU

>hsa-miR-1-5p

ACAUACUUCUUUAUAUGCCCAU

>hsa-miR-100-5p

AACCCGUAGAUCCGAACUUGUG

>hsa-miR-100-3p

CAAGCUUGUAUCUAUAGGUAUG

>hsa-miR-101-5p

CAGUUAUCACAGUGCUGAUGCU

>hsa-miR-101-3p

UACAGUACUGUGAUAACUGAA

>hsa-miR-101-2-5p

UCGGUUAUCAUGGUACCGAUGC

>hsa-miR-10392-5p

GCGCUUCGACGGGCUGGGCUGUG

>hsa-miR-10393-5p

AGAAUUCUCUUAUCCAACAUCAACA

>hsa-miR-10393-3p

UGGUCAGAUUUGAACUCUUCAA

>hsa-miR-10394-5p

UCUGCAGGUCCUGGUGAACGCCAU

>hsa-miR-10394-3p

UGGGCGCGCCGGGACUGUGAGAC

>hsa-miR-10395-3p

AUGUAUUCGUACUGUCUGAUG

>hsa-miR-10396a-5p

GGCGGGGCUCGGAGCCGGG

>hsa-miR-10396b-3p

GGCCCCGGGCCCUCGACCGGAC

>hsa-miR-10397-3p

CAUAGAUCUCGUCGCUUACUGGGA

>hsa-miR-10398-5p

UGGCUCCCUUCUCUCCGUCUG

>hsa-miR-10398-3p

GCCCGGAGAGCUGGGAGCCAG

>hsa-miR-10399-5p

AAUUACAGAUUGUCUCAGAGA

>hsa-miR-10399-3p

CUCUCGGACAAGCUGUAGGUC

>hsa-miR-103a-3p

AGCAGCAUUGUACAGGGCUAUGA

>hsa-miR-103a-1-5p

GGCUUCUUUACAGUGCUGCCUUG

>hsa-miR-103a-2-5p

AGCUUCUUUACAGUGCUGCCUUG

>hsa-miR-103b

UCAUAGCCCUGUACAAUGCUGCU

>hsa-miR-10401-5p

CGUGUGGGAAGGCGUGGGGU

>hsa-miR-10401-3p

ACCUCGCCGUCCCGCCCGCCG

>hsa-miR-10522-5p

AGAAGAAUUGGCCUACUCAGG

>hsa-miR-10527-5p

AAAGCAAAUGUUGGGUGAACGGC

>hsa-miR-106a-5p

AAAAGUGCUUACAGUGCAGGUAG

>hsa-miR-106a-3p

CUGCAAUGUAAGCACUUCUUAC

>hsa-miR-106b-5p

UAAAGUGCUGACAGUGCAGAU

>hsa-miR-106b-3p

CCGCACUGUGGGUACUUGCUGC

>hsa-miR-107

AGCAGCAUUGUACAGGGCUAUCA

>hsa-miR-10a-5p

UACCCUGUAGAUCCGAAUUUGUG

>hsa-miR-10a-3p

CAAAUUCGUAUCUAGGGGAAUA

>hsa-miR-10b-5p

UACCCUGUAGAACCGAAUUUGUG

>hsa-miR-10b-3p

ACAGAUUCGAUUCUAGGGGAAU

>hsa-miR-11399

UUCAGGUCUGGGGCUGAAACCU

>hsa-miR-11400

UCGGCUGUGUAUCUCUGUGUC

>hsa-miR-11401

UCACGUCUGCGGCUGUCACG

>hsa-miR-1179

AAGCAUUCUUUCAUUGGUUGG

>hsa-miR-1180-3p

UUUCCGGCUCGCGUGGGUGUGU

>hsa-miR-12114

CAGGUGGAGGUGUGAGGUC

>hsa-miR-12117

GAAGUGGAGCACAUCAGUGA

>hsa-miR-12136

GAAAAAGUCAUGGAGGCC

>hsa-miR-122-5p

UGGAGUGUGACAAUGGUGUUUG

>hsa-miR-122-3p

AACGCCAUUAUCACACUAAAUA

>hsa-miR-1226-5p

GUGAGGGCAUGCAGGCCUGGAUGGGG

>hsa-miR-1226-3p

UCACCAGCCCUGUGUUCCCUAG

>hsa-miR-1227-3p

CGUGCCACCCUUUUCCCCAG

>hsa-miR-1228-5p

GUGGGCGGGGGCAGGUGUGUG

>hsa-miR-1228-3p

UCACACCUGCCUCGCCCCCC

>hsa-miR-1229-3p

CUCUCACCACUGCCCUCCCACAG

>hsa-miR-122b-5p

UUUAGUGUGAUAAUGGCGUUUGA

>hsa-miR-122b-3p

AAACACCAUUGUCACACUCCAC

>hsa-miR-1233-3p

UGAGCCCUGUCCUCCCGCAG

>hsa-miR-1234-3p

UCGGCCUGACCACCCACCCCAC

>hsa-miR-1236-3p

CCUCUUCCCCUUGUCUCUCCAG

>hsa-miR-1237-3p

UCCUUCUGCUCCGUCCCCCAG

>hsa-miR-124-5p

CGUGUUCACAGCGGACCUUGAU

>hsa-miR-124-3p

UAAGGCACGCGGUGAAUGCCAA

>hsa-miR-1243

AACUGGAUCAAUUAUAGGAGUG

>hsa-miR-1244

AAGUAGUUGGUUUGUAUGAGAUGGUU

>hsa-miR-1246

AAUGGAUUUUUGGAGCAGG

>hsa-miR-1247-3p

CCCCGGGAACGUCGAGACUGGAGC

>hsa-miR-1248

ACCUUCUUGUAUAAGCACUGUGCUAAA

>hsa-miR-1249-5p

AGGAGGGAGGAGAUGGGCCAAGUU

>hsa-miR-1250-5p

ACGGUGCUGGAUGUGGCCUUU

>hsa-miR-1255a

AGGAUGAGCAAAGAAAGUAGAUU

>hsa-miR-1255b-5p

CGGAUGAGCAAAGAAAGUGGUU

>hsa-miR-1256

AGGCAUUGACUUCUCACUAGCU

>hsa-miR-125a-5p

UCCCUGAGACCCUUUAACCUGUGA

>hsa-miR-125a-3p

ACAGGUGAGGUUCUUGGGAGCC

>hsa-miR-125b-5p

UCCCUGAGACCCUAACUUGUGA

>hsa-miR-125b-1-3p

ACGGGUUAGGCUCUUGGGAGCU

>hsa-miR-125b-2-3p

UCACAAGUCAGGCUCUUGGGAC

>hsa-miR-126-5p

CAUUAUUACUUUUGGUACGCG

>hsa-miR-126-3p

UCGUACCGUGAGUAAUAAUGCG

>hsa-miR-1260a

AUCCCACCUCUGCCACCA

>hsa-miR-1260b

AUCCCACCACUGCCACCAU

>hsa-miR-1262

AUGGGUGAAUUUGUAGAAGGAU

>hsa-miR-1263

AUGGUACCCUGGCAUACUGAGU

>hsa-miR-1266-5p

CCUCAGGGCUGUAGAACAGGGCU

>hsa-miR-1268a

CGGGCGUGGUGGUGGGGG

>hsa-miR-1269a

CUGGACUGAGCCGUGCUACUGG

>hsa-miR-1269b

CUGGACUGAGCCAUGCUACUGG

>hsa-miR-127-5p

CUGAAGCUCAGAGGGCUCUGAU

>hsa-miR-127-3p

UCGGAUCCGUCUGAGCUUGGCU

>hsa-miR-1270

CUGGAGAUAUGGAAGAGCUGUGU

>hsa-miR-1271-5p

CUUGGCACCUAGCAAGCACUCA

>hsa-miR-1271-3p

AGUGCCUGCUAUGUGCCAGGCA

>hsa-miR-1273c

GGCGACAAAACGAGACCCUGUC

>hsa-miR-1273h-5p

CUGGGAGGUCAAGGCUGCAGU

>hsa-miR-1273h-3p

CUGCAGACUCGACCUCCCAGGC

>hsa-miR-1275

GUGGGGGAGAGGCUGUC

>hsa-miR-1276

UAAAGAGCCCUGUGGAGACA

>hsa-miR-1277-3p

UACGUAGAUAUAUAUGUAUUUU

>hsa-miR-1278

UAGUACUGUGCAUAUCAUCUAU

>hsa-miR-128-1-5p

CGGGGCCGUAGCACUGUCUGAGA

>hsa-miR-128-3p

UCACAGUGAACCGGUCUCUUU

>hsa-miR-128-2-5p

GGGGGCCGAUACACUGUACGAGA

>hsa-miR-1283

UCUACAAAGGAAAGCGCUUUCU

>hsa-miR-1284

UCUAUACAGACCCUGGCUUUUC

>hsa-miR-1285-5p

GAUCUCACUUUGUUGCCCAGG

>hsa-miR-1285-3p

UCUGGGCAACAAAGUGAGACCU

>hsa-miR-1286

UGCAGGACCAAGAUGAGCCCU

>hsa-miR-1287-5p

UGCUGGAUCAGUGGUUCGAGUC

>hsa-miR-1287-3p

CUCUAGCCACAGAUGCAGUGAU

>hsa-miR-1288-3p

UGGACUGCCCUGAUCUGGAGA

>hsa-miR-1289

UGGAGUCCAGGAAUCUGCAUUUU

>hsa-miR-129-5p

CUUUUUGCGGUCUGGGCUUGC

>hsa-miR-129-1-3p

AAGCCCUUACCCCAAAAAGUAU

>hsa-miR-129-2-3p

AAGCCCUUACCCCAAAAAGCAU

>hsa-miR-1291

UGGCCCUGACUGAAGACCAGCAGU

>hsa-miR-1292-5p

UGGGAACGGGUUCCGGCAGACGCUG

>hsa-miR-1292-3p

UCGCGCCCCGGCUCCCGUUC

>hsa-miR-1293

UGGGUGGUCUGGAGAUUUGUGC

>hsa-miR-1294

UGUGAGGUUGGCAUUGUUGUCU

>hsa-miR-1295a

UUAGGCCGCAGAUCUGGGUGA

>hsa-miR-1295b-5p

CACCCAGAUCUGCGGCCUAAU

>hsa-miR-1296-5p

UUAGGGCCCUGGCUCCAUCUCC

>hsa-miR-1298-5p

UUCAUUCGGCUGUCCAGAUGUA

>hsa-miR-1301-3p

UUGCAGCUGCCUGGGAGUGACUUC

>hsa-miR-1302

UUGGGACAUACUUAUGCUAAA

>hsa-miR-1303

UUUAGAGACGGGGUCUUGCUCU

>hsa-miR-1304-5p

UUUGAGGCUACAGUGAGAUGUG

>hsa-miR-1305

UUUUCAACUCUAAUGGGAGAGA

>hsa-miR-1306-5p

CCACCUCCCCUGCAAACGUCCA

>hsa-miR-1306-3p

ACGUUGGCUCUGGUGGUG

>hsa-miR-1307-5p

UCGACCGGACCUCGACCGGCU

>hsa-miR-1307-3p

ACUCGGCGUGGCGUCGGUCGUG

>hsa-miR-130a-5p

GCUCUUUUCACAUUGUGCUACU

>hsa-miR-130a-3p

CAGUGCAAUGUUAAAAGGGCAU

>hsa-miR-130b-5p

ACUCUUUCCCUGUUGCACUAC

>hsa-miR-130b-3p

CAGUGCAAUGAUGAAAGGGCAU

>hsa-miR-132-5p

ACCGUGGCUUUCGAUUGUUACU

>hsa-miR-132-3p

UAACAGUCUACAGCCAUGGUCG

>hsa-miR-1322

GAUGAUGCUGCUGAUGCUG

>hsa-miR-1323

UCAAAACUGAGGGGCAUUUUCU

>hsa-miR-133a-5p

AGCUGGUAAAAUGGAACCAAAU

>hsa-miR-133a-3p

UUUGGUCCCCUUCAACCAGCUG

>hsa-miR-133b

UUUGGUCCCCUUCAACCAGCUA

>hsa-miR-134-5p

UGUGACUGGUUGACCAGAGGGG

>hsa-miR-1343-3p

CUCCUGGGGCCCGCACUCUCGC

>hsa-miR-135a-5p

UAUGGCUUUUUAUUCCUAUGUGA

>hsa-miR-135a-3p

UAUAGGGAUUGGAGCCGUGGCG

>hsa-miR-135b-5p

UAUGGCUUUUCAUUCCUAUGUGA

>hsa-miR-135b-3p

AUGUAGGGCUAAAAGCCAUGGG

>hsa-miR-136-5p

ACUCCAUUUGUUUUGAUGAUGGA

>hsa-miR-136-3p

CAUCAUCGUCUCAAAUGAGUCU

>hsa-miR-137-5p

ACGGGUAUUCUUGGGUGGAUAAU

>hsa-miR-137-3p

UUAUUGCUUAAGAAUACGCGUAG

>hsa-miR-138-5p

AGCUGGUGUUGUGAAUCAGGCCG

>hsa-miR-138-2-3p

GCUAUUUCACGACACCAGGGUU

>hsa-miR-139-5p

UCUACAGUGCACGUGUCUCCAGU

>hsa-miR-139-3p

UGGAGACGCGGCCCUGUUGGAGU

>hsa-miR-140-5p

CAGUGGUUUUACCCUAUGGUAG

>hsa-miR-140-3p

UACCACAGGGUAGAACCACGG

>hsa-miR-141-5p

CAUCUUCCAGUACAGUGUUGGA

>hsa-miR-141-3p

UAACACUGUCUGGUAAAGAUGG

>hsa-miR-142-5p

CAUAAAGUAGAAAGCACUACU

>hsa-miR-142-3p

UGUAGUGUUUCCUACUUUAUGGA

>hsa-miR-143-5p

GGUGCAGUGCUGCAUCUCUGGU

>hsa-miR-143-3p

UGAGAUGAAGCACUGUAGCUC

>hsa-miR-144-5p

GGAUAUCAUCAUAUACUGUAAG

>hsa-miR-144-3p

UACAGUAUAGAUGAUGUACU

>hsa-miR-145-5p

GUCCAGUUUUCCCAGGAAUCCCU

>hsa-miR-145-3p

GGAUUCCUGGAAAUACUGUUCU

>hsa-miR-1468-5p

CUCCGUUUGCCUGUUUCGCUG

>hsa-miR-146a-5p

UGAGAACUGAAUUCCAUGGGUU

>hsa-miR-146a-3p

CCUCUGAAAUUCAGUUCUUCAG

>hsa-miR-146b-5p

UGAGAACUGAAUUCCAUAGGCUG

>hsa-miR-146b-3p

GCCCUGUGGACUCAGUUCUGGU

>hsa-miR-147b-5p

UGGAAACAUUUCUGCACAAACU

>hsa-miR-147b-3p

GUGUGCGGAAAUGCUUCUGCU

>hsa-miR-148a-5p

AAAGUUCUGAGACACUCCGACU

>hsa-miR-148a-3p

UCAGUGCACUACAGAACUUUGU

>hsa-miR-148b-5p

AAGUUCUGUUAUACACUCAGGC

>hsa-miR-148b-3p

UCAGUGCAUCACAGAACUUUGU

>hsa-miR-149-5p

UCUGGCUCCGUGUCUUCACUCCC

>hsa-miR-149-3p

AGGGAGGGACGGGGGCUGUGC

>hsa-miR-150-5p

UCUCCCAACCCUUGUACCAGUG

>hsa-miR-150-3p

CUGGUACAGGCCUGGGGGACAG

>hsa-miR-151a-5p

UCGAGGAGCUCACAGUCUAGU

>hsa-miR-151a-3p

CUAGACUGAAGCUCCUUGAGG

>hsa-miR-151b

UCGAGGAGCUCACAGUCU

>hsa-miR-152-5p

AGGUUCUGUGAUACACUCCGACU

>hsa-miR-152-3p

UCAGUGCAUGACAGAACUUGG

>hsa-miR-153-3p

UUGCAUAGUCACAAAAGUGAUC

>hsa-miR-153-5p

UCAUUUUUGUGAUGUUGCAGCU

>hsa-miR-1537-5p

AGCUGUAAUUAGUCAGUUUUCU

>hsa-miR-1537-3p

AAAACCGUCUAGUUACAGUUGU

>hsa-miR-1538

CGGCCCGGGCUGCUGCUGUUCCU

>hsa-miR-155-5p

UUAAUGCUAAUCGUGAUAGGGGUU

>hsa-miR-155-3p

CUCCUACAUAUUAGCAUUAACA

>hsa-miR-15a-5p

UAGCAGCACAUAAUGGUUUGUG

>hsa-miR-15a-3p

CAGGCCAUAUUGUGCUGCCUCA

>hsa-miR-15b-5p

UAGCAGCACAUCAUGGUUUACA

>hsa-miR-15b-3p

CGAAUCAUUAUUUGCUGCUCUA

>hsa-miR-16-5p

UAGCAGCACGUAAAUAUUGGCG

>hsa-miR-16-1-3p

CCAGUAUUAACUGUGCUGCUGA

>hsa-miR-16-2-3p

CCAAUAUUACUGUGCUGCUUUA

>hsa-miR-17-5p

CAAAGUGCUUACAGUGCAGGUAG

>hsa-miR-17-3p

ACUGCAGUGAAGGCACUUGUAG

>hsa-miR-181a-5p

AACAUUCAACGCUGUCGGUGAGU

>hsa-miR-181a-3p

ACCAUCGACCGUUGAUUGUACC

>hsa-miR-181a-2-3p

ACCACUGACCGUUGACUGUACC

>hsa-miR-181b-5p

AACAUUCAUUGCUGUCGGUGGGU

>hsa-miR-181b-3p

CUCACUGAACAAUGAAUGCAA

>hsa-miR-181b-2-3p

CUCACUGAUCAAUGAAUGCA

>hsa-miR-181c-5p

AACAUUCAACCUGUCGGUGAGU

>hsa-miR-181c-3p

AACCAUCGACCGUUGAGUGGAC

>hsa-miR-181d-5p

AACAUUCAUUGUUGUCGGUGGGU

>hsa-miR-181d-3p

CCACCGGGGGAUGAAUGUCAC

>hsa-miR-182-5p

UUUGGCAAUGGUAGAACUCACACU

>hsa-miR-182-3p

UGGUUCUAGACUUGCCAACUA

>hsa-miR-183-5p

UAUGGCACUGGUAGAAUUCACU

>hsa-miR-183-3p

GUGAAUUACCGAAGGGCCAUAA

>hsa-miR-184

UGGACGGAGAACUGAUAAGGGU

>hsa-miR-1843

UAUGGAGGUCUCUGUCUGGC

>hsa-miR-185-5p

UGGAGAGAAAGGCAGUUCCUGA

>hsa-miR-185-3p

AGGGGCUGGCUUUCCUCUGGUC

>hsa-miR-186-5p

CAAAGAAUUCUCCUUUUGGGCU

>hsa-miR-186-3p

GCCCAAAGGUGAAUUUUUUGGG

>hsa-miR-187-3p

UCGUGUCUUGUGUUGCAGCCGG

>hsa-miR-188-5p

CAUCCCUUGCAUGGUGGAGGG

>hsa-miR-188-3p

CUCCCACAUGCAGGGUUUGCA

>hsa-miR-18a-5p

UAAGGUGCAUCUAGUGCAGAUAG

>hsa-miR-18a-3p

ACUGCCCUAAGUGCUCCUUCUGG

>hsa-miR-18b-5p

UAAGGUGCAUCUAGUGCAGUUAG

>hsa-miR-1908-5p

CGGCGGGGACGGCGAUUGGUC

>hsa-miR-1908-3p

CCGGCCGCCGGCUCCGCCCCG

>hsa-miR-1909-3p

CGCAGGGGCCGGGUGCUCACCG

>hsa-miR-190a-5p

UGAUAUGUUUGAUAUAUUAGGU

>hsa-miR-190a-3p

CUAUAUAUCAAACAUAUUCCU

>hsa-miR-190b-5p

UGAUAUGUUUGAUAUUGGGUUG

>hsa-miR-190b-3p

ACUAAAUGUCAAACAUAUUCU

>hsa-miR-191-5p

CAACGGAAUCCCAAAAGCAGCUG

>hsa-miR-191-3p

GCUGCGCUUGGAUUUCGUCCCC

>hsa-miR-1910-5p

CCAGUCCUGUGCCUGCCGCCU

>hsa-miR-1910-3p

GAGGCAGAAGCAGGAUGACA

>hsa-miR-1911-5p

UGAGUACCGCCAUGUCUGUUGGG

>hsa-miR-1912-5p

CUCAUUGCAUGGGCUGUGUAUA

>hsa-miR-1913

UCUGCCCCCUCCGCUGCUGCCA

>hsa-miR-1914-5p

CCCUGUGCCCGGCCCACUUCUG

>hsa-miR-1915-5p

ACCUUGCCUUGCUGCCCGGGCC

>hsa-miR-1915-3p

CCCCAGGGCGACGCGGCGGG

>hsa-miR-192-5p

CUGACCUAUGAAUUGACAGCC

>hsa-miR-192-3p

CUGCCAAUUCCAUAGGUCACAG

>hsa-miR-193a-5p

UGGGUCUUUGCGGGCGAGAUGA

>hsa-miR-193a-3p

AACUGGCCUACAAAGUCCCAGU

>hsa-miR-193b-5p

CGGGGUUUUGAGGGCGAGAUGA

>hsa-miR-193b-3p

AACUGGCCCUCAAAGUCCCGCU

>hsa-miR-194-5p

UGUAACAGCAACUCCAUGUGGA

>hsa-miR-194-3p

CCAGUGGGGCUGCUGUUAUCUG

>hsa-miR-195-5p

UAGCAGCACAGAAAUAUUGGC

>hsa-miR-195-3p

CCAAUAUUGGCUGUGCUGCUCC

>hsa-miR-196a-5p

UAGGUAGUUUCAUGUUGUUGGG

>hsa-miR-196b-5p

UAGGUAGUUUCCUGUUGUUGGG

>hsa-miR-196b-3p

UCGACAGCACGACACUGCCUUC

>hsa-miR-197-5p

CGGGUAGAGAGGGCAGUGGGAGG

>hsa-miR-197-3p

UUCACCACCUUCUCCACCCAGC

>hsa-miR-1972

UCAGGCCAGGCACAGUGGCUCA

>hsa-miR-1976

CCUCCUGCCCUCCUUGCUGU

>hsa-miR-199a-5p

CCCAGUGUUCAGACUACCUGUUC

>hsa-miR-199a-3p

ACAGUAGUCUGCACAUUGGUUA

>hsa-miR-199b-5p

CCCAGUGUUUAGACUAUCUGUUC

>hsa-miR-19a-5p

AGUUUUGCAUAGUUGCACUACA

>hsa-miR-19a-3p

UGUGCAAAUCUAUGCAAAACUGA

>hsa-miR-19b-1-5p

AGUUUUGCAGGUUUGCAUCCAGC

>hsa-miR-19b-3p

UGUGCAAAUCCAUGCAAAACUGA

>hsa-miR-19b-2-5p

AGUUUUGCAGGUUUGCAUUUCA

>hsa-miR-200a-5p

CAUCUUACCGGACAGUGCUGGA

>hsa-miR-200a-3p

UAACACUGUCUGGUAACGAUGU

>hsa-miR-200b-5p

CAUCUUACUGGGCAGCAUUGGA

>hsa-miR-200b-3p

UAAUACUGCCUGGUAAUGAUGA

>hsa-miR-200c-5p

CGUCUUACCCAGCAGUGUUUGG

>hsa-miR-200c-3p

UAAUACUGCCGGGUAAUGAUGGA

>hsa-miR-202-5p

UUCCUAUGCAUAUACUUCUUUG

>hsa-miR-203a-5p

AGUGGUUCUUAACAGUUCAACAGUU

>hsa-miR-203a-3p

GUGAAAUGUUUAGGACCACUAG

>hsa-miR-203b-5p

UAGUGGUCCUAAACAUUUCACA

>hsa-miR-203b-3p

UUGAACUGUUAAGAACCACUGGA

>hsa-miR-204-5p

UUCCCUUUGUCAUCCUAUGCCU

>hsa-miR-205-5p

UCCUUCAUUCCACCGGAGUCUG

>hsa-miR-206

UGGAAUGUAAGGAAGUGUGUGG

>hsa-miR-208a-3p

AUAAGACGAGCAAAAAGCUUGU

>hsa-miR-208b-3p

AUAAGACGAACAAAAGGUUUGU

>hsa-miR-20a-5p

UAAAGUGCUUAUAGUGCAGGUAG

>hsa-miR-20a-3p

ACUGCAUUAUGAGCACUUAAAG

>hsa-miR-20b-5p

CAAAGUGCUCAUAGUGCAGGUAG

>hsa-miR-20b-3p

ACUGUAGUAUGGGCACUUCCAG

>hsa-miR-21-5p

UAGCUUAUCAGACUGAUGUUGA

>hsa-miR-21-3p

CAACACCAGUCGAUGGGCUGU

>hsa-miR-210-5p

AGCCCCUGCCCACCGCACACUG

>hsa-miR-210-3p

CUGUGCGUGUGACAGCGGCUGA

>hsa-miR-211-5p

UUCCCUUUGUCAUCCUUCGCCU

>hsa-miR-2110

UUGGGGAAACGGCCGCUGAGUG

>hsa-miR-2113

AUUUGUGCUUGGCUCUGUCAC

>hsa-miR-2114-5p

UAGUCCCUUCCUUGAAGCGGUC

>hsa-miR-2115-5p

AGCUUCCAUGACUCCUGAUGGA

>hsa-miR-2115-3p

CAUCAGAAUUCAUGGAGGCUAG

>hsa-miR-2116-5p

GGUUCUUAGCAUAGGAGGUCU

>hsa-miR-2116-3p

CCUCCCAUGCCAAGAACUCCC

>hsa-miR-212-5p

ACCUUGGCUCUAGACUGCUUACU

>hsa-miR-212-3p

UAACAGUCUCCAGUCACGGCC

>hsa-miR-214-5p

UGCCUGUCUACACUUGCUGUGC

>hsa-miR-214-3p

ACAGCAGGCACAGACAGGCAGU

>hsa-miR-215-5p

AUGACCUAUGAAUUGACAGAC

>hsa-miR-215-3p

UCUGUCAUUUCUUUAGGCCAAUA

>hsa-miR-216a-5p

UAAUCUCAGCUGGCAACUGUGA

>hsa-miR-216a-3p

UCACAGUGGUCUCUGGGAUUAU

>hsa-miR-216b-5p

AAAUCUCUGCAGGCAAAUGUGA

>hsa-miR-216b-3p

ACACACUUACCCGUAGAGAUUCUA

>hsa-miR-217-5p

UACUGCAUCAGGAACUGAUUGGA

>hsa-miR-218-5p

UUGUGCUUGAUCUAACCAUGU

>hsa-miR-219a-5p

UGAUUGUCCAAACGCAAUUCU

>hsa-miR-219a-1-3p

AGAGUUGAGUCUGGACGUCCCG

>hsa-miR-219a-2-3p

AGAAUUGUGGCUGGACAUCUGU

>hsa-miR-219b-5p

AGAUGUCCAGCCACAAUUCUCG

>hsa-miR-219b-3p

AGAAUUGCGUUUGGACAAUCAGU

>hsa-miR-22-5p

AGUUCUUCAGUGGCAAGCUUUA

>hsa-miR-22-3p

AAGCUGCCAGUUGAAGAACUGU

>hsa-miR-221-5p

ACCUGGCAUACAAUGUAGAUUU

>hsa-miR-221-3p

AGCUACAUUGUCUGCUGGGUUUC

>hsa-miR-222-5p

CUCAGUAGCCAGUGUAGAUCCU

>hsa-miR-222-3p

AGCUACAUCUGGCUACUGGGU

>hsa-miR-223-5p

CGUGUAUUUGACAAGCUGAGUU

>hsa-miR-223-3p

UGUCAGUUUGUCAAAUACCCCA

>hsa-miR-224-5p

UCAAGUCACUAGUGGUUCCGUUUAG

>hsa-miR-224-3p

AAAAUGGUGCCCUAGUGACUACA

>hsa-miR-2276-3p

UCUGCAAGUGUCAGAGGCGAGG

>hsa-miR-2277-5p

AGCGCGGGCUGAGCGCUGCCAGUC

>hsa-miR-2277-3p

UGACAGCGCCCUGCCUGGCUC

>hsa-miR-2278

GAGAGCAGUGUGUGUUGCCUGG

>hsa-miR-2355-5p

AUCCCCAGAUACAAUGGACAA

>hsa-miR-2355-3p

AUUGUCCUUGCUGUUUGGAGAU

>hsa-miR-23a-5p

GGGGUUCCUGGGGAUGGGAUUU

>hsa-miR-23a-3p

AUCACAUUGCCAGGGAUUUCC

>hsa-miR-23b-5p

UGGGUUCCUGGCAUGCUGAUUU

>hsa-miR-23b-3p

AUCACAUUGCCAGGGAUUACCAC

>hsa-miR-23c

AUCACAUUGCCAGUGAUUACCC

>hsa-miR-24-1-5p

UGCCUACUGAGCUGAUAUCAGU

>hsa-miR-24-3p

UGGCUCAGUUCAGCAGGAACAG

>hsa-miR-24-2-5p

UGCCUACUGAGCUGAAACACAG

>hsa-miR-2467-5p

UGAGGCUCUGUUAGCCUUGGCUC

>hsa-miR-25-5p

AGGCGGAGACUUGGGCAAUUG

>hsa-miR-25-3p

CAUUGCACUUGUCUCGGUCUGA

>hsa-miR-2682-5p

CAGGCAGUGACUGUUCAGACGUC

>hsa-miR-2682-3p

CGCCUCUUCAGCGCUGUCUUCC

>hsa-miR-26a-5p

UUCAAGUAAUCCAGGAUAGGCU

>hsa-miR-26a-1-3p

CCUAUUCUUGGUUACUUGCACG

>hsa-miR-26a-2-3p

CCUAUUCUUGAUUACUUGUUUC

>hsa-miR-26b-5p

UUCAAGUAAUUCAGGAUAGGU

>hsa-miR-26b-3p

CCUGUUCUCCAUUACUUGGCU

>hsa-miR-27a-5p

AGGGCUUAGCUGCUUGUGAGCA

>hsa-miR-27a-3p

UUCACAGUGGCUAAGUUCCGC

>hsa-miR-27b-5p

AGAGCUUAGCUGAUUGGUGAAC

>hsa-miR-27b-3p

UUCACAGUGGCUAAGUUCUGC

>hsa-miR-28-5p

AAGGAGCUCACAGUCUAUUGAG

>hsa-miR-28-3p

CACUAGAUUGUGAGCUCCUGGA

>hsa-miR-296-3p

GAGGGUUGGGUGGAGGCUCUCC

>hsa-miR-29a-5p

ACUGAUUUCUUUUGGUGUUCAG

>hsa-miR-29a-3p

UAGCACCAUCUGAAAUCGGUUA

>hsa-miR-29b-1-5p

GCUGGUUUCAUAUGGUGGUUUAGA

>hsa-miR-29b-3p

UAGCACCAUUUGAAAUCAGUGUU

>hsa-miR-29b-2-5p

CUGGUUUCACAUGGUGGCUUAG

>hsa-miR-29c-5p

UGACCGAUUUCUCCUGGUGUUC

>hsa-miR-29c-3p

UAGCACCAUUUGAAAUCGGUUA

>hsa-miR-301a-5p

GCUCUGACUUUAUUGCACUACU

>hsa-miR-301a-3p

CAGUGCAAUAGUAUUGUCAAAGC

>hsa-miR-301b-5p

GCUCUGACGAGGUUGCACUACU

>hsa-miR-301b-3p

CAGUGCAAUGAUAUUGUCAAAGC

>hsa-miR-302a-5p

ACUUAAACGUGGAUGUACUUGCU

>hsa-miR-302a-3p

UAAGUGCUUCCAUGUUUUGGUGA

>hsa-miR-302b-5p

ACUUUAACAUGGAAGUGCUUUC

>hsa-miR-302b-3p

UAAGUGCUUCCAUGUUUUAGUAG

>hsa-miR-302c-5p

UUUAACAUGGGGGUACCUGCUG

>hsa-miR-302c-3p

UAAGUGCUUCCAUGUUUCAGUGG

>hsa-miR-302d-5p

ACUUUAACAUGGAGGCACUUGC

>hsa-miR-302d-3p

UAAGUGCUUCCAUGUUUGAGUGU

>hsa-miR-3059-5p

UUUCCUCUCUGCCCCAUAGGGUGU

>hsa-miR-3064-5p

UCUGGCUGUUGUGGUGUGCAA

>hsa-miR-3064-3p

UUGCCACACUGCAACACCUUACA

>hsa-miR-3065-5p

UCAACAAAAUCACUGAUGCUGGA

>hsa-miR-3065-3p

UCAGCACCAGGAUAUUGUUGGAG

>hsa-miR-3074-5p

GUUCCUGCUGAACUGAGCCAG

>hsa-miR-3074-3p

GAUAUCAGCUCAGUAGGCACCG

>hsa-miR-30a-5p

UGUAAACAUCCUCGACUGGAAG

>hsa-miR-30a-3p

CUUUCAGUCGGAUGUUUGCAGC

>hsa-miR-30b-5p

UGUAAACAUCCUACACUCAGCU

>hsa-miR-30b-3p

CUGGGAGGUGGAUGUUUACUUC

>hsa-miR-30c-5p

UGUAAACAUCCUACACUCUCAGC

>hsa-miR-30c-1-3p

CUGGGAGAGGGUUGUUUACUCC

>hsa-miR-30c-2-3p

CUGGGAGAAGGCUGUUUACUCU

>hsa-miR-30d-5p

UGUAAACAUCCCCGACUGGAAG

>hsa-miR-30d-3p

CUUUCAGUCAGAUGUUUGCUGC

>hsa-miR-30e-5p

UGUAAACAUCCUUGACUGGAAG

>hsa-miR-30e-3p

CUUUCAGUCGGAUGUUUACAGC

>hsa-miR-31-5p

AGGCAAGAUGCUGGCAUAGCU

>hsa-miR-31-3p

UGCUAUGCCAACAUAUUGCCAU

>hsa-miR-3115

AUAUGGGUUUACUAGUUGGU

>hsa-miR-3117-3p

AUAGGACUCAUAUAGUGCCAG

>hsa-miR-3118

UGUGACUGCAUUAUGAAAAUUCU

>hsa-miR-3120-5p

CCUGUCUGUGCCUGCUGUACA

>hsa-miR-3120-3p

CACAGCAAGUGUAGACAGGCA

>hsa-miR-3121-5p

UCCUUUGCCUAUUCUAUUUAAG

>hsa-miR-3121-3p

UAAAUAGAGUAGGCAAAGGACA

>hsa-miR-3122

GUUGGGACAAGAGGACGGUCUU

>hsa-miR-3124-5p

UUCGCGGGCGAAGGCAAAGUC

>hsa-miR-3126-3p

CAUCUGGCAUCCGUCACACAGA

>hsa-miR-3127-5p

AUCAGGGCUUGUGGAAUGGGAAG

>hsa-miR-3127-3p

UCCCCUUCUGCAGGCCUGCUGG

>hsa-miR-3128

UCUGGCAAGUAAAAAACUCUCAU

>hsa-miR-3129-5p

GCAGUAGUGUAGAGAUUGGUUU

>hsa-miR-3129-3p

AAACUAAUCUCUACACUGCUGC

>hsa-miR-3130-5p

UACCCAGUCUCCGGUGCAGCC

>hsa-miR-3130-3p

GCUGCACCGGAGACUGGGUAA

>hsa-miR-3133

UAAAGAACUCUUAAAACCCAAU

>hsa-miR-3134

UGAUGGAUAAAAGACUACAUAUU

>hsa-miR-3135a

UGCCUAGGCUGAGACUGCAGUG

>hsa-miR-3136-5p

CUGACUGAAUAGGUAGGGUCAUU

>hsa-miR-3136-3p

UGGCCCAACCUAUUCAGUUAGU

>hsa-miR-3137

UCUGUAGCCUGGGAGCAAUGGGGU

>hsa-miR-3138

UGUGGACAGUGAGGUAGAGGGAGU

>hsa-miR-3139

UAGGAGCUCAACAGAUGCCUGUU

>hsa-miR-3140-5p

ACCUGAAUUACCAAAAGCUUU

>hsa-miR-3140-3p

AGCUUUUGGGAAUUCAGGUAGU

>hsa-miR-3141

GAGGGCGGGUGGAGGAGGA

>hsa-miR-3142

AAGGCCUUUCUGAACCUUCAGA

>hsa-miR-3143

AUAACAUUGUAAAGCGCUUCUUUCG

>hsa-miR-3144-5p

AGGGGACCAAAGAGAUAUAUAG

>hsa-miR-3144-3p

AUAUACCUGUUCGGUCUCUUUA

>hsa-miR-3145-5p

AACUCCAAACACUCAAAACUCA

>hsa-miR-3145-3p

AGAUAUUUUGAGUGUUUGGAAUUG

>hsa-miR-3146

CAUGCUAGGAUAGAAAGAAUGG

>hsa-miR-3148

UGGAAAAAACUGGUGUGUGCUU

>hsa-miR-3149

UUUGUAUGGAUAUGUGUGUGUAU

>hsa-miR-3150a-5p

CAACCUCGACGAUCUCCUCAGC

>hsa-miR-3150a-3p

CUGGGGAGAUCCUCGAGGUUGG

>hsa-miR-3150b-5p

CAACCUCGAGGAUCUCCCCAGC

>hsa-miR-3150b-3p

UGAGGAGAUCGUCGAGGUUGG

>hsa-miR-3151-5p

GGUGGGGCAAUGGGAUCAGGU

>hsa-miR-3154

CAGAAGGGGAGUUGGGAGCAGA

>hsa-miR-3155a

CCAGGCUCUGCAGUGGGAACU

>hsa-miR-3155b

CCAGGCUCUGCAGUGGGA

>hsa-miR-3157-5p

UUCAGCCAGGCUAGUGCAGUCU

>hsa-miR-3157-3p

CUGCCCUAGUCUAGCUGAAGCU

>hsa-miR-3158-5p

CCUGCAGAGAGGAAGCCCUUC

>hsa-miR-3158-3p

AAGGGCUUCCUCUCUGCAGGAC

>hsa-miR-3159

UAGGAUUACAAGUGUCGGCCAC

>hsa-miR-3160-5p

GGCUUUCUAGUCUCAGCUCUCC

>hsa-miR-3160-3p

AGAGCUGAGACUAGAAAGCCCA

>hsa-miR-3162-5p

UUAGGGAGUAGAAGGGUGGGGAG

>hsa-miR-3162-3p

UCCCUACCCCUCCACUCCCCA

>hsa-miR-3163

UAUAAAAUGAGGGCAGUAAGAC

>hsa-miR-3164

UGUGACUUUAAGGGAAAUGGCG

>hsa-miR-3165

AGGUGGAUGCAAUGUGACCUCA

>hsa-miR-3173-5p

UGCCCUGCCUGUUUUCUCCUUU

>hsa-miR-3173-3p

AAAGGAGGAAAUAGGCAGGCCA

>hsa-miR-3174

UAGUGAGUUAGAGAUGCAGAGCC

>hsa-miR-3175

CGGGGAGAGAACGCAGUGACGU

>hsa-miR-3176

ACUGGCCUGGGACUACCGG

>hsa-miR-3177-5p

UGUGUACACACGUGCCAGGCGCU

>hsa-miR-3177-3p

UGCACGGCACUGGGGACACGU

>hsa-miR-3179

AGAAGGGGUGAAAUUUAAACGU

>hsa-miR-3180-5p

CUUCCAGACGCUCCGCCCCACGUCG

>hsa-miR-3180-3p

UGGGGCGGAGCUUCCGGAGGCC

>hsa-miR-3180

UGGGGCGGAGCUUCCGGAG

>hsa-miR-3181

AUCGGGCCCUCGGCGCCGG

>hsa-miR-3183

GCCUCUCUCGGAGUCGCUCGGA

>hsa-miR-3184-5p

UGAGGGGCCUCAGACCGAGCUUUU

>hsa-miR-3184-3p

AAAGUCUCGCUCUCUGCCCCUCA

>hsa-miR-3186-5p

CAGGCGUCUGUCUACGUGGCUU

>hsa-miR-3186-3p

UCACGCGGAGAGAUGGCUUUG

>hsa-miR-3187-5p

CCUGGGCAGCGUGUGGCUGAAGG

>hsa-miR-3187-3p

UUGGCCAUGGGGCUGCGCGG

>hsa-miR-3188

AGAGGCUUUGUGCGGAUACGGGG

>hsa-miR-3189-5p

UGCCCCAUCUGUGCCCUGGGUAGGA

>hsa-miR-3189-3p

CCCUUGGGUCUGAUGGGGUAG

>hsa-miR-3190-5p

UCUGGCCAGCUACGUCCCCA

>hsa-miR-3190-3p

UGUGGAAGGUAGACGGCCAGAGA

>hsa-miR-3191-5p

CUCUCUGGCCGUCUACCUUCCA

>hsa-miR-3191-3p

UGGGGACGUAGCUGGCCAGACAG

>hsa-miR-3192-5p

UCUGGGAGGUUGUAGCAGUGGAA

>hsa-miR-3194-3p

AGCUCUGCUGCUCACUGGCAGU

>hsa-miR-3198

GUGGAGUCCUGGGGAAUGGAGA

>hsa-miR-3199

AGGGACUGCCUUAGGAGAAAGUU

>hsa-miR-32-5p

UAUUGCACAUUACUAAGUUGCA

>hsa-miR-32-3p

CAAUUUAGUGUGUGUGAUAUUU

>hsa-miR-3200-5p

AAUCUGAGAAGGCGCACAAGGU

>hsa-miR-3200-3p

CACCUUGCGCUACUCAGGUCUG

>hsa-miR-3202

UGGAAGGGAGAAGAGCUUUAAU

>hsa-miR-320a-5p

GCCUUCUCUUCCCGGUUCUUCC

>hsa-miR-320a-3p

AAAAGCUGGGUUGAGAGGGCGA

>hsa-miR-320b

AAAAGCUGGGUUGAGAGGGCAA

>hsa-miR-320c

AAAAGCUGGGUUGAGAGGGU

>hsa-miR-320d

AAAAGCUGGGUUGAGAGGA

>hsa-miR-320e

AAAGCUGGGUUGAGAAGG

>hsa-miR-323a-3p

CACAUUACACGGUCGACCUCU

>hsa-miR-324-5p

CGCAUCCCCUAGGGCAUUGGUG

>hsa-miR-324-3p

CCCACUGCCCCAGGUGCUGCUGG

>hsa-miR-326

CCUCUGGGCCCUUCCUCCAG

>hsa-miR-328-3p

CUGGCCCUCUCUGCCCUUCCGU

>hsa-miR-329-3p

AACACACCUGGUUAACCUCUUU

>hsa-miR-330-5p

UCUCUGGGCCUGUGUCUUAGGC

>hsa-miR-330-3p

GCAAAGCACACGGCCUGCAGAGA

>hsa-miR-331-5p

CUAGGUAUGGUCCCAGGGAUCC

>hsa-miR-331-3p

GCCCCUGGGCCUAUCCUAGAA

>hsa-miR-335-5p

UCAAGAGCAAUAACGAAAAAUGU

>hsa-miR-335-3p

UUUUUCAUUAUUGCUCCUGACC

>hsa-miR-338-5p

AACAAUAUCCUGGUGCUGAGUG

>hsa-miR-338-3p

UCCAGCAUCAGUGAUUUUGUUG

>hsa-miR-339-5p

UCCCUGUCCUCCAGGAGCUCACG

>hsa-miR-339-3p

UGAGCGCCUCGACGACAGAGCCG

>hsa-miR-33a-5p

GUGCAUUGUAGUUGCAUUGCA

>hsa-miR-33a-3p

CAAUGUUUCCACAGUGCAUCAC

>hsa-miR-33b-5p

GUGCAUUGCUGUUGCAUUGC

>hsa-miR-33b-3p

CAGUGCCUCGGCAGUGCAGCCC

>hsa-miR-340-5p

UUAUAAAGCAAUGAGACUGAUU

>hsa-miR-340-3p

UCCGUCUCAGUUACUUUAUAGC

>hsa-miR-342-5p

AGGGGUGCUAUCUGUGAUUGA

>hsa-miR-342-3p

UCUCACACAGAAAUCGCACCCGU

>hsa-miR-345-5p

GCUGACUCCUAGUCCAGGGCUC

>hsa-miR-345-3p

GCCCUGAACGAGGGGUCUGGAG

>hsa-miR-346

UGUCUGCCCGCAUGCCUGCCUCU

>hsa-miR-34a-5p

UGGCAGUGUCUUAGCUGGUUGU

>hsa-miR-34a-3p

CAAUCAGCAAGUAUACUGCCCU

>hsa-miR-34b-5p

UAGGCAGUGUCAUUAGCUGAUUG

>hsa-miR-34b-3p

CAAUCACUAACUCCACUGCCAU

>hsa-miR-34c-5p

AGGCAGUGUAGUUAGCUGAUUGC

>hsa-miR-34c-3p

AAUCACUAACCACACGGCCAGG

>hsa-miR-3529-5p

AGGUAGACUGGGAUUUGUUGUU

>hsa-miR-3529-3p

AACAACAAAAUCACUAGUCUUCCA

>hsa-miR-3605-5p

UGAGGAUGGAUAGCAAGGAAGCC

>hsa-miR-3605-3p

CCUCCGUGUUACCUGUCCUCUAG

>hsa-miR-3609

CAAAGUGAUGAGUAAUACUGGCUG

>hsa-miR-361-5p

UUAUCAGAAUCUCCAGGGGUAC

>hsa-miR-361-3p

UCCCCCAGGUGUGAUUCUGAUUU

>hsa-miR-3610

GAAUCGGAAAGGAGGCGCCG

>hsa-miR-3611

UUGUGAAGAAAGAAAUUCUUA

>hsa-miR-3614-5p

CCACUUGGAUCUGAAGGCUGCCC

>hsa-miR-3614-3p

UAGCCUUCAGAUCUUGGUGUUUU

>hsa-miR-3615

UCUCUCGGCUCCUCGCGGCUC

>hsa-miR-3617-5p

AAAGACAUAGUUGCAAGAUGGG

>hsa-miR-3617-3p

CAUCAGCACCCUAUGUCCUUUCU

>hsa-miR-3619-5p

UCAGCAGGCAGGCUGGUGCAGC

>hsa-miR-3619-3p

GGGACCAUCCUGCCUGCUGUGG

>hsa-miR-362-5p

AAUCCUUGGAACCUAGGUGUGAGU

>hsa-miR-362-3p

AACACACCUAUUCAAGGAUUCA

>hsa-miR-3620-5p

GUGGGCUGGGCUGGGCUGGGCC

>hsa-miR-3620-3p

UCACCCUGCAUCCCGCACCCAG

>hsa-miR-363-5p

CGGGUGGAUCACGAUGCAAUUU

>hsa-miR-363-3p

AAUUGCACGGUAUCCAUCUGUA

>hsa-miR-3648

AGCCGCGGGGAUCGCCGAGGG

>hsa-miR-3651

CAUAGCCCGGUCGCUGGUACAUGA

>hsa-miR-3654

GACUGGACAAGCUGAGGAA

>hsa-miR-3657

UGUGUCCCAUUAUUGGUGAUU

>hsa-miR-365a-5p

AGGGACUUUUGGGGGCAGAUGUG

>hsa-miR-365a-3p

UAAUGCCCCUAAAAAUCCUUAU

>hsa-miR-365b-5p

AGGGACUUUCAGGGGCAGCUGU

>hsa-miR-3661

UGACCUGGGACUCGGACAGCUG

>hsa-miR-3662

GAAAAUGAUGAGUAGUGACUGAUG

>hsa-miR-3667-5p

AAAGACCCAUUGAGGAGAAGGU

>hsa-miR-3667-3p

ACCUUCCUCUCCAUGGGUCUUU

>hsa-miR-367-3p

AAUUGCACUUUAGCAAUGGUGA

>hsa-miR-3677-5p

CAGUGGCCAGAGCCCUGCAGUG

>hsa-miR-3677-3p

CUCGUGGGCUCUGGCCACGGCC

>hsa-miR-3678-3p

CUGCAGAGUUUGUACGGACCGG

>hsa-miR-3679-5p

UGAGGAUAUGGCAGGGAAGGGGA

>hsa-miR-3680-5p

GACUCACUCACAGGAUUGUGCA

>hsa-miR-3680-3p

UUUUGCAUGACCCUGGGAGUAGG

>hsa-miR-3681-5p

UAGUGGAUGAUGCACUCUGUGC

>hsa-miR-3682-5p

CUACUUCUACCUGUGUUAUCAU

>hsa-miR-3682-3p

UGAUGAUACAGGUGGAGGUAG

>hsa-miR-3684

UUAGACCUAGUACACGUCCUU

>hsa-miR-3685

UUUCCUACCCUACCUGAAGACU

>hsa-miR-3686

AUCUGUAAGAGAAAGUAAAUGA

>hsa-miR-3688-5p

AGUGGCAAAGUCUUUCCAUAU

>hsa-miR-3688-3p

UAUGGAAAGACUUUGCCACUCU

>hsa-miR-369-3p

AAUAAUACAUGGUUGAUCUUU

>hsa-miR-3690

ACCUGGACCCAGCGUAGACAAAG

>hsa-miR-3691-5p

AGUGGAUGAUGGAGACUCGGUAC

>hsa-miR-3691-3p

ACCAAGUCUGCGUCAUCCUCUC

>hsa-miR-3692-5p

CCUGCUGGUCAGGAGUGGAUACUG

>hsa-miR-3692-3p

GUUCCACACUGACACUGCAGAAGU

>hsa-miR-370-3p

GCCUGCUGGGGUGGAACCUGGU

>hsa-miR-371a-5p

ACUCAAACUGUGGGGGCACU

>hsa-miR-371a-3p

AAGUGCCGCCAUCUUUUGAGUGU

>hsa-miR-371b-5p

ACUCAAAAGAUGGCGGCACUUU

>hsa-miR-371b-3p

AAGUGCCCCCACAGUUUGAGUGC

>hsa-miR-372-3p

AAAGUGCUGCGACAUUUGAGCGU

>hsa-miR-373-3p

GAAGUGCUUCGAUUUUGGGGUGU

>hsa-miR-374a-5p

UUAUAAUACAACCUGAUAAGUG

>hsa-miR-374a-3p

CUUAUCAGAUUGUAUUGUAAUU

>hsa-miR-374b-5p

AUAUAAUACAACCUGCUAAGUG

>hsa-miR-374b-3p

CUUAGCAGGUUGUAUUAUCAUU

>hsa-miR-374c-5p

AUAAUACAACCUGCUAAGUGCU

>hsa-miR-374c-3p

CACUUAGCAGGUUGUAUUAUAU

>hsa-miR-375-3p

UUUGUUCGUUCGGCUCGCGUGA

>hsa-miR-376a-3p

AUCAUAGAGGAAAAUCCACGU

>hsa-miR-376b-5p

CGUGGAUAUUCCUUCUAUGUUU

>hsa-miR-376c-5p

GGUGGAUAUUCCUUCUAUGUU

>hsa-miR-377-3p

AUCACACAAAGGCAACUUUUGU

>hsa-miR-378a-5p

CUCCUGACUCCAGGUCCUGUGU

>hsa-miR-378a-3p

ACUGGACUUGGAGUCAGAAGGC

>hsa-miR-378c

ACUGGACUUGGAGUCAGAAGAGUGG

>hsa-miR-378d

ACUGGACUUGGAGUCAGAAA

>hsa-miR-378e

ACUGGACUUGGAGUCAGGA

>hsa-miR-378f

ACUGGACUUGGAGCCAGAAG

>hsa-miR-378g

ACUGGGCUUGGAGUCAGAAG

>hsa-miR-378h

ACUGGACUUGGUGUCAGAUGG

>hsa-miR-378i

ACUGGACUAGGAGUCAGAAGG

>hsa-miR-379-5p

UGGUAGACUAUGGAACGUAGG

>hsa-miR-379-3p

UAUGUAACAUGGUCCACUAACU

>hsa-miR-380-5p

UGGUUGACCAUAGAACAUGCGC

>hsa-miR-381-3p

UAUACAAGGGCAAGCUCUCUGU

>hsa-miR-382-5p

GAAGUUGUUCGUGGUGGAUUCG

>hsa-miR-382-3p

AAUCAUUCACGGACAACACUU

>hsa-miR-383-5p

AGAUCAGAAGGUGAUUGUGGCU

>hsa-miR-3907

AGGUGCUCCAGGCUGGCUCACA

>hsa-miR-3909

UGUCCUCUAGGGCCUGCAGUCU

>hsa-miR-3910

AAAGGCAUAAAACCAAGACA

>hsa-miR-3911

UGUGUGGAUCCUGGAGGAGGCA

>hsa-miR-3912-3p

UAACGCAUAAUAUGGACAUGU

>hsa-miR-3913-5p

UUUGGGACUGAUCUUGAUGUCU

>hsa-miR-3913-3p

AGACAUCAAGAUCAGUCCCAAA

>hsa-miR-3916

AAGAGGAAGAAAUGGCUGGUUCUCAG

>hsa-miR-3917

GCUCGGACUGAGCAGGUGGG

>hsa-miR-3918

ACAGGGCCGCAGAUGGAGACU

>hsa-miR-3919

GCAGAGAACAAAGGACUCAGU

>hsa-miR-3922-5p

UCAAGGCCAGAGGUCCCACAGCA

>hsa-miR-3924

AUAUGUAUAUGUGACUGCUACU

>hsa-miR-3925-5p

AAGAGAACUGAAAGUGGAGCCU

>hsa-miR-3925-3p

ACUCCAGUUUUAGUUCUCUUG

>hsa-miR-3928-5p

UGAAGCUCUAAGGUUCCGCCUGC

>hsa-miR-3928-3p

GGAGGAACCUUGGAGCUUCGGC

>hsa-miR-3934-5p

UCAGGUGUGGAAACUGAGGCAG

>hsa-miR-3939

UACGCGCAGACCACAGGAUGUC

>hsa-miR-3940-5p

GUGGGUUGGGGCGGGCUCUG

>hsa-miR-3940-3p

CAGCCCGGAUCCCAGCCCACUU

>hsa-miR-3942-5p

AAGCAAUACUGUUACCUGAAAU

>hsa-miR-3942-3p

UUUCAGAUAACAGUAUUACAU

>hsa-miR-3943

UAGCCCCCAGGCUUCACUUGGCG

>hsa-miR-3944-5p

UGUGCAGCAGGCCAACCGAGA

>hsa-miR-3944-3p

UUCGGGCUGGCCUGCUGCUCCGG

>hsa-miR-409-5p

AGGUUACCCGAGCAACUUUGCAU

>hsa-miR-409-3p

GAAUGUUGCUCGGUGAACCCCU

>hsa-miR-410-3p

AAUAUAACACAGAUGGCCUGU

>hsa-miR-411-5p

UAGUAGACCGUAUAGCGUACG

>hsa-miR-412-5p

UGGUCGACCAGUUGGAAAGUAAU

>hsa-miR-421

AUCAACAGACAUUAAUUGGGCGC

>hsa-miR-422a

ACUGGACUUAGGGUCAGAAGGC

>hsa-miR-423-5p

UGAGGGGCAGAGAGCGAGACUUU

>hsa-miR-423-3p

AGCUCGGUCUGAGGCCCCUCAGU

>hsa-miR-424-5p

CAGCAGCAAUUCAUGUUUUGAA

>hsa-miR-424-3p

CAAAACGUGAGGCGCUGCUAU

>hsa-miR-425-5p

AAUGACACGAUCACUCCCGUUGA

>hsa-miR-425-3p

AUCGGGAAUGUCGUGUCCGCCC

>hsa-miR-4254

GCCUGGAGCUACUCCACCAUCUC

>hsa-miR-4286

ACCCCACUCCUGGUACC

>hsa-miR-429

UAAUACUGUCUGGUAAAACCGU

>hsa-miR-431-5p

UGUCUUGCAGGCCGUCAUGCA

>hsa-miR-432-5p

UCUUGGAGUAGGUCAUUGGGUGG

>hsa-miR-4326

UGUUCCUCUGUCUCCCAGAC

>hsa-miR-433-3p

AUCAUGAUGGGCUCCUCGGUGU

>hsa-miR-4420

GUCACUGAUGUCUGUAGCUGAG

>hsa-miR-4421

ACCUGUCUGUGGAAAGGAGCUA

>hsa-miR-4423-5p

AGUUGCCUUUUUGUUCCCAUGC

>hsa-miR-4423-3p

AUAGGCACCAAAAAGCAACAA

>hsa-miR-4424

AGAGUUAACUCAAAAUGGACUA

>hsa-miR-4425

UGUUGGGAUUCAGCAGGACCAU

>hsa-miR-4426

GAAGAUGGACGUACUUU

>hsa-miR-4431

GCGACUCUGAAAACUAGAAGGU

>hsa-miR-4433a-5p

CGUCCCACCCCCCACUCCUGU

>hsa-miR-4433a-3p

ACAGGAGUGGGGGUGGGACAU

>hsa-miR-4433b-5p

AUGUCCCACCCCCACUCCUGU

>hsa-miR-4433b-3p

CAGGAGUGGGGGGUGGGACGU

>hsa-miR-4435

AUGGCCAGAGCUCACACAGAGG

>hsa-miR-4437

UGGGCUCAGGGUACAAAGGUU

>hsa-miR-4439

GUGACUGAUACCUUGGAGGCAU

>hsa-miR-4440

UGUCGUGGGGCUUGCUGGCUUG

>hsa-miR-4445-3p

CACGGCAAAAGAAACAAUCCA

>hsa-miR-4449

CGUCCCGGGGCUGCGCGAGGCA

>hsa-miR-4454

GGAUCCGAGUCACGGCACCA

>hsa-miR-4457

UCACAAGGUAUUGACUGGCGUA

>hsa-miR-4458

AGAGGUAGGUGUGGAAGAA

>hsa-miR-4465

CUCAAGUAGUCUGACCAGGGGA

>hsa-miR-4466

GGGUGCGGGCCGGCGGGG

>hsa-miR-4467

UGGCGGCGGUAGUUAUGGGCUU

>hsa-miR-4469

GCUCCCUCUAGGGUCGCUCGGA

>hsa-miR-4470

UGGCAAACGUGGAAGCCGAGA

>hsa-miR-4471

UGGGAACUUAGUAGAGGUUUAA

>hsa-miR-4473

CUAGUGCUCUCCGUUACAAGUA

>hsa-miR-4477a

CUAUUAAGGACAUUUGUGAUUC

>hsa-miR-4477b

AUUAAGGACAUUUGUGAUUGAU

>hsa-miR-4479

CGCGCGGCCGUGCUCGGAGCAG

>hsa-miR-448

UUGCAUAUGUAGGAUGUCCCAU

>hsa-miR-4482-5p

AACCCAGUGGGCUAUGGAAAUG

>hsa-miR-4484

AAAAGGCGGGAGAAGCCCCA

>hsa-miR-4485-5p

ACCGCCUGCCCAGUGA

>hsa-miR-4485-3p

UAACGGCCGCGGUACCCUAA

>hsa-miR-4487

AGAGCUGGCUGAAGGGCAG

>hsa-miR-4488

AGGGGGCGGGCUCCGGCG

>hsa-miR-4498

UGGGCUGGCAGGGCAAGUGCUG

>hsa-miR-449a

UGGCAGUGUAUUGUUAGCUGGU

>hsa-miR-449b-5p

AGGCAGUGUAUUGUUAGCUGGC

>hsa-miR-449b-3p

CAGCCACAACUACCCUGCCACU

>hsa-miR-449c-5p

UAGGCAGUGUAUUGCUAGCGGCUGU

>hsa-miR-4501

UAUGUGACCUCGGAUGAAUCA

>hsa-miR-4504

UGUGACAAUAGAGAUGAACAUG

>hsa-miR-4505

AGGCUGGGCUGGGACGGA

>hsa-miR-4507

CUGGGUUGGGCUGGGCUGGG

>hsa-miR-450a-5p

UUUUGCGAUGUGUUCCUAAUAU

>hsa-miR-450a-1-3p

AUUGGGAACAUUUUGCAUGUAU

>hsa-miR-450a-2-3p

AUUGGGGACAUUUUGCAUUCAU

>hsa-miR-450b-5p

UUUUGCAAUAUGUUCCUGAAUA

>hsa-miR-450b-3p

UUGGGAUCAUUUUGCAUCCAUA

>hsa-miR-4511

GAAGAACUGUUGCAUUUGCCCU

>hsa-miR-4512

CAGGGCCUCACUGUAUCGCCCA

>hsa-miR-4515

AGGACUGGACUCCCGGCAGCCC

>hsa-miR-4517

AAAUAUGAUGAAACUCACAGCUGAG

>hsa-miR-4518

GCUCAGGGAUGAUAACUGUGCUGAGA

>hsa-miR-4519

CAGCAGUGCGCAGGGCUG

>hsa-miR-451a

AAACCGUUACCAUUACUGAGUU

>hsa-miR-452-5p

AACUGUUUGCAGAGGAAACUGA

>hsa-miR-4521

GCUAAGGAAGUCCUGUGCUCAG

>hsa-miR-4522

UGACUCUGCCUGUAGGCCGGU

>hsa-miR-4523

GACCGAGAGGGCCUCGGCUGU

>hsa-miR-4524a-3p

UGAGACAGGCUUAUGCUGCUAU

>hsa-miR-4524b-5p

AUAGCAGCAUAAGCCUGUCUC

>hsa-miR-4526

GCUGACAGCAGGGCUGGCCGCU

>hsa-miR-4527

UGGUCUGCAAAGAGAUGACUGU

>hsa-miR-4529-5p

AGGCCAUCAGCAGUCCAAUGAA

>hsa-miR-4536-5p

UGUGGUAGAUAUAUGCACGAU

>hsa-miR-4536-3p

UCGUGCAUAUAUCUACCACAU

>hsa-miR-454-5p

ACCCUAUCAAUAUUGUCUCUGC

>hsa-miR-454-3p

UAGUGCAAUAUUGCUUAUAGGGU

>hsa-miR-455-5p

UAUGUGCCUUUGGACUACAUCG

>hsa-miR-455-3p

GCAGUCCAUGGGCAUAUACAC

>hsa-miR-4632-3p

UGCCGCCCUCUCGCUGCUCUAG

>hsa-miR-4637

UACUAACUGCAGAUUCAAGUGA

>hsa-miR-4638-5p

ACUCGGCUGCGGUGGACAAGU

>hsa-miR-4638-3p

CCUGGACACCGCUCAGCCGGCCG

>hsa-miR-4639-5p

UUGCUAAGUAGGCUGAGAUUGA

>hsa-miR-4641

UGCCCAUGCCAUACUUUUGCCUCA

>hsa-miR-4642

AUGGCAUCGUCCCCUGGUGGCU

>hsa-miR-4644

UGGAGAGAGAAAAGAGACAGAAG

>hsa-miR-4645-3p

AGACAGUAGUUCUUGCCUGGUU

>hsa-miR-4646-5p

ACUGGGAAGAGGAGCUGAGGGA

>hsa-miR-4646-3p

AUUGUCCCUCUCCCUUCCCAG

>hsa-miR-4647

GAAGAUGGUGCUGUGCUGAGGAA

>hsa-miR-4648

UGUGGGACUGCAAAUGGGAG

>hsa-miR-4651

CGGGGUGGGUGAGGUCGGGC

>hsa-miR-4654

UGUGGGAUCUGGAGGCAUCUGG

>hsa-miR-4657

AAUGUGGAAGUGGUCUGAGGCAU

>hsa-miR-4658

GUGAGUGUGGAUCCUGGAGGAAU

>hsa-miR-4659a-5p

CUGCCAUGUCUAAGAAGAAAAC

>hsa-miR-4659a-3p

UUUCUUCUUAGACAUGGCAACG

>hsa-miR-4659b-5p

UUGCCAUGUCUAAGAAGAA

>hsa-miR-4659b-3p

UUUCUUCUUAGACAUGGCAGCU

>hsa-miR-4660

UGCAGCUCUGGUGGAAAAUGGAG

>hsa-miR-4661-5p

AACUAGCUCUGUGGAUCCUGAC

>hsa-miR-4661-3p

CAGGAUCCACAGAGCUAGUCCA

>hsa-miR-4662a-5p

UUAGCCAAUUGUCCAUCUUUAG

>hsa-miR-4662a-3p

AAAGAUAGACAAUUGGCUAAAU

>hsa-miR-4662b

AAAGAUGGACAAUUGGCUAAAU

>hsa-miR-4664-5p

UGGGGUGCCCACUCCGCAAGUU

>hsa-miR-4664-3p

CUUCCGGUCUGUGAGCCCCGUC

>hsa-miR-4665-5p

CUGGGGGACGCGUGAGCGCGAGC

>hsa-miR-4666a-5p

AUACAUGUCAGAUUGUAUGCC

>hsa-miR-4666a-3p

CAUACAAUCUGACAUGUAUUU

>hsa-miR-4667-5p

ACUGGGGAGCAGAAGGAGAACC

>hsa-miR-4667-3p

UCCCUCCUUCUGUCCCCACAG

>hsa-miR-4669

UGUGUCCGGGAAGUGGAGGAGG

>hsa-miR-4670-5p

AAGCGACCAUGAUGUAACUUCA

>hsa-miR-4670-3p

UGAAGUUACAUCAUGGUCGCUU

>hsa-miR-4671-5p

ACCGAAGACUGUGCGCUAAUCU

>hsa-miR-4671-3p

UUAGUGCAUAGUCUUUGGUCU

>hsa-miR-4672

UUACACAGCUGGACAGAGGCA

>hsa-miR-4673

UCCAGGCAGGAGCCGGACUGGA

>hsa-miR-4676-5p

GAGCCAGUGGUGAGACAGUGA

>hsa-miR-4676-3p

CACUGUUUCACCACUGGCUCUU

>hsa-miR-4677-5p

UUGUUCUUUGGUCUUUCAGCCA

>hsa-miR-4677-3p

UCUGUGAGACCAAAGAACUACU

>hsa-miR-4679

UCUGUGAUAGAGAUUCUUUGCU

>hsa-miR-4680-5p

AGAACUCUUGCAGUCUUAGAUGU

>hsa-miR-4680-3p

UCUGAAUUGUAAGAGUUGUUA

>hsa-miR-4683

UGGAGAUCCAGUGCUCGCCCGAU

>hsa-miR-4684-3p

UGUUGCAAGUCGGUGGAGACGU

>hsa-miR-4685-3p

UCUCCCUUCCUGCCCUGGCUAG

>hsa-miR-4687-5p

CAGCCCUCCUCCCGCACCCAAA

>hsa-miR-4687-3p

UGGCUGUUGGAGGGGGCAGGC

>hsa-miR-4688

UAGGGGCAGCAGAGGACCUGGG

>hsa-miR-4689

UUGAGGAGACAUGGUGGGGGCC

>hsa-miR-4690-5p

GAGCAGGCGAGGCUGGGCUGAA

>hsa-miR-4690-3p

GCAGCCCAGCUGAGGCCUCUG

>hsa-miR-4691-3p

CCAGCCACGGACUGAGAGUGCAU

>hsa-miR-4695-3p

UGAUCUCACCGCUGCCUCCUUC

>hsa-miR-4699-5p

AGAAGAUUGCAGAGUAAGUUCC

>hsa-miR-4699-3p

AAUUUACUCUGCAAUCUUCUCC

>hsa-miR-4700-5p

UCUGGGGAUGAGGACAGUGUGU

>hsa-miR-4703-5p

UAGCAAUACAGUACAAAUAUAGU

>hsa-miR-4703-3p

UGUAGUUGUAUUGUAUUGCCAC

>hsa-miR-4706

AGCGGGGAGGAAGUGGGCGCUGCUU

>hsa-miR-4707-5p

GCCCCGGCGCGGGCGGGUUCUGG

>hsa-miR-4707-3p

AGCCCGCCCCAGCCGAGGUUCU

>hsa-miR-4709-5p

ACAACAGUGACUUGCUCUCCAA

>hsa-miR-4709-3p

UUGAAGAGGAGGUGCUCUGUAGC

>hsa-miR-4711-3p

CGUGUCUUCUGGCUUGAU

>hsa-miR-4713-5p

UUCUCCCACUACCAGGCUCCCA

>hsa-miR-4713-3p

UGGGAUCCAGACAGUGGGAGAA

>hsa-miR-4715-5p

AAGUUGGCUGCAGUUAAGGUGG

>hsa-miR-4717-5p

UAGGCCACAGCCACCCAUGUGU

>hsa-miR-4717-3p

ACACAUGGGUGGCUGUGGCCU

>hsa-miR-4723-5p

UGGGGGAGCCAUGAGAUAAGAGCA

>hsa-miR-4724-5p

AACUGAACCAGGAGUGAGCUUCG

>hsa-miR-4725-5p

AGACCCUGCAGCCUUCCCACC

>hsa-miR-4725-3p

UGGGGAAGGCGUCAGUGUCGGG

>hsa-miR-4726-5p

AGGGCCAGAGGAGCCUGGAGUGG

>hsa-miR-4727-5p

AUCUGCCAGCUUCCACAGUGG

>hsa-miR-4727-3p

AUAGUGGGAAGCUGGCAGAUUC

>hsa-miR-4729

UCAUUUAUCUGUUGGGAAGCUA

>hsa-miR-4732-5p

UGUAGAGCAGGGAGCAGGAAGCU

>hsa-miR-4732-3p

GCCCUGACCUGUCCUGUUCUG

>hsa-miR-4733-3p

CCACCAGGUCUAGCAUUGGGAU

>hsa-miR-4734

GCUGCGGGCUGCGGUCAGGGCG

>hsa-miR-4737

AUGCGAGGAUGCUGACAGUG

>hsa-miR-4738-5p

ACCAGCGCGUUUUCAGUUUCAU

>hsa-miR-4738-3p

UGAAACUGGAGCGCCUGGAGGA

>hsa-miR-4739

AAGGGAGGAGGAGCGGAGGGGCCCU

>hsa-miR-4740-5p

AGGACUGAUCCUCUCGGGCAGG

>hsa-miR-4741

CGGGCUGUCCGGAGGGGUCGGCU

>hsa-miR-4742-5p

UCAGGCAAAGGGAUAUUUACAGA

>hsa-miR-4742-3p

UCUGUAUUCUCCUUUGCCUGCAG

>hsa-miR-4743-5p

UGGCCGGAUGGGACAGGAGGCAU

>hsa-miR-4744

UCUAAAGACUAGACUUCGCUAUG

>hsa-miR-4745-3p

UGGCCCGGCGACGUCUCACGGUC

>hsa-miR-4746-5p

CCGGUCCCAGGAGAACCUGCAGA

>hsa-miR-4747-5p

AGGGAAGGAGGCUUGGUCUUAG

>hsa-miR-4748

GAGGUUUGGGGAGGAUUUGCU

>hsa-miR-4749-3p

CGCCCCUCCUGCCCCCACAG

>hsa-miR-4750-5p

CUCGGGCGGAGGUGGUUGAGUG

>hsa-miR-4753-5p

CAAGGCCAAAGGAAGAGAACAG

>hsa-miR-4753-3p

UUCUCUUUCUUUAGCCUUGUGU

>hsa-miR-4754

AUGCGGACCUGGGUUAGCGGAGU

>hsa-miR-4755-5p

UUUCCCUUCAGAGCCUGGCUUU

>hsa-miR-4755-3p

AGCCAGGCUCUGAAGGGAAAGU

>hsa-miR-4758-3p

UGCCCCACCUGCUGACCACCCUC

>hsa-miR-4761-5p

ACAAGGUGUGCAUGCCUGACC

>hsa-miR-4761-3p

GAGGGCAUGCGCACUUUGUCC

>hsa-miR-4762-5p

CCAAAUCUUGAUCAGAAGCCU

>hsa-miR-4762-3p

CUUCUGAUCAAGAUUUGUGGUG

>hsa-miR-4763-5p

CGCCUGCCCAGCCCUCCUGCU

>hsa-miR-4765

UGAGUGAUUGAUAGCUAUGUUC

>hsa-miR-4766-5p

UCUGAAAGAGCAGUUGGUGUU

>hsa-miR-4766-3p

AUAGCAAUUGCUCUUUUGGAA

>hsa-miR-4767

CGCGGGCGCUCCUGGCCGCCGCC

>hsa-miR-4769-5p

GGUGGGAUGGAGAGAAGGUAUGAG

>hsa-miR-4769-3p

UCUGCCAUCCUCCCUCCCCUAC

>hsa-miR-4771

AGCAGACUUGACCUACAAUUA

>hsa-miR-4772-5p

UGAUCAGGCAAAAUUGCAGACU

>hsa-miR-4772-3p

CCUGCAACUUUGCCUGAUCAGA

>hsa-miR-4773

CAGAACAGGAGCAUAGAAAGGC

>hsa-miR-4774-5p

UCUGGUAUGUAGUAGGUAAUAA

>hsa-miR-4775

UUAAUUUUUUGUUUCGGUCACU

>hsa-miR-4777-5p

UUCUAGAUGAGAGAUAUAUAUA

>hsa-miR-4777-3p

AUACCUCAUCUAGAAUGCUGUA

>hsa-miR-4779

UAGGAGGGAAUAGUAAAAGCAG

>hsa-miR-4781-5p

UAGCGGGGAUUCCAAUAUUGG

>hsa-miR-4781-3p

AAUGUUGGAAUCCUCGCUAGAG

>hsa-miR-4782-5p

UUCUGGAUAUGAAGACAAUCAA

>hsa-miR-4783-3p

CCCCGGUGUUGGGGCGCGUCUGC

>hsa-miR-4784

UGAGGAGAUGCUGGGACUGA

>hsa-miR-4785

AGAGUCGGCGACGCCGCCAGC

>hsa-miR-4786-5p

UGAGACCAGGACUGGAUGCACC

>hsa-miR-4786-3p

UGAAGCCAGCUCUGGUCUGGGC

>hsa-miR-4787-3p

GAUGCGCCGCCCACUGCCCCGCGC

>hsa-miR-4789-5p

GUAUACACCUGAUAUGUGUAUG

>hsa-miR-4791

UGGAUAUGAUGACUGAAA

>hsa-miR-4793-3p

UCUGCACUGUGAGUUGGCUGGCU

>hsa-miR-4794

UCUGGCUAUCUCACGAGACUGU

>hsa-miR-4795-5p

AGAAGUGGCUAAUAAUAUUGA

>hsa-miR-4795-3p

AUAUUAUUAGCCACUUCUGGAU

>hsa-miR-4796-5p

UGUCUAUACUCUGUCACUUUAC

>hsa-miR-4796-3p

UAAAGUGGCAGAGUAUAGACAC

>hsa-miR-4797-5p

GACAGAGUGCCACUUACUGAA

>hsa-miR-4797-3p

UCUCAGUAAGUGGCACUCUGU

>hsa-miR-4798-5p

UUCGGUAUACUUUGUGAAUUGG

>hsa-miR-4799-5p

AUCUAAAUGCAGCAUGCCAGUC

>hsa-miR-4799-3p

ACUGGCAUGCUGCAUUUAUAUA

>hsa-miR-4800-3p

CAUCCGUCCGUCUGUCCAC

>hsa-miR-4802-5p

UAUGGAGGUUCUAGACCAUGUU

>hsa-miR-4802-3p

UACAUGGAUGGAAACCUUCAAGC

>hsa-miR-4803

UAACAUAAUAGUGUGGAUUGA

>hsa-miR-4804-3p

UGCUUAACCUUGCCCUCGAAA

>hsa-miR-483-5p

AAGACGGGAGGAAAGAAGGGAG

>hsa-miR-483-3p

UCACUCCUCUCCUCCCGUCUU

>hsa-miR-484

UCAGGCUCAGUCCCCUCCCGAU

>hsa-miR-485-5p

AGAGGCUGGCCGUGAUGAAUUC

>hsa-miR-486-5p

UCCUGUACUGAGCUGCCCCGAG

>hsa-miR-486-3p

CGGGGCAGCUCAGUACAGGAU

>hsa-miR-487b-3p

AAUCGUACAGGGUCAUCCACUU

>hsa-miR-489-3p

GUGACAUCACAUAUACGGCAGC

>hsa-miR-490-3p

CAACCUGGAGGACUCCAUGCUG

>hsa-miR-491-5p

AGUGGGGAACCCUUCCAUGAGG

>hsa-miR-491-3p

CUUAUGCAAGAUUCCCUUCUAC

>hsa-miR-493-5p

UUGUACAUGGUAGGCUUUCAUU

>hsa-miR-493-3p

UGAAGGUCUACUGUGUGCCAGG

>hsa-miR-494-3p

UGAAACAUACACGGGAAACCUC

>hsa-miR-495-3p

AAACAAACAUGGUGCACUUCUU

>hsa-miR-497-5p

CAGCAGCACACUGUGGUUUGU

>hsa-miR-4999-5p

UGCUGUAUUGUCAGGUAGUGA

>hsa-miR-4999-3p

UCACUACCUGACAAUACAGU

>hsa-miR-499a-5p

UUAAGACUUGCAGUGAUGUUU

>hsa-miR-499a-3p

AACAUCACAGCAAGUCUGUGCU

>hsa-miR-499b-5p

ACAGACUUGCUGUGAUGUUCA

>hsa-miR-499b-3p

AACAUCACUGCAAGUCUUAACA

>hsa-miR-5000-5p

CAGUUCAGAAGUGUUCCUGAGU

>hsa-miR-5000-3p

UCAGGACACUUCUGAACUUGGA

>hsa-miR-5001-5p

AGGGCUGGACUCAGCGGCGGAGCU

>hsa-miR-5001-3p

UUCUGCCUCUGUCCAGGUCCUU

>hsa-miR-5002-5p

AAUUUGGUUUCUGAGGCACUUAGU

>hsa-miR-5004-3p

CUUGGAUUUUCCUGGGCCUCAG

>hsa-miR-5008-5p

UGAGGCCCUUGGGGCACAGUGG

>hsa-miR-5008-3p

CCUGUGCUCCCAGGGCCUCGC

>hsa-miR-5009-5p

UUGGACUUUUUCAGAUUUGGGGAU

>hsa-miR-500a-5p

UAAUCCUUGCUACCUGGGUGAGA

>hsa-miR-500a-3p

AUGCACCUGGGCAAGGAUUCUG

>hsa-miR-500b-5p

AAUCCUUGCUACCUGGGU

>hsa-miR-500b-3p

GCACCCAGGCAAGGAUUCUG

>hsa-miR-501-5p

AAUCCUUUGUCCCUGGGUGAGA

>hsa-miR-501-3p

AAUGCACCCGGGCAAGGAUUCU

>hsa-miR-5010-5p

AGGGGGAUGGCAGAGCAAAAUU

>hsa-miR-5010-3p

UUUUGUGUCUCCCAUUCCCCAG

>hsa-miR-502-5p

AUCCUUGCUAUCUGGGUGCUA

>hsa-miR-502-3p

AAUGCACCUGGGCAAGGAUUCA

>hsa-miR-503-5p

UAGCAGCGGGAACAGUUCUGCAG

>hsa-miR-503-3p

GGGGUAUUGUUUCCGCUGCCAGG

>hsa-miR-504-5p

AGACCCUGGUCUGCACUCUAUC

>hsa-miR-505-5p

GGGAGCCAGGAAGUAUUGAUGU

>hsa-miR-505-3p

CGUCAACACUUGCUGGUUUCCU

>hsa-miR-506-3p

UAAGGCACCCUUCUGAGUAGA

>hsa-miR-508-3p

UGAUUGUAGCCUUUUGGAGUAGA

>hsa-miR-5087

GGGUUUGUAGCUUUGCUGGCAUG

>hsa-miR-5089-5p

GUGGGAUUUCUGAGUAGCAUC

>hsa-miR-5089-3p

AUGCUACUCGGAAAUCCCACUGA

>hsa-miR-509-5p

UACUGCAGACAGUGGCAAUCA

>hsa-miR-509-3p

UGAUUGGUACGUCUGUGGGUAG

>hsa-miR-509-3-5p

UACUGCAGACGUGGCAAUCAUG

>hsa-miR-5090

CCGGGGCAGAUUGGUGUAGGGUG

>hsa-miR-5091

ACGGAGACGACAAGACUGUGCUG

>hsa-miR-5092

AAUCCACGCUGAGCUUGGCAUC

>hsa-miR-5094

AAUCAGUGAAUGCCUUGAACCU

>hsa-miR-5100

UUCAGAUCCCAGCGGUGCCUCU

>hsa-miR-511-5p

GUGUCUUUUGCUCUGCAGUCA

>hsa-miR-511-3p

AAUGUGUAGCAAAAGACAGA

>hsa-miR-512-3p

AAGUGCUGUCAUAGCUGAGGUC

>hsa-miR-513a-5p

UUCACAGGGAGGUGUCAU

>hsa-miR-514a-3p

AUUGACACUUCUGUGAGUAGA

>hsa-miR-515-5p

UUCUCCAAAAGAAAGCACUUUCUG

>hsa-miR-516a-5p

UUCUCGAGGAAAGAAGCACUUUC

>hsa-miR-516b-5p

AUCUGGAGGUAAGAAGCACUUU

>hsa-miR-517a-3p

AUCGUGCAUCCCUUUAGAGUGU

>hsa-miR-517c-3p

AUCGUGCAUCCUUUUAGAGUGU

>hsa-miR-5187-5p

UGGGAUGAGGGAUUGAAGUGGA

>hsa-miR-5188

AAUCGGACCCAUUUAAACCGGAG

>hsa-miR-5189-5p

UCUGGGCACAGGCGGAUGGACAGG

>hsa-miR-5189-3p

UGCCAACCGUCAGAGCCCAGA

>hsa-miR-518b

CAAAGCGCUCCCCUUUAGAGGU

>hsa-miR-518f-5p

CUCUAGAGGGAAGCACUUUCUC

>hsa-miR-518f-3p

GAAAGCGCUUCUCUUUAGAGG

>hsa-miR-5193

UCCUCCUCUACCUCAUCCCAGU

>hsa-miR-5196-3p

UCAUCCUCGUCUCCCUCCCAG

>hsa-miR-519a-3p

AAAGUGCAUCCUUUUAGAGUGU

>hsa-miR-519c-5p

CUCUAGAGGGAAGCGCUUUCUG

>hsa-miR-519d-3p

CAAAGUGCCUCCCUUUAGAGUG

>hsa-miR-520a-3p

AAAGUGCUUCCCUUUGGACUGU

>hsa-miR-520e-3p

AAAGUGCUUCCUUUUUGAGGG

>hsa-miR-520f-3p

AAGUGCUUCCUUUUAGAGGGUU

>hsa-miR-520g-3p

ACAAAGUGCUUCCCUUUAGAGUGU

>hsa-miR-525-5p

CUCCAGAGGGAUGCACUUUCU

>hsa-miR-526a-5p

CUCUAGAGGGAAGCACUUUCUG

>hsa-miR-526b-5p

CUCUUGAGGGAAGCACUUUCUGU

>hsa-miR-532-5p

CAUGCCUUGAGUGUAGGACCGU

>hsa-miR-532-3p

CCUCCCACACCCAAGGCUUGCA

>hsa-miR-539-5p

GGAGAAAUUAUCCUUGGUGUGU

>hsa-miR-541-5p

AAAGGAUUCUGCUGUCGGUCCCACU

>hsa-miR-542-5p

UCGGGGAUCAUCAUGUCACGAGA

>hsa-miR-542-3p

UGUGACAGAUUGAUAACUGAAA

>hsa-miR-543

AAACAUUCGCGGUGCACUUCUU

>hsa-miR-545-5p

UCAGUAAAUGUUUAUUAGAUGA

>hsa-miR-545-3p

UCAGCAAACAUUUAUUGUGUGC

>hsa-miR-548a-3p

CAAAACUGGCAAUUACUUUUGC

>hsa-miR-548a-5p

AAAAGUAAUUGCGAGUUUUACC

>hsa-miR-548ab

AAAAGUAAUUGUGGAUUUUGCU

>hsa-miR-548ac

CAAAAACCGGCAAUUACUUUUG

>hsa-miR-548ad-5p

AAAAGUAAUUGUGGUUUUUG

>hsa-miR-548ae-3p

CAAAAACUGCAAUUACUUUCA

>hsa-miR-548ag

AAAGGUAAUUGUGGUUUCUGC

>hsa-miR-548ah-3p

CAAAAACUGCAGUUACUUUUGC

>hsa-miR-548aj-3p

UAAAAACUGCAAUUACUUUUA

>hsa-miR-548ak

AAAAGUAACUGCGGUUUUUGA

>hsa-miR-548al

AACGGCAAUGACUUUUGUACCA

>hsa-miR-548am-3p

CAAAAACUGCAGUUACUUUUGU

>hsa-miR-548an

AAAAGGCAUUGUGGUUUUUG

>hsa-miR-548ao-3p

AAAGACCGUGACUACUUUUGCA

>hsa-miR-548ap-5p

AAAAGUAAUUGCGGUCUUU

>hsa-miR-548ap-3p

AAAAACCACAAUUACUUUU

>hsa-miR-548aq-5p

GAAAGUAAUUGCUGUUUUUGCC

>hsa-miR-548aq-3p

CAAAAACUGCAAUUACUUUUGC

>hsa-miR-548ar-5p

AAAAGUAAUUGCAGUUUUUGC

>hsa-miR-548ar-3p

UAAAACUGCAGUUAUUUUUGC

>hsa-miR-548at-5p

AAAAGUUAUUGCGGUUUUGGCU

>hsa-miR-548at-3p

CAAAACCGCAGUAACUUUUGU

>hsa-miR-548au-5p

AAAAGUAAUUGCGGUUUUUGC

>hsa-miR-548av-3p

AAAACUGCAGUUACUUUUGC

>hsa-miR-548aw

GUGCAAAAGUCAUCACGGUU

>hsa-miR-548ax

AGAAGUAAUUGCGGUUUUGCCA

>hsa-miR-548ay-3p

CAAAACCGCGAUUACUCUUGCA

>hsa-miR-548az-5p

CAAAAGUGAUUGUGGUUUUUGC

>hsa-miR-548az-3p

AAAAACUGCAAUCACUUUUGC

>hsa-miR-548b-5p

AAAAGUAAUUGUGGUUUUGGCC

>hsa-miR-548b-3p

CAAGAACCUCAGUUGCUUUUGU

>hsa-miR-548ba

AAAGGUAACUGUGAUUUUUGCU

>hsa-miR-548bc

AAAAACUGUGAUUACUUUUGC

>hsa-miR-548c-5p

AAAAGUAAUUGCGGUUUUUGCC

>hsa-miR-548d-5p

AAAAGUAAUUGUGGUUUUUGCC

>hsa-miR-548d-3p

CAAAAACCACAGUUUCUUUUGC

>hsa-miR-548e-5p

CAAAAGCAAUCGCGGUUUUUGC

>hsa-miR-548e-3p

AAAAACUGAGACUACUUUUGCA

>hsa-miR-548f-5p

UGCAAAAGUAAUCACAGUUUUU

>hsa-miR-548f-3p

AAAAACUGUAAUUACUUUU

>hsa-miR-548g-5p

UGCAAAAGUAAUUGCAGUUUUUG

>hsa-miR-548g-3p

AAAACUGUAAUUACUUUUGUAC

>hsa-miR-548h-5p

AAAAGUAAUCGCGGUUUUUGUC

>hsa-miR-548h-3p

CAAAAACCGCAAUUACUUUUGCA

>hsa-miR-548i

AAAAGUAAUUGCGGAUUUUGCC

>hsa-miR-548j-5p

AAAAGUAAUUGCGGUCUUUGGU

>hsa-miR-548j-3p

CAAAAACUGCAUUACUUUUGC

>hsa-miR-548k

AAAAGUACUUGCGGAUUUUGCU

>hsa-miR-548l

AAAAGUAUUUGCGGGUUUUGUC

>hsa-miR-548n

CAAAAGUAAUUGUGGAUUUUGU

>hsa-miR-548o-3p

CCAAAACUGCAGUUACUUUUGC

>hsa-miR-548p

UAGCAAAAACUGCAGUUACUUU

>hsa-miR-548q

GCUGGUGCAAAAGUAAUGGCGG

>hsa-miR-548s

AUGGCCAAAACUGCAGUUAUUUU

>hsa-miR-548t-5p

CAAAAGUGAUCGUGGUUUUUG

>hsa-miR-548t-3p

AAAAACCACAAUUACUUUUGCACCA

>hsa-miR-548u

CAAAGACUGCAAUUACUUUUGCG

>hsa-miR-548v

AGCUACAGUUACUUUUGCACCA

>hsa-miR-548w

AAAAGUAACUGCGGUUUUUGCCU

>hsa-miR-548x-3p

UAAAAACUGCAAUUACUUUC

>hsa-miR-549a-5p

AGCUCAUCCAUAGUUGUCACUG

>hsa-miR-549a-3p

UGACAACUAUGGAUGAGCUCU

>hsa-miR-550a-5p

AGUGCCUGAGGGAGUAAGAGCCC

>hsa-miR-550a-3p

UGUCUUACUCCCUCAGGCACAU

>hsa-miR-550a-3-5p

AGUGCCUGAGGGAGUAAGAG

>hsa-miR-550b-2-5p

AUGUGCCUGAGGGAGUAAGACA

>hsa-miR-550b-3p

UCUUACUCCCUCAGGCACUG

>hsa-miR-551a

GCGACCCACUCUUGGUUUCCA

>hsa-miR-552-5p

GUUUAACCUUUUGCCUGUUGG

>hsa-miR-552-3p

AACAGGUGACUGGUUAGACAA

>hsa-miR-556-5p

GAUGAGCUCAUUGUAAUAUGAG

>hsa-miR-556-3p

AUAUUACCAUUAGCUCAUCUUU

>hsa-miR-5579-5p

UAUGGUACUCCUUAAGCUAAC

>hsa-miR-5579-3p

UUAGCUUAAGGAGUACCAGAUC

>hsa-miR-5581-3p

UUCCAUGCCUCCUAGAAGUUCC

>hsa-miR-5582-3p

UAAAACUUUAAGUGUGCCUAGG

>hsa-miR-5585-5p

UGAAGUACCAGCUACUCGAGAG

>hsa-miR-5585-3p

CUGAAUAGCUGGGACUACAGGU

>hsa-miR-5586-5p

UAUCCAGCUUGUUACUAUAUGC

>hsa-miR-5586-3p

CAGAGUGACAAGCUGGUUAAAG

>hsa-miR-5587-5p

AUGGUCACCUCCGGGACU

>hsa-miR-5587-3p

GCCCCGGGCAGUGUGAUCAUC

>hsa-miR-5588-5p

ACUGGCAUUAGUGGGACUUUU

>hsa-miR-5680

GAGAAAUGCUGGACUAAUCUGC

>hsa-miR-5682

GUAGCACCUUGCAGGAUAAGGU

>hsa-miR-5684

AACUCUAGCCUGAGCAACAG

>hsa-miR-5685

ACAGCCCAGCAGUUAUCACGGG

>hsa-miR-5690

UCAGCUACUACCUCUAUUAGG

>hsa-miR-5692c

AAUAAUAUCACAGUAGGUGUAC

>hsa-miR-5693

GCAGUGGCUCUGAAAUGAACUC

>hsa-miR-5695

ACUCCAAGAAGAAUCUAGACAG

>hsa-miR-5696

CUCAUUUAAGUAGUCUGAUGCC

>hsa-miR-5697

UCAAGUAGUUUCAUGAUAAAGG

>hsa-miR-5698

UGGGGGAGUGCAGUGAUUGUGG

>hsa-miR-5699-5p

UGCCCCAACAAGGAAGGACAAG

>hsa-miR-570-5p

AAAGGUAAUUGCAGUUUUUCCC

>hsa-miR-570-3p

CGAAAACAGCAAUUACCUUUGC

>hsa-miR-5701

UUAUUGUCACGUUCUGAUU

>hsa-miR-5704

UUAGGCCAUCAUCCCAUUAUGC

>hsa-miR-5706

UUCUGGAUAACAUGCUGAAGCU

>hsa-miR-572

GUCCGCUCGGCGGUGGCCCA

>hsa-miR-573

CUGAAGUGAUGUGUAACUGAUCAG

>hsa-miR-574-5p

UGAGUGUGUGUGUGUGAGUGUGU

>hsa-miR-574-3p

CACGCUCAUGCACACACCCACA

>hsa-miR-576-5p

AUUCUAAUUUCUCCACGUCUUU

>hsa-miR-576-3p

AAGAUGUGGAAAAAUUGGAAUC

>hsa-miR-577

UAGAUAAAAUAUUGGUACCUG

>hsa-miR-578

CUUCUUGUGCUCUAGGAUUGU

>hsa-miR-579-5p

UCGCGGUUUGUGCCAGAUGACG

>hsa-miR-579-3p

UUCAUUUGGUAUAAACCGCGAUU

>hsa-miR-580-3p

UUGAGAAUGAUGAAUCAUUAGG

>hsa-miR-581

UCUUGUGUUCUCUAGAUCAGU

>hsa-miR-582-5p

UUACAGUUGUUCAACCAGUUACU

>hsa-miR-582-3p

UAACUGGUUGAACAACUGAACC

>hsa-miR-583

CAAAGAGGAAGGUCCCAUUAC

>hsa-miR-584-5p

UUAUGGUUUGCCUGGGACUGAG

>hsa-miR-584-3p

UCAGUUCCAGGCCAACCAGGCU

>hsa-miR-585-3p

UGGGCGUAUCUGUAUGCUA

>hsa-miR-586

UAUGCAUUGUAUUUUUAGGUCC

>hsa-miR-588

UUGGCCACAAUGGGUUAGAAC

>hsa-miR-589-5p

UGAGAACCACGUCUGCUCUGAG

>hsa-miR-589-3p

UCAGAACAAAUGCCGGUUCCCAGA

>hsa-miR-590-5p

GAGCUUAUUCAUAAAAGUGCAG

>hsa-miR-590-3p

UAAUUUUAUGUAUAAGCUAGU

>hsa-miR-592

UUGUGUCAAUAUGCGAUGAUGU

>hsa-miR-597-5p

UGUGUCACUCGAUGACCACUGU

>hsa-miR-597-3p

UGGUUCUCUUGUGGCUCAAGCGU

>hsa-miR-598-5p

GCGGUGAUCCCGAUGGUGUGAGC

>hsa-miR-598-3p

UACGUCAUCGUUGUCAUCGUCA

>hsa-miR-600

ACUUACAGACAAGAGCCUUGCUC

>hsa-miR-605-5p

UAAAUCCCAUGGUGCCUUCUCCU

>hsa-miR-607

GUUCAAAUCCAGAUCUAUAAC

>hsa-miR-6084

UUCCGCCAGUCGGUGGCCGG

>hsa-miR-612

GCUGGGCAGGGCUUCUGAGCUCCUU

>hsa-miR-615-3p

UCCGAGCCUGGGUCUCCCUCUU

>hsa-miR-616-5p

ACUCAAAACCCUUCAGUGACUU

>hsa-miR-616-3p

AGUCAUUGGAGGGUUUGAGCAG

>hsa-miR-618

AAACUCUACUUGUCCUUCUGAGU

>hsa-miR-619-5p

GCUGGGAUUACAGGCAUGAGCC

>hsa-miR-624-5p

UAGUACCAGUACCUUGUGUUCA

>hsa-miR-624-3p

CACAAGGUAUUGGUAUUACCU

>hsa-miR-625-5p

AGGGGGAAAGUUCUAUAGUCC

>hsa-miR-625-3p

GACUAUAGAACUUUCCCCCUCA

>hsa-miR-627-5p

GUGAGUCUCUAAGAAAAGAGGA

>hsa-miR-627-3p

UCUUUUCUUUGAGACUCACU

>hsa-miR-628-5p

AUGCUGACAUAUUUACUAGAGG

>hsa-miR-628-3p

UCUAGUAAGAGUGGCAGUCGA

>hsa-miR-629-5p

UGGGUUUACGUUGGGAGAACU

>hsa-miR-629-3p

GUUCUCCCAACGUAAGCCCAGC

>hsa-miR-632

GUGUCUGCUUCCUGUGGGA

>hsa-miR-635

ACUUGGGCACUGAAACAAUGUCC

>hsa-miR-636

UGUGCUUGCUCGUCCCGCCCGCA

>hsa-miR-641

AAAGACAUAGGAUAGAGUCACCUC

>hsa-miR-642a-5p

GUCCCUCUCCAAAUGUGUCUUG

>hsa-miR-642a-3p

AGACACAUUUGGAGAGGGAACC

>hsa-miR-642b-5p

GGUUCCCUCUCCAAAUGUGUCU

>hsa-miR-642b-3p

AGACACAUUUGGAGAGGGACCC

>hsa-miR-643

ACUUGUAUGCUAGCUCAGGUAG

>hsa-miR-6500-5p

AGGAGCUAUCCACUCCAGGUGUCC

>hsa-miR-6500-3p

ACACUUGUUGGGAUGACCUGC

>hsa-miR-6501-5p

AGUUGCCAGGGCUGCCUUUGGU

>hsa-miR-6501-3p

CCAGAGCAGCCUGCGGUAACAGU

>hsa-miR-6502-5p

AGCUCUAGAAAGAUUGUUGACC

>hsa-miR-6503-5p

AGGUCUGCAUUCAAAUCCCCAGA

>hsa-miR-6503-3p

GGGACUAGGAUGCAGACCUCC

>hsa-miR-6504-5p

UCUGGCUGUGCUGUAAUGCAG

>hsa-miR-6506-5p

ACUGGGAUGUCACUGAAUAUGGU

>hsa-miR-6506-3p

UCGUAUCAGAGAUUCCAGACAC

>hsa-miR-6509-5p

AUUAGGUAGUGGCAGUGGAAC

>hsa-miR-6509-3p

UUCCACUGCCACUACCUAAUUU

>hsa-miR-651-5p

UUUAGGAUAAGCUUGACUUUUG

>hsa-miR-6510-3p

CACCGACUCUGUCUCCUGCAG

>hsa-miR-6511a-5p

CAGGCAGAAGUGGGGCUGACAGG

>hsa-miR-6511a-3p

CCUCACCAUCCCUUCUGCCUGC

>hsa-miR-6511b-5p

CUGCAGGCAGAAGUGGGGCUGACA

>hsa-miR-6511b-3p

CCUCACCACCCCUUCUGCCUGCA

>hsa-miR-6513-5p

UUUGGGAUUGACGCCACAUGUCU

>hsa-miR-6513-3p

UCAAGUGUCAUCUGUCCCUAG

>hsa-miR-6514-5p

UAUGGAGUGGACUUUCAGCUGGC

>hsa-miR-6514-3p

CUGCCUGUUCUUCCACUCCAG

>hsa-miR-6515-5p

UUGGAGGGUGUGGAAGACAUC

>hsa-miR-6515-3p

UCUCUUCAUCUACCCCCCAG

>hsa-miR-6516-5p

UUUGCAGUAACAGGUGUGAGCA

>hsa-miR-6516-3p

AUCAUGUAUGAUACUGCAAACA

>hsa-miR-652-5p

CAACCCUAGGAGAGGGUGCCAUUCA

>hsa-miR-652-3p

AAUGGCGCCACUAGGGUUGUG

>hsa-miR-6529-5p

GAGAGAUCAGAGGCGCAGAGUG

>hsa-miR-653-5p

GUGUUGAAACAAUCUCUACUG

>hsa-miR-654-5p

UGGUGGGCCGCAGAACAUGUGC

>hsa-miR-654-3p

UAUGUCUGCUGACCAUCACCUU

>hsa-miR-656-3p

AAUAUUAUACAGUCAACCUCU

>hsa-miR-659-5p

AGGACCUUCCCUGAACCAAGGA

>hsa-miR-659-3p

CUUGGUUCAGGGAGGGUCCCCA

>hsa-miR-660-5p

UACCCAUUGCAUAUCGGAGUUG

>hsa-miR-660-3p

ACCUCCUGUGUGCAUGGAUUA

>hsa-miR-663b

GGUGGCCCGGCCGUGCCUGAGG

>hsa-miR-664a-5p

ACUGGCUAGGGAAAAUGAUUGGAU

>hsa-miR-664a-3p

UAUUCAUUUAUCCCCAGCCUACA

>hsa-miR-664b-5p

UGGGCUAAGGGAGAUGAUUGGGUA

>hsa-miR-664b-3p

UUCAUUUGCCUCCCAGCCUACA

>hsa-miR-670-5p

GUCCCUGAGUGUAUGUGGUG

>hsa-miR-670-3p

UUUCCUCAUAUUCAUUCAGGA

>hsa-miR-671-5p

AGGAAGCCCUGGAGGGGCUGGAG

>hsa-miR-671-3p

UCCGGUUCUCAGGGCUCCACC

>hsa-miR-6716-3p

UCCGAACUCUCCAUUCCUCUGC

>hsa-miR-6717-5p

AGGCGAUGUGGGGAUGUAGAGA

>hsa-miR-6718-5p

UAGUGGUCAGAGGGCUUAUGA

>hsa-miR-6720-5p

UUCCAGCCCUGGUAGGCGCCGCG

>hsa-miR-6720-3p

CGCGCCUGCAGGAACUGGUAGA

>hsa-miR-6721-5p

UGGGCAGGGGCUUAUUGUAGGAG

>hsa-miR-6724-5p

CUGGGCCCGCGGCGGGCGUGGGG

>hsa-miR-6727-5p

CUCGGGGCAGGCGGCUGGGAGCG

>hsa-miR-6728-5p

UUGGGAUGGUAGGACCAGAGGGG

>hsa-miR-6729-5p

UGGGCGAGGGCGGCUGAGCGGC

>hsa-miR-6729-3p

UCAUCCCCCUCGCCCUCUCAG

>hsa-miR-6730-5p

AGAAAGGUGGAGGGGUUGUCAGA

>hsa-miR-6730-3p

CCUGACACCCCAUCUGCCCUCA

>hsa-miR-6731-5p

UGGGAGAGCAGGGUAUUGUGGA

>hsa-miR-6731-3p

UCUAUUCCCCACUCUCCCCAG

>hsa-miR-6732-5p

UAGGGGGUGGCAGGCUGGCC

>hsa-miR-6732-3p

UAACCCUGUCCUCUCCCUCCCAG

>hsa-miR-6733-5p

UGGGAAAGACAAACUCAGAGUU

>hsa-miR-6733-3p

UCAGUGUCUGGAUUUCCUAG

>hsa-miR-6734-5p

UUGAGGGGAGAAUGAGGUGGAGA

>hsa-miR-6734-3p

CCCUUCCCUCACUCUUCUCUCAG

>hsa-miR-6735-5p

CAGGGCAGAGGGCACAGGAAUCUGA

>hsa-miR-6735-3p

AGGCCUGUGGCUCCUCCCUCAG

>hsa-miR-6736-5p

CUGGGUGAGGGCAUCUGUGGU

>hsa-miR-6736-3p

UCAGCUCCUCUCUACCCACAG

>hsa-miR-6737-3p

UCUGUGCUUCACCCCUACCCAG

>hsa-miR-6738-5p

CGAGGGGUAGAAGAGCACAGGGG

>hsa-miR-6738-3p

CUUCUGCCUGCAUUCUACUCCCAG

>hsa-miR-6739-5p

UGGGAAAGAGAAAGAACAAGUA

>hsa-miR-6739-3p

AUUGUUCUGUCUUUCUCCCAG

>hsa-miR-6740-5p

AGUUUGGGAUGGAGAGAGGAGA

>hsa-miR-6740-3p

UGUCUUCUCUCCUCCCAAACAG

>hsa-miR-6741-3p

UCGGCUCUCUCCCUCACCCUAG

>hsa-miR-6742-5p

AGUGGGGUGGGACCCAGCUGUU

>hsa-miR-6742-3p

ACCUGGGUUGUCCCCUCUAG

>hsa-miR-6743-3p

AGCCGCUCUUCUCCCUGCCCACA

>hsa-miR-6746-5p

CCGGGAGAAGGAGGUGGCCUGG

>hsa-miR-6746-3p

CAGCCGCCGCCUGUCUCCACAG

>hsa-miR-6747-3p

UCCUGCCUUCCUCUGCACCAG

>hsa-miR-6748-5p

UGUGGGUGGGAAGGACUGGAUU

>hsa-miR-6748-3p

UCCUGUCCCUGUCUCCUACAG

>hsa-miR-6749-5p

UCGGGCCUGGGGUUGGGGGAGC

>hsa-miR-675-5p

UGGUGCGGAGAGGGCCCACAGUG

>hsa-miR-675-3p

CUGUAUGCCCUCACCGCUCA

>hsa-miR-6751-3p

ACUGAGCCUCUCUCUCUCCAG

>hsa-miR-6751-5p

UUGGGGGUGAGGUUGGUGUCUGG

>hsa-miR-6753-3p

UGGUCUGUCUCUGCCCUGGCAC

>hsa-miR-6754-5p

CCAGGGAGGCUGGUUUGGAGGA

>hsa-miR-6755-5p

UAGGGUAGACACUGACAACGUU

>hsa-miR-6755-3p

UGUUGUCAUGUUUUUUCCCUAG

>hsa-miR-6758-5p

UAGAGAGGGGAAGGAUGUGAUGU

>hsa-miR-6758-3p

ACUCAUUCUCCUCUGUCCAG

>hsa-miR-6759-5p

UUGUGGGUGGGCAGAAGUCUGU

>hsa-miR-676-3p

CUGUCCUAAGGUUGUUGAGUU

>hsa-miR-6761-5p

UCUGAGAGAGCUCGAUGGCAG

>hsa-miR-6762-5p

CGGGGCCAUGGAGCAGCCUGUGU

>hsa-miR-6762-3p

UGGCUGCUUCCCUUGGUCUCCAG

>hsa-miR-6763-5p

CUGGGGAGUGGCUGGGGAG

>hsa-miR-6764-5p

UCCCAGGGUCUGGUCAGAGUUG

>hsa-miR-6764-3p

UCUCUGGUCUUUCCUUGACAG

>hsa-miR-6765-3p

UCACCUGGCUGGCCCGCCCAG

>hsa-miR-6766-5p

CGGGUGGGAGCAGAUCUUAUUGAG

>hsa-miR-6766-3p

UGAUUGUCUUCCCCCACCCUCA

>hsa-miR-6767-5p

UCGCAGACAGGGACACAUGGAGA

>hsa-miR-6767-3p

CCACGUGCUUCUCUUUCCGCAG

>hsa-miR-6769a-3p

GAGCCCCUCUCUGCUCUCCAG

>hsa-miR-6769b-5p

UGGUGGGUGGGGAGGAGAAGUGC

>hsa-miR-6769b-3p

CCCUCUCUGUCCCACCCAUAG

>hsa-miR-6770-5p

UGAGAAGGCACAGCUUGCACGUGA

>hsa-miR-6770-3p

CUGGCGGCUGUGUCUUCACAG

>hsa-miR-6771-5p

CUCGGGAGGGCAUGGGCCAGGC

>hsa-miR-6772-3p

UUGCUCCUGACUCUGUGCCCACA

>hsa-miR-6774-5p

ACUUGGGCAGGAGGGACCCUGUAUG

>hsa-miR-6774-3p

UCGUGUCCCUCUUGUCCACAG

>hsa-miR-6775-3p

AGGCCCUGUCCUCUGCCCCAG

>hsa-miR-6776-5p

UCUGGGUGCAGUGGGGGUU

>hsa-miR-6776-3p

CAACCACCACUGUCUCUCCCCAG

>hsa-miR-6777-5p

ACGGGGAGUCAGGCAGUGGUGGA

>hsa-miR-6777-3p

UCCACUCUCCUGGCCCCCAG

>hsa-miR-6779-5p

CUGGGAGGGGCUGGGUUUGGC

>hsa-miR-6779-3p

AAGCCCUGUCUCCUCCCAUCU

>hsa-miR-6780a-5p

UUGGGAGGGAAGACAGCUGGAGA

>hsa-miR-6780a-3p

CUCCUCUGUUUUCUUUCCUAG

>hsa-miR-6780b-3p

UCCCUUGUCUCCUUUCCCUAG

>hsa-miR-6781-5p

CGGGCCGGAGGUCAAGGGCGU

>hsa-miR-6781-3p

UGCCUCUUUUCCACGGCCUCAG

>hsa-miR-6782-5p

UAGGGGUGGGGGAAUUCAGGGGUGU

>hsa-miR-6782-3p

CACCUUUGUGUCCCCAUCCUGCA

>hsa-miR-6783-5p

UAGGGGAAAAGUCCUGAUCCGG

>hsa-miR-6783-3p

UUCCUGGGCUUCUCCUCUGUAG

>hsa-miR-6785-5p

UGGGAGGGCGUGGAUGAUGGUG

>hsa-miR-6786-5p

GCGGUGGGGCCGGAGGGGCGU

>hsa-miR-6786-3p

UGACGCCCCUUCUGAUUCUGCCU

>hsa-miR-6787-3p

UCUCAGCUGCUGCCCUCUCCAG

>hsa-miR-6788-3p

UUCGCCACUUCCCUCCCUGCAG

>hsa-miR-6789-3p

CGGCGCCCGUGUCUCCUCCAG

>hsa-miR-6790-5p

GUGAGUGUGGAUUUGGCGGGGUU

>hsa-miR-6791-3p

UGCCUCCUUGGUCUCCGGCAG

>hsa-miR-6793-5p

UGUGGGUUCUGGGUUGGGGUGA

>hsa-miR-6793-3p

UCCCCAACCCCUGCCCGCAG

>hsa-miR-6795-5p

UGGGGGGACAGGAUGAGAGGCUGU

>hsa-miR-6796-5p

UUGUGGGGUUGGAGAGCUGGCUG

>hsa-miR-6796-3p

GAAGCUCUCCCCUCCCCGCAG

>hsa-miR-6797-5p

AGGAGGGAAGGGGCUGAGAACAGGA

>hsa-miR-6797-3p

UGCAUGACCCUUCCCUCCCCAC

>hsa-miR-6798-3p

CUACCCCCCAUCCCCCUGUAG

>hsa-miR-6799-5p

GGGGAGGUGUGCAGGGCUGG

>hsa-miR-6799-3p

UGCCCUGCAUGGUGUCCCCACAG

>hsa-miR-6800-3p

CACCUCUCCUGGCAUCGCCCC

>hsa-miR-6801-3p

ACCCCUGCCACUCACUGGCC

>hsa-miR-6802-5p

CUAGGUGGGGGGCUUGAAGC

>hsa-miR-6802-3p

UUCACCCCUCUCACCUAAGCAG

>hsa-miR-6803-3p

UCCCUCGCCUUCUCACCCUCAG

>hsa-miR-6804-5p

UGAGGGUGUCAGCAGGUGACG

>hsa-miR-6804-3p

CGCACCUGCCUCUCACCCACAG

>hsa-miR-6805-5p

UAGGGGGCGGCUUGUGGAGUGU

>hsa-miR-6806-3p

UGAAGCUCUGACAUUCCUGCAG

>hsa-miR-6808-3p

GUGUGACCACCGUUCCUGCAG

>hsa-miR-6810-5p

AUGGGGACAGGGAUCAGCAUGGC

>hsa-miR-6810-3p

UCCCCUGCUCCCUUGUUCCCCAG

>hsa-miR-6812-5p

AUGGGGUGAGAUGGGGAGGAGCAGC

>hsa-miR-6812-3p

CCGCUCUUCCCCUGACCCCAG

>hsa-miR-6813-5p

CAGGGGCUGGGGUUUCAGGUUCU

>hsa-miR-6815-5p

UAGGUGGCGCCGGAGGAGUCAUU

>hsa-miR-6817-3p

UCUCUCUGACUCCAUGGCA

>hsa-miR-6818-5p

UUGUGUGAGUACAGAGAGCAUC

>hsa-miR-6818-3p

UUGUCUCUUGUUCCUCACACAG

>hsa-miR-6819-5p

UUGGGGUGGAGGGCCAAGGAGC

>hsa-miR-6819-3p

AAGCCUCUGUCCCCACCCCAG

>hsa-miR-6820-5p

UGCGGCAGAGCUGGGGUCA

>hsa-miR-6820-3p

UGUGACUUCUCCCCUGCCACAG

>hsa-miR-6821-5p

GUGCGUGGUGGCUCGAGGCGGGG

>hsa-miR-6822-5p

CAGGGAACCAGUUGGGGCUU

>hsa-miR-6823-5p

UCAGGGUUGGUAGGGGUUGCU

>hsa-miR-6823-3p

UGAGCCUCUCCUUCCCUCCAG

>hsa-miR-6824-5p

GUAGGGGAGGUUGGGCCAGGGA

>hsa-miR-6824-3p

UCUCUGGUCUUGCCACCCCAG

>hsa-miR-6825-5p

UGGGGAGGUGUGGAGUCAGCAU

>hsa-miR-6825-3p

GCGCUGACCCGCCUUCUCCGCA

>hsa-miR-6826-5p

UCAAUAGGAAAGAGGUGGGACCU

>hsa-miR-6826-3p

CUCCCCUCUCUUUCCUGUUCAG

>hsa-miR-6832-5p

AGUAGAGAGGAAAAGUUAGGGUC

>hsa-miR-6832-3p

ACCCUUUUUCUCUUUCCCAG

>hsa-miR-6833-3p

UUUCUCUCUCCACUUCCUCAG

>hsa-miR-6835-5p

AGGGGGUAGAAAGUGGCUGAAG

>hsa-miR-6835-3p

AAAAGCACUUUUCUGUCUCCCAG

>hsa-miR-6837-5p

ACCAGGGCCAGCAGGGAAUGU

>hsa-miR-6837-3p

CCUUCACUGUGACUCUGCUGCAG

>hsa-miR-6838-3p

AAGUCCUGCUUCUGUUGCAG

>hsa-miR-6838-5p

AAGCAGCAGUGGCAAGACUCCU

>hsa-miR-6839-5p

UCUGGAUUGAAGAGACGACCCA

>hsa-miR-6840-5p

ACCCCCGGGCAAAGACCUGCAGAU

>hsa-miR-6842-5p

UGGGGGUGGUCUCUAGCCAAGG

>hsa-miR-6842-3p

UUGGCUGGUCUCUGCUCCGCAG

>hsa-miR-6844

UUCUUUGUUUUUAAUUCACAG

>hsa-miR-6846-5p

UGGGGGCUGGAUGGGGUAGAGU

>hsa-miR-6846-3p

UGACCCCUUCUGUCUCCCUAG

>hsa-miR-6847-5p

ACAGAGGACAGUGGAGUGUGAGC

>hsa-miR-6847-3p

GGCUCAUGUGUCUGUCCUCUUC

>hsa-miR-6848-5p

UGGGGGCUGGGAUGGGCCAUGGU

>hsa-miR-6851-5p

AGGAGGUGGUACUAGGGGCCAGC

>hsa-miR-6851-3p

UGGCCCUUUGUACCCCUCCAG

>hsa-miR-6852-5p

CCCUGGGGUUCUGAGGACAUG

>hsa-miR-6853-5p

AGCGUGGGAUGUCCAUGAAGUCAG

>hsa-miR-6853-3p

UGUUCAUUGGAACCCUGCGCAG

>hsa-miR-6854-5p

AAGCUCAGGUUUGAGAACUGCUGA

>hsa-miR-6854-3p

UGCGUUUCUCCUCUUGAGCAG

>hsa-miR-6855-5p

UUGGGGUUUGGGGUGCAGACAUUGC

>hsa-miR-6855-3p

AGACUGACCUUCAACCCCACAG

>hsa-miR-6857-5p

UUGGGGAUUGGGUCAGGCCAGU

>hsa-miR-6857-3p

UGACUGAGCUUCUCCCCACAG

>hsa-miR-6858-5p

GUGAGGAGGGGCUGGCAGGGAC

>hsa-miR-6859-5p

GAGAGGAACAUGGGCUCAGGACA

>hsa-miR-6859-3p

UGACCCCCAUGUCGCCUCUGUAG

>hsa-miR-6860

ACUGGGCAGGGCUGUGGUGAGU

>hsa-miR-6862-5p

CGGGCAUGCUGGGAGAGACUUU

>hsa-miR-6862-3p

CCUCACCCAGCUCUCUGGCCCUCU

>hsa-miR-6863

UAGACGUGGUGAAGGAUUGAGUG

>hsa-miR-6865-5p

UAGGUGGCAGAGGAGGGACUUCA

>hsa-miR-6865-3p

ACACCCUCUUUCCCUACCGCC

>hsa-miR-6866-5p

UUAGAGGCUGGAAUAGAGAUUCU

>hsa-miR-6867-5p

UGUGUGUGUAGAGGAAGAAGGGA

>hsa-miR-6867-3p

CUCUCCCUCUUUACCCACUAG

>hsa-miR-6868-3p

UUCCUUCUGUUGUCUGUGCAG

>hsa-miR-6869-5p

GUGAGUAGUGGCGCGCGGCGGC

>hsa-miR-6870-3p

GCUCAUCCCCAUCUCCUUUCAG

>hsa-miR-6873-3p

UUCUCUCUGUCUUUCUCUCUCAG

>hsa-miR-6874-3p

CAGUUCUGCUGUUCUGACUCUAG

>hsa-miR-6875-3p

AUUCUUCCUGCCCUGGCUCCAU

>hsa-miR-6876-5p

CAGGAAGGAGACAGGCAGUUCA

>hsa-miR-6876-3p

AGCUGUCUGUGUUUUCCUUCUCAG

>hsa-miR-6877-5p

AGGGCCGAAGGGUGGAAGCUGC

>hsa-miR-6878-5p

AGGGAGAAAGCUAGAAGCUGAAG

>hsa-miR-6881-5p

UGGGGUAAGGAUAGGAGGGUCA

>hsa-miR-6881-3p

AUCCUCUUUCGUCCUUCCCACU

>hsa-miR-6882-5p

UACAAGUCAGGAGCUGAAGCAG

>hsa-miR-6882-3p

UGCUGCCUCUCCUCUUGCCUGCAG

>hsa-miR-6883-3p

UUCCCUAUCUCACUCUCCUCAG

>hsa-miR-6884-5p

AGAGGCUGAGAAGGUGAUGUUG

>hsa-miR-6885-5p

AGGGGGGCACUGCGCAAGCAAAGCC

>hsa-miR-6885-3p

CUUUGCUUCCUGCUCCCCUAG

>hsa-miR-6886-3p

UGCCCUUCUCUCCUCCUGCCU

>hsa-miR-6891-5p

UAAGGAGGGGGAUGAGGGG

>hsa-miR-6891-3p

CCCUCAUCUUCCCCUCCUUUC

>hsa-miR-6892-5p

GUAAGGGACCGGAGAGUAGGA

>hsa-miR-6894-5p

AGGAGGAUGGAGAGCUGGGCCAGA

>hsa-miR-6894-3p

UUGCCUGCCCUCUUCCUCCAG

>hsa-miR-6895-3p

UGUCUCUCGCCCUUGGCCUUAG

>hsa-miR-7-5p

UGGAAGACUAGUGAUUUUGUUGUU

>hsa-miR-7-1-3p

CAACAAAUCACAGUCUGCCAUA

>hsa-miR-7-2-3p

CAACAAAUCCCAGUCUACCUAA

>hsa-miR-708-5p

AAGGAGCUUACAAUCUAGCUGGG

>hsa-miR-708-3p

CAACUAGACUGUGAGCUUCUAG

>hsa-miR-7108-5p

GUGUGGCCGGCAGGCGGGUGG

>hsa-miR-7109-3p

CAAGCCUCUCCUGCCCUUCCAG

>hsa-miR-7110-5p

UGGGGGUGUGGGGAGAGAGAG

>hsa-miR-7110-3p

UCUCUCUCCCACUUCCCUGCAG

>hsa-miR-7111-5p

UGGGGGAGGAAGGACAGGCCAU

>hsa-miR-7111-3p

AUCCUCUCUUCCCUCCUCCCAG

>hsa-miR-7113-5p

UCCAGGGAGACAGUGUGUGAG

>hsa-miR-7114-5p

UCUGUGGAGUGGGGUGCCUGU

>hsa-miR-7114-3p

UGACCCACCCCUCUCCACCAG

>hsa-miR-7151-5p

GAUCCAUCUCUGCCUGUAUUGGC

>hsa-miR-7151-3p

CUACAGGCUGGAAUGGGCUCA

>hsa-miR-7152-5p

UUUCCUGUCCUCCAACCAGACC

>hsa-miR-7152-3p

UCUGGUCCUGGACAGGAGGC

>hsa-miR-7160-3p

CAGGGCCCUGGCUUUAGCAGA

>hsa-miR-744-5p

UGCGGGGCUAGGGCUAACAGCA

>hsa-miR-744-3p

CUGUUGCCACUAACCUCAACCU

>hsa-miR-758-3p

UUUGUGACCUGGUCCACUAACC

>hsa-miR-760

CGGCUCUGGGUCUGUGGGGA

>hsa-miR-765

UGGAGGAGAAGGAAGGUGAUG

>hsa-miR-766-5p

AGGAGGAAUUGGUGCUGGUCUU

>hsa-miR-766-3p

ACUCCAGCCCCACAGCCUCAGC

>hsa-miR-767-5p

UGCACCAUGGUUGUCUGAGCAUG

>hsa-miR-769-5p

UGAGACCUCUGGGUUCUGAGCU

>hsa-miR-769-3p

CUGGGAUCUCCGGGGUCUUGGUU

>hsa-miR-7702

CUUAGACUGCCAGACUCCCUGA

>hsa-miR-7703

UUGCACUCUGGCCUUCUCCCAGG

>hsa-miR-7704

CGGGGUCGGCGGCGACGUG

>hsa-miR-7705

AAUAGCUCAGAAUGUCAGUUCUG

>hsa-miR-7706

UGAAGCGCCUGUGCUCUGCCGAGA

>hsa-miR-7845-5p

AAGGGACAGGGAGGGUCGUGG

>hsa-miR-7848-3p

CUACCCUCGGUCUGCUUACCACA

>hsa-miR-7851-3p

UACCUGGGAGACUGAGGUUGGA

>hsa-miR-7854-3p

UGAGGUGACCGCAGAUGGGAA

>hsa-miR-7974

AGGCUGUGAUGCUCUCCUGAGCCC

>hsa-miR-7976

UGCCCUGAGACUUUUGCUC

>hsa-miR-7977

UUCCCAGCCAACGCACCA

>hsa-miR-8485

CACACACACACACACACGUAU

>hsa-miR-873-5p

GCAGGAACUUGUGAGUCUCCU

>hsa-miR-874-5p

CGGCCCCACGCACCAGGGUAAGA

>hsa-miR-874-3p

CUGCCCUGGCCCGAGGGACCGA

>hsa-miR-875-5p

UAUACCUCAGUUUUAUCAGGUG

>hsa-miR-877-5p

GUAGAGGAGAUGGCGCAGGG

>hsa-miR-877-3p

UCCUCUUCUCCCUCCUCCCAG

>hsa-miR-885-5p

UCCAUUACACUACCCUGCCUCU

>hsa-miR-885-3p

AGGCAGCGGGGUGUAGUGGAUA

>hsa-miR-887-3p

GUGAACGGGCGCCAUCCCGAGG

>hsa-miR-888-5p

UACUCAAAAAGCUGUCAGUCA

>hsa-miR-889-3p

UUAAUAUCGGACAACCAUUGU

>hsa-miR-891a-5p

UGCAACGAACCUGAGCCACUGA

>hsa-miR-892a

CACUGUGUCCUUUCUGCGUAG

>hsa-miR-892b

CACUGGCUCCUUUCUGGGUAGA

>hsa-miR-9-5p

UCUUUGGUUAUCUAGCUGUAUGA

>hsa-miR-9-3p

AUAAAGCUAGAUAACCGAAAGU

>hsa-miR-92a-1-5p

AGGUUGGGAUCGGUUGCAAUGCU

>hsa-miR-92a-3p

UAUUGCACUUGUCCCGGCCUGU

>hsa-miR-92b-5p

AGGGACGGGACGCGGUGCAGUG

>hsa-miR-92b-3p

UAUUGCACUCGUCCCGGCCUCC

>hsa-miR-93-5p

CAAAGUGCUGUUCGUGCAGGUAG

>hsa-miR-93-3p

ACUGCUGAGCUAGCACUUCCCG

>hsa-miR-933

UGUGCGCAGGGAGACCUCUCCC

>hsa-miR-935

CCAGUUACCGCUUCCGCUACCGC

>hsa-miR-937-5p

GUGAGUCAGGGUGGGGCUGG

>hsa-miR-937-3p

AUCCGCGCUCUGACUCUCUGCC

>hsa-miR-939-5p

UGGGGAGCUGAGGCUCUGGGGGUG

>hsa-miR-939-3p

CCCUGGGCCUCUGCUCCCCAG

>hsa-miR-940

AAGGCAGGGCCCCCGCUCCCC

>hsa-miR-941

CACCCGGCUGUGUGCACAUGUGC

>hsa-miR-942-5p

UCUUCUCUGUUUUGGCCAUGUG

>hsa-miR-942-3p

CACAUGGCCGAAACAGAGAAGU

>hsa-miR-943

CUGACUGUUGCCGUCCUCCAG

>hsa-miR-944

AAAUUAUUGUACAUCGGAUGAG

>hsa-miR-95-5p

UCAAUAAAUGUCUGUUGAAUU

>hsa-miR-95-3p

UUCAACGGGUAUUUAUUGAGCA

>hsa-miR-96-5p

UUUGGCACUAGCACAUUUUUGCU

>hsa-miR-96-3p

AAUCAUGUGCAGUGCCAAUAUG

>hsa-miR-98-5p

UGAGGUAGUAAGUUGUAUUGUU

>hsa-miR-98-3p

CUAUACAACUUACUACUUUCCC

>hsa-miR-9901

CGGUCGCCGCGGUUCGCCGCC

>hsa-miR-9903

UUAUCCUCCAGUAGACUAGGGA

>hsa-miR-9985

UUCACAGUGGCUAAGCUAU

>hsa-miR-99a-5p

AACCCGUAGAUCCGAUCUUGUG

>hsa-miR-99b-5p

CACCCGUAGAACCGACCUUGCG

>hsa-miR-99b-3p

CAAGCUCGUGUCUGUGGGUCCG
